# Identification of a Novel Prognostic Gene Signature From the Immune Cell Infiltration Landscape of Osteosarcoma

**DOI:** 10.3389/fcell.2021.718624

**Published:** 2021-09-06

**Authors:** Lei Fan, Jingtao Ru, Tao Liu, Chao Ma

**Affiliations:** ^1^Department of Orthopedics, Henan Provincial People’s Hospital, People’s Hospital of Zhengzhou University, Zhengzhou, China; ^2^Charité – Universitätsmedizin Berlin, Berlin, Germany

**Keywords:** osteosarcoma, immune cell infiltration, ICI, tumor microenvironment, gene signature

## Abstract

**Background:** The tumor microenvironment (TME) mainly comprises tumor cells and tumor-infiltrating immune cells mixed with stromal components. Latestresearch hasdisplayed that tumor immune cell infiltration (ICI) is associated with the clinical outcome of patients with osteosarcoma (OS). This work aimed to build a gene signature according to ICI in OS for predicting patient outcomes.

**Methods:** The TARGET-OS dataset was used for model training, while the GSE21257 dataset was taken forvalidation. Unsupervised clustering was performed on the training cohort based on the ICI profiles. The Kaplan–Meier estimator and univariate Cox proportional hazards models were used to identify the differentially expressed genes between clusters to preliminarily screen for potential prognostic genes. We incorporated these potential prognostic genes into a LASSO regression analysis and produced a gene signature, which was next assessed with the Kaplan–Meier estimator, Cox proportional hazards models, ROC curves, IAUC, and IBS in the training and validation cohorts. In addition, we compared our signature to previous models. GSEAswere deployed to further study the functional mechanism of the signature. We conducted an analysis of 22 TICsfor identifying the role of TICs in the gene signature’s prognosis ability.

**Results:** Data from the training cohort were used to generate a nine-gene signature. The Kaplan–Meier estimator, Cox proportional hazards models, ROC curves, IAUC, and IBS validated the signature’s capacity and independence in predicting the outcomes of OS patients in the validation cohort. A comparison with previous studies confirmed the superiority of our signature regarding its prognostic ability. Annotation analysis revealed the mechanism related to the gene signature specifically. The immune-infiltration analysis uncoveredkey roles for activated mast cells in the prognosis of OS.

**Conclusion:** We identified a robust nine-gene signature (ZFP90, UHRF2, SELPLG, PLD3, PLCB4, IFNGR1, DLEU2, ATP6V1E1, and ANXA5) that can predict OS outcome precisely and is strongly linked to activated mast cells.

## Introduction

Osteosarcoma (OS) is a rare malignant tumor that mainly affects children and adolescents (Yang et al., 2021). Since the introduction of chemotherapy in 1970, the 5-year survival rate of patients with non-metastatic OS has reached 70% (Li et al., 2021). In addition, most patients can receive limb salvage surgery and obtain proper postoperative limb function (Li et al., 2021). Despite advances in surgical techniques, multi-agent systemic chemotherapy, precise radiotherapy, and immunotherapy, the 5-year survival rate of a localized tumor remains at 60%–70%, while that of metastasis and recurrence is less than 20% (Zhang H. et al., 2019). Therefore, studying the molecular mechanism of the occurrence and development of osteosarcoma, looking for new possible molecular therapeutic targets and prognostic criteria has become a key measure to improve the prognosis of patients with osteosarcoma (Yiqi et al., 2020).

Recently, attention has been paid to the tumor microenvironment (TME), which plays a vital role in the occurrence and development of cancer. Tumor microenvironment comprises tumor cells, fibroblasts, endothelial cells, immune cells, various signal molecules, and extracellular matrix (Cortini et al., 2016; Chen Y. et al., 2020; Luo et al., 2020). Osteosarcomasare inextricably linked to their local TME, composed of bone, stromal, vascular, and immune cells (Corre et al., 2020). The OS TME is now considered to be essential and supportive for growth and dissemination (Corre et al., 2020). One latest study has shown that tumor immune cell infiltration (ICI)is associated with the clinical outcomes of OS patients (Chen Y. et al., 2020). Extensive research on the TME has shown that infiltrating immune cells play a vital role in tumor spread, recurrence, metastasis, and the response to immunotherapy (Ma et al., 2020; Zhang X. et al., 2020). However, the detailed profile of immune cells infiltrating in OS has not been elucidated (Zhang X. et al., 2020).

Previous studieshaveprimarily focused on one or two kinds of immune cells or key genes, which could bias OS microenvironment exploration. The identification of multiple genes from tumor-infiltrating immune cell profiles can help to construct a gene signature with better and more accurate prognostic potential. To fill in the gaps and find potential diagnostic approaches targeting OS prognosis, this work clustered patients based on the ICI content to identify a prognostic gene signature. More importantly, the signature was validated in another independent cohort and compared to previous prognosis models. Also, the functional annotation and analysis of 22 tumor-infiltrating immune cells (TICs) were performed to further study the gene signature.

## Materials and Methods

### Mining Public Databases

The Therapeutically Applicable Research to Generate Effective Treatments (TARGET) is an open childhood cancer database that aims to use comprehensive genomic methods to identify molecular changes in the occurrence and development of difficult-to-treat childhood cancers (Gao et al., 2020; Zhang F. et al., 2020). One project named TARGET-OS (*n* = 88) in the TARGET database was treated as the training cohort, and the level 3 gene expression data and clinical characteristics of OS cases were downloadedon the GDC Xena Hub.^1^ The GSE21257 dataset was downloaded from the Gene Expression Omnibus (GEO) database (Buddingh et al., 2011) and was taken as the validation cohort. This dataset includes data from 53 unique diagnostic biopsy specimens analyzed on the GPL10295 platform (Illumina human-6 v2.0 expression beadchip).

### Consensus Clustering for TICs

CIBERSORT can describe the cell composition of complex tissues based on the gene expression profile of complex tissues. It uses linear support vector regression (a machine learning method) to deconvolute a mixture of gene expressions (Newman et al., 2015, 2019; Thorsson et al., 2018). The infiltration levels of distinct immune cells in OS patients in the training cohort were quantified by using the “CIBERSORT” R package and employing the 22 TIC signature and 1,000 permutations (Newman et al., 2015, 2019; Thorsson et al., 2018). The ESTIMATE (Estimation of STromal and Immune cells in MAlignant Tumor tissues using Expression data) algorithm provides researchers with a score of tumor purity, stromal cell presence level, and ICI level in tumor tissue based on expression data (Yoshihara et al., 2013). The “ESTIMATE” R package was used to assess the immune score and stromal score for OSsusing the gene expression data from the training cohort. The combination of CIBERSORT and ESTIMATE results from each OS sample was defined as the tumor ICI pattern. The “ConsensusClusterPlus” R package was used to cluster the samples according to the ICI pattern of each sample (repeat time = 1000). Pam and Euclidean distances were used as the clustering algorithm and distance measure, respectively.

### Differentially Expressed Genes (DEGs) Between ICI Phenotypes

This section tries to study genes associated with the ICI patterns. Based on the ICI cluster we have produced, DEGs were identified between the ICI clusters using the “limma” R package (Ritchie et al., 2015), with cutoffs of | log2(fold-change)| > 0.2 and p-value < 0.05.

### Gene Signature Construction and Validation

The Kaplan–Meier estimator and univariate Cox proportional hazards model were adopted to identify the potential prognostic DEGs using the data of the training cohort. Genes with p-values < 0.05 in both tests were considered to be potential prognostic genes. To minimize the risk of overfitting, the LASSO Cox regression analysis was used to construct a prognostic model. The LASSO algorithm was run using the “glmnet” R package for variable selection and shrinkage of these identified potential prognostic genes (Tibshirani, 1997;Sauerbrei et al., 2007; Friedman et al., 2010; Goeman, 2010). The “glmnet” R package outputted genes with coefficients. The risk score of each patient could be obtained according to the following formula:

$$Riskscore=\sum_{i}^{n}Expi*\beta i$$

(n: hub genes; Expi: gene expression level; βi: coefficient).

Osteosarcoma (OS) patients were classified into high-risk or low-risk groups according to the median risk score. The Kaplan–Meier estimator was used to validate the survival difference between high- and low-risk groups. Univariate and multivariate Cox proportional hazards models were applied to test the prognostic ability of the gene signature we constructed. Additionally, ROC curve analysis, integrated AUC analysis (IAUC, also known as time-dependent AUC), and integrated Brier score analysis (IBS, also known as time-dependent BS) were performed to confirm the predictive capacity of the signature.

### Comparison of the Gene Signature With Previously Published Models

We searched PubMed^2^ using the keyword “gene signature prognosis osteosarcoma” and set the following screening criteria: (1) the impact factor of the journal was greater than 4, and (2) the online publication date was in the most recent year, from May 18, 2020, to May 18, 2021. After finding suitable studies, the gene signatures were extracted. We applied these signatures to the training cohort to obtain coefficients *via* the multivariate Cox proportional hazards model. We adopted the Kaplan–Meier estimator and Cox proportional hazards models to test these signatures in the training and validation cohorts to compare the prognostic ability difference between previous studies and ours.

### Gene Set Enrichment Analysis (GSEA)

GSEA is a computational method used to determine whether an *a priori* defined gene set shows a statistically significant difference between two biological states (Subramanian et al., 2005). In our study, GSEA was run using Hallmark gene set collections (v7.4^3^) *via* GSEA software (v4.1.0 for Windows^4^) to reveal potential mechanisms of gene signature in OS prognosis. In this analysis, the significant cutoff should meet all the following indicators: | NES | > 1, NOM p-value (nominal p-value) < 0.05, and FD R q-value < 0.25.

### Identification of Relationships Between the Gene Signature and 22 TICs

We used the CIBERSORT algorithm for the calculation of the relative contents of 22 TICs in the training cohort (Newman et al., 2015, 2019; Thorsson et al., 2018). For identifying the inner relationships among the 22 TICs, the Pearson coefficient was equipped for the analysis. Also, for obtaining the correlations between the 22 TICs and the signature, we adopt comprehensive analyses, including the Spearman coefficient and Wilcoxon rank-sum test. We used the univariate and multivariate Cox proportional hazards models and the Kaplan–Meier estimator to test 22 TICs to know which kind of TICs were impacting the OS prognosis. In this section, we learned about the relationships between 22 TICs and the gene signature and the prognostic ability of 22 TICs. Combining the above evidence, potential candidate TICs could be inferred, which played a vital role in the prognosis of the gene signature.

### Statistical Analysis

We used the “glmnet” R package for performing the LASSO regression analysis. A Kaplan–Meier estimator was built by applying the “survival” and “survminer” R packages. Additionally, the “survival” R package was used to build Cox proportional hazards models. The “timeROC” and “survival” R packages were used to plot the ROC curves and call the IAUC analysis. Moreover, the “Rcpp,” “ranger,” and “survival” R packages were used to calculate the IBS. All the processes were run in R software (version 4.0.4, 2021-02-15). In the present study, a p-value < 0.05 indicates statistical significance.

## Results

### Patient Characteristics

**FIGURE 1:**
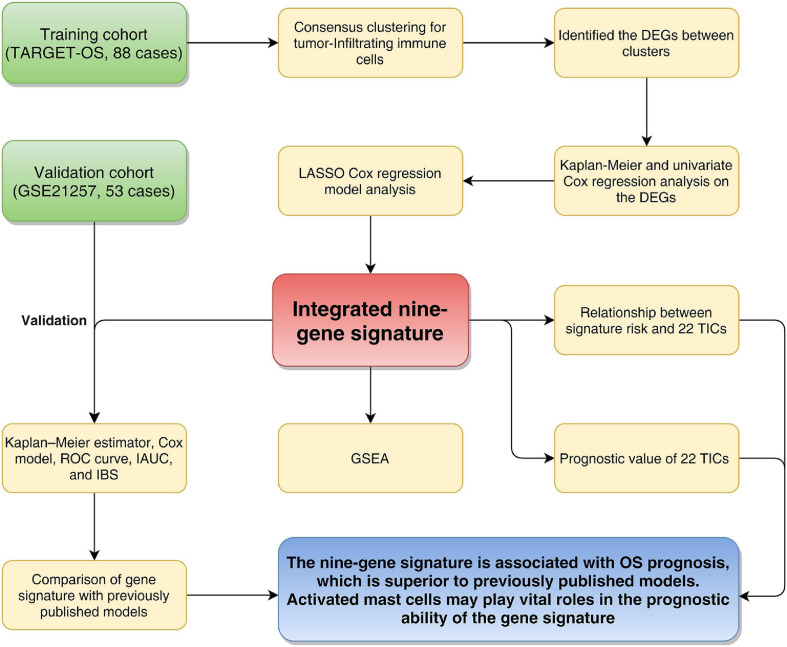
Flowchart of the study. TARGET: therapeutically applicable research to generate effective treatments; OS, osteosarcoma; DEGs, differentially expressed genes; LASSO, least absolute shrinkage and selection operator Cox regression model; AUC, area under the ROC curve; IAUC, integrated AUC; ROC, receiver operating characteristic; IBS, integrated Brier score; GSEA, Gene Set Enrichment Analysis; TICs, tumor-infiltrating immune cells.

Figure 1 shows the flowchart of this study. Eighty-eight OS patients from the TARGET-OS cohort were used to train the model. The dataset GSE21257, containing 53 OS cases, was selected to validate the model. We collected the clinical characteristics of the OS patients in these two cohorts and showed them in Table 1 in detail.

**TABLE 1:** Clinical characteristics of patients involved in the study.

**Characteristics**		**Training cohort (TARGET-OS, *n* = 88)**	**Validation cohort (GSE21257, *n* = 53)**
**Age**			
	< 14	39(44.32%)	15 (28.3%)
	=14	45(51.14%)	38 (71.7%)
	Unknown	4 (4.55%)	0
**Gender**			
	Female	37 (42.05%)	19 (35.85%)
	Male	47 (53.41%)	34 (64.15%)
	Unknown	4 (4.55%)	0
**Race**			
	Non-White	13 (14.77%)	NA
	White	51 (57.95%)	NA
	Unknown	24 (27.27%)	NA
**Ethnicity**			
	Not Hispanic or Latino	52 (59.09%)	NA
	Hispanic or Latino	11 (12.5%)	NA
	Unknown	25 (28.41%)	NA
**Tumor location**			
	Femur	NA	27 (50.94%)
	Fibula	NA	2 (3.77%)
	Humerus	NA	8 (15.09%)
	Tibia	NA	15 (28.3%)
	Unknown	NA	1 (1.89%)
**Histological subtype**			
	Chondroblastic	NA	6 (11.32%)
	Fibroblastic	NA	5 (9.43%)
	Osteoblastic	NA	32 (60.38%)
	Others	NA	10 (18.87%)
**Metastatic status**			
	Non-metastatic	63 (71.59%)	39 (73.58%)
	Metastatic	21 (23.86%)	14 (26.42%)
	Unknown	4 (4.55%)	0
**Survival status**			
	Alive	58 (65.91%)	30 (56.6%)
	Dead	27 (30.68%)	23 (43.4%)
	Unknown	3 (3.41%)	0

### Consensus Clustering for TICs

**FIGURE 2:**
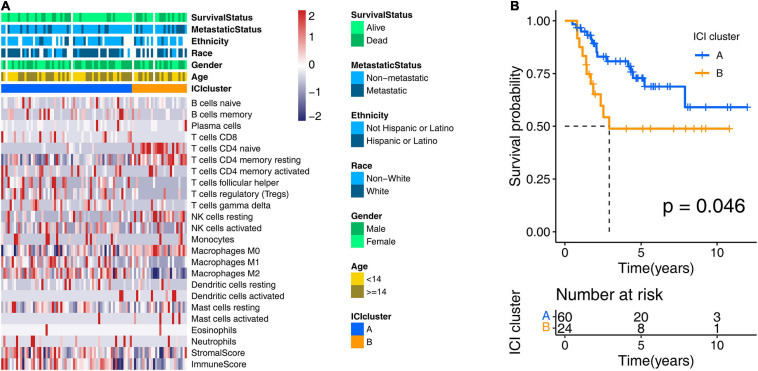
Consensus clustering for tumor-infiltrating immune cells. **(A)** Two ICI clusters were generated from unsupervised clustering of the ICI profile data of the training cohort. Rows represent ICI types, and columns represent OS samples. **(B)** Overall survival-based Kaplan–Meier estimator of two ICI clusters in the training cohort. The log-rank test showed an overall *p* = 0.046. ICI, immune cell infiltration; OS, osteosarcoma.

To obtain the ICI profile of each OS sample, we adopted the CIBERSORT and ESTIMATE algorithms. The CIBERSORT algorithm could output the relative content of 22 TICs for each patient, and the ESTIMATE algorithm calculated the immune score and stromal score. Combining the results above, we got the ICI profiles for all samples. We found two independent ICI subtypes (Figure 2A and Supplementary Figure 1) from the clustering results generated from the “ConsesusClusterPlus” R package, which runs based on the tumor samples with matched ICI profiles in the training cohort. More importantly, in the subsequent Kaplan–Meier analysis, we discovered significant survival differences (log-rank test, *p* = 0.046; Figure 2B) between these two subgroups. In detail, ICI cluster A correlated with a good prognosis, while ICI cluster B witnessed a shorter overall survival.

### Prognostic Gene Signature Identification

**FIGURE 3:**
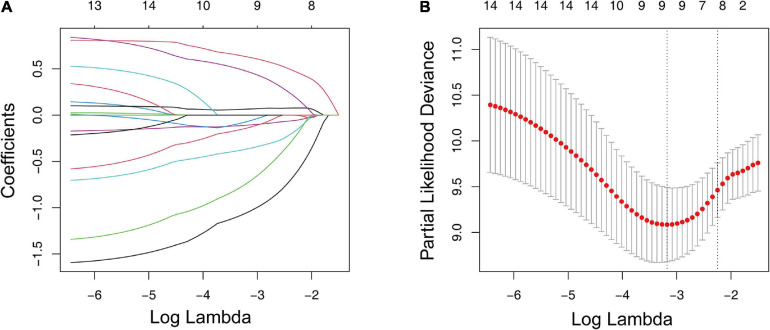
LASSO regression analysis for the construction of a prognostic gene signature. **(A)** Cross-validation for tuning parameter screening upon LASSO regression analysis. **(B)** Screening of the optimal parameter (lambda) at which the vertical lines were drawn. LASSO, the least absolute shrinkage and selection operator Cox regression model.

We conducted a differential analysis using the “limma” R package to determine the transcriptome variation between the two ICI clusters to reveal the potential biological characteristics of different immunophenotypes. A total of 4,501 DEGs were found (Supplementary Table 1). In the subsequent analysis, a Kaplan–Meier estimator and univariate Cox proportional hazards model were constructed to test the DEGs’ prognostic capacities. We only treated the DEGs with a p-value < 0.05 in both tests as potential prognostic genes. There were 15 genes that passed our tests and were determined as potential prognostic genes (Supplementary Table 2). We then put them into an overall survival-based Cox model with Lasso regression (Figure 3A) for further shrinkage and selection. The Lasso algorithm outputted the result, showing that it could achieve the optimal performance when the model had nine genes (Figure 3B). The regression coefficient of each gene was generated by the “glmnet” R package and is shown inTable 2.

**TABLE 2:** Prognostic genes obtained from the LASSO Cox regression model.

**Gene symbol**	**Description**	**Risk coefficient**
ZFP90	ZFP90 Zinc Finger Protein	−1.050029729
UHRF2	Ubiquitin Like With PHD And Ring Finger Domains 2	0.628646168
SELPLG	Selectin P Ligand	−0.364076723
PLD3	Phospholipase D Family Member 3	−0.108140251
PLCB4	Phospholipase C Beta 4	0.064875459
IFNGR1	Interferon Gamma Receptor 1	−0.067753129
DLEU2	Deleted In Lymphocytic Leukemia 2	0.470586047
ATP6V1E1	ATPase H+ Transporting V1 Subunit E1	−0.137457373
ANXA5	Annexin A5	−0.730741255

### Validation of the Nine-Gene Signature

**FIGURE 4:**
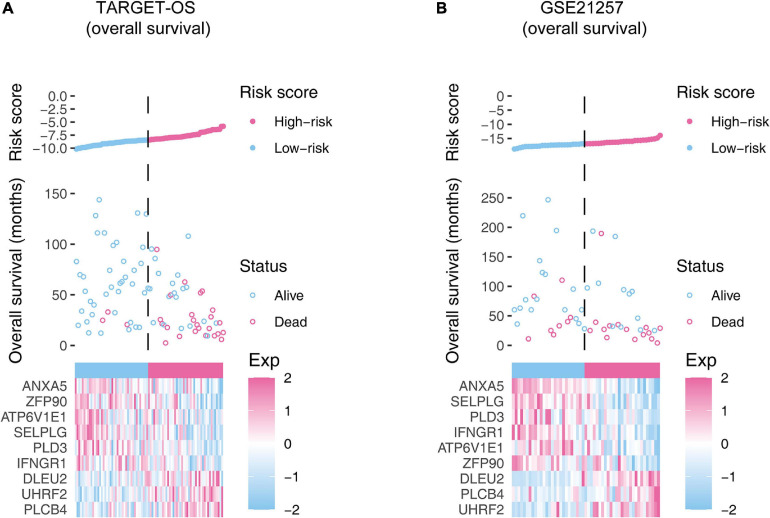
The overall distributions of the risk score (upper), survival status (middle), and gene expression profiles (bottom) of the nine-gene signature in the training **(A)** and validation **(B)** cohorts. The top parts show the distributions of the risk score. The center parts show the distributions of the patient survival time and status. The bottom parts indicate heatmaps of the nine gene expression profiles.

According to the median risk score, OSs were assigned to the high-risk group or low-risk group. In Figure 4, we showed the overall view of the signature in the OS cohorts, including the risk score distribution, the survival status/time distribution maps, and the expression heat maps. As exhibited in Supplementary Figure 2, in the training cohort and the validation cohort, ANXA5, ZFP90, ATP6V1E1, SELPLG, PLD3, and IFNGR1 were associated with favorable prognoses for OS patients, while DLEU2, UHRF2, and PLCB4 were associated with unfavorable prognoses.

**FIGURE 5:**
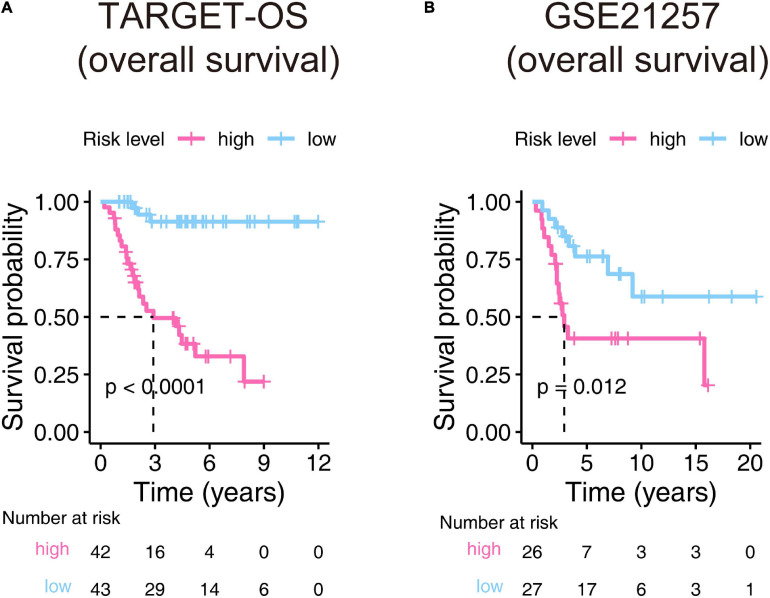
Kaplan–Meier estimator evaluates the nine-gene signature’s prognostic capacity in the training **(A)** and validation **(B)** cohorts. The bottom parts indicate the number of patients at risk. The two-sided log-rank test measured the differences between the high- and low-risk groups with a *p*-value < 0.05.

**FIGURE 6:**
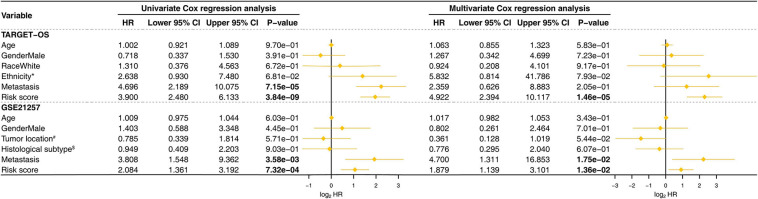
Univariate and multivariate Cox proportional hazards models were built to test the predictive ability of the nine-gene signature in the training and validation cohorts. HR, hazard ratio; CI, confidence interval; The bold p-value indicates that<0.05, which considers significant. ^*^Hispanic or Latino vs. Non-Hispanic or Latino; ^#^Femur vs. Non-Femur; ^$^Osteoblastic vs. Non-Osteoblastic.

The Kaplan–Meier estimators built in the TARGET-OS cohort and the GSE21257 cohort witnessed significant survival differences between the high- and low-risk groups. These analyses showed that the high-risk groups had poorer survival possibilities than those in the low-risk groups (TARGET-OS: Figure 5A, *p*-value < 0.0001; GSE21257: Figure 5B, *p*-value = 0.012). We constructed univariate and multivariate Cox proportional hazard models using the available clinical covariables of the training and validation cohorts to validate the nine-gene signature’s prognostic and independence ability (Figure 6). The covariables we included were as follows: risk score, sex, age, race, ethnicity, metastasis status, tumor location, and histological subtype. The Cox models built in the training cohort showed that the risk score is the only factor that significantly affects the prognosis of OS in both univariate (*p*-value = 3.84e–09) and multivariate (*p*-value = 1.46e–05) analyses. Consistent with the training cohort, the Cox proportional hazards models construed in the validation cohort also confirmed the strong predictive ability of the gene signature (*p*-value = 1.36e–02). In this analysis, the metastasis factor also validated having prognosis ability but was weaker than the risk score after comparing p-values. Based on the evidence mentioned above, we can reasonably infer that the nine-gene signature has a strong independent prognostic ability.

**FIGURE 7:**
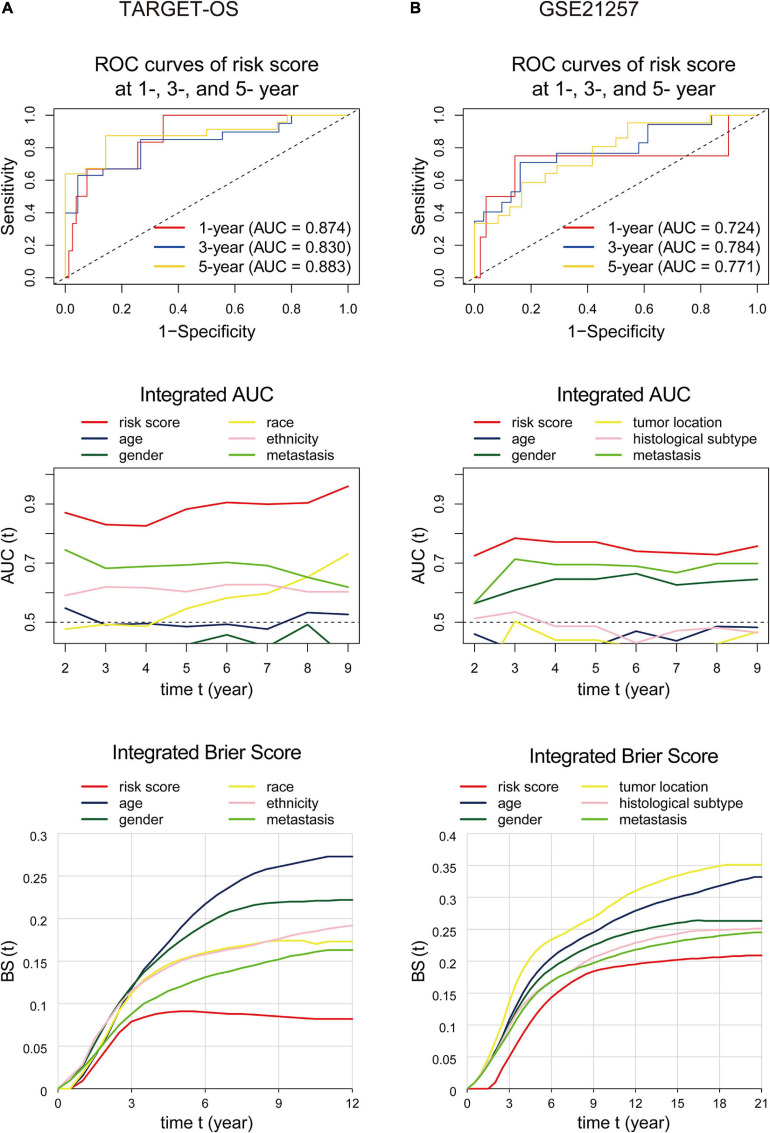
ROC curves, integrated AUC analysis, and integrated Brier score analysis performed to examine the predictive ability of the nine-gene signature in the training **(A)** and validation **(B)** cohorts. The ROC curvesdisplay prognostic capacities with the nine-gene signature at 1-, 3-, and 5-year time points. The integrated AUC and integrated Brier score analysis show the predictive ability comparisons between the nine-gene signature and other clinical factors. ROC, receiver operating characteristic; AUC, area under the ROC curve.

The area under the ROC curve, called AUC, is currently considered the standard method for evaluating the accuracy of predictive distribution models (Kamarudin et al., 2017). IAUC is an efficient tool in assessing the performance of a candidate marker given the true disease status of individuals at specific time points (Kamarudin et al., 2017). IBS is an overall measure for the prediction of the model at all times (Kronek and Reddy, 2008). This section used ROC curves, IAUC, and IBS to validate the nine-gene signature’s predictive ability and compare it with other variables. As displayed in Figure 7A, for the analyses performed in the training cohort, the gene signature’s AUCs were 0.874, 0.830, and 0.883 at 1, 3, and 5 years, respectively, and the IAUC results showed that the risk score stayed at a higher level compared to all other factors at all time points. Also, the IBS remained at the lowest level the whole time. Consistently, the risk score’s AUC in GSE21257 (Figure 7B) was=0.724 at the 1-, 3-, and 5-year time points and were greater than those of the other variates at any time. The signature’s IBS also stayed lower than other clinical characteristics at all time points. The above results indicated that the gene signature we found was superior to other clinical factors.

### Comparison of the Nine-Gene Signature With Previous Models

**FIGURE 8:**
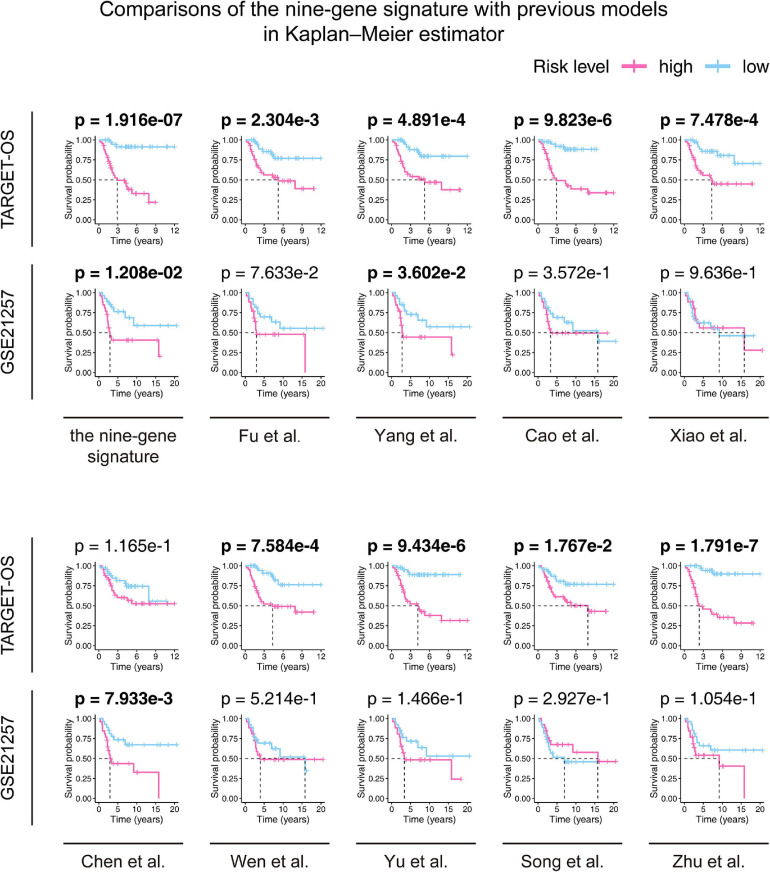
Comparisons between the nine-gene signature and previous studies conducted in the training and validation cohorts using the Kaplan–Meier estimator. The two-sided log-rank test measured the differences between the high- and low-risk groups. The bold p-value indicates<0.05, which is considered significant.

**FIGURE 9:**
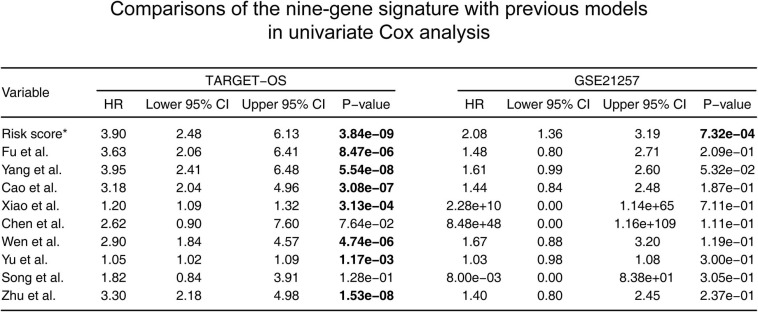
Comparisons between the nine-gene signature and previous studies conducted in the training and validation cohorts using the univariate Cox proportionalhazards model. HR, hazard ratio; CI, confidence interval. *: the nine-gene signature identified in this study; the bold p-value indicates that<0.05, which is considered significant.

According to the screening criteria, nine studies were selected as candidates. The details are shown inTable 3. Kaplan–Meier curves were plotted against our nine-gene signature and the candidate signatures in the training and validation cohorts (Figure 8). Kaplan–Meier plots indicated that only the nine-gene signature and that of Yang et al. signature could predict prognosis in both the TARGET-OS and GSE21257 cohorts. However, the p-value of our nine-gene signature (*p*-value = 1.208e–02) witnessed more strength than that of Yang et al. (*p*-value = 3.602e–02). Additionally, univariate Cox models were built using these gene signatures in the training and validation cohorts (Figure 9). The univariate Cox regression results showed that only our nine-gene signature exhibited prognosis capable in these cohorts (*p*-value = 7.32e–04). From the evidence mentioned above, the nine-gene signature is superior to our predecessors.

**TABLE 3:** Candidate research for comparison to our signature.

**Studies**	**Published online date**	**PMID**	**Gene signature composition**
Fu et al.	2021 Mar 18	33816483	DCN, P4HA1
Yang et al.	2021 May 5	33952718	P4HA1, ABCB6, STC2
Cao et al.	2020 Dec 23	33425993	GJA5, APBB1IP, NPC2, FKBP11
Xiao et al.	2020 Dec 15	33384961	IFITM3, VAMP8, ACTA2, GZMA, CDCA7, EVI2B, SLC7A7
Chen et al.	2020 Dec 14	33381518	MSR1, TLR7
Wen et al.	2020 Dec 3	33281116	COCH, MYOM2, PDE1B
Yu et al.	2020 Aug 21	32820615	CXCR3, SSTR3, SAA1, CCL4, PYY, CCR9, CXCL9, CXCL11, C3, CXCL2, S1PR4, CXCL10, CXCR6
Song et al.	2020 Jul 24	32850346	CD4, CD68, CSF1R
Zhu et al.	2020 Jun 22	32581649	SLC18B1, RBMXL1, DOK3, HS3ST2, ATP6V0D1, CCAR1, C1QTNF1

### Functional Annotation of the Gene Signature Using GSEA

For understanding the underlying molecular mechanisms of the signature, we performed GSEA comparing the high-risk group with the low-risk group in the TARGET-OS cohort. As displayed in Supplementary Figure 3, enriched gene sets were all detected in the low-risk group and were primarily involved in mechanisms related to transplant rejection, blood coagulation system, IL-6/JAK/STAT3 signaling axis, drug metabolism, inflammatory response, interferon response, apical junction complex, apical surface of epithelial cells, and innate immune system.

### The Nine-Gene Signature and 22 TICs

**FIGURE 10:**
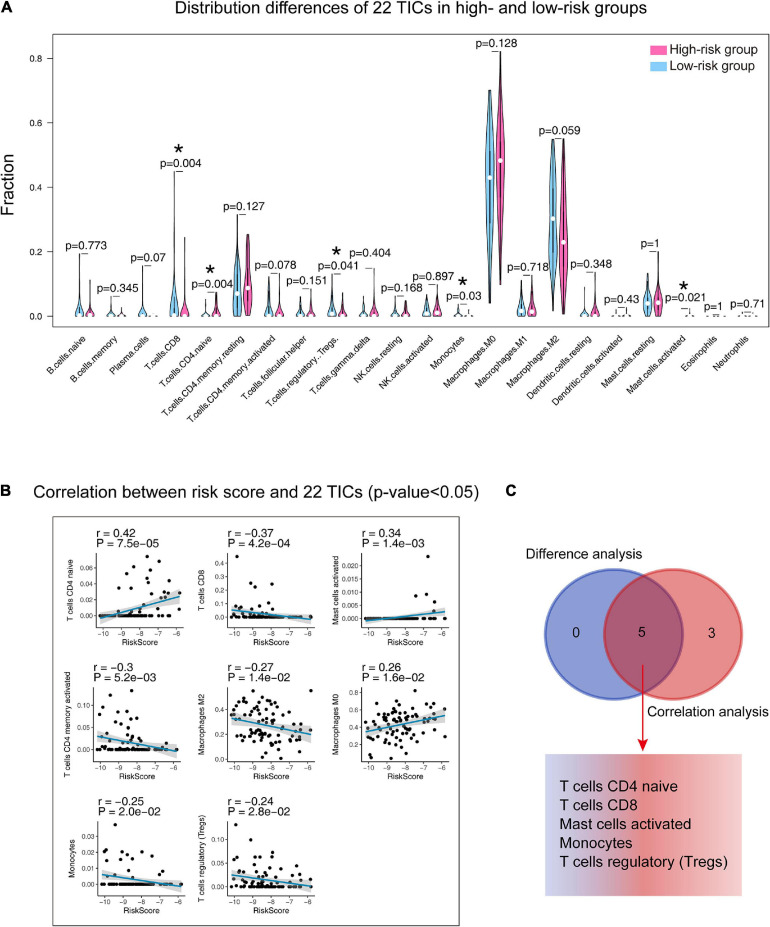
Integrating analysis for identifying the relationship between 22 TICs and the nine-gene signature. **(A)** Wilcoxon rank-sum was adopted to test the differences in each TIC distributed in the high- and low-risk groups. **(B)** The Spearman coefficient examines the correlation between each TIC and the nine-gene signature. Only correlations with *p*-value < 0.05 were plotted. The blue line displays the trend of the TIC and risk score. The shading besides the blue line characterizes the 95% CI. **(C)** Venn diagram integrating the results from **(A)** and **(B)**. TIC: tumor-infiltrating immune cell; *: *p*-value < 0.05; CI: confidence interval; *p*-value < 0.05 was considered statistically significant.

The annotation analysis indicated that the differences between the two groups were related to immunity, so we carried out analyses on 22 TICs to further study the interactions between the gene signature and the immune microenvironment. Firstly, the CIBERSORT algorithm was adopted to draw the 22 TIC profiles in preparation for the next analyses. Supplementary Figure 4 shows the overall view of the distribution of the 22 TICs and their inner association. The Wilcoxon rank-sum test (Figure 10A) found five kinds of TICs associated with the signature, and the Spearman coefficient (Figure 10B and Supplementary Table 3) discovered eight kinds of TICs having correlations with the risk score. We built a Venn diagram to visualize these results and found that there were five TICs (Figure 10C) showing significant relationships with the gene signature risk score, which included CD4 naïve T cells, CD8 T cells, activated mast cells, monocytes, and regulatory T cells (Tregs). Specifically, the risk score was positively correlated with CD4 T cells and activated mast cells and negatively correlated with the remaining.

**FIGURE 11:**
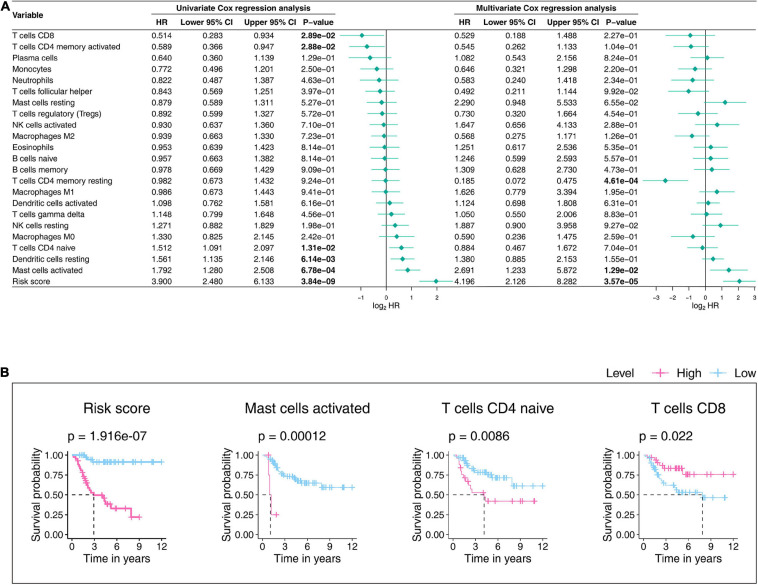
Univariateand multivariate Cox proportionalhazards models **(A)** and Kaplan–Meier estimators **(B)** evaluate the 22 TICs’prognostic ability. **(A)** The bold p-value indicates that<0.05. **(B)** In the established Kaplan–Meier estimators, we only selected those with a p-value less than 0.05 for display. A *p*-value < 0.05 was considered statistically significant; TIC, tumor-infiltrating immune cell.

We further tested the prognostic abilities of the 22 TICs by consulting the Kaplan-Meier estimator and Cox proportional hazards model. As we displayed the results in Figure 11A, the Cox models pointed that activated mast cells impacted OS outcomes not only in the univariate but also in the multivariate Cox regression. Additionally, the Kaplan–Meier estimator (Figure 11B and Supplementary Table 4) indicated that activated mast cells, CD4 T cells, and CD8 naïve T cellswere able to predict OS prognosis. Activated mast cells have potential prognostic ability in OS.

Combining all the analysis results shown in this part, we noticed that activated mast cells not only are significantly related to the gene signature but also predict OS prognosis. Accordingly, the significant infiltration of activated mast cells mayplaykey roles in the prognostic ability of the nine-gene signature in OS patients.

## Discussion

In the present research, we found a novel nine-gene signature for OS prognosis from the comprehensive characterization of ICI profiles by mining the TARGET and GEO databases. After ICI clusters of the OS patients with survival differences in the training cohort were subtyped, DEGs were found. Kaplan–Meier analysis, univariate Cox analysis, and LASSO Cox regression analysis were applied to the DEGs, and a nine-gene signature was produced that was associatedwith the prognosis of OS patients.

We specially applied the nine-gene signature to the training and validation cohorts to the constructed Kaplan–Meier estimator, Cox proportional-hazards model, ROC curve, IAUC, and IBS and found significant statistical differences in these analyses, proving that this gene signature is effective and applicable for the OS prognosis prediction. In addition, comparisons with other discovered signatures were performed, which exhibited the superiority of our nine-gene signature. The immune infiltration results showed vital roles that activated mast cells may play that allows the nine-gene signature toinfluence the prognosis of OS. Compared with previous research, we are the first to adopt ICI profiles and use the LASSO method for model training and validate the model in an independent cohort. This work aimed to present future OS research with more hints.

The nine-gene signature that we found exhibited powerful prediction abilities not only in the TARGET-OS cohort but also in the independent validation cohort after being tested by a wide range of statistical approaches. The signature consisted of nine genes (Table 2), which were ZFP90, UHRF2, SELPLG, PLD3, PLCB4, IFNGR1, DLEU2, ATP6V1E1, and ANXA5. In our research, PLCB4, DLEU2, and UHRF2 unfavorably impacted OS prognosis, while the remaining factors exhibited protective effects (Supplementary Figure 2). PLCB4 encodes the β4 isoform of PLC isoenzymes, a superfamily that adjusts the metabolism of inositol lipids (Wu et al., 2019). Boosted expression of PLCB4 is correlated with a decrease in survival rates in patients with solid tumors, including mesothelioma, melanoma, and gastrointestinal tumors (Wu et al., 2019). However, there is still no research to reveal the mechanism of PLCB4 in the occurrence and development of OS. LncRNA DLEU2 is a cancer-related lncRNA thatregulates tumor progression in a variety of cancers (Liu W. et al., 2020). Compared with that in normal tissues, DLEU2 displaysan upregulated expression in pancreatic cancer tissues (Liu W. et al., 2020). Liu et al. reported that DLEU2 is highly expressed in OS and revealed that DLEU2 overexpression helps in the migration and proliferation of OS cells (Liu W. et al., 2020). They found that DLEU2 promotes the expansion of OS cells by sponging miR-337-3p and couldcontrol the expression of JAK2, thus participating in the progression of OS (Liu W. et al., 2020). UHRF1 is a well-known epigenetic regulator. A significant overexpression of UHRF1has been detected in several kinds of cancers (Liu W. et al., 2016). Liu et al. reported that UHRF1 promotes the proliferation of human OS cells and increases the invasiveness of human OS cells by downregulating the expression of E-cadherin and increasing EMT in an Rb1-dependent manner (Liu W. et al., 2016).

Recently, with the widespread application of bioinformatics, we can mine for possible gene signatures associated with OS prognosis from the publicly available TARGET and GEO databases. In addition, more and more studies are involved. For example, in the nine studies (Cao et al., 2020; Chen Z. et al., 2020; Song et al., 2020; Wen et al., 2020; Xiao et al., 2020; Yu et al., 2020; Zhu et al., 2020; Fu et al., 2021; Yang et al., 2021) we included in the study based on the inclusion criteria, they all looked for potential survival-related OS gene signatures from public datasets. However, after we validated them in the training and validation cohorts, we found that the nine-gene signature we generated was superior to others in predicting OS prognosis. Among the nine studies, the one by Yang et al. (2021)revealed a gene signature that closely resembled ours. Yang et al. first filtered genes through GSEAand then established a three-gene signature prognostic model, which they claimed can accurately predict the prognosis of OS. However, in our validation, although the three-gene signature’s ability was similar to ours in Kaplan–Meier analysis, it could not show stable prognostic capacity *via* Cox regression analysis (Yang et al., 2021).

Gene Set Enrichment Analysis (GSEA) of the HALLMARK collection found that the IL-6/JAK/STAT3 signaling axis plays a key role in gene signature functioning. The IL-6/JAK/STAT3 pathway is important to the growth and advancement of many human cancers (Johnson et al., 2018). IL-6 produced in the TME activates the JAK/STAT3 signaling pathway, favoring tumor growth and metastasis (Wang et al., 2019). IL-6 has been identified as a primary mediator of lung tropism in OS and suggests pleiotropic, redundant mechanisms that might affect metastasis (Gross et al., 2018). The JAK/STAT3 signaling pathway has been demonstrated to be a target to inhibit the growth and metastasis of osteosarcoma (Wang et al., 2019). Upstream kinase signals trigger the STAT3 signal cascade (Lee et al., 2019). It undergoes phosphorylation, homodimerization, translocation into the nucleus, and binding to DNA, leading to the expression of target genes involved in tumor cell proliferation, angiogenesis, metastasis, and immune editing (Lee et al., 2019). There is evidence that dysregulated STAT3 plays a carcinogenic role in OS by promoting processes including cell transformation, tumor growth, invasion, metastasis, chemotherapy resistance, and immune evasion (Liu Y. et al., 2021). STAT3 inhibitors may directly or indirectly downregulate the expression of target genes involved in OS (Liu Y. et al., 2021). Liu and colleagues reported that STAT3 is a potential target for the treatment of OS and may be effective for the treatment of OS (Liu Y. et al., 2021). Further study is needed to develop more about the connections between IL-6/JAK/STAT3 signaling and the signature we found and the therapeutic approach targeting the IL-6/JAK/STAT3 pathway.

In addition, based on the CIBERSORT algorithm and survival analysis, we revealed that activated mast cells have clear correlations with the gene signature and strong prognostic abilities as well, indicating that the infiltration of these cells may play a key role in the predictive ability of the gene signature. Mast cells can elevate tumor expansion by inducing angiogenesis and promote tissue remodeling by inducing changes in the composition of the extracellular matrix, and they can also promote pro-inflammatory pathways that can lead to impaired tumor progression (Maciel et al., 2015). Mast cells have also been shown to influence the extent of the dendritic cells, tumor-associated macrophages, and lymphocyte infiltrate through the release of mediators, enhancing the migration and proliferation of these cells (Inagaki et al., 2016). Activated mast cells can enhance the tissue homeostasis of TME disorders and facilitate the growth and spread of tumors (Oldford and Marshall, 2015). Studies have confirmed that some mechanisms triggered by mast cells can affect the OS homeostasis, impacting the occurrence and development of OS (Campillo-Navarro et al., 2014; Maciel et al., 2015; Inagaki et al., 2016). Our research shows that activated mast cells can potentially target the gene signature in OS therapy, which suggests that we should make more effort to these immune cells in future study.

This research has certain limitations. Our nine-gene signature was produced from retrospective data. Its clinical adaptability should be further confirmed by more prospective data. In addition, although we have confirmed that the nine-gene signature is superior to previous findings, there is still no wet-lab experiment data backing up these 9 genes’ prognostic abilities and their roles in the immune infiltration. Accordingly, further study is needed to uncover the relationships between the nine-gene signature and OS progression.

## Conclusion

The present study defined a novel, robust nine-gene signature from OS ICI characteristics. The signature was closely associated with OS prognosis and can acutely determine the risk score of OS patients. In addition, we determined the signature’s stability and wide applicability by applying it to one independent validation cohort and proved that our signature is superior to previous signatures. We identified the vital role of activated mast cells in the signature’s prognostic ability. Our efforts may advance the unearthing of OS treatment approaches.

## Data Availability Statement

Publicly available datasets were analyzed in this study. This data can be found here: The following publicly available datasets were used in this study: TARGET-OS: https://ocg.cancer.gov/programs/target/projects/osteosarcoma; GSE21257: https://www.ncbi.nlm.nih.gov/geo/query/acc.cgi?acc=GSE21257.

## Author Contributions

LF and CM organized and wrote the manuscript. JR produced the figures and visualized the data. TL revised the manuscript. All authors reviewed the manuscript and approved the manuscript for publication.

## Conflict of Interest

The authors declare that the research was conducted in the absence of any commercial or financial relationships that could be construed as a potential conflict of interest.

## Publisher’s Note

All claims expressed in this article are solely those of the authors and do not necessarily represent those of their affiliated organizations, or those of the publisher, the editors and the reviewers. Any product that may be evaluated in this article, or claim that may be made by its manufacturer, is not guaranteed or endorsed by the publisher.

## Footnotes

1
https://gdc.xenahubs.net

2
https://pubmed.ncbi.nlm.nih.gov/

3
https://www.gsea-msigdb.org/gsea/downloads.jsp

4
http://www.gsea-msigdb.org/gsea/downloads.jsp

## Supplementary Material

Supplementary Figure 1 Consensus clustering for tumor-infiltrating immune cells. Consensus matrixes of the samples in the training cohort for each k (*k* = 2–5), displaying the clustering stability using 1000 iterations of hierarchical clustering.

Supplementary Figure 2 The prognostic prediction ability of each gene in the nine-gene signature tested using the Kaplan–Meier estimator in the training **(A)** and validation **(B)** cohorts. The two-sided log-rank test measured the differences between the high and low expression level groups. *P*-value < 0.05 was considered as significantly.

Supplementary Figure 3 GSEA conducted using HALLMARK collection. GSEA: Gene Set Enrichment Analysis; NES: Normalized Enrichment Score; NOM: Nominal; FDR: False Discovery Rate; A gene set with | NES | > 1, NOM *p*-value < 0.05, and FDR *q*-value < 0.25 was considered significantly enriched.

Supplementary Figure 4 Profiles and correlation analysis of the 22 TICs in OS tumor samples of the training cohort. **(A)** The distribution of 22 TICs in high- and low-risk OS patients in the training cohort, which are displayed in the form of bar plots. **(B)** The inner correlations between 22 TICs, which are shown in the form of a heatmap and performed via the Pearson coefficient. *P*-value < 0.05 was considered as significantly. TIC: tumor-infiltrating immune cell; OS: osteosarcoma.

Supplementary Table 1 Differentially expressed genes identified between two ICI clusters (*p*-value < 0.05 and log2| fold-change| > 0.2).

Supplementary Table 2 Potential prognostic genes identified in the training cohort (*p*-value < 0.05).

Supplementary Table 3 Correlations that determined between the nine-gene signature and the 22 TICs by the Spearman coefficient.

Supplementary Table 4 The prognostic ability of 22 TICs tested by the Kaplan-Meier estimator.
