# Supplementary material for: Identification of a Novel Prognostic Gene Signature From the Immune Cell Infiltration Landscape of Osteosarcoma
Source: Front Cell Dev Biol. 2021 Sep 6;9:718624. doi: 10.3389/fcell.2021.718624 (PMC8450587; doi:10.3389/fcell.2021.718624)
Supplement: Supplementary Figure 1 — Consensus clustering for tumor-infiltrating immune cells. Consensus matrixes of the samples in the training cohort for each k (k = 2–5), displaying the clustering stability using 1000 iterations of hierarchical clustering. [file Data_Sheet_1.pdf]

Figure S1

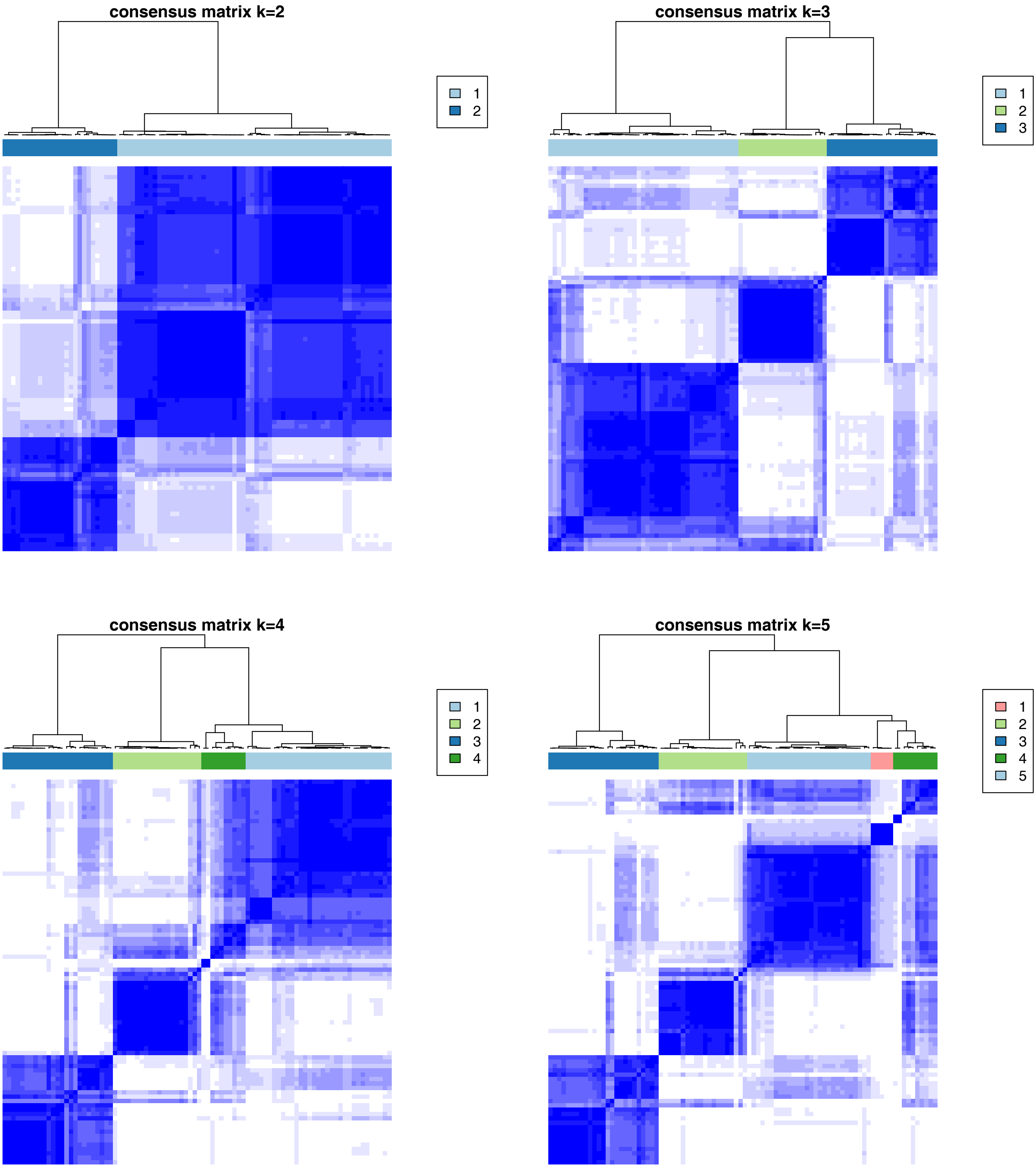
consensus matrix k=2
1
2
consensus matrix k=3
1
2
3
consensus matrix k=4
1
2
3
4
consensus matrix k=5
1
2
3
4
5

Figure S2

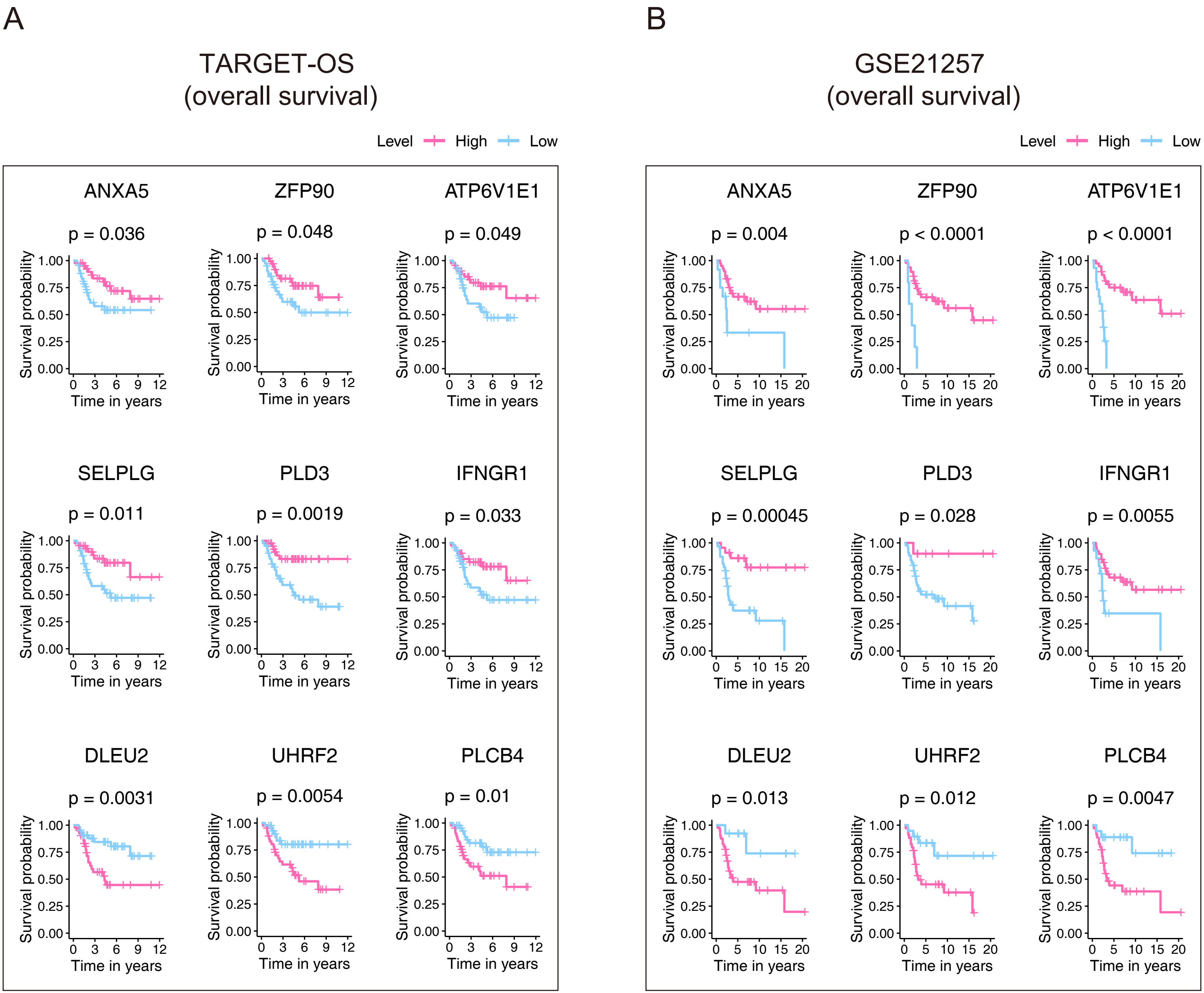

A
TARGET-OS
(overall survival)
Level High Low
ANXA5
p = 0.036
ZFP90
p = 0.048
ATP6V1E1
p = 0.049
SELPLG
p = 0.011
PLD3
p = 0.0019
IFNGR1
p = 0.033
DLEU2
p = 0.0031
UHRF2
p = 0.0054
PLCB4
p = 0.01
Survival probability
Time in years
B
GSE21257
(overall survival)
Level High Low
ANXA5
p = 0.004
ZFP90
p < 0.0001
ATP6V1E1
p < 0.0001
SELPLG
p = 0.00045
PLD3
p = 0.028
IFNGR1
p = 0.0055
DLEU2
p = 0.013
UHRF2
p = 0.012
PLCB4
p = 0.0047
Survival probability
Time in years

Figure S3

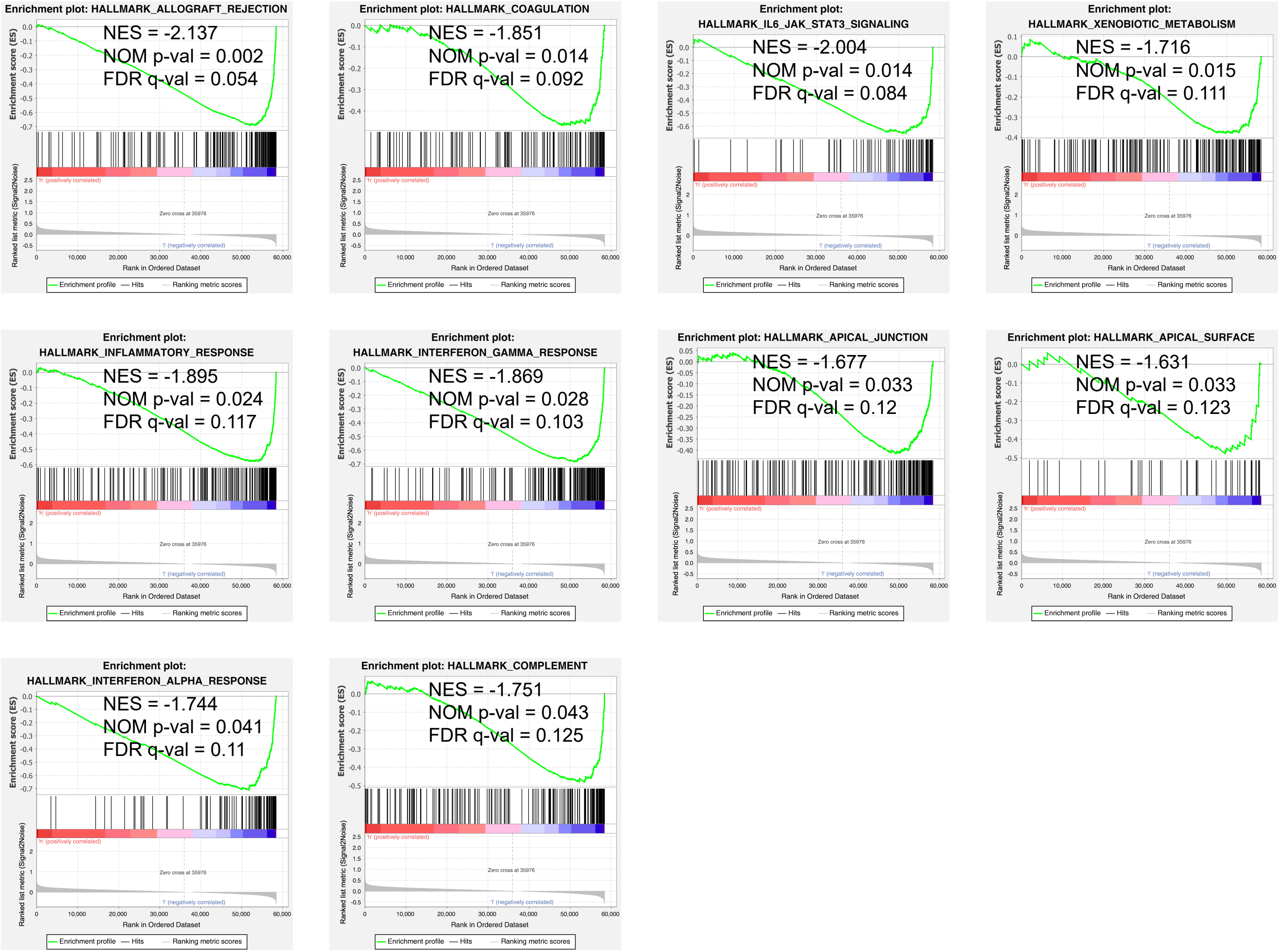
Enrichment plot: HALLMARK_ALLOGRAFT_REJECTION
NES = -2.137
NOM p-val = 0.002
FDR q-val = 0.054
Enrichment plot: HALLMARK_COAGULATION
NES = -1.851
NOM p-val = 0.014
FDR q-val = 0.092
Enrichment plot: HALLMARK_IL6_JAK_STAT3_SIGNALING
NES = -2.004
NOM p-val = 0.014
FDR q-val = 0.084
Enrichment plot: HALLMARK_XENOBIOTIC_METABOLISM
NES = -1.716
NOM p-val = 0.015
FDR q-val = 0.111
Enrichment plot: HALLMARK_INFLAMMATORY_RESPONSE
NES = -1.895
NOM p-val = 0.024
FDR q-val = 0.117
Enrichment plot: HALLMARK_INTERFERON_GAMMA_RESPONSE
NES = -1.869
NOM p-val = 0.028
FDR q-val = 0.103
Enrichment plot: HALLMARK_APICAL_JUNCTION
NES = -1.677
NOM p-val = 0.033
FDR q-val = 0.12
Enrichment plot: HALLMARK_APICAL_SURFACE
NES = -1.631
NOM p-val = 0.033
FDR q-val = 0.123
Enrichment plot: HALLMARK_INTERFERON_ALPHA_RESPONSE
NES = -1.744
NOM p-val = 0.041
FDR q-val = 0.11
Enrichment plot: HALLMARK_COMPLEMENT
NES = -1.751
NOM p-val = 0.043
FDR q-val = 0.125
Enrichment score (ES)
Ranked list metric (Signal2Noise)
'h' (positively correlated)
Zero cross at 35976
'l' (negatively correlated)
Rank in Ordered Dataset
Enrichment profile
Hits
Ranking metric scores

Figure S4

A

B

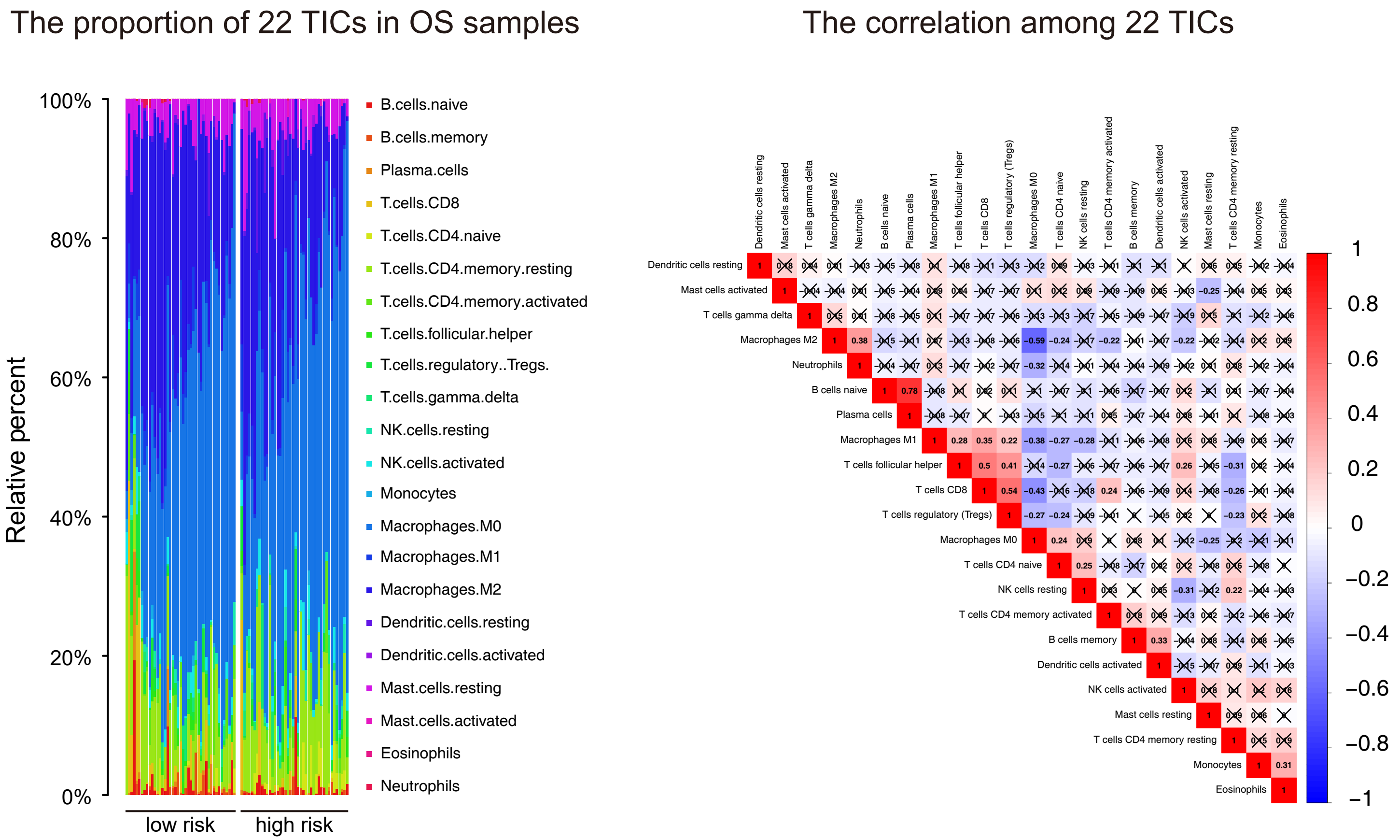

The proportion of 22 TICs in OS samples
Relative percent
100%
80%
60%
40%
20%
0%
low risk
high risk
B.cells.naive
B.cells.memory
Plasma.cells
T.cells.CD8
T.cells.CD4.naive
T.cells.CD4.memory.resting
T.cells.CD4.memory.activated
T.cells.follicular.helper
T.cells.regulatory..Tregs.
T.cells.gamma.delta
NK.cells.resting
NK.cells.activated
Monocytes
Macrophages.M0
Macrophages.M1
Macrophages.M2
Dendritic.cells.resting
Dendritic.cells.activated
Mast.cells.resting
Mast.cells.activated
Eosinophils
Neutrophils
The correlation among 22 TICs
Dendritic cells resting
Mast cells activated
T cells gamma delta
Macrophages M2
Neutrophils
B cells naive
Plasma cells
Macrophages M1
T cells follicular helper
T cells CD8
T cells regulatory (Tregs)
Macrophages M0
T cells CD4 naive
NK cells resting
T cells CD4 memory activated
B cells memory
Dendritic cells activated
NK cells activated
Mast cells resting
T cells CD4 memory resting
Monocytes
Eosinophils
1
0.8
0.6
0.4
0.2
0
−0.2
−0.4
−0.6
−0.8
−1

Table S1. Differentially expressed genes identified between two ICI clusters (p-value < 0.05 and log2|fold-change| > 0.2).

<table>
<tr><th>Gene</th><th>log2|fold-change|</th><th>average expression</th><th>t</th><th>p-value</th><th>B</th></tr>
<tr><td>DEF6</td><td>-0.832532215</td><td>1.570073862</td><td>-5.552095367</td><td>3.00E-07</td><td>6.17410153</td></tr>
<tr><td>TREM2</td><td>-1.276480581</td><td>2.984409267</td><td>-5.360262238</td><td>6.70E-07</td><td>5.476771489</td></tr>
<tr><td>PARVG</td><td>-0.631259042</td><td>1.104859034</td><td>-5.296207605</td><td>8.74E-07</td><td>5.246346276</td></tr>
<tr><td>C1orf162</td><td>-1.019613761</td><td>1.935017129</td><td>-5.239133296</td><td>1.11E-06</td><td>5.042101765</td></tr>
<tr><td>C1QA</td><td>-1.679834253</td><td>5.711790772</td><td>-5.015058237</td><td>2.76E-06</td><td>4.250497029</td></tr>
<tr><td>VSIG4</td><td>-1.503696198</td><td>2.715814852</td><td>-5.012817417</td><td>2.78E-06</td><td>4.242666776</td></tr>
<tr><td>LY86</td><td>-0.945329637</td><td>1.930266703</td><td>-4.988643029</td><td>3.07E-06</td><td>4.158304744</td></tr>
<tr><td>AIF1</td><td>-1.227298786</td><td>3.514843394</td><td>-4.958995651</td><td>3.46E-06</td><td>4.055125959</td></tr>
<tr><td>RNASE1</td><td>-1.252348987</td><td>6.199248805</td><td>-4.890628279</td><td>4.55E-06</td><td>3.81840008</td></tr>
<tr><td>ALOX5AP</td><td>-1.111341593</td><td>2.367748302</td><td>-4.853018893</td><td>5.28E-06</td><td>3.688906094</td></tr>
<tr><td>NCF4</td><td>-0.984042011</td><td>2.189214787</td><td>-4.796914899</td><td>6.59E-06</td><td>3.496717484</td></tr>
<tr><td>SASH3</td><td>-0.877573895</td><td>1.759515069</td><td>-4.773282569</td><td>7.23E-06</td><td>3.416121459</td></tr>
<tr><td>HLA-DMA</td><td>-1.146425602</td><td>3.756490497</td><td>-4.757719542</td><td>7.69E-06</td><td>3.363162541</td></tr>
<tr><td>B3GALNT2</td><td>0.452206093</td><td>1.431484281</td><td>4.705714104</td><td>9.43E-06</td><td>3.186879437</td></tr>
<tr><td>MIR4479</td><td>0.745546805</td><td>0.743588183</td><td>4.691144589</td><td>9.98E-06</td><td>3.137684004</td></tr>
<tr><td>C1QB</td><td>-1.58772833</td><td>5.39687012</td><td>-4.660296584</td><td>1.13E-05</td><td>3.033801801</td></tr>
<tr><td>C1QC</td><td>-1.513892644</td><td>6.131019252</td><td>-4.646886926</td><td>1.19E-05</td><td>2.98876324</td></tr>
<tr><td>MS4A4A</td><td>-1.063558689</td><td>2.152367956</td><td>-4.606822839</td><td>1.38E-05</td><td>2.854635941</td></tr>
<tr><td>FAM26F</td><td>-0.860349079</td><td>2.16224982</td><td>-4.568533263</td><td>1.60E-05</td><td>2.727064878</td></tr>
<tr><td>RP11-700A24.1</td><td>0.411573846</td><td>0.390295926</td><td>4.500658159</td><td>2.08E-05</td><td>2.502427295</td></tr>
<tr><td>TNFAIP8L2</td><td>-0.840079025</td><td>2.002444798</td><td>-4.487733685</td><td>2.19E-05</td><td>2.45987404</td></tr>
<tr><td>MS4A6A</td><td>-1.025175557</td><td>2.262413582</td><td>-4.481011549</td><td>2.24E-05</td><td>2.43776998</td></tr>
<tr><td>RNU7-180P</td><td>0.636889257</td><td>0.256767707</td><td>4.469625655</td><td>2.34E-05</td><td>2.400374613</td></tr>
<tr><td>LST1</td><td>-0.832405928</td><td>1.653633634</td><td>-4.430304317</td><td>2.72E-05</td><td>2.271660515</td></tr>
<tr><td>RP11-733O18.1</td><td>-0.493688023</td><td>0.68415136</td><td>-4.406787853</td><td>2.97E-05</td><td>2.195004379</td></tr>
<tr><td>MNDA</td><td>-0.760672407</td><td>1.636466085</td><td>-4.389407547</td><td>3.17E-05</td><td>2.138506699</td></tr>
<tr><td>PYCARD</td><td>-0.83598238</td><td>2.316241593</td><td>-4.387163033</td><td>3.20E-05</td><td>2.13122027</td></tr>
<tr><td>HLA-DPB1</td><td>-1.165752218</td><td>5.050715901</td><td>-4.378572544</td><td>3.31E-05</td><td>2.103353387</td></tr>
<tr><td>PILRA</td><td>-0.678004738</td><td>1.8673977</td><td>-4.377837975</td><td>3.31E-05</td><td>2.100972027</td></tr>
<tr><td>MTCL1</td><td>0.429135554</td><td>1.274865439</td><td>4.37550363</td><td>3.34E-05</td><td>2.093406027</td></tr>
<tr><td>EFCAB14P1</td><td>0.215922802</td><td>0.271327918</td><td>4.372637774</td><td>3.38E-05</td><td>2.084120634</td></tr>
</table>

TPR	0.504847198	3.42565203	4.34908563	3.69E-05	2.007950607
HLA-DMB	-0.8708376	2.131250498	-4.346444771	3.73E-05	1.999425315
CTD-3099C6.9	0.603524582	1.938527356	4.329333558	3.97E-05	1.944262615
RP11-848P1.3	-1.109403728	2.589474766	-4.316626901	4.17E-05	1.903384926
CD14	-1.345057004	4.853030055	-4.311846542	4.24E-05	1.888025354
TMEM150A	-0.593918207	2.423715202	-4.302554609	4.39E-05	1.85819962
NCF1B	-0.479811396	0.617424069	-4.299628653	4.44E-05	1.848815893
KDM2A	0.486972828	3.733828047	4.299031515	4.45E-05	1.846901313
LRRC25	-0.824979956	1.979614693	-4.29070176	4.59E-05	1.820210982
CD74	-1.358961472	7.580497164	-4.278004588	4.81E-05	1.779587755
HLA-DRB1	-1.326196672	6.532075456	-4.275573286	4.86E-05	1.77181753
CRYZP1	0.240098548	0.251544625	4.273100467	4.90E-05	1.763917411
NCF1	-0.513482592	0.76713023	-4.27130499	4.93E-05	1.758183022
ITGAM	-0.737603869	1.572105565	-4.248888932	5.36E-05	1.686716231
SRIP2	0.224682929	0.100518602	4.233274727	5.68E-05	1.637073058
BDH2	-0.507552183	2.38430639	-4.221044473	5.94E-05	1.598268247
PCED1B-AS1	-0.557298868	0.866420055	-4.219824254	5.97E-05	1.594400516
ADAP2	-0.885196246	2.494764729	-4.216495993	6.04E-05	1.583854479
CXCL16	-0.734783097	3.540526841	-4.216304505	6.04E-05	1.58324788
MBNL1-AS1	0.430386907	0.74507464	4.210010446	6.18E-05	1.563319124
CPEB3	0.385339376	0.751164625	4.177798865	6.96E-05	1.461621173
RN7SL138P	-1.210977847	2.793929349	-4.160451892	7.42E-05	1.407057852
REEP5	-0.398346489	4.339927909	-4.131991414	8.23E-05	1.317850442
RP11-54D18.4	0.362183831	0.529562801	4.127290928	8.37E-05	1.303154652
APOA1-AS	0.259117976	0.332983427	4.114830102	8.76E-05	1.264248393
CD37	-0.701560716	1.804755224	-4.085396553	9.74E-05	1.172648505
DGKH	0.368712609	1.114228115	4.067082912	0.000104046	1.115868925
CXorf21	-0.534566578	0.99658538	-4.063076714	0.000105558	1.103470131
HMGA1P2	0.44650412	0.748063923	4.06079249	0.000106429	1.096404229
UCKL1-AS1	0.357918679	0.500121303	4.060418433	0.000106573	1.095247386
FCGR1C	-0.472098357	0.588319401	-4.040789399	0.000114363	1.034638038
RP11-211G3.2	0.355048182	0.501200974	4.040539027	0.000114466	1.033866191
VAMP8	-1.089331323	4.432809335	-4.037932896	0.000115541	1.025833844

CORO1A	-0.823866592	2.350943582	-4.030266274	0.000118761	1.002224188
C3AR1	-0.848800625	2.579125141	-4.029425502	0.000119119	0.999636781
LILRA1	-0.227794437	0.28972782	-4.022211384	0.000122237	0.977450435
KRT18P55	0.222111633	0.103250956	4.015595532	0.000125165	0.957126881
USP37	0.376841086	0.963542336	4.009682566	0.000127838	0.938981122
STAB1	-1.016176526	3.435109895	-4.005091663	0.000129951	0.924904627
RP11-458F8.2	0.50162643	1.291599521	4.003681885	0.000130607	0.920584132
RP11-430C1.2	0.456807376	0.357035676	3.998272289	0.000133152	0.904014822
LILRB2	-0.643239639	1.23339763	-3.995802252	0.00013433	0.89645413
CEBPA	-0.776886559	2.032466725	-3.994948812	0.000134739	0.893842497
PCED1B	-0.532054999	1.057293246	-3.993138844	0.000135612	0.888304986
ADRB2	-0.227326163	0.419103752	-3.979883402	0.000142165	0.847801035
FGL2	-0.867201485	1.993798835	-3.979041254	0.000142591	0.84523073
ARHGAP9	-0.582052539	1.40408291	-3.978112748	0.000143063	0.842397274
ZC3HAV1L	0.70209976	2.831427187	3.976806896	0.000143729	0.838413034
RTEL1	0.236558813	0.468386327	3.974629355	0.000144845	0.83177114
ADORA3	-0.554998738	1.198636342	-3.972106291	0.00014615	0.82407835
HOMER3	-0.605756982	3.713684961	-3.969827724	0.000147338	0.817133806
PROZ	0.217686009	0.189813357	3.959467617	0.000152855	0.785591907
FCER1G	-1.172044894	5.257895108	-3.958564029	0.000153346	0.782843479
MIR4665	0.478675965	0.393648236	3.955754944	0.00015488	0.774301789
SIGLEC1	-0.829866778	1.597309775	-3.944462562	0.000161198	0.740005302
EIF1AX-AS1	0.373606661	0.291969154	3.941876828	0.00016268	0.732161264
HCLS1	-0.73561228	2.921832341	-3.938378497	0.000164704	0.721554241
BEX4	-0.782247428	2.710635364	-3.934400681	0.000167035	0.709501043
RNA5SP21	0.34623029	0.211382282	3.932539419	0.000168137	0.703864016
NPC2	-0.862595336	5.504269655	-3.927447285	0.000171187	0.688451059
FCGR2B	-0.476583849	0.669645041	-3.927036764	0.000171435	0.687209068
NCF1C	-0.713725837	1.138746161	-3.924371875	0.000173054	0.679148818
CD163	-1.077046218	2.734002916	-3.902197378	0.000187107	0.612221855
ARHGDIB	-0.856497571	5.099836882	-3.887738587	0.000196851	0.568719705
FRY	0.337628037	0.652479371	3.886875226	0.000197448	0.566125551
CYBB	-1.080848509	2.971710912	-3.882770478	0.00020031	0.553797283

AC027612.4	0.203711307	0.141917606	3.872388031	0.000207726	0.522653735
MMS22L	0.319137391	0.918823284	3.871717647	0.000208214	0.520644766
RPL12P29	0.25251574	0.178270651	3.870670251	0.000208978	0.517506458
snoU109	0.687787424	2.107467714	3.864675282	0.000213404	0.499554827
MPEG1	-0.973163837	2.888496931	-3.8629874	0.000214667	0.494503949
IL10RA	-0.697453414	1.648851112	-3.859699907	0.000217146	0.484670636
FCGR2A	-0.914014327	2.932072394	-3.847217549	0.00022681	0.447386059
RP11-304L19.1	0.310890704	0.508182048	3.84698028	0.000226997	0.446678136
LPXN	-0.603000128	2.193808612	-3.842859155	0.00023028	0.434386955
TLR7	-0.494126606	0.790183927	-3.840863711	0.000231885	0.428438804
RNU7-18P	0.523079556	0.398692682	3.840349471	0.000232301	0.426906264
PIK3R5	-0.402838266	0.900145069	-3.836990596	0.000235032	0.416899568
CD86	-0.704855709	1.96318571	-3.835234944	0.000236472	0.411671539
SLCO2B1	-0.78847499	2.17632366	-3.814467746	0.000254156	0.349954414
RP11-108M9.3	0.406868234	0.31341613	3.807248783	0.000260593	0.328554412
IKZF1	-0.39703286	0.621490652	-3.80502266	0.000262609	0.321960867
ZNF502	-0.384906228	1.512622065	-3.801135843	0.000266165	0.310454867
SCAI	0.302998463	1.064013164	3.798346114	0.000268746	0.30220151
C14orf159	-0.349429236	2.234952514	-3.79803835	0.000269032	0.301291254
NOTCH2NL	0.255037163	0.484866366	3.797664412	0.00026938	0.300185344
GUSBP2	0.366576666	0.633493567	3.795093875	0.000271784	0.292585087
GIMAP2	-0.637544467	1.792557407	-3.795006451	0.000271866	0.292326665
AC108925.1	0.328307851	0.254791743	3.791114807	0.000275547	0.280827233
CENPL	0.500576286	2.331455711	3.788545106	0.000278002	0.273238468
AC006486.10	0.205225683	0.338834916	3.785341012	0.000281094	0.263781195
CD53	-0.992156747	3.609533177	-3.782422588	0.000283938	0.255171906
NUPL1	0.490190828	2.325788376	3.775524388	0.000290771	0.234840575
CNTLN	0.407626207	1.589808643	3.769189674	0.000297183	0.216192599
HLA-DRA	-1.201130692	7.271355646	-3.768958021	0.00029742	0.215511074
CD81	-0.471968618	5.634499355	-3.767192086	0.000299233	0.210316637
INTS2	0.345121827	1.832423953	3.758054704	0.000308782	0.18346625
RP11-849H4.2	0.258960346	0.539465142	3.754340665	0.000312746	0.17256538
OSBPL2	0.363804894	2.147270887	3.752139097	0.000315118	0.166107208

TTC33	0.497782332	2.222082351	3.751877607	0.000315401	0.165340315
PRR5	-0.642032653	1.967036422	-3.747929991	0.000319702	0.153767356
MIR1296	0.459790775	0.405798514	3.745297175	0.000322601	0.146053608
RP11-160H22.5	0.301555768	0.313912523	3.742055275	0.000326205	0.136560513
GPR34	-0.808561176	2.075624632	-3.741528268	0.000326795	0.135017843
RNU6-1190P	0.482617663	0.208809056	3.728062167	0.000342207	0.095650818
FCGBP	-1.085094166	1.864773953	-3.726845898	0.000343633	0.092100028
CD300A	-0.650591994	1.942064399	-3.719442719	0.000352433	0.070504523
ITGB2	-0.895849781	3.324544112	-3.717339327	0.000354972	0.064374276
RP11-625H11.2	0.37509919	0.236320303	3.715606781	0.000357077	0.059326662
POU5F1P4	0.249789724	0.29552534	3.710965103	0.000362773	0.045811671
TMEM176B	-1.03550787	3.273903673	-3.70329677	0.000372375	0.023509984
LY96	-0.716079036	3.230539685	-3.701043925	0.000375241	0.016964214
RP11-344B5.2	-0.583652245	1.142565773	-3.700832332	0.000375511	0.016349563
AK3P2	0.287473917	0.201297729	3.697845642	0.000379346	0.007676216
SNX20	-0.385543873	0.685845929	-3.697396479	0.000379926	0.006372272
GGTA1P	-0.58222226	1.172958865	-3.696019403	0.00038171	0.00237524
SIGLEC7	-0.479847075	0.955539956	-3.688893005	0.000391066	-0.01829279
LGALS9	-0.783156912	2.585348798	-3.681327347	0.000401238	-0.040204084
BRCA1	0.393913909	1.928017526	3.680868091	0.000401863	-0.041533139
CTB-187L3.1	0.483804413	0.593346357	3.676262023	0.000408188	-0.054856334
LAMTOR5	-0.550719451	4.901735023	-3.669347362	0.00041786	-0.074835138
HCST	-0.934203757	2.085625656	-3.66822936	0.000419444	-0.078062935
RNA5SP179	0.329773555	0.195271939	3.664634932	0.000424576	-0.088435742
TMSB4X	-0.765190428	9.34831324	-3.660215	0.000430969	-0.101180948
CTD-2035E11.3	0.463345712	0.465473347	3.656162872	0.000436909	-0.112856012
RP11-777F6.3	0.321658071	0.322028956	3.655638812	0.000437683	-0.114365274
RP4-654C18.1	0.488142116	0.581060457	3.655611556	0.000437724	-0.114443767
HLA-DPA1	-1.125493311	3.923387851	-3.652271846	0.000442687	-0.124058294
PLS3-AS1	0.282732714	0.306084812	3.652126499	0.000442904	-0.124476588
CYP2S1	-0.401866028	0.806034142	-3.649672951	0.000446586	-0.131535823
RP11-404E16.1	0.315570909	0.433954664	3.647657706	0.000449632	-0.137331483
SUGT1P3	0.236610602	0.385489232	3.645802781	0.000452453	-0.14266407

AC104073.1	0.299617391	0.107573909	3.642473216	0.000457559	-0.152231172
CX3CR1	-0.712631862	1.003586776	-3.633096578	0.000472234	-0.179140469
CTD-2008P7.5	0.306255637	0.179359349	3.63216405	0.000473718	-0.181813967
ADPRHL2	-0.446848197	4.319310985	-3.630897483	0.00047574	-0.185444352
HLA-DOA	-0.748038482	1.766469637	-3.630757525	0.000475964	-0.185845458
KATNAL1	0.344492585	1.593202144	3.62654879	0.000482746	-0.197902239
CTD-2216M2.1	0.268937728	0.284981758	3.623849304	0.000487143	-0.205630229
PTPN6	-0.667876752	2.00293147	-3.621652479	0.00049075	-0.211916198
FCGR1A	-0.69106626	1.304275478	-3.621032389	0.000491773	-0.213690024
RFX3	0.287262086	0.768821696	3.619793504	0.000493822	-0.217233326
PRDM10	0.280044052	1.292906987	3.619379756	0.000494509	-0.218416483
RP11-315A16.1	0.3268526	0.135229195	3.617839492	0.000497071	-0.222820185
ZMYND15	-0.324986817	0.653310783	-3.614460665	0.000502737	-0.232475765
CTD-2012K14.2	0.204085567	0.163770322	3.613679966	0.000504054	-0.234705832
CD180	-0.356329325	0.681517934	-3.612994729	0.000505214	-0.236662929
CMTM3	-0.480269237	4.715866747	-3.612736377	0.000505651	-0.237400735
TCN2	-0.679524376	2.31084513	-3.611608893	0.000507566	-0.240620187
LILRA2	-0.208097941	0.305648685	-3.607713291	0.000514234	-0.251738289
CTD-2568A17.8	0.300797971	0.27675256	3.601001616	0.000525918	-0.270873394
HNRNPA1P54	0.303868337	0.153038711	3.60069501	0.000526457	-0.271746925
LRP8	0.413811091	1.271680873	3.600676886	0.000526489	-0.271798558
CARD11	-0.351183794	0.723291318	-3.600223421	0.000527289	-0.273090394
SAMHD1	-0.648441737	4.295941818	-3.597250205	0.000532557	-0.281557635
RNU6-1282P	0.335108949	0.145619541	3.593418083	0.000539422	-0.292463519
WDR90	0.363530314	1.285992865	3.59061573	0.000544495	-0.300433497
RP11-661D19.1	0.345477458	0.290709949	3.590310274	0.00054505	-0.301301956
XXbac-B476C20.14	0.396289995	0.279787729	3.588304872	0.000548712	-0.3070023
LRRC8E	0.359925731	0.840644429	3.587645339	0.000549921	-0.30887652
UHRF1BP1L	0.407292079	2.227919569	3.587264021	0.000550621	-0.309960014
LPAR5	-0.587302485	1.477495417	-3.58615315	0.000552666	-0.313116019
RP5-1091N2.9	-0.264240432	0.455193751	-3.585903257	0.000553127	-0.313825873
EDEM3	0.508777844	3.008396909	3.584229814	0.000556224	-0.318578597
TLR2	-0.584523635	1.531182384	-3.583173898	0.000558186	-0.32157667

HVCN1	-0.427622925	1.702829481	-3.583063781	0.000558391	-0.32188929
LINC01476	0.224518274	0.153716918	3.582798636	0.000558885	-0.322642004
AL050335.1	0.755627695	0.267701086	3.579302056	0.000565438	-0.332564609
MIR4442	1.140707989	3.675274254	3.578397976	0.000567144	-0.335129078
RP11-365O16.5	0.311657046	0.186256172	3.576710813	0.000570341	-0.339913558
ARRB2	-0.572419572	3.181063116	-3.574689497	0.000574194	-0.3456435
HOXB-AS2	0.223487866	0.327129495	3.572339114	0.000578704	-0.35230334
SPATA21	0.254602488	0.153327689	3.571014448	0.000581261	-0.356055408
UBA52	-0.471003643	6.882761709	-3.570931205	0.000581422	-0.356291158
ODCP	0.223575141	0.310760185	3.569426489	0.000584341	-0.360551929
PGAM1P9	0.205518291	0.176212399	3.569111631	0.000584953	-0.361443319
SCN1B	-0.538498179	1.577684886	-3.567756342	0.000587596	-0.365279622
GIMAP1	-0.467468948	1.100191804	-3.564117085	0.000594749	-0.375575766
SRGN	-1.0306177	4.904301258	-3.563417748	0.000596133	-0.377553457
PRSS48	0.292521752	0.254923365	3.560984425	0.000600973	-0.384432592
VPS13A	0.331631774	0.949827741	3.55996346	0.000603014	-0.387317908
FOLR2	-1.097815941	4.181972005	-3.557506874	0.000607953	-0.39425794
PARVB	-0.465593478	2.647628976	-3.557313946	0.000608343	-0.394802828
CD52	-0.927353088	1.899383598	-3.556147666	0.000610702	-0.398096314
VAMP5	-0.822039919	5.382612173	-3.554250591	0.000614559	-0.403451848
FCGR1B	-0.249041927	0.330712982	-3.553001082	0.000617112	-0.406978145
TNK2-AS1	0.249294941	0.408385891	3.552663577	0.000617803	-0.407930479
RP11-64D24.2	0.253373285	0.148742417	3.550718014	0.000621803	-0.413418985
RP11-115H15.2	0.382465287	0.360881295	3.550202783	0.000622866	-0.414872108
IRF8	-0.510625792	1.161198187	-3.549441739	0.000624439	-0.417018225
RP5-1021I20.5	0.240690359	0.248167394	3.546120396	0.000631351	-0.426380408
RNU6-879P	0.449281696	0.404958418	3.545927015	0.000631755	-0.426925312
CAPNS1	-0.48364086	6.323072824	-3.544853548	0.000634006	-0.429949722
LSM10	-0.414375691	4.512961596	-3.543613713	0.000636615	-0.433442034
RP11-274J7.2	0.229967526	0.165899322	3.540038053	0.000644196	-0.443508854
RP5-1153D9.5	0.2163341	0.137067256	3.53985425	0.000644588	-0.444026128
MS4A7	-0.832288075	2.43197401	-3.533083444	0.000659186	-0.463067607
CCL2	-0.964779538	2.029317839	-3.532173007	0.000661172	-0.465626006

KCNJ5	-0.356628035	0.76949008	-3.531637228	0.000662344	-0.467131365
RP11-2G1.1	0.388054147	0.154874931	3.525857294	0.00067511	-0.483360501
RP11-110H1.8	0.309183018	0.154247756	3.525115465	0.000676765	-0.485442043
ZNF483	0.25034611	0.340161572	3.523211279	0.000681031	-0.490783657
FAM168A	0.416733532	3.611821844	3.522133663	0.000683457	-0.493805652
TTC14	0.407787104	1.780383704	3.5220661	0.000683609	-0.493995099
SMARCA2	0.519956324	3.613394332	3.521960244	0.000683848	-0.494291914
ME1	0.692916507	3.358302332	3.521095403	0.000685802	-0.496716647
BNIP3P42	0.268288306	0.257535117	3.51763872	0.000693663	-0.506403734
SIAH2-AS1	0.331860054	0.492591142	3.516973744	0.000695185	-0.508266485
COMMD1	-0.355776072	3.372270752	-3.516877205	0.000695407	-0.508536892
HPGDS	-0.550980961	1.153014139	-3.516681419	0.000695855	-0.509085276
VAV1	-0.539821327	1.251937734	-3.516268585	0.000696803	-0.51024152
ANKRD26	0.356761223	1.30867693	3.515452242	0.00069868	-0.512527603
FCGR2C	-0.442182963	0.735109889	-3.514587195	0.000700674	-0.514949653
AC092638.1	0.207808607	0.069004683	3.514209625	0.000701546	-0.516006678
MPP1	-0.466639558	2.720046501	-3.511197781	0.000708539	-0.524435523
CAPG	-0.821989903	4.652752099	-3.508797055	0.000714161	-0.531150347
P2RY13	-0.409751696	0.65748973	-3.505619572	0.000721666	-0.540032619
CASP1	-0.601459697	1.667363026	-3.504341988	0.000724705	-0.5436023
RP11-82O19.2	0.221644824	0.334406449	3.50345451	0.000726823	-0.546081432
TNFSF8	-0.356506903	0.697665378	-3.503231882	0.000727355	-0.546703263
AL355480.1	0.727892949	0.892228038	3.502496687	0.000729115	-0.548756558
C11orf30	0.327442686	1.605002332	3.496229589	0.000744283	-0.566246918
CTSS	-0.874990046	3.466590341	-3.496070181	0.000744673	-0.566691499
HOXA6	0.467957397	1.024796403	3.493941469	0.000749895	-0.572626961
RP4-646N3.1	0.230321639	0.30762211	3.491944321	0.000754826	-0.578193187
RP11-407N17.2	0.374447201	0.162626567	3.49146694	0.000756009	-0.579523345
IGFLR1	-0.278789988	0.716720581	-3.490995565	0.000757179	-0.580836638
LINC01034	0.20201327	0.074224929	3.486520604	0.000768372	-0.593297828
TAF9	-0.490500211	3.789297101	-3.486257882	0.000769034	-0.594029052
RBM26-AS1	0.310145227	0.562310044	3.484391733	0.000773751	-0.599221885
RP5-1031D4.3	-0.245105477	0.33107544	-3.480791089	0.00078293	-0.609235452

RP5-843L14.1	0.235719637	0.0914868	3.470954961	0.000808533	-0.636551575
Clostridiales-1	0.426107012	0.219595158	3.470247947	0.000810403	-0.638512859
FTL	-0.59079508	10.90788896	-3.46913937	0.000813344	-0.6415875
CRYBB1	-0.452749062	0.939275667	-3.469071741	0.000813524	-0.641775046
NOS2	0.410694138	0.690168314	3.46512427	0.000824081	-0.652717364
MMP20	0.660119352	0.472370802	3.46226479	0.000831808	-0.660638082
ZNF770	0.510377922	2.408078197	3.462238071	0.000831881	-0.66071207
RP11-545I10.2	0.585450642	0.313641529	3.46193241	0.000832711	-0.661558458
RP11-571O6.2	0.203933842	0.15802249	3.461049403	0.000835114	-0.664003232
NPAT	0.43244376	1.975873324	3.455682414	0.000849858	-0.678852915
RP11-110H1.9	0.211365698	0.123613746	3.453694721	0.00085538	-0.684348276
AC009229.5	0.262517954	0.205639634	3.453510678	0.000855893	-0.684856982
HLA-DQA1	-0.947701436	2.574956377	-3.45187465	0.000860467	-0.689378174
CTB-43E15.1	0.249796104	0.097672838	3.451484139	0.000861562	-0.690457125
IGHMBP2	0.326637507	2.042568489	3.448353934	0.000870387	-0.699102393
NFAM1	-0.502466	1.375394478	-3.446548445	0.000875516	-0.70408632
RP11-856B14.10	0.427878436	0.194867508	3.445718325	0.000877884	-0.706377165
MIR6865	0.25043876	0.169625983	3.444060985	0.000882629	-0.710949634
RP11-351C8.1	0.307786964	0.111359154	3.443826331	0.000883303	-0.711596896
RNU2-30P	0.431248037	0.242297256	3.441820831	0.000889082	-0.717127464
RP11-1212A22.1	0.307562271	0.594938289	3.439562761	0.000895631	-0.723351706
OR4K2	0.266853117	0.099878863	3.439089667	0.000897009	-0.724655381
SAMSN1	-0.454046092	1.081813996	-3.437189935	0.000902562	-0.729889022
CCDC88B	-0.315139603	0.914706444	-3.434207945	0.000911344	-0.738099907
AC000403.1	0.720046897	1.45927598	3.431989195	0.000917931	-0.744205808
RAVER1	0.522934101	3.1394545	3.431241024	0.000920162	-0.746264084
SETP20	0.288176998	0.461962501	3.429683655	0.000924822	-0.750547463
CKLF	-0.518418059	2.940196158	-3.428605735	0.000928061	-0.753511329
KIAA1958	0.404636739	0.850674811	3.427349582	0.000931849	-0.756964395
CEP85	0.424385398	2.555246522	3.423645143	0.000943104	-0.767142167
CBL	0.449490534	2.42464146	3.42342933	0.000943764	-0.767734853
GS1-309P15.4	0.2362053	0.238753097	3.422871381	0.000945472	-0.769267013
PFDN1	-0.352176378	4.216610786	-3.421242167	0.000950474	-0.773739879

TMC1	0.232466516	0.195231021	3.421023128	0.000951148	-0.77434111
KLK1	0.525123653	0.636301254	3.420515023	0.000952715	-0.775735679
CD24P2	0.280604625	0.273706993	3.418860552	0.000957831	-0.780275557
AP001055.6	-0.202284709	0.29486161	-3.414514298	0.000971397	-0.792193959
LLNLR-470E3.1	-0.347484472	0.478086775	-3.413482914	0.000974642	-0.795020599
MIR548AA1	0.558337576	0.510486199	3.412567344	0.000977532	-0.797529302
RP11-560I19.2	0.363176818	0.21259997	3.410245055	0.000984898	-0.803890247
RP11-330L19.2	0.386909502	0.368970463	3.409438858	0.000987467	-0.806097738
SRP14	-0.401047573	6.252260934	-3.409210826	0.000988195	-0.806722053
C5orf42	0.375689788	1.416647424	3.408101764	0.000991742	-0.809758053
RP11-158H5.8	0.324539457	0.26700558	3.407249458	0.000994476	-0.812090698
SPEN	0.455944461	3.028381756	3.406839563	0.000995793	-0.813212369
AHCTF1	0.363834381	2.416648916	3.40673459	0.000996131	-0.813499612
IRAK1BP1	0.371896069	0.626502044	3.405859336	0.000998951	-0.815894343
PSIP1	0.629825471	3.684451337	3.404875139	0.00100213	-0.818586602
TYROBP	-1.054620024	6.187481804	-3.400482772	0.001016437	-0.830594827
RP11-466F5.6	0.266052112	0.176898479	3.400309731	0.001017004	-0.831067667
RP4-799G3.2	0.209568738	0.073222924	3.398749779	0.001022134	-0.835329465
CTSB	-0.627406708	6.958437428	-3.3980149	0.001024559	-0.837336654
LILRB5	-0.499965287	0.853051012	-3.396826167	0.001028493	-0.840582786
BX842568.4	0.323078489	0.386921999	3.395903196	0.001031557	-0.843102604
TXNRD1	0.48364451	3.800336922	3.394875233	0.00103498	-0.845908462
NCR3LG1	0.598641961	1.293695408	3.392870261	0.001041687	-0.851379285
CTD-2201E18.5	0.253388781	0.51999688	3.390265523	0.001050462	-0.858483056
RP11-165F24.2	0.303465256	0.170598706	3.387494464	0.001059874	-0.866035969
UQCRBP2	0.306880632	0.172223614	3.386594801	0.001062946	-0.868487139
CCL4L1	-0.568953176	1.069473968	-3.385199908	0.001067727	-0.872286627
TOPBP1	0.419415665	3.139280076	3.384087844	0.001071552	-0.875314893
ANP32BP1	0.409297708	1.152363909	3.384056828	0.001071659	-0.875399341
MFNG	-0.467087324	1.543641777	-3.38294477	0.001075498	-0.87842683
HMHA1	-0.529827704	2.377570233	-3.382339787	0.001077592	-0.880073538
AL390718.1	0.558372134	0.207041671	3.380319728	0.001084611	-0.885570356
MRC1	-0.729320504	1.850771663	-3.380283769	0.001084737	-0.885668183

PTAFR	-0.625587725	1.917051386	-3.380118374	0.001085313	-0.88611813
RP11-4M23.7	0.397188345	0.212193206	3.379790363	0.001086458	-0.887010418
CD63	-0.591910617	8.211765407	-3.378765711	0.00109004	-0.889797354
DIDO1	0.315846194	2.467220139	3.376026188	0.001099674	-0.897245458
RPL35AP	0.370982056	0.182100167	3.373980284	0.00110692	-0.902804836
RNU6ATAC11P	0.390873727	0.160328947	3.372973931	0.001110501	-0.905538498
TNFSF13B	-0.639061122	1.476319962	-3.368471482	0.001126655	-0.917761518
ETF1P2	0.250343995	0.315077136	3.367419089	0.001130463	-0.920616743
STRIP1	-0.330420348	2.631244422	-3.36653533	0.00113367	-0.923013937
RP11-55J15.2	0.210638582	0.142539808	3.36478471	0.001140048	-0.927761098
ACTB	-0.41513323	10.70632938	-3.362674246	0.001147781	-0.933481597
GIMAP4	-0.765076502	3.001996328	-3.362624138	0.001147965	-0.933617384
MSR1	-0.66182758	2.079533196	-3.361994584	0.001150282	-0.935323279
RP11-348P10.2	-0.283483563	0.841738998	-3.361402741	0.001152464	-0.936926768
RNU6-673P	0.441778163	0.161261258	3.361223985	0.001153124	-0.937411034
MIR4516	0.408846725	0.445382361	3.360487366	0.001155847	-0.939406395
SLFNL1-AS1	0.215349877	0.524977293	3.35757054	0.001166687	-0.947304308
RP11-2I17.1	0.213938327	0.118744631	3.35644792	0.001170885	-0.950342666
SS18L2	-0.396503576	2.932773707	-3.353896482	0.001180478	-0.957245278
SMAD9	0.719070067	2.172689245	3.353333141	0.001182606	-0.958768801
YY1P1	0.325205142	0.215907083	3.352037498	0.001187514	-0.962272062
CAV2	-0.524914109	1.879020196	-3.351928585	0.001187928	-0.962566503
HLA-DQB1	-0.979168834	3.103158331	-3.351795734	0.001188432	-0.96292565
GAPVD1	0.333296081	2.406923805	3.35146016	0.001189707	-0.963832787
RALBP1	0.409686398	3.944988371	3.349709577	0.001196381	-0.968563931
RP11-177J6.1	-0.372316734	0.810122341	-3.348059215	0.001202704	-0.973022519
EIF4EP1	0.277636119	0.381975311	3.347697878	0.001204093	-0.973998481
LMAN1	0.456813896	5.264082678	3.347490043	0.001204893	-0.974559802
ASH1L	0.507850153	2.801431055	3.347315446	0.001205565	-0.975031334
RP4-545K15.5	0.296510834	0.650217865	3.346117322	0.001210185	-0.978266594
AC005624.2	0.424516846	0.817452163	3.345722056	0.001211713	-0.979333728
HLA-DQB2	-0.600189285	0.999338858	-3.342716984	0.00122339	-0.987443697
RP11-144G7.2	0.224411307	0.170219143	3.342710496	0.001223415	-0.9874612

RNU6-1067P	0.5437871	0.301058613	3.34250378	0.001224222	-0.988018877
CREB3L2	0.436545187	3.661169948	3.341793953	0.001226998	-0.989933639
NDUFAF1	-0.435850923	2.998206912	-3.339469773	0.001236126	-0.996200993
LCP2	-0.517043615	1.737754949	-3.338815585	0.001238707	-0.997964478
TMEM176A	-0.772029819	1.882597082	-3.338440347	0.00124019	-0.998975885
TATDN2P2	0.249791211	0.506217574	3.337698162	0.001243127	-1.000976098
ITCH-AS1	0.300321983	0.252403309	3.336924946	0.001246194	-1.003059586
RP11-386G11.10	0.215505889	0.373272294	3.335783438	0.001250736	-1.006134803
RP1-202O8.2	0.323435544	0.193384898	3.334266092	0.001256796	-1.010221301
WASF4P	0.277287483	0.612081951	3.333634195	0.001259328	-1.011922705
RPL35AP3	0.342659204	0.163947324	3.333587011	0.001259517	-1.012049741
NANOGNBP2	0.218875766	0.145091426	3.332535312	0.001263743	-1.014880909
ATP6V0D1	-0.399761277	3.633296389	-3.332513787	0.001263829	-1.014938849
KIF14	0.456534378	1.826263315	3.332513645	0.00126383	-1.014939232
AC004470.1	0.31920785	0.135342751	3.331921725	0.001266214	-1.016532374
AC010287.1	0.253711386	0.261934019	3.331132997	0.001269397	-1.018654893
RNU1-85P	0.409187571	0.193176275	3.330594978	0.001271573	-1.02010252
IL7R	0.877654543	1.971604342	3.33012333	0.001273483	-1.021371421
PIWIL3	0.509137408	0.186170246	3.329599735	0.001275607	-1.022779918
SUMO2P16	0.245591656	0.202318377	3.329028903	0.001277927	-1.024315298
CTD-2532N20.1	-0.205612095	0.31827326	-3.326696828	0.001287443	-1.03058587
TMEM53	-0.305835252	1.437428935	-3.326615369	0.001287776	-1.030804841
L3MBTL2	-0.347192717	2.600384763	-3.322551386	0.001304523	-1.04172413
FCGR3A	-1.076478669	3.060164812	-3.322434194	0.001305009	-1.042038856
COMMD9	-0.367742562	2.462678151	-3.322349037	0.001305362	-1.042267547
RP11-159F24.6	0.438303538	0.625256001	3.322203442	0.001305966	-1.042658533
ASH1L-IT1	0.365441835	0.283983159	3.32052282	0.001312958	-1.047170808
BLOC1S1	-0.509723485	3.607225158	-3.318694267	0.001320606	-1.05207831
XK	0.20379017	0.312943617	3.317845965	0.001324168	-1.054354304
SETD1B	0.353754766	2.880134019	3.317003791	0.001327713	-1.056613423
RNU6ATAC19P	0.427780866	0.143248844	3.31687633	0.00132825	-1.056955298
FITM2	0.313007507	2.499409659	3.315387414	0.001334542	-1.060948119
DOK2	-0.62781405	2.328949842	-3.314553188	0.00133808	-1.06318467

MRFAP1L1	-0.447576961	4.387826364	-3.313694578	0.00134173	-1.065486151
UBE2L6	-0.725209893	4.159680015	-3.31272376	0.001345868	-1.068087863
RP11-274B21.1	0.405299195	1.143535304	3.311796794	0.001349831	-1.070571516
EBI3	-0.587279136	1.09492919	-3.310504222	0.001355375	-1.074033874
CTC-444N24.9	0.281472921	0.21615136	3.30782228	0.001366946	-1.081214628
RP11-263K19.4	0.326305251	0.772490196	3.307067945	0.001370217	-1.083233528
PGAP3	-0.466400533	2.673079972	-3.306447843	0.001372912	-1.084892905
AC092638.2	0.205153551	0.097997497	3.306314856	0.00137349	-1.085248743
RP11-214K3.24	0.235543441	0.196011649	3.303780765	0.001384558	-1.092027262
SOWAHD	-0.283428623	0.537197803	-3.303047458	0.001387776	-1.093988074
COL5A2	0.667539081	7.854303963	3.30304076	0.001387806	-1.094005983
CEP192	0.353079124	1.951046284	3.302287409	0.001391119	-1.096020048
MEIS1-AS2	0.24198386	0.198577082	3.302251108	0.001391279	-1.096117089
MS4A14	-0.314998481	0.605662333	-3.301303441	0.001395459	-1.098650138
RP11-595B24.1	0.286138358	0.118973987	3.299521461	0.001403349	-1.103411756
RP11-738O11.12	0.209809107	0.066732226	3.299097054	0.001405235	-1.104545525
RN7SL644P	0.336137756	0.285356176	3.295146826	0.001422898	-1.115092954
HADHAP1	0.290892038	0.53376832	3.293692041	0.001429456	-1.11897494
TP53TG5	0.306175661	0.525263897	3.293690572	0.001429462	-1.118978858
AC010323.1	0.265331126	0.116192151	3.293599661	0.001429873	-1.119221403
SLC7A5P1	0.355699456	0.753986619	3.292806113	0.001433463	-1.121338335
RP11-58G13.1	0.208401419	0.09953924	3.292529448	0.001434716	-1.122076297
RP11-97O12.6	0.230048167	0.339794618	3.292124643	0.001436552	-1.12315597
RDX	0.478543168	3.976172645	3.291231974	0.001440609	-1.125536489
GFAP	0.201911449	0.139753725	3.289685198	0.001447663	-1.129660189
RP11-576C12.1	0.435993753	0.407719921	3.28922785	0.001449755	-1.130879194
RNU4-58P	0.483678641	0.192382008	3.289094249	0.001450367	-1.131235268
WIBG	-0.371839107	2.964790472	-3.28888159	0.001451341	-1.131802024
NEXN-AS1	0.384367244	0.761600147	3.287575844	0.001457335	-1.135281351
LRRFIP1P1	0.25083196	0.637815438	3.285875069	0.001465178	-1.139811713
RP11-701H24.8	0.268139201	0.313800366	3.285744925	0.00146578	-1.140158304
RP11-449J10.1	0.236252786	0.181855268	3.285286387	0.001467902	-1.141379373
AC000120.7	0.273922517	0.370000429	3.284137119	0.001473233	-1.144439261

ALOX5	-1.006962886	1.746708757	-3.283336504	0.001476958	-1.146570392
POMK	0.544905397	2.412381295	3.282979528	0.001478622	-1.14752049
RP11-210K20.4	0.203385845	0.150663165	3.282142484	0.001482529	-1.149747984
SECTM1	-0.792270578	2.047059079	-3.278926746	0.001497632	-1.158301523
UBA7	-0.508619961	2.481441282	-3.278416023	0.001500044	-1.159659411
RP5-875O13.1	0.249306339	0.196022681	3.276442981	0.001509396	-1.164903735
RNA5SP35	0.458167384	0.204505762	3.275853235	0.001512201	-1.166470808
RP11-654A16.3	0.345536337	0.290587336	3.274959228	0.001516464	-1.168845953
RNA5SP30	0.352184022	0.140600531	3.27485868	0.001516944	-1.169113053
RP11-202G11.2	0.29441775	0.144710142	3.273670286	0.00152263	-1.172269474
HMOX1	-1.092873796	4.978137128	-3.273538905	0.00152326	-1.172618375
ZDHHC3	-0.297472757	2.561210889	-3.272890188	0.001526373	-1.174340973
PTGS1	-0.531463052	1.774690457	-3.272475658	0.001528365	-1.175441577
NHP2L1	-0.406353662	4.956208644	-3.271901747	0.001531128	-1.176965173
RP11-339B21.8	0.292928192	0.424593017	3.270032561	0.001540158	-1.181926009
RP11-369K16.1	0.37523882	0.385294299	3.268931092	0.001545502	-1.184848307
CTD-2534I21.9	0.346359745	0.140296322	3.26382234	0.001570519	-1.198392506
BABAM1	-0.387040329	3.890028193	-3.263549196	0.001571867	-1.199116206
EIF4EP3	0.216719227	0.119756364	3.261703809	0.001581003	-1.204004377
TMTC2	0.752062745	2.962462041	3.261452053	0.001582254	-1.204671082
CAMSAP2	0.425176419	3.022353933	3.259666036	0.00159115	-1.209399708
HOXB2	0.625440282	3.265474886	3.258846115	0.00159525	-1.211569857
FEZ1	0.633912288	1.516736219	3.25701368	0.001604449	-1.216418401
MYO1H	0.304689012	0.324084369	3.256858426	0.00160523	-1.2168291
OXCT1-AS1	0.297239385	0.439296071	3.256679505	0.001606132	-1.217302391
GLIS3	0.597883554	1.02259092	3.2552681	0.001613258	-1.221035199
SEPT7P3	0.258698018	0.28441337	3.254343252	0.001617943	-1.223480519
RP11-1028N23.4	0.288196189	0.115778267	3.25377406	0.001620833	-1.224985214
RP11-527L4.2	0.337467715	0.214291456	3.253460769	0.001622426	-1.225813331
P2RY6	-0.442671695	0.854755339	-3.253029773	0.001624619	-1.226952478
OR7E128P	0.277743929	0.471410374	3.25220744	0.001628812	-1.229125633
DDX19B	-0.317165227	1.981159566	-3.251300324	0.001633449	-1.231522355
RPL7P41	0.350386229	0.143629602	3.251225288	0.001633833	-1.231720589

CTD-2562G15.2	0.294027416	0.250122134	3.251174489	0.001634093	-1.231854789
RNU6-1116P	0.242189854	0.091498783	3.250970761	0.001635137	-1.232392978
RNU6-306P	0.524602081	0.300889387	3.250962984	0.001635177	-1.232413524
GGT8P	0.480907329	0.40225618	3.25040504	0.001638038	-1.233887316
RP11-30L15.4	0.290712166	0.344409093	3.248341214	0.001648661	-1.239337177
PPP1R2P3	0.254393315	0.527056202	3.24823643	0.001649202	-1.239613806
CLEC2B	-0.681193598	2.033141922	-3.247815134	0.001651379	-1.240725954
AC092309.1	0.449251887	0.215134202	3.24769026	0.001652025	-1.241055577
AC141586.5	0.350759961	1.320991704	3.247268795	0.001654207	-1.242168026
RNU6-116P	0.586946316	0.29374201	3.243993556	0.001671252	-1.250809216
RP11-621L6.2	0.257034484	0.217621061	3.241808562	0.001682715	-1.256570267
C7orf55-LUC7L2	0.365899761	2.962909542	3.240543045	0.001689388	-1.25990563
ZNF292	0.382943025	1.85115188	3.238822624	0.001698498	-1.26443833
NDUFA13	-0.576889936	3.817996191	-3.238015492	0.001702789	-1.266564203
KIAA0020	0.486612761	3.508447353	3.237548055	0.001705278	-1.267795181
RP5-1061H20.4	0.29512594	0.495025013	3.237207793	0.001707092	-1.268691164
BRPF3	0.465592398	2.892528277	3.236771368	0.001709421	-1.26984026
KNTC1	0.428842029	2.063195236	3.233901454	0.001724814	-1.277393711
LILRB1	-0.452395801	1.142342235	-3.233226495	0.001728453	-1.27916942
USP28	0.367903297	2.667613867	3.232362782	0.00173312	-1.2814413
PRKCD	-0.473352284	2.799826872	-3.232305153	0.001733432	-1.281592866
RP11-468N14.8	0.262916645	0.077679918	3.231517693	0.001737698	-1.283663741
RN7SL266P	0.201320466	0.071419959	3.231089579	0.001740021	-1.284789437
IFFO2	0.515415791	2.647062255	3.229584445	0.001748212	-1.288746182
RP11-184E9.1	0.327338795	0.23942378	3.228003263	0.001756857	-1.292901324
EEA1	0.387090051	1.926676288	3.227618235	0.001758967	-1.293912893
TLR8	-0.248015627	0.413293728	-3.225872767	0.001768567	-1.298497531
HNRNPUL2-BSCL2	0.432047661	1.012185388	3.22537912	0.001771291	-1.299793796
RP11-246E12.2	0.291628048	0.460403185	3.223706159	0.001780551	-1.30418569
LYPD1	0.670387833	1.380593026	3.223423613	0.001782119	-1.304927265
MST1R	0.405551767	0.389910012	3.222610705	0.001786638	-1.307060555
C1orf198	0.554372742	4.393535472	3.22179953	0.001791159	-1.309188886
KLHDC8B	-0.42433827	2.981388212	-3.221198039	0.001794518	-1.31076679

RP11-146F11.2	0.424328588	0.223236662	3.221139367	0.001794846	-1.310920696
RNU7-128P	0.613289635	0.347382974	3.219750395	0.001802626	-1.314563502
CEP295	0.320614652	1.067670984	3.217312922	0.001816356	-1.320953267
AC018892.3	0.221785032	0.08146312	3.216067585	0.001823408	-1.324216449
FNDC3B	0.380821037	3.731176502	3.214559484	0.001831983	-1.328166858
MIR1273A	0.404281864	0.200347422	3.214513429	0.001832246	-1.328287475
RNA5SP503	0.364656436	0.114618667	3.214273701	0.001833612	-1.328915296
ZNF543	0.346108342	1.722616426	3.214116169	0.001834511	-1.329327833
RP11-473N11.2	0.209993904	0.601033227	3.214066169	0.001834796	-1.329458768
RP11-567B20.1	0.435089334	0.146367955	3.213786817	0.001836391	-1.330190279
AC092069.1	0.222174951	0.124438162	3.213617228	0.00183736	-1.330634338
SP2-AS1	0.246016876	0.808804103	3.212729858	0.001842437	-1.332957583
HSPE1P16	0.273038879	0.106759238	3.212617685	0.001843079	-1.333251231
PCF11	0.338614999	2.25331423	3.211565903	0.001849116	-1.336004211
A4GNT	0.204232336	0.134300237	3.211414937	0.001849984	-1.336399302
snoMe28S-Am2634	0.37826613	0.33982248	3.211028522	0.001852207	-1.33741051
RP11-1023L17.1	0.22057109	0.432797155	3.210744936	0.00185384	-1.338152567
NPTN-IT1	0.291450324	0.566614116	3.210174344	0.001857131	-1.339645479
IQGAP3	0.535899764	2.599520088	3.210006065	0.001858102	-1.340085729
AOAH	-0.672411681	1.430126276	-3.209254295	0.001862448	-1.342052288
PCNT	0.425654191	2.449918107	3.208032146	0.001869533	-1.345248559
RP13-297E16.5	0.227273004	0.170833934	3.207785131	0.001870968	-1.345894461
BARD1	0.473438395	1.737194052	3.207740432	0.001871227	-1.346011336
AC096753.1	0.307494281	0.104719691	3.206062023	0.001881006	-1.35039903
RASAL2	0.374054566	2.16585956	3.20559238	0.001883751	-1.351626454
RP3-463P15.1	0.210725222	0.076683062	3.205069052	0.001886814	-1.35299402
RNU6-1137P	0.260288001	0.111646114	3.203287278	0.001897278	-1.357648889
RP11-804F13.1	0.228985229	0.082334263	3.20326925	0.001897384	-1.357695977
HLA-DRB6	-0.893592203	2.029879215	-3.202993455	0.001899009	-1.358416308
TTLL1	-0.430588964	2.326469969	-3.202721744	0.001900611	-1.359125926
PIK3IP1	-0.46950774	1.789157402	-3.202202228	0.001903677	-1.360482597
IPPK	0.244129706	1.039975531	3.202015878	0.001904778	-1.360969194
RP11-411H5.1	0.349024657	0.126278132	3.200465995	0.001913957	-1.365015395

CTD-2616J11.10	0.272247465	0.403522209	3.19864024	0.001924824	-1.36977987
C7orf66	0.407447668	0.213950374	3.195712124	0.001942372	-1.377416686
AC007787.3	0.405295871	0.435815269	3.195503004	0.001943631	-1.377961887
MGAT2	0.441270785	1.779845171	3.193398472	0.001956343	-1.383447112
GOLPH3	0.448398816	5.470905579	3.192439358	0.001962163	-1.385946012
AL391001.1	0.306737094	0.233113781	3.191990133	0.001964894	-1.387116237
TMCO2	0.254268606	0.118686243	3.189195783	0.001981962	-1.394392622
ENPP7P4	0.384558041	0.346170905	3.188656348	0.001985273	-1.395796726
RP11-546M21.6	0.225969809	0.232072665	3.188406453	0.001986808	-1.396447119
TMC8	-0.412924446	1.016271154	-3.188178311	0.001988211	-1.397040862
POU5F1P6	0.290103796	0.372115858	3.18557605	0.002004278	-1.403810974
RNU6-264P	0.281877237	0.122795974	3.185105803	0.002007194	-1.405033923
PRRC2B	0.537432699	3.770755172	3.184520246	0.002010831	-1.406556564
RNU7-50P	0.667085916	0.300821308	3.184096812	0.002013465	-1.407657496
RP11-312J18.7	0.32430918	0.15900372	3.183963979	0.002014292	-1.408002841
CD3E	-0.646387547	0.900356901	-3.183886422	0.002014775	-1.408204469
FGD2	-0.284833046	0.601146454	-3.182684531	0.002022273	-1.411328624
OCLM	0.345752787	0.615916105	3.182646089	0.002022513	-1.411428533
NPL	-0.488437719	2.011926969	-3.182113861	0.002025842	-1.412811685
CIDECP	-0.332141265	1.802345365	-3.181928335	0.002027003	-1.413293787
DPPA2	0.225926213	0.099931221	3.181373977	0.002030478	-1.414734195
ATP5G1P7	0.321812507	0.2359788	3.181065933	0.002032411	-1.415534513
CLIC4P1	0.316495795	0.578649355	3.181025628	0.002032664	-1.415639223
SNX4	0.44982339	3.895419423	3.179936434	0.002039514	-1.41846851
PHC3	0.322926884	1.649342255	3.179646679	0.00204134	-1.419221053
PLEKHO2	-0.467410724	3.936241306	-3.176190802	0.002063236	-1.428192447
LFNG	-0.745033911	3.855460368	-3.176157744	0.002063446	-1.428278231
PPFIA1	0.335320853	2.525879578	3.174710843	0.002072679	-1.432032093
AL359753.1	0.333283673	0.23087933	3.174620855	0.002073255	-1.432265514
NAA16	0.353272558	1.48555133	3.173928522	0.002077687	-1.434061206
RPL36P2	0.250945433	0.244113917	3.173823938	0.002078358	-1.434332438
KATNAL2	0.343686982	0.85876502	3.173810322	0.002078445	-1.434367751
PSMB10	-0.571361883	3.117635809	-3.172547217	0.002086558	-1.437642972

RP11-619L19.2	0.465463827	0.198093091	3.170282666	0.002101176	-1.443512409
RP11-571E6.3	0.246097241	0.096150947	3.164993095	0.002135696	-1.457209705
AC093388.3	0.396854769	0.876887736	3.163998644	0.002142244	-1.45978285
HTR1D	0.32062171	0.229341169	3.163771003	0.002143746	-1.460371784
CYCSP35	0.398281765	0.143168132	3.161509744	0.002158716	-1.466220144
AL160165.1	0.501994514	0.687493881	3.161205933	0.002160735	-1.467005653
RNU2-32P	0.391485156	0.159358379	3.1606324	0.002164551	-1.468488373
AC007191.4	0.322288572	0.753801503	3.15944219	0.002172489	-1.471564687
TPM3P2	0.334064891	0.16045074	3.159359926	0.002173039	-1.471777279
RP5-1182A14.5	0.217816518	0.206399557	3.158979698	0.002175581	-1.47275984
KDM4C	0.249707626	1.094151252	3.158343293	0.002179843	-1.47440419
RP11-410K21.2	0.38679802	0.179957977	3.158058372	0.002181753	-1.475140288
RP11-700F16.2	0.248169086	0.187168429	3.157803151	0.002183466	-1.475799611
RP11-867G23.3	0.238286675	0.531239542	3.157634204	0.0021846	-1.476236038
C5orf51	0.441989126	3.164800345	3.156987492	0.002188947	-1.477906464
CREBZF	0.572899506	3.09483384	3.156528863	0.002192035	-1.479090922
SMC4	0.551179301	3.524371066	3.156493008	0.002192277	-1.479183516
CTD-2318O12.1	0.517298697	0.696680242	3.156069703	0.002195131	-1.480276616
RCSD1	-0.44273153	1.293166664	-3.153464282	0.002212773	-1.487002096
SARS	-0.364814984	5.331588486	-3.152258394	0.002220983	-1.490113447
AL121932.1	0.604111841	0.310090771	3.15018526	0.002235165	-1.49546025
COX7BP2	0.384294759	0.396396264	3.149533867	0.002239638	-1.497139689
FCGRT	-0.537207879	4.903754685	-3.147007683	0.002257065	-1.503650216
AC009095.4	0.275860584	0.594454598	3.146265377	0.002262209	-1.505562531
RP11-641J8.1	0.380355126	0.48207404	3.146110968	0.002263281	-1.505960274
AC011453.4	0.380601748	0.133256162	3.14510142	0.002270299	-1.508560389
MIR4257	0.205228487	0.117191445	3.142411608	0.002289095	-1.515484906
LPAR6	-0.57821891	2.613748588	-3.141767421	0.002293618	-1.517142585
LINC01509	0.303317491	0.277860679	3.141678478	0.002294243	-1.517371441
CTD-2587H24.1	0.232112774	0.1683091	3.139750204	0.002307836	-1.522331762
CTD-2378E12.1	0.232642132	0.189393571	3.139422603	0.002310152	-1.523174251
RNF141	-0.270474225	1.866385631	-3.139013831	0.002313046	-1.524225394
CCR5	-0.473347893	0.864290066	-3.138659236	0.002315559	-1.525137135

IRF5	-0.40278357	1.180241292	-3.137849963	0.002321304	-1.527217654
AL354828.1	0.230086122	0.087231455	3.136482609	0.00233104	-1.530731967
CHCHD2P1	0.266091819	0.12804078	3.13583805	0.002335643	-1.532388172
RP11-274M17.1	0.218580512	0.134731094	3.135122702	0.002340762	-1.534225958
MIR5688	0.580232949	0.220931059	3.135112379	0.002340835	-1.534252478
KRR1P1	0.216635621	0.376180385	3.134819935	0.002342931	-1.535003693
AC019070.1	0.251270262	0.126233406	3.134228567	0.002347174	-1.536522608
DAND5	0.240317484	0.198990148	3.13271302	0.00235808	-1.540414231
RP11-255B23.1	0.259571701	0.764415413	3.132604621	0.002358862	-1.540692524
EIF4A1P11	0.27964537	0.270862951	3.132092779	0.002362557	-1.542006466
CDK12	0.299198824	2.191973595	3.131807159	0.002364621	-1.542739605
DFNA5	-0.415109949	1.540946637	-3.13076891	0.002372139	-1.545404178
NLRP9	0.216470364	0.088162502	3.130172959	0.002376464	-1.546933322
LL0XNC01-237H1.2	0.307106687	0.732092643	3.12994398	0.002378128	-1.547520796
RP11-16L9.1	0.313748471	0.388811569	3.129580457	0.002380772	-1.548453391
C9orf129	0.275393082	0.357124746	3.129430174	0.002381866	-1.548838907
MKLN1	0.299174024	2.034148083	3.128253883	0.002390443	-1.551855917
RP1-7G5.5	0.230187604	0.280228495	3.127449926	0.002396321	-1.553917437
RP11-218E20.5	0.299266036	0.412696051	3.127024934	0.002399434	-1.555007045
RP11-423F24.3	0.218232118	0.334036169	3.126829669	0.002400866	-1.55550763
LINC00630	0.23320979	0.585674862	3.126341767	0.002404446	-1.556758325
DNMT1	0.39472368	3.763716088	3.126205988	0.002405444	-1.557106353
HNRNPA1P57	0.264660951	0.219407567	3.125397589	0.002411389	-1.559178207
ASS1P12	0.239795334	0.419634395	3.123777931	0.002423343	-1.563327989
SLAMF8	-0.677359751	1.831128357	-3.123560721	0.00242495	-1.563884383
RNU1-8P	0.313686605	0.162784829	3.123104851	0.002428327	-1.565052016
BHLHA15	0.480052388	0.58610563	3.122725725	0.002431138	-1.566022983
RP4-724E16.2	0.292649276	0.508407572	3.121572691	0.002439707	-1.568975413
RP11-519G16.2	0.277983534	0.257698087	3.121507187	0.002440195	-1.569143117
ATP13A3	0.422694367	3.219470127	3.121337828	0.002441456	-1.569576695
MBNL1	0.334703259	3.584103382	3.119456569	0.002455506	-1.574391696
BST2	-1.060195743	4.989602987	-3.11894447	0.002459344	-1.575701998
LINC00266-1	0.215663019	0.093926164	3.118779675	0.00246058	-1.576123622

SCIMP	-0.269104203	0.60343519	-3.116689348	0.002476311	-1.581470162
LINC00886	0.202371983	0.375817169	3.116680609	0.002476377	-1.581492509
RNU6-319P	0.391250688	0.26756623	3.116586554	0.002477087	-1.581733012
SENP2	0.357721418	2.53300679	3.1155398	0.002485001	-1.58440923
ARHGEF3	-0.4005125	1.615419155	-3.114962218	0.002489379	-1.585885626
RN7SKP3	0.292706751	0.125059904	3.113008013	0.002504242	-1.590879311
HOXB3	0.428062539	1.704317064	3.112631298	0.002507117	-1.591841669
FAM174A	-0.324048019	3.066156074	-3.111650713	0.002514614	-1.594346252
IP6K2	-0.326593339	3.046597729	-3.111318076	0.002517162	-1.595195724
RPL7P61	0.296782263	0.212708961	3.110736235	0.002521624	-1.596681432
RP11-639B1.1	0.329871561	0.207281269	3.110564498	0.002522943	-1.597119913
KRT19P2	0.209359588	0.238148431	3.110564365	0.002522944	-1.597120254
CTD-2382H12.2	0.400703841	0.162214138	3.109233758	0.002533182	-1.600516943
RP11-708L7.6	0.313747006	0.205663891	3.10874038	0.002536988	-1.601776116
LOX	0.736867443	5.695223922	3.108528732	0.002538623	-1.602316226
RNASE6	-0.861434762	3.581205817	-3.107556191	0.002546145	-1.604797709
AC009299.4	0.237848866	0.131457519	3.107119658	0.002549528	-1.605911346
SIRPA	-0.559808403	3.126879885	-3.106852563	0.002551601	-1.606592672
RP11-277J6.2	0.22814701	0.18403068	3.104956231	0.002566358	-1.611428648
HK3	-0.464361451	1.178068399	-3.104542835	0.002569585	-1.612482575
ATAD2	0.672111374	3.449031013	3.104262794	0.002571774	-1.613196459
CTC-435M10.10	0.296041893	0.160790735	3.103793746	0.002575443	-1.614392047
NAGK	-0.371401722	3.257222217	-3.103663731	0.002576461	-1.614723427
WDR66	0.258863879	0.325146477	3.103499668	0.002577746	-1.615141573
RP11-80F22.4	0.353923001	0.125433793	3.102927277	0.002582235	-1.616600281
RP11-247I13.6	0.480339025	0.306629255	3.100930676	0.002597947	-1.621686869
FAM32A	-0.318036319	4.832029221	-3.100886737	0.002598294	-1.621798779
SRP19	-0.326762095	2.372087405	-3.100571607	0.002600783	-1.62260137
RP11-535M15.2	0.358807948	0.886144784	3.098792987	0.002614869	-1.627130056
AL357075.1	0.376019058	0.461758148	3.097684813	0.002623682	-1.629950638
AC083899.1	0.302408912	0.109770594	3.097136091	0.002628056	-1.631346983
CTC-453G23.4	0.275608159	0.220253466	3.095798852	0.002638743	-1.63474907
CEACAM20	0.224189127	0.089506268	3.095553326	0.00264071	-1.635373593

EGFLAM-AS2	0.243581365	0.128677207	3.094641633	0.002648024	-1.63769224
LYPLA1P3	0.213092899	0.519543493	3.094474263	0.002649369	-1.638117842
DPH5	-0.361338105	3.102246266	-3.094221096	0.002651405	-1.638761583
RNU6-1133P	0.381424668	0.172125113	3.094135524	0.002652093	-1.638979161
SRGAP2B	0.292523536	1.219546269	3.093924271	0.002653793	-1.639516282
RP11-791G16.5	0.236969419	0.099365634	3.093502319	0.002657192	-1.640589029
PDE1B	-0.317257862	0.545495287	-3.09333969	0.002658502	-1.641002458
AC004461.4	0.390634818	0.419755149	3.091946166	0.002669761	-1.644544313
SCO2	-0.425919156	3.369951735	-3.090622755	0.002680493	-1.64790681
KRT8P51	0.281713265	0.140526504	3.090472778	0.002681712	-1.648287797
RP5-849L7.1	0.263320788	0.181973936	3.088906206	0.002694474	-1.652266503
DGKD	0.303082186	2.10423019	3.088715908	0.002696028	-1.652749704
RNU2-47P	0.255139181	0.094322021	3.088554464	0.002697347	-1.653159622
TMPRSS9	0.537934236	0.774377087	3.088403899	0.002698578	-1.653541904
AC068724.2	0.239362362	0.105524242	3.088403561	0.00269858	-1.653542762
RP11-565A3.2	0.255580993	0.162599254	3.088091894	0.00270113	-1.654334029
RP11-116B19.2	0.448603892	0.238093738	3.087863082	0.002703003	-1.654914902
KLHL20	0.444195749	3.008459479	3.086741887	0.002712198	-1.657760737
AC009973.1	0.581495578	0.259356918	3.086272438	0.002716057	-1.658952059
FOCAD-AS1	0.399156337	0.344286241	3.086206121	0.002716602	-1.659120339
RNF169	0.315831912	1.408594058	3.085258407	0.002724409	-1.661524883
CTD-2301A4.3	0.234124747	0.274712846	3.085063109	0.00272602	-1.662020321
HMHB1	0.396428362	0.243506828	3.084946448	0.002726983	-1.66231626
TLR1	-0.351292159	0.993498493	-3.08402007	0.002734641	-1.664665924
CTA-221G9.11	0.251802256	0.101416755	3.083906895	0.002735578	-1.664952943
UNKL	0.305393503	1.325361663	3.082710877	0.002745498	-1.667985614
FPR1	-0.554879707	1.138499562	-3.082387788	0.002748183	-1.668804694
COX6B1	-0.465851274	6.954798812	-3.081380218	0.002756574	-1.671358603
MIR92B	0.357593663	0.43646732	3.0809971	0.00275977	-1.672329529
RP3-460G2.2	-0.839385742	1.7561028	-3.080950224	0.002760162	-1.672448319
TWF2	-0.394475204	4.56762976	-3.080945606	0.0027602	-1.672460022
ANKRD40	0.31323386	3.444400554	3.080906848	0.002760524	-1.672558238
RP11-472N13.3	-0.31166013	0.571041053	-3.07955202	0.002771858	-1.675990906

CECR1	-0.660888924	1.917361579	-3.078373556	0.002781753	-1.678975761
MTCO2P4	0.317864533	0.149252618	3.07777307	0.002786807	-1.680496351
C6orf99	0.400112887	0.572091784	3.07717712	0.002791831	-1.682005221
CTD-2201E9.4	0.290414169	0.235929666	3.07679199	0.002795083	-1.682980201
AP000889.2	0.261067593	0.095795761	3.076217513	0.00279994	-1.684434345
RP11-124N2.1	0.202605968	0.380173648	3.075544409	0.00280564	-1.686137869
MPHOSPH9	0.331034591	1.762461012	3.075379862	0.002807035	-1.686554267
RNU6-396P	0.323057503	0.25058734	3.075376614	0.002807063	-1.686562487
VN1R7P	0.277614328	0.122051966	3.074918386	0.002810951	-1.687721974
VENTX	-0.253283184	0.436322757	-3.074368777	0.002815622	-1.68911251
RP11-272P10.2	0.379544065	0.259985637	3.074356993	0.002815722	-1.689142322
ARL11	-0.310106412	0.730737477	-3.074259299	0.002816553	-1.689389471
MSRB2	-0.369659279	3.224389746	-3.072926057	0.002827917	-1.692761732
TSHZ3	-0.625741221	1.692068511	-3.071137052	0.002843232	-1.697284977
AP1B1	-0.378723224	4.049664318	-3.070672761	0.00284722	-1.698458534
RN7SKP30	0.356289736	0.416259367	3.07039269	0.002849627	-1.699166382
WASF3-AS1	0.20268515	0.080338553	3.069807965	0.00285466	-1.700644045
ALG1L11P	0.233210932	0.238573583	3.069307513	0.002858974	-1.701908568
SLC25A25	0.40372067	2.046912679	3.069103666	0.002860733	-1.702423592
FOXN3P1	0.203477599	0.14794548	3.067975204	0.002870489	-1.705274201
RNF181	-0.457913969	5.348348829	-3.065912535	0.002888401	-1.710482576
RP11-274H24.1	0.209564174	0.250026042	3.065238232	0.002894279	-1.712184641
BOLA3-AS1	0.491086512	1.69509565	3.064908596	0.002897156	-1.713016595
RP11-50B3.4	0.205239643	0.313906539	3.064839398	0.002897761	-1.713191231
ARL2BPP1	0.229135132	0.085394405	3.063636739	0.002908284	-1.716225918
RP11-697H9.3	0.248489923	0.126135949	3.063221316	0.002911927	-1.717273942
CASP1P2	0.235750416	0.126941874	3.062000218	0.00292266	-1.720353871
SVILP1	0.249091193	0.456059028	3.061964679	0.002922973	-1.720443495
RP4-741O10.1	0.261972906	0.252671511	3.061279237	0.002929014	-1.722171919
GACAT2	0.270914467	0.207700267	3.061241231	0.002929349	-1.722267747
TET2-AS1	0.312295364	0.289206485	3.060193844	0.002938604	-1.724908244
TRAPPC3	-0.309609995	4.143833256	-3.05989131	0.002941283	-1.725670811
RP11-409C19.2	0.230839178	0.316119024	3.059150909	0.002947847	-1.727536812

CCNH	-0.312234577	2.126811524	-3.058801821	0.002950947	-1.728416481
OSTN-AS1	0.210599224	0.12503659	3.058709751	0.002951765	-1.728648474
RP11-774D14.1	0.300823358	0.184189625	3.057674673	0.002960976	-1.731256245
IL1RL2	0.485856202	1.079566854	3.056492004	0.002971533	-1.734235006
YARS2	-0.409256827	3.025772021	-3.056197266	0.00297417	-1.734977215
AC007237.2	0.29347992	0.248306843	3.05570835	0.002978548	-1.736208277
NRROS	-0.476245641	1.760137647	-3.054617768	0.002988335	-1.738953741
FUCA1	-0.541160587	3.762757036	-3.054053512	0.002993411	-1.740373913
SIGLEC10	-0.36240889	0.79189815	-3.053734813	0.002996281	-1.741175954
MTMR2	0.387982193	3.489878022	3.053589635	0.002997589	-1.741541287
BRAF	0.275030057	1.848293771	3.053056613	0.003002398	-1.742882496
OXCT2P1	0.238628012	0.471936163	3.052350123	0.003008782	-1.744659907
TMED8	0.362192263	2.483283955	3.051729028	0.003014405	-1.746222211
TXNDC5	0.567040333	2.562549045	3.051670732	0.003014933	-1.746368836
AC007389.1	0.326172356	0.198223273	3.051418948	0.003017215	-1.747002093
GOLGA2P8	0.201745686	0.245839023	3.050216665	0.003028137	-1.750025364
RP11-29B9.2	0.281037084	0.145481654	3.050179849	0.003028472	-1.750117927
TMEM168	0.306972192	2.281376919	3.049323222	0.003036277	-1.752271415
PSMD10P3	0.211029002	0.180954346	3.048514971	0.003043658	-1.754302852
RP11-662B19.2	0.209501999	0.226631876	3.048277101	0.003045833	-1.754900628
HERC2P10	0.225124615	0.611814685	3.048012646	0.003048254	-1.755565168
LMBRD2	0.336554553	1.717353717	3.047704714	0.003051074	-1.756338905
C11orf85	0.240661419	0.179132953	3.047386262	0.003053993	-1.75713901
TMEM136	0.393523743	1.678895852	3.046109034	0.003065728	-1.760347358
NUP210L	0.264346078	0.209281378	3.04560516	0.003070369	-1.761612779
NRBP1	-0.292561361	4.757346659	-3.044851211	0.003077325	-1.763505925
SERPINI2	0.227306689	0.154048062	3.044822999	0.003077586	-1.763576756
RNU6-336P	0.294896745	0.162843837	3.044599664	0.003079649	-1.764137471
BRI3	-0.382375756	3.458366815	-3.043941708	0.003085736	-1.765789171
AC012363.1	0.440651421	0.226331563	3.043299874	0.003091685	-1.767400128
RP11-350E12.4	0.444269922	0.244192306	3.04313049	0.003093256	-1.767825224
BAIAP2L2	0.788375389	1.742654893	3.043062319	0.003093889	-1.767996306
RP11-115M14.1	0.30218603	0.123236518	3.042426615	0.003099795	-1.769591515

TXN2	-0.43071175	4.93202532	-3.041468192	0.003108719	-1.771996042
EPHX3	0.262082347	0.471698337	3.041422776	0.003109143	-1.77210997
SRPK1	0.491905736	3.23031986	3.041269918	0.003110568	-1.772493405
LINC00471	0.336174603	0.635669304	3.040031636	0.00312214	-1.775599009
FKTN	0.312362087	2.073270423	3.039689605	0.003125343	-1.776456646
RNU6-1151P	0.495504047	0.194315205	3.039361221	0.003128421	-1.777279991
AC024937.2	0.297858077	0.334220853	3.037941153	0.003141766	-1.780839662
KIAA1456	0.352129027	0.523012912	3.03763386	0.00314466	-1.781609778
MUS81	0.33860694	2.503882254	3.037317681	0.003147641	-1.782402099
KMT2C	0.369352979	2.073688512	3.03728436	0.003147955	-1.782485596
EFTUD1P1	0.255537613	0.181753885	3.037154054	0.003149185	-1.782812112
FYB	-0.607892316	1.628634914	-3.037113703	0.003149566	-1.782913219
RNA5SP72	0.399978908	0.164114564	3.036626404	0.003154167	-1.784134161
CTD-3022G6.1	0.250148121	0.101296065	3.036295447	0.003157296	-1.784963295
RP11-92G12.3	0.391058577	0.475423813	3.036060458	0.00315952	-1.785551963
SAT1	-0.54819153	5.688555799	-3.036004832	0.003160046	-1.785691305
GAL3ST4	-0.506453689	2.938237947	-3.035695514	0.003162976	-1.786466104
TMEM179	0.902332187	1.311638735	3.034158734	0.003177567	-1.790314609
RP11-945F5.1	0.449490545	0.218110878	3.033584614	0.003183034	-1.79175196
PSKH2	0.211482756	0.112907587	3.033356111	0.003185212	-1.792323977
RP11-430B1.1	0.610727704	0.887287186	3.031996369	0.003198203	-1.795727131
RP4-597A16.3	0.324339159	0.144567743	3.0316717	0.003201313	-1.796539534
ATP9B	0.25105946	1.236177041	3.031374947	0.003204157	-1.797282024
FAM83D	0.520073002	3.42154197	3.031039733	0.003207373	-1.798120674
POLQ	0.290416871	1.033427065	3.030877191	0.003208933	-1.798527301
TFAP2D	0.28001211	0.123756842	3.030780477	0.003209862	-1.79876924
PLTP	-0.748143097	5.600204859	-3.030481749	0.003212732	-1.799516497
RMI1	0.357685217	2.423489444	3.028595827	0.003230908	-1.804232713
POLR3GP2	0.313290495	0.163690495	3.027637631	0.003240179	-1.806628029
XRCC6P1	0.230394491	0.362006254	3.027280373	0.003243642	-1.807520957
XXbac-BPG34I8.3	0.240726227	0.093905969	3.027063888	0.003245743	-1.808061997
RP11-496H15.2	0.351965378	0.26253154	3.026657464	0.003249689	-1.809077648
EPB41L3	-0.592194899	1.602758162	-3.025877408	0.003257275	-1.811026705

NRBF2P5	0.225837653	0.448089959	3.025779399	0.003258229	-1.811271564
COX4I1P1	0.322678619	0.35793271	3.025554428	0.003260421	-1.811833592
PKD1P6	0.401753276	1.386353386	3.025516652	0.003260789	-1.81192796
CHML	0.675421517	2.059227452	3.024766392	0.003268109	-1.813802016
RP1-223H12.5	0.537472257	0.235545525	3.024178445	0.003273855	-1.815270378
RP11-367E12.4	0.412100099	1.111216892	3.023323251	0.00328223	-1.81740577
KIAA1033	0.305718497	2.699303415	3.023233113	0.003283114	-1.817630812
GON4L	0.30290777	2.151100236	3.022735852	0.003287995	-1.818872206
RP11-544D21.2	0.221088724	0.075678008	3.022671459	0.003288627	-1.81903295
RP1-102G20.2	0.405102124	0.257985249	3.022310304	0.003292176	-1.819934445
RHOD	0.975698691	3.143099498	3.022286952	0.003292406	-1.819992731
TMEM86A	-0.42177418	1.726222509	-3.021214866	0.003302964	-1.822668291
INCENP	0.38433632	3.069393887	3.020980226	0.003305279	-1.823253774
RP11-93G23.2	0.202313666	0.097985337	3.020698751	0.003308057	-1.82395607
CTD-2647L4.4	0.429391784	1.485018549	3.020584259	0.003309188	-1.824241721
RPL7AP2	0.308801931	0.305953984	3.020415827	0.003310853	-1.824661931
VAV2	0.376501747	3.193885755	3.019925209	0.003315706	-1.825885838
CDHR3	0.259757617	0.693865138	3.019589989	0.003319025	-1.826721998
RP11-330C7.4	0.345369589	0.172322214	3.019568921	0.003319234	-1.826774546
LINC01584	0.210561333	0.106920071	3.019385627	0.00332105	-1.827231712
TMSB10	-0.535297294	10.92270055	-3.018112344	0.003333694	-1.830406891
RN7SKP247	0.302384827	0.112009691	3.017550529	0.003339287	-1.831807547
DIAPH3	0.389960123	1.525473449	3.016999303	0.003344783	-1.833181606
ATG7	-0.240229274	1.645827224	-3.016858516	0.003346188	-1.833532517
FARP1	0.396929306	1.607998281	3.016504259	0.003349726	-1.834415446
RNU7-148P	0.334376476	0.119120459	3.016465934	0.003350109	-1.834510961
RP11-98F14.11	0.402532248	0.441804632	3.016155034	0.003353217	-1.835285753
ERMP1	0.497863709	2.081295285	3.015820715	0.003356562	-1.836118838
RNU6-1158P	0.260359447	0.126309875	3.015678913	0.003357982	-1.836472172
RP11-120E11.2	-0.329409974	0.909793005	-3.014978863	0.003364999	-1.838216315
GNAT3	0.292880567	0.245819832	3.014794511	0.003366849	-1.838675565
RNU6-208P	0.374090008	0.20623358	3.014537602	0.003369429	-1.83931553
RPL21P18	0.257025681	0.266557281	3.014450752	0.003370302	-1.839531866

RNU7-159P	0.219697931	0.08342889	3.013074436	0.003384158	-1.842959483
CTD-2313N18.7	0.259219912	0.401993375	3.012250674	0.003392477	-1.845010409
RP11-474P12.3	0.213993041	0.242608316	3.012061046	0.003394394	-1.845482465
LAIR1	-0.584560415	1.912595568	-3.011694451	0.003398105	-1.84639499
HMGB3P5	0.267461255	0.13609031	3.011494432	0.00340013	-1.84689284
RP11-573D15.2	0.276523443	0.191531599	3.011394243	0.003401145	-1.847142201
AL355480.3	0.38229507	0.501442356	3.011252469	0.003402582	-1.847495053
CTB-180A7.3	0.227337377	0.116182838	3.011081062	0.003404321	-1.847921636
RP11-498P14.4	0.226956512	0.119397336	3.011018589	0.003404954	-1.84807711
ATG10-IT1	0.21040533	0.119246883	3.01093347	0.003405818	-1.848288939
RNU6-1242P	0.564832922	0.601801981	3.010442147	0.003410806	-1.849511553
KLHL2	0.497725159	2.878703255	3.009658086	0.003418781	-1.851462293
H2AFZP1	0.423485902	0.629284994	3.009506673	0.003420323	-1.851838961
FKBP1B	-0.420531042	1.241021431	-3.008537725	0.003430207	-1.854249046
WHSC1L1	0.397740292	2.561580356	3.00769861	0.003438787	-1.856335696
ATP5F1	-0.352211966	5.27390284	-3.007076559	0.003445161	-1.857882268
STX17	0.290119448	1.750159415	3.006988094	0.003446068	-1.858102193
PLEK	-0.652106386	2.500926153	-3.006747565	0.003448536	-1.858700127
ANKRD22	-0.455832232	0.515088095	-3.006456589	0.003451524	-1.859423416
AL359771.1	0.301382809	0.138866985	3.006250533	0.003453642	-1.859935582
CERS5	-0.243424468	3.024412014	-3.006107964	0.003455107	-1.86028993
MIR548N	0.342529652	0.274762402	3.005182031	0.00346464	-1.862590971
MCM6	0.422920488	4.527770411	3.004838634	0.003468182	-1.863444205
RP11-2N5.2	0.358326367	0.162071579	3.004326822	0.003473467	-1.864715754
CCL28	0.518166383	1.956796032	3.003816501	0.003478744	-1.865983429
RP11-317B3.2	0.220477172	0.134543227	3.003700453	0.003479945	-1.866271676
RP11-172F10.1	0.243162029	0.122193799	3.003003771	0.003487163	-1.868001955
TJP1	0.465927161	3.429979195	3.002889097	0.003488352	-1.868286728
DPPA2P4	0.219186701	0.116136342	3.00284381	0.003488822	-1.868399189
ASXL2	0.253644104	1.932903595	3.002517655	0.003492208	-1.869209083
WAS	-0.667143928	2.538619847	-3.002229243	0.003495204	-1.869925196
LINC00368	0.205577564	0.098998756	3.00175191	0.003500168	-1.871110273
RN7SL150P	0.532953611	0.1930326	3.000604148	0.003512131	-1.873959214

NPM1P30	0.230765731	0.133472131	3.000135505	0.003517027	-1.875122217
RP11-678G14.5	0.268863824	0.231072334	2.999992773	0.003518519	-1.875476397
RP4-580N22.2	0.356824499	0.556432782	2.999501099	0.003523664	-1.876696353
RP11-548H3.1	-0.322773457	0.983089832	-2.999001227	0.003528902	-1.877936489
CTB-102L5.8	0.231530512	0.240614844	2.998708086	0.003531977	-1.878663665
CD3D	-0.651942868	0.908788712	-2.998290037	0.003536366	-1.8797006
RP11-271F18.4	0.280435331	0.115917716	2.996646003	0.003553678	-1.883777361
OSTCP4	0.288755589	0.556375563	2.995340016	0.003567486	-1.88701459
RP11-651L5.3	0.206007675	0.395142411	2.995113741	0.003569883	-1.887575359
GPR65	-0.381267999	0.801418989	-2.994971582	0.00357139	-1.887927647
ANXA13	0.374219736	0.326798703	2.994775124	0.003573474	-1.888414475
BNIPL	0.31854547	0.495104358	2.993799023	0.003583842	-1.8908329
NBEAL1	0.224109834	0.840524714	2.993666574	0.003585251	-1.891161014
MIR6895	0.278914276	0.300641132	2.992685352	0.003595706	-1.89359141
CLPS	0.217676497	0.11667089	2.99207522	0.003602221	-1.89510233
MYO7A	-0.245998768	0.6886777	-2.991893177	0.003604167	-1.89555309
NCF2	-0.476033851	1.845255206	-2.991080668	0.003612864	-1.897564696
RP11-47P18.2	0.381799333	0.155906822	2.990602344	0.003617993	-1.898748724
RP11-15E18.1	0.34964006	0.78056772	2.990507593	0.00361901	-1.89898325
RP11-249C24.12	0.209082507	0.126964622	2.990033275	0.003624104	-1.900157184
AP000868.1	0.257309427	0.108223505	2.989555124	0.003629246	-1.901340454
SMC2	0.431526716	3.205516913	2.989547123	0.003629333	-1.901360253
ARHGAP25	-0.358450478	1.341577764	-2.9886903	0.003638564	-1.903480235
AL033528.1	0.327532641	0.107394818	2.988239908	0.003643425	-1.904594416
KLHL11	0.39395341	1.466302355	2.988055627	0.003645416	-1.905050252
RP11-613F22.7	0.224628649	0.106557538	2.9872317	0.003654329	-1.907088041
AL136380.1	0.598890032	0.433921897	2.986616918	0.003660993	-1.908608267
RPRML	0.207633106	0.209730341	2.986304823	0.00366438	-1.909379916
RP11-84O12.3	0.222572482	0.129198131	2.986200334	0.003665515	-1.909638249
RPGRIP1L	0.237308406	1.083541319	2.98527455	0.003675582	-1.911926793
RP3-364H10.1	0.327481367	0.103428546	2.985147301	0.003676968	-1.912241308
SYNJ2-IT1	0.251084289	0.125956137	2.984744623	0.003681356	-1.91323652
RP11-968O1.5	0.225257073	0.367487097	2.984665606	0.003682218	-1.913431798

IL18	-0.44691661	1.320226801	-2.984185704	0.003687455	-1.914617702
DENND1C	-0.333366113	0.941603542	-2.984180662	0.00368751	-1.914630161
INTS4P2	0.249105545	0.383975876	2.983287407	0.003697277	-1.916837112
RP1-172I22.1	0.342596058	0.174797915	2.982997308	0.003700455	-1.917553742
UBE2D3P2	0.2735098	0.761616739	2.982524201	0.003705641	-1.918722336
AC021231.1	0.212667757	0.09441265	2.981902213	0.003712471	-1.920258448
TMEM38B	0.398161483	2.477448717	2.98086578	0.003723877	-1.92281754
RP11-190P13.2	0.234154366	0.138304547	2.980536624	0.003727506	-1.923630122
RN7SL372P	0.395068171	0.157287785	2.98044803	0.003728483	-1.92384882
RNU6ATAC38P	0.361279471	0.166277554	2.979001056	0.003744479	-1.927420006
VKORC1L1	0.322326016	3.513495677	2.978988652	0.003744616	-1.927450613
CIT	0.369049079	1.922252862	2.978764039	0.003747105	-1.928004842
RNU6-563P	0.213235662	0.130234101	2.978716834	0.003747629	-1.928121314
STIL	0.396937968	2.06058259	2.97798386	0.003755762	-1.929929656
COTL1P2	0.240757776	0.143543367	2.977902936	0.003756661	-1.930129285
EDN2	0.399584386	0.227621794	2.977087546	0.00376573	-1.932140498
RP11-246A10.1	0.280890974	0.179296862	2.976785229	0.003769098	-1.932886071
IKBKAP	0.329700439	3.339643194	2.976096341	0.003776782	-1.934584778
IGSF6	-0.551964325	2.157841233	-2.974954611	0.00378955	-1.937399443
RP11-316M1.3	0.21919657	0.352792192	2.973407195	0.003806917	-1.941212856
BRK1	-0.365027655	6.735798467	-2.973278827	0.003808361	-1.941529132
PGPEP1	-0.363350172	2.150862283	-2.973189689	0.003809364	-1.941748746
RNU6-346P	0.329793668	0.145775283	2.973112174	0.003810237	-1.94193972
MIR4634	0.414640742	0.140469093	2.972738172	0.003814449	-1.94286109
CH17-340M24.3	-0.516188373	2.207244397	-2.97065596	0.003837978	-1.947989021
RNU6-183P	0.201913167	0.091724062	2.969649124	0.003849404	-1.950467559
CASZ1	0.297758791	0.521644632	2.968933674	0.003857541	-1.952228383
RP11-631F7.1	0.223574474	0.178362061	2.968706335	0.003860131	-1.952787824
AC144573.1	0.453022949	0.179455807	2.9679947	0.003868246	-1.954538814
UBN2	0.260842511	1.443321364	2.967933017	0.00386895	-1.95469057
RP11-1394O16.1	0.205020917	0.13855559	2.967849184	0.003869907	-1.954896819
LA16c-390E6.3	0.298730065	0.228406608	2.967238053	0.003876892	-1.956400191
DNAH10	0.379109839	0.646139568	2.967185194	0.003877497	-1.956530211

C5AR1	-0.6116134	2.354298002	-2.966998711	0.00387963	-1.9569889
AP000253.1	0.275912603	0.260579385	2.966821901	0.003881655	-1.957423775
AC007064.22	0.211004121	0.083244069	2.965731001	0.003894166	-1.960106455
RP11-469A15.2	0.249993153	0.138748401	2.965490486	0.003896929	-1.960697811
FKSG68	0.425542522	0.248674725	2.964927261	0.003903407	-1.962082464
AXDND1	0.215038778	0.11268247	2.964751103	0.003905435	-1.962515495
RP11-711K1.8	0.421296135	0.196397813	2.964673661	0.003906327	-1.962705855
RNU6-980P	0.396126415	0.149154526	2.964595045	0.003907233	-1.962899098
DCD	0.20059458	0.079951418	2.964475414	0.003908611	-1.963193151
AC102953.6	0.430870641	0.342671858	2.964305164	0.003910574	-1.963611606
ENTPD2	0.33463477	0.318742108	2.963873134	0.003915558	-1.964673404
CEBPA-AS1	-0.370218861	0.73096155	-2.963719271	0.003917335	-1.965051522
AL160279.1	0.2217822	0.101154028	2.963707185	0.003917474	-1.965081225
SNORD114-18	0.347033809	0.137728657	2.963579072	0.003918954	-1.965396049
CTSH	-0.672788898	3.081713047	-2.963102428	0.003924464	-1.966567256
RPS10P21	0.226390108	0.107243436	2.962957018	0.003926147	-1.96692453
ST14	-0.525248028	1.725315768	-2.961886043	0.003938559	-1.969555474
RNU6-1155P	0.32151652	0.131546198	2.96144134	0.003943723	-1.970647705
RAB11FIP1	0.615361289	1.599584269	2.961233077	0.003946144	-1.971159171
F11-AS1	0.214150221	0.124131116	2.96099236	0.003948944	-1.971750305
RP11-848P1.2	-0.370756944	1.184838498	-2.959873542	0.003961981	-1.974497309
RYR2	0.217967683	0.15408608	2.959364677	0.003967923	-1.975746435
HMGA1P8	0.376020229	0.765037795	2.958590022	0.003976986	-1.977647676
TNFRSF1B	-0.557737248	3.115285991	-2.958424589	0.003978923	-1.978053645
GCA	-0.440403592	1.359885989	-2.957841401	0.003985762	-1.979484643
PRKAA1	0.385203301	2.993697631	2.956378391	0.004002964	-1.983073503
CELF2	-0.555628756	1.404439184	-2.956369676	0.004003066	-1.983094876
PGM3	0.430019603	3.046794231	2.955077925	0.004018312	-1.986262439
RP11-15E18.5	0.416259711	0.958894894	2.95495331	0.004019786	-1.986567955
KCTD17	-0.348521988	3.071493535	-2.954659976	0.004023257	-1.987287076
NAA25	0.316738446	1.948269095	2.954320083	0.004027282	-1.988120265
DLGAP1	0.212038787	0.165292978	2.95389663	0.004032302	-1.98915818
RP11-534C12.1	-0.406589595	1.112952946	-2.953887573	0.004032409	-1.989180376

SRGAP2D	0.373130857	1.343895967	2.953701737	0.004034614	-1.989635836
RP1-161N10.1	0.216009445	0.154891234	2.95341815	0.004037981	-1.990330824
KLC2	0.34566434	2.720092563	2.953252347	0.00403995	-1.990737134
KLHDC10	0.321373471	3.10358433	2.953138651	0.004041301	-1.991015744
RALGPS2	0.66586065	1.691286172	2.952961775	0.004043404	-1.991449157
RNU4-61P	0.528918304	0.23835014	2.952122761	0.004053393	-1.993504767
TSC22D3	-0.605154085	4.263689514	-2.952067643	0.00405405	-1.993639792
HAUS6	0.335886532	1.965680524	2.95157757	0.004059895	-1.994840255
RN7SL74P	0.283255734	0.09581768	2.951459363	0.004061307	-1.995129784
HELB	0.281542602	1.076208442	2.951266379	0.004063611	-1.995602451
RPL35AP32	0.5470833	1.472386808	2.951010927	0.004066664	-1.996228079
RPS16P9	0.294540987	0.343419831	2.950990037	0.004066914	-1.996279239
SLC7A1	0.477313542	3.249980445	2.950862199	0.004068442	-1.996592309
MSRA	-0.420491522	1.362809083	-2.950620348	0.004071336	-1.997184562
MRPS36P4	0.350271355	0.15539298	2.950085545	0.004077741	-1.99849407
RP4-761J14.10	-0.333129572	0.892976318	-2.946909652	0.004115967	-2.006266576
CKS1BP6	0.337120487	0.550718278	2.946903056	0.004116047	-2.006282712
RP11-582E3.6	-0.348847509	1.75049934	-2.946689106	0.004118634	-2.00680608
MLLT1	0.372228719	4.797788557	2.946514732	0.004120743	-2.007232613
RP11-458D21.1	0.302615742	0.524350777	2.945884983	0.004128371	-2.008772864
TMEM154	0.216364055	0.388412518	2.945812708	0.004129247	-2.008949619
LINC01492	0.269779799	0.104526085	2.945495131	0.004133099	-2.009726239
CTD-2201E18.3	0.349991537	1.738324296	2.944534658	0.004144769	-2.012074623
YWHAH	-0.360133179	5.619847678	-2.943931865	0.004152109	-2.013548155
RP11-175P13.2	0.328853995	0.382017785	2.9437318	0.004154548	-2.014037162
CTD-2622I13.3	0.251752193	0.449598523	2.94365983	0.004155425	-2.014213067
LIN28A	0.224825716	0.116245417	2.94312277	0.004161979	-2.015525615
RNY1P7	0.281908011	0.096882574	2.942891592	0.004164804	-2.016090543
CTD-2537I9.13	0.263886748	0.425582285	2.942532622	0.004169193	-2.016967685
RP11-302F12.3	0.461910898	0.185724277	2.942394828	0.004170879	-2.017304359
SEPHS1P6	0.339540719	0.573379877	2.942048066	0.004175124	-2.018151558
RNU7-97P	0.68949945	0.338012301	2.941945154	0.004176385	-2.018402974
CHD6	0.337271286	2.089815736	2.941597848	0.004180642	-2.019251397

ARHGAP18	-0.447284061	2.400385841	-2.940728596	0.004191314	-2.021374514
RP11-6J21.2	0.296840732	0.105810191	2.940682946	0.004191876	-2.021486
HNRNPKP2	0.367350241	1.384463063	2.94034542	0.004196027	-2.022310249
ARHGEF33	0.255073651	0.374188591	2.939614602	0.004205029	-2.02409467
CCL4	-0.502592419	1.039387816	-2.939437289	0.004207215	-2.024527558
AC010894.3	-0.323596001	0.866566904	-2.938672782	0.004216656	-2.026393765
RP3-327A19.7	-0.373620278	0.697651702	-2.938608592	0.004217449	-2.026550439
RP11-958N24.1	0.260214869	0.637885409	2.938532354	0.004218392	-2.026736515
PPM1M	-0.346223028	2.432262875	-2.937935271	0.004225781	-2.028193704
BPIFB1	0.253840115	0.107221828	2.937831375	0.004227068	-2.028447239
FKBP8	-0.352075082	6.149953506	-2.937000182	0.004237377	-2.030475322
MIR3126	0.455484484	0.436494023	2.936937454	0.004238156	-2.030628358
TUBA1A	-0.523482197	6.223616348	-2.936902198	0.004238594	-2.030714372
RP11-178F10.1	0.257582556	0.328876481	2.936594143	0.004242422	-2.031465878
RP11-90C4.1	0.32850345	0.134188284	2.936556192	0.004242894	-2.031558456
C6orf1	-0.400440295	2.968180871	-2.934384615	0.00426997	-2.036854216
RP11-214K3.5	0.267455601	0.179057154	2.934213074	0.004272115	-2.037272413
AL138807.1	0.455644611	0.247501091	2.933898498	0.004276053	-2.038039265
RP11-893F2.5	0.282279517	0.558501823	2.933673458	0.004278871	-2.038587809
RP11-212D3.2	0.258647282	0.281192805	2.933522349	0.004280765	-2.038956127
RP11-219E7.1	-0.496491529	1.151270122	-2.933428875	0.004281937	-2.039183954
RPS6KA1	-0.400370615	2.263972671	-2.933188302	0.004284954	-2.039770286
RPS12P2	0.236104747	0.114697549	2.93261366	0.004292169	-2.041170663
KLHL2P1	0.266300626	0.36914168	2.931819482	0.004302158	-2.043105677
RP11-3K24.2	0.329329225	0.18809536	2.93155646	0.004305471	-2.043746437
RNU6-625P	0.313116785	0.201013795	2.931204509	0.004309908	-2.04460377
RPS6P21	0.205292972	0.152430736	2.930737788	0.004315799	-2.045740547
CEACAM22P	0.205246346	0.114496137	2.930278298	0.004321605	-2.046859571
RN7SKP117	0.330138631	0.178223195	2.930034272	0.004324692	-2.047453803
RP11-397P13.6	0.285275924	0.451727157	2.929976808	0.004325419	-2.047593729
ZNF107	0.286859526	1.269500471	2.9297746	0.004327979	-2.048086095
MNS1	0.546189272	2.29192732	2.92968834	0.004329071	-2.048296124
RP11-142O6.1	0.286156116	0.294543607	2.929394246	0.004332797	-2.04901216

BX322557.10	0.50318846	1.964403606	2.928780135	0.004340587	-2.050507158
FIS1	-0.441545938	4.905978772	-2.928329007	0.004346318	-2.051605228
CTA-384D8.36	-0.493104175	2.411943664	-2.927564159	0.00435605	-2.053466599
RP1-177I10.1	0.206823679	0.092330854	2.926937158	0.004364043	-2.054992206
MSANTD2P1	0.204982507	0.076141772	2.926519674	0.004369373	-2.056007877
RP5-1119A7.14	0.475052715	0.331789878	2.926272693	0.004372529	-2.056608687
TFR2	0.468896192	1.36186095	2.925439461	0.004383191	-2.058635316
RNA5SP97	0.304104841	0.13831799	2.925226106	0.004385925	-2.059154175
RP11-209A2.1	0.398354764	0.387371178	2.924711145	0.00439253	-2.060406385
CTB-4E7.1	0.894330775	1.243966454	2.924547739	0.004394628	-2.060803695
XXbac-B562F10.11	0.26995658	0.835290799	2.923802132	0.004404212	-2.062616362
GPX1	-0.570476338	7.073577961	-2.923504465	0.004408044	-2.063339925
AC121336.1	0.359842817	0.178357933	2.922833605	0.00441669	-2.064970419
RP11-346L1.2	0.356422769	0.149116654	2.920649272	0.004444953	-2.07027726
RP11-732A21.3	0.369395304	0.816270235	2.920263208	0.004449966	-2.07121487
SNORA73	0.402310697	0.180542338	2.920113081	0.004451916	-2.071579447
RP11-473P24.2	0.287246551	0.40820393	2.919946603	0.00445408	-2.071983715
YEATS2	0.319061237	2.797530131	2.919680788	0.004457537	-2.07262917
FPR3	-0.728909813	2.764106517	-2.919093776	0.004465181	-2.074054392
TNPO2	0.435332593	3.895280013	2.918973139	0.004466753	-2.074347263
RP11-736N17.9	0.678606367	0.753738614	2.917900085	0.004480761	-2.076951874
RP11-9E13.4	0.351486403	0.463784848	2.917861701	0.004481263	-2.077045028
ATPAF2	-0.561048094	2.399429899	-2.916451195	0.00449974	-2.080467528
SETP5	0.212168403	0.234489869	2.916390254	0.004500539	-2.080615366
CTD-2535P7.1	0.224491164	0.081096883	2.915914373	0.00450679	-2.081769739
AC009081.1	0.231188336	0.078207004	2.915508752	0.004512124	-2.08275356
RP11-305E17.4	0.235659124	0.143035282	2.915414477	0.004513365	-2.082982203
RP11-2P2.1	0.379265291	0.225375779	2.915241827	0.004515637	-2.083400915
NANOGP5	0.224972224	0.305593571	2.915139397	0.004516986	-2.083649321
RNU6-740P	0.277101326	0.081870846	2.914976492	0.004519132	-2.084044369
RP11-500K19.2	0.312027038	0.158965298	2.913014007	0.004545059	-2.088802053
PIK3CD	-0.499027769	1.581904546	-2.912390015	0.004553331	-2.090314264
AP000692.10	0.275592806	0.543749359	2.912317857	0.004554289	-2.09048912

RP11-123C21.2	0.256172161	0.189086307	2.911687248	0.004562664	-2.092017072
COPS7A	-0.407060957	4.610958109	-2.91167754	0.004562793	-2.092040593
SNORA1	0.323857049	0.215193874	2.911611455	0.004563672	-2.092200699
RPL35AP33	0.284956027	0.345350501	2.911557113	0.004564395	-2.092332354
RHOC	-0.389391434	5.9473506	-2.911063154	0.004570968	-2.093528975
HMGN2P32	0.388017722	0.516438086	2.910352372	0.004580441	-2.09525057
RNF130	-0.344261949	2.624494646	-2.910030734	0.004584734	-2.096029501
CTD-2283N19.1	0.279676195	0.277571556	2.909838224	0.004587305	-2.096495682
ANKRD45	0.44496847	0.939365856	2.908877519	0.004600156	-2.098821749
BIN2	-0.379346959	1.168002426	-2.908483777	0.004605432	-2.0997749
MAP2K7	0.425627594	3.608590278	2.908470154	0.004605615	-2.099807875
CXCL10	-1.074061793	2.357375227	-2.908138272	0.004610067	-2.100611197
SLC38A7	-0.305378994	2.451682074	-2.905346481	0.004647674	-2.107365799
RP11-40H20.4	0.338601615	0.13176108	2.905099613	0.004651014	-2.107962834
LGMN	-0.428015326	5.424693544	-2.904909885	0.004653581	-2.108421651
RP11-867O8.5	0.211933291	0.175235234	2.904816893	0.00465484	-2.108646522
AC007679.1	0.427474043	0.22870073	2.904392623	0.004660588	-2.109672414
GAB1	0.373500854	1.999727143	2.904047521	0.004665269	-2.110506788
RP11-324I22.2	0.251301591	0.102085213	2.902367885	0.00468811	-2.114566599
CYP1A2	0.25023028	0.091892479	2.901973201	0.004693492	-2.115520306
VWA8	0.291813928	1.636859298	2.901230257	0.004703639	-2.117315259
ZBED6	0.45246896	1.163077216	2.901116684	0.004705192	-2.117589619
RP11-255P5.2	0.246185903	0.089034852	2.899905741	0.004721778	-2.120514371
RNU6-636P	0.445094379	0.232879552	2.899430287	0.004728305	-2.121662449
FP236383.9	1.378865806	3.784549832	2.899156694	0.004732065	-2.122323026
UBE2V2P3	0.206220604	0.377280089	2.899003219	0.004734176	-2.122693562
RP5-1171I10.5	-0.354942926	0.716253134	-2.89837638	0.004742804	-2.124206778
RPS27P23	0.264692554	0.280794304	2.898002628	0.004747955	-2.125108904
AC090587.2	0.220265672	0.45392268	2.897855309	0.004749987	-2.125464463
RP11-463M14.1	0.527150677	0.177680108	2.897789837	0.00475089	-2.125622478
RP3-391O22.1	0.343282049	0.37076848	2.897689579	0.004752273	-2.125864441
AC004813.1	0.234565855	0.232173224	2.897405931	0.00475619	-2.126548961
AC004076.5	0.248314809	0.51122962	2.8974051	0.004756201	-2.126550968

PLD4	-0.260993939	0.479704332	-2.89740479	0.004756205	-2.126551714
RPL19P14	0.25406734	0.164817856	2.897300449	0.004757647	-2.126803504
DYM	0.276237541	2.910775394	2.89728319	0.004757885	-2.126845152
ZNF385B	0.537938915	0.88498475	2.896626191	0.00476697	-2.128430411
TRBC2	-0.781572077	1.153541304	-2.895871443	0.004777427	-2.130251166
ZBTB37	0.27358179	1.09752079	2.894948022	0.004790249	-2.132478307
RNA5SP477	0.457715101	0.343097462	2.894147758	0.004801387	-2.134407949
VPS28	-0.406040557	4.451945135	-2.893620423	0.004808739	-2.135679254
AC004158.1	0.294733496	0.301929839	2.893154099	0.004815249	-2.136803317
C2	-0.759919053	2.297940348	-2.892602999	0.004822953	-2.138131542
RP11-15G8.1	0.292940812	0.109128848	2.892380835	0.004826062	-2.138666928
MTRF1	0.253191597	1.367573358	2.892159387	0.004829163	-2.139200556
GAL3ST2	0.351004072	0.22501796	2.892009614	0.004831261	-2.13956145
RP11-1058N17.1	0.336307622	0.250428554	2.89196403	0.0048319	-2.139671287
SRP72P1	0.292427386	0.263423769	2.891351887	0.004840486	-2.14114613
RP11-67M9.1	0.267388435	0.114534129	2.89125172	0.004841892	-2.141387439
GAB3	-0.257756471	0.824223103	-2.891217301	0.004842375	-2.141470354
RP11-360A18.1	0.228906317	0.070270566	2.890987303	0.004845606	-2.142024407
PKD1	0.402661382	3.072720184	2.890930608	0.004846402	-2.142160975
RAB6A	0.330103883	4.563739843	2.889723939	0.004863387	-2.145067137
RP11-467I20.6	0.279651659	0.161795028	2.889676641	0.004864053	-2.14518103
BTK	-0.400505735	1.248955412	-2.889556856	0.004865743	-2.145469463
SNX30	0.326029002	2.089692484	2.888807822	0.004876318	-2.147272872
CTIF	0.29173031	2.404251457	2.888728225	0.004877443	-2.147464492
CTC-527H23.2	0.275484419	0.103927558	2.88843125	0.004881642	-2.14817938
ZNF273	0.281960144	0.966085661	2.888259614	0.004884071	-2.148592522
KHSRPP1	0.234541971	0.461957526	2.887665374	0.004892488	-2.150022753
GS1-124K5.3	0.282506637	0.678313486	2.886980709	0.004902202	-2.151670325
RP11-697E2.12	0.208557961	0.147281305	2.88689602	0.004903405	-2.151874098
LINC01053	0.28645501	0.120636762	2.886566779	0.004908084	-2.152666249
MYO19	0.315192934	1.772432479	2.886491138	0.004909159	-2.152848231
SIGLEC9	-0.405068567	1.236558852	-2.885921934	0.00491726	-2.154217532
RARRES3	-0.858273114	2.784735545	-2.88588502	0.004917785	-2.154306324

RP1-21O18.3	0.319061014	0.234434954	2.885697402	0.004920458	-2.154757613
C12orf10	-0.378444375	3.735738167	-2.884383146	0.004939219	-2.157918206
IFFO1	-0.385930974	2.454337106	-2.884154232	0.004942494	-2.158468591
SPOCK2	-0.396578541	1.057629826	-2.883577558	0.004950751	-2.15985495
CCL8	-0.608191431	1.057895887	-2.883446195	0.004952634	-2.160170725
RP11-503I22.2	0.201349821	0.080791644	2.883136814	0.004957071	-2.160914375
AC004862.6	0.365864268	0.614757785	2.882818475	0.004961641	-2.161679492
VPS18	-0.311425525	3.331755671	-2.882302979	0.004969048	-2.162918321
SMC5	0.358500805	2.88756097	2.882206563	0.004970435	-2.163150006
RP11-328P23.3	0.566223934	0.626953253	2.8820003	0.004973402	-2.163645629
METTL7B	-0.631703505	1.178027368	-2.881247596	0.004984245	-2.165454038
HMGN1P5	0.349789175	0.170829026	2.880945865	0.004988598	-2.166178855
FDPSP5	0.272571752	0.381280551	2.880532662	0.004994564	-2.167171349
AC009501.4	0.209019025	0.468738098	2.880372487	0.004996879	-2.167556049
AC078883.4	0.241476436	0.186233896	2.87927636	0.005012745	-2.170188216
AC007567.1	0.432333411	0.151779926	2.879158189	0.005014458	-2.170471935
LGR4	0.644175685	2.984274859	2.878750829	0.005020368	-2.171449904
SLC25A5P6	0.230728593	0.254004008	2.87856567	0.005023057	-2.171894388
DLEU2	0.320551817	1.105367849	2.878169966	0.005028807	-2.172844215
MPP3	0.290443222	1.012632171	2.878067396	0.005030298	-2.173090402
BRWD1	0.305357417	1.881229585	2.877990408	0.005031418	-2.173275182
RICTOR	0.342651085	1.519337653	2.877638331	0.005036541	-2.17412016
SCEL	0.238899459	0.091905006	2.877489191	0.005038713	-2.174478067
SRA1	-0.342418081	3.351110322	-2.876746202	0.005049545	-2.176260873
RNU6-933P	0.528568436	0.220227785	2.876618696	0.005051406	-2.176566788
RP11-174M13.2	0.293614221	0.099672367	2.875868097	0.005062375	-2.178367411
RP11-196H14.2	0.219785167	0.113472076	2.875452403	0.005068459	-2.179364462
C8orf58	-0.288166895	1.803817226	-2.874907523	0.005076444	-2.180671192
SLC25A15	0.398360438	2.077438689	2.874780663	0.005078304	-2.180975398
NOS1AP	0.254269714	0.462084189	2.874758121	0.005078635	-2.181029453
TRPM2	-0.36159309	0.997101672	-2.874316905	0.005085112	-2.182087385
RP11-357J22.1	0.267511712	0.121937322	2.874245564	0.00508616	-2.18225843
RP11-686D22.4	-0.494540588	1.673726456	-2.874146595	0.005087614	-2.182495713

DDX28	-0.318363209	2.525195871	-2.873568662	0.005096113	-2.183881192
RP11-73M18.11	0.276058582	0.218491103	2.873519375	0.005096838	-2.183999338
RP11-140I16.3	-0.339191461	0.573740027	-2.873119983	0.00510272	-2.184956657
SYNGAP1	0.317868993	1.648306746	2.872601708	0.005110362	-2.186198772
CTD-3032H12.1	0.376608988	0.166235584	2.872444642	0.00511268	-2.186575165
RP11-196D18.2	0.213106125	0.10918903	2.872238336	0.005115726	-2.187069532
MED13	0.313597475	2.925108328	2.872178637	0.005116608	-2.187212581
PSMD10P1	0.271157022	0.499078336	2.869884626	0.005150598	-2.19270764
FAM219A	0.401900018	3.194509891	2.869866394	0.005150869	-2.192751297
RP11-845C23.2	0.200136358	0.219577148	2.869714392	0.005153129	-2.193115275
AC017101.10	0.290594526	0.214597016	2.869663375	0.005153887	-2.193237435
AC124276.1	0.309649002	0.153488084	2.8695283	0.005155896	-2.19356086
ATP7A	0.332785063	1.875044928	2.869349487	0.005158557	-2.193988995
RNASET2	-0.386490599	2.115536916	-2.869049107	0.005163029	-2.194708151
SUPT4H1	-0.339813459	4.769288711	-2.868661151	0.005168811	-2.195636886
RNU7-54P	0.361815471	0.130241977	2.867985459	0.005178894	-2.197254197
COLGALT2	0.658477405	2.112863774	2.867925594	0.005179789	-2.197397472
AC025166.1	0.251083227	0.205983389	2.867502096	0.005186119	-2.198410967
NPM1P49	0.301814968	0.175204562	2.866850376	0.005195874	-2.199970391
SAMD4B	0.346281814	4.521124295	2.866849018	0.005195895	-2.19997364
RP11-14N9.2	0.216792001	0.093574928	2.866522484	0.005200789	-2.200754858
NLRP3	-0.246975963	0.62222464	-2.866481705	0.005201401	-2.200852415
CD7	-0.482561755	0.76639913	-2.865379855	0.005217949	-2.203487974
B2M	-0.62479574	8.380727688	-2.865208734	0.005220523	-2.203897212
RP11-324I22.4	0.265137648	0.707015334	2.864286494	0.005234417	-2.206102417
CTC-550M4.1	0.322722045	0.108418717	2.863703389	0.005243219	-2.207496406
RP11-166O4.4	0.36426624	0.376753966	2.863330662	0.005248853	-2.208387338
FTX	0.211960886	0.640274454	2.863043153	0.005253203	-2.209074509
DLGAP1-AS4	0.216646203	0.089141482	2.862989197	0.005254019	-2.209203462
RNU6-1310P	0.459467972	0.238008161	2.862896481	0.005255423	-2.209425044
GMPS	0.313036909	3.350907739	2.862313761	0.005264252	-2.210817563
CTB-35F21.1	0.240163447	0.288600825	2.860959895	0.005284817	-2.214051996
FARP2	0.358026254	2.026638261	2.860809431	0.005287107	-2.214411382

RP11-727A23.4	0.382688159	0.65913978	2.860776786	0.005287604	-2.214489352
MSMO1	0.551439664	3.845036986	2.860292408	0.005294983	-2.215646187
TAT	0.268648287	0.114808684	2.860057765	0.005298562	-2.216206524
OSTCP1	0.286563064	0.270313301	2.85975611	0.005303165	-2.216926833
HACE1	0.2621121	1.116680966	2.859589239	0.005305713	-2.217325272
MARK2	0.289973023	3.256650613	2.859177113	0.00531201	-2.218309224
CTD-2182N23.1	0.291515202	0.285385248	2.858589662	0.005320999	-2.219711568
PLEKHM3	0.252302536	0.802243748	2.858536885	0.005321807	-2.219837543
SDSL	-0.487057641	2.265562417	-2.857779332	0.005333421	-2.221645575
IGFL2	0.226808253	0.225567764	2.857712007	0.005334454	-2.221806239
MMP11	-0.843279452	3.38431194	-2.857507183	0.005337599	-2.222295012
ESCO2	0.318709465	1.152363576	2.85740824	0.005339119	-2.222531109
RP11-361M4.1	0.318372343	0.13979878	2.857200805	0.005342306	-2.223026069
LINC01558	0.236807952	0.267750043	2.856511467	0.00535291	-2.224670685
AC116050.1	0.230911835	0.118894922	2.856373095	0.005355041	-2.225000773
RNU7-153P	0.407392103	0.128095374	2.8563673	0.005355131	-2.225014597
LTN1	0.423959572	2.259959939	2.855846774	0.005363154	-2.2262562
SFXN2	0.302437731	0.940130785	2.855740999	0.005364786	-2.226508479
CORO7	-0.262943697	1.16996983	-2.855254287	0.0053723	-2.227669224
RP11-415J8.3	0.266280109	0.730983231	2.855146989	0.005373958	-2.227925095
RNU6-121P	0.462896643	0.189342573	2.854929685	0.005377317	-2.228443269
RNU4ATAC11P	0.28159422	0.317123036	2.854675212	0.005381253	-2.229050032
RP11-586K12.11	0.266601441	0.088822165	2.85451946	0.005383663	-2.229421387
NCAPG2	0.467053866	3.036062253	2.854446405	0.005384794	-2.229595564
RINT1	0.296272134	2.812133236	2.853839687	0.005394194	-2.231041947
RP11-6B19.3	0.253157786	0.092686423	2.853669063	0.005396841	-2.231448662
RP11-295B17.6	0.41246932	0.232505492	2.853507781	0.005399343	-2.23183309
RNU6-583P	0.39011717	0.501551507	2.853206804	0.005404016	-2.232550444
DAGLB	-0.315595924	2.568614936	-2.852611196	0.005413275	-2.233969843
BLZF1	0.352431319	2.863107769	2.851974556	0.005423187	-2.235486762
NGEF	0.83562901	2.052893573	2.851616665	0.005428767	-2.236339386
SLC5A11	0.24454897	0.162749695	2.851434103	0.005431615	-2.23677428
RP1-267L14.3	0.208275914	0.225388473	2.850853771	0.005440678	-2.238156582

RP5-1021I20.7	0.382614148	0.436794157	2.850751098	0.005442283	-2.238401116
NDUFA5P6	0.340250613	0.178448529	2.850240782	0.005450267	-2.23961642
RP11-823P9.4	0.418720191	0.272944928	2.849103551	0.005468096	-2.24232407
SMG7-AS1	0.211857502	0.243432638	2.848754908	0.005473573	-2.243153982
CSTA	-0.436479846	1.158241809	-2.848538427	0.005476976	-2.243669254
LINC00904	0.259482509	0.099949595	2.847354741	0.005495618	-2.246486121
AC005808.3	0.25296442	0.1042943	2.847227298	0.005497629	-2.246789344
GS1-278J22.1	0.290949856	0.108726837	2.845976837	0.005517393	-2.249763976
GNA14-AS1	0.208463906	0.170275367	2.845722116	0.005521427	-2.250369782
SPCS2	0.327131724	4.476577919	2.845408575	0.005526396	-2.251115423
RNA5SP384	0.469511352	0.220400116	2.845327096	0.005527688	-2.251309177
CTC-431G16.2	0.214181934	0.126701207	2.844995621	0.005532947	-2.252097375
SYCP2	0.474770393	1.034655907	2.844954831	0.005533594	-2.252194362
CDON	0.459361384	1.395714468	2.844546401	0.005540081	-2.253165433
AC134300.1	0.308054116	0.112274	2.843935728	0.005549793	-2.254617138
RN7SL547P	0.344909475	0.146053653	2.843318887	0.005559619	-2.256083247
CTD-2531D15.4	0.202392037	0.208365665	2.842471449	0.005573145	-2.258097021
SNAI3	-0.275670475	0.878669906	-2.841555355	0.0055878	-2.260273389
AC006160.5	0.308466225	0.426271484	2.841501724	0.005588659	-2.260400783
TIAM2	0.441941383	1.925206517	2.841350899	0.005591076	-2.260759039
RASGRP4	-0.321607138	0.856394785	-2.841350791	0.005591077	-2.260759295
RP11-91N2.3	0.221025043	0.135461105	2.841063054	0.00559569	-2.261442716
CCDC36	0.411323116	0.777262148	2.840909082	0.00559816	-2.2618084
LINC01272	-0.358794203	0.7324679	-2.840871358	0.005598765	-2.261897992
RP11-139G7.1	0.237134471	0.097307184	2.840623935	0.005602737	-2.262485584
DZIP1L	0.320012816	1.098094777	2.840303868	0.005607878	-2.263245631
TMEM229A	0.298701621	0.185660332	2.840208083	0.005609417	-2.263473074
CTA-313A17.3	0.241775774	0.243300742	2.839945163	0.005613645	-2.264097347
ARPC4	-0.39881921	4.839837783	-2.839034459	0.005628311	-2.266259349
RP11-267N12.2	0.22277114	0.108812634	2.838882248	0.005630765	-2.266620641
RP11-487I5.4	0.249143186	0.157300012	2.838256564	0.005640866	-2.268105616
RP11-831F12.3	0.228213584	0.361039227	2.838036688	0.005644419	-2.268627397
RNA5SP222	0.368799017	0.133883187	2.837775067	0.00564865	-2.2692482

CCDC26	0.38539184	0.218992026	2.837687725	0.005650063	-2.269455443
RP3-510O8.3	0.374729788	0.215718755	2.836804202	0.005664375	-2.271551568
RP1-184J9.2	0.227036281	0.155557376	2.836781311	0.005664746	-2.271605868
RNU6-1077P	0.402863988	0.169941303	2.836771586	0.005664904	-2.271628937
RP11-252E2.2	0.229359298	0.121345834	2.836220954	0.005673842	-2.272935009
AC084357.2	0.388764282	0.172423344	2.835936303	0.005678467	-2.273610105
RENBP	-0.707942242	2.747562653	-2.835645503	0.005683196	-2.274299728
IDE	0.374834876	2.270622877	2.83548205	0.005685855	-2.274687327
MPP4	0.261176655	0.24411254	2.83493735	0.005694726	-2.27597885
RNU2-3P	0.233605635	0.102902759	2.834721256	0.005698249	-2.276491169
PSMD13	-0.341685559	4.364206543	-2.834672491	0.005699044	-2.276606777
RP11-24F11.2	-0.216783205	0.398914267	-2.834046938	0.005709255	-2.278089646
CYP2T3P	0.239663022	0.153836955	2.833125088	0.005724332	-2.280274398
THEMIS2	-0.525647656	2.564445779	-2.832439335	0.005735572	-2.281899234
NLRP7	0.209973289	0.100133971	2.83202258	0.005742412	-2.282886543
KCNQ5-AS1	0.248024138	0.131157807	2.831780094	0.005746396	-2.283460948
SRRM2	0.339122044	4.85758781	2.831267326	0.005754828	-2.28467547
PISD	-0.288820045	2.239418549	-2.830304761	0.005770688	-2.286954878
AC064834.3	0.281600767	0.129606696	2.829124692	0.005790185	-2.289748485
SPARCL1	-0.623419517	3.48551826	-2.828249045	0.005804692	-2.291820811
KCND2	0.220013151	0.149081879	2.827671982	0.005814271	-2.293186217
AC069257.8	0.206436377	0.323775038	2.827359277	0.005819467	-2.293926023
SIK2	0.344443839	2.490605908	2.82690574	0.005827012	-2.294998892
RNU6-436P	0.337070618	0.217294321	2.826891491	0.005827249	-2.295032598
DIS3	0.358481341	2.350047068	2.826415756	0.005835173	-2.296157823
HMGN2P28	0.227266772	0.507362013	2.82566723	0.00584766	-2.29792795
AC008982.2	0.357733188	0.889277787	2.824374702	0.00586928	-2.300983642
RP11-70D24.2	-0.284799758	0.620692703	-2.824330058	0.005870028	-2.301089166
RP11-425M5.8	0.276038531	0.119657004	2.823518142	0.005883648	-2.303008025
PFDN5	-0.478955082	5.512892071	-2.823168283	0.005889526	-2.303834733
GOLGA2	0.358016515	3.928032337	2.823006533	0.005892245	-2.304216917
DCUN1D2-AS	0.340650879	0.530794296	2.822634173	0.00589851	-2.30509666
CTD-2306M5.1	0.220952856	0.09307486	2.822271744	0.005904613	-2.30595285

RP11-503E24.2	0.343371459	0.689248619	2.821580289	0.005916273	-2.307586072
USP12-AS2	0.341950674	0.44568708	2.81969936	0.005948098	-2.312027184
REEP6	-0.415514472	1.260252871	-2.819020889	0.005959616	-2.313628547
RNU7-57P	0.215771379	0.069574741	2.819019286	0.005959643	-2.31363233
RP11-118M9.3	0.225741874	0.299647788	2.818939427	0.005961	-2.313820797
CTC-430J12.2	0.208275893	0.099169733	2.818850502	0.005962512	-2.314030653
UBE2V1P4	0.277912004	0.111633849	2.818408638	0.005970028	-2.315073344
KDM6A	0.364145976	2.361403753	2.818339167	0.00597121	-2.315237266
AC011233.2	0.268131222	0.084805857	2.818338059	0.005971229	-2.31523988
RP11-110I1.11	0.488554861	1.553330628	2.816566896	0.006001447	-2.319417961
CTD-2312P21.1	0.224979787	0.217467	2.816561943	0.006001532	-2.319429641
ASPM	0.460389408	2.481930703	2.816185797	0.006007968	-2.320316673
QDPR	-0.419480116	3.22950234	-2.815757663	0.006015301	-2.321326185
RP11-1090M7.3	0.392991475	0.429489699	2.815690533	0.006016451	-2.321484461
NLRC4	-0.224557622	0.784679324	-2.815429398	0.006020928	-2.322100126
ATP6V0A4	0.200671251	0.108244061	2.815136034	0.006025962	-2.322791721
RNU6-996P	0.256349409	0.102481796	2.81512674	0.006026122	-2.322813631
STXBP1	0.440280253	3.135794712	2.814661123	0.006034119	-2.323911182
RPL10AP1	0.324305967	0.66615693	2.814150817	0.006042895	-2.325113904
RP5-1057J7.7	0.432800571	1.019513961	2.813708032	0.006050519	-2.326157342
RPS6KA5	0.228764212	0.450406166	2.813650366	0.006051513	-2.326293225
RNA5SP227	0.476868752	0.310190938	2.811641951	0.006086212	-2.331024362
NDUFA9	-0.403371866	2.345008024	-2.811431673	0.006089855	-2.331519545
COG6	0.307537681	1.865976724	2.811343438	0.006091385	-2.33172732
RP11-458I7.1	0.335241236	0.41946415	2.81064087	0.006103575	-2.333381535
NPM1P26	0.21797651	0.452723689	2.810582658	0.006104586	-2.333518582
AC018867.1	0.399288273	0.354276293	2.809968416	0.006115264	-2.334964528
KIAA0930	-0.424263713	3.456685508	-2.809383143	0.006125454	-2.33634204
MIR4530	0.278090067	0.288705848	2.808097153	0.0061479	-2.339367951
ITGAL	-0.410862646	0.929827396	-2.807804255	0.006153022	-2.340056974
CD8A	-0.566536526	0.715062267	-2.807748516	0.006153998	-2.34018809
MIR5584	0.207764416	0.081543905	2.807437655	0.006159439	-2.340919297
APOL2	-0.482099592	3.907012045	-2.8073982	0.00616013	-2.341012099

RP11-177A2.5	0.213667675	0.218000673	2.807382629	0.006160403	-2.341048722
PKLR	0.272309262	0.191406988	2.807322329	0.006161459	-2.34119055
RN7SL376P	0.416707823	0.26544539	2.807041694	0.006166377	-2.341850579
IFNGR1	-0.351151691	4.225060279	-2.806881718	0.006169183	-2.342226805
SMAD4	0.293370632	2.69052458	2.806879103	0.006169228	-2.342232955
RAD1	0.28468011	2.378583407	2.8067953	0.006170698	-2.342430032
MGMT	-0.506233554	2.35435789	-2.806739968	0.006171669	-2.342560152
RP11-554A11.9	0.234397606	0.199846657	2.806118976	0.006182573	-2.344020352
RAD51AP1P1	0.225258922	0.119943458	2.806065122	0.006183519	-2.34414697
MTND4P20	0.206866791	0.074029997	2.805917187	0.00618612	-2.344494782
RP11-876N24.3	-0.24577639	0.758572915	-2.805534779	0.006192847	-2.345393789
RPS6P8	0.287058315	0.229241177	2.80545448	0.00619426	-2.345582553
RP11-745A24.3	0.263069392	0.112355175	2.804352706	0.006213683	-2.348172108
CSGALNACT2	0.443877847	3.484659839	2.804080488	0.006218491	-2.348811787
RP11-393K12.4	0.453164324	0.185120766	2.804005252	0.00621982	-2.348988575
HECTD4	0.282632668	1.687345782	2.803782698	0.006223754	-2.349511502
AP001429.1	0.218935559	0.40978398	2.802098352	0.006253599	-2.353468037
MYL6	-0.43805652	6.679007403	-2.801241592	0.00626883	-2.355479824
LINC01151	0.561083519	0.225693973	2.800942198	0.006274161	-2.35618272
CHKB	-0.251231047	0.786497304	-2.800855865	0.006275699	-2.356385396
HMGB1P8	0.294006897	0.365976696	2.800177506	0.006287794	-2.357977737
HIGD2B	0.220961824	0.118709882	2.799558169	0.006298856	-2.359431255
AL355098.1	0.509817959	0.211353741	2.799438599	0.006300994	-2.359711844
RBX1	-0.383459845	4.271419454	-2.798894332	0.006310733	-2.36098892
RP1-40E16.2	0.2460278	0.158479774	2.798788018	0.006312637	-2.36123835
CARD16	-0.593985841	1.734636612	-2.798107083	0.006324844	-2.36283577
RN7SL269P	0.258760751	0.500103929	2.798041172	0.006326027	-2.362990375
RP11-310E22.4	0.220064851	0.242694904	2.797992367	0.006326903	-2.363104854
AP000345.1	0.207638966	0.092682037	2.797301405	0.006339316	-2.364725416
RP11-627G18.4	0.34390393	0.138112654	2.797115109	0.006342666	-2.365162292
HMGN1P34	0.305343521	0.14143168	2.797093782	0.00634305	-2.365212303
RP11-412H8.2	0.220438852	0.083589284	2.796205004	0.006359058	-2.367296208
AL138935.1	0.265840466	0.096881202	2.796195802	0.006359224	-2.367317781

RPL21P1	0.39629354	0.694907606	2.795153019	0.006378053	-2.369762078
RNU6-741P	0.278274275	0.132217451	2.795119451	0.00637866	-2.369840751
RP11-63A23.2	0.318928575	0.125491931	2.794819422	0.006384088	-2.370543877
AMZ2P2	0.235654526	0.116764832	2.794220999	0.006394926	-2.371946115
XRCC2	0.361073616	1.77061234	2.794060114	0.006397842	-2.372323061
OGFRL1	-0.384406766	2.133366353	-2.794038198	0.00639824	-2.37237441
RP3-380B8.4	0.396923462	0.169246912	2.793859722	0.006401477	-2.372792548
RP5-1077B9.5	0.391717112	0.217208407	2.79372609	0.006403902	-2.37310561
RP11-708H21.1	0.226894214	0.135377705	2.793707867	0.006404232	-2.373148301
RP11-10G12.2	0.273819777	0.14755234	2.792477259	0.006426602	-2.37603068
RN7SL371P	0.417758475	0.15795426	2.792420571	0.006427635	-2.37616343
RP13-270P17.2	0.201255132	0.594216891	2.792254573	0.006430658	-2.376552151
RN7SL847P	0.229437395	0.114723199	2.792215744	0.006431366	-2.376643076
RP11-62C3.6	0.332354792	0.22816279	2.791245229	0.00644907	-2.378915345
CLIC2	-0.439829286	1.326302719	-2.791144553	0.006450909	-2.379151021
RP5-867C24.5	0.26569915	0.344002809	2.790803665	0.00645714	-2.379948966
RNU6-906P	0.318936783	0.130349824	2.790520369	0.006462322	-2.380612039
RAD23A	0.419203818	5.366079115	2.790383356	0.00646483	-2.380932708
CTD-2356P16.6	0.254860619	0.095736737	2.790311584	0.006466144	-2.381100681
SIT1	-0.341519335	0.500661504	-2.789873947	0.006474162	-2.382124829
CATSPERD	0.233850737	0.128992197	2.789832711	0.006474918	-2.38222132
PTAR1	0.391947363	2.317674166	2.789692974	0.00647748	-2.382548297
ZNF609	0.294164304	2.412422152	2.789348745	0.006483795	-2.383353719
RP11-216L13.21	0.213766371	0.33311821	2.788247449	0.006504039	-2.385929966
RP11-1C8.5	0.210389361	0.116142969	2.788109398	0.006506581	-2.386252846
RP11-469J4.3	0.241741845	0.217944843	2.787757395	0.006513065	-2.387076072
ABHD14A	-0.36800178	2.401729587	-2.786813491	0.006530484	-2.38928315
CLEC10A	-0.295611778	0.520853247	-2.786479093	0.006536665	-2.390064906
TPT1P1	0.356877871	0.669508257	2.785907709	0.006547239	-2.391400513
PPP6R3	0.27407119	3.296802201	2.78548832	0.00655501	-2.392380687
LINC00603	0.224825461	0.104369355	2.785356445	0.006557455	-2.392688875
RNU6-180P	0.332728765	0.117203867	2.78442395	0.00657477	-2.394867738
PDS5B	0.271190947	1.937165254	2.783457706	0.006592756	-2.397124825

RNU6-1340P	0.211673882	0.138809882	2.783353754	0.006594694	-2.397367615
ARFGAP2	-0.310894753	3.637500526	-2.783169039	0.006598138	-2.39779901
RP11-454H13.1	0.237346909	0.258587352	2.782749018	0.006605976	-2.398779871
RP11-439E19.7	0.22896059	0.139586081	2.782667067	0.006607507	-2.398971233
RP11-358M3.1	0.318066669	0.148769116	2.782573149	0.006609261	-2.399190535
RP11-541P9.3	0.21702392	0.105448987	2.782131449	0.006617517	-2.400221837
RP11-678K21.2	0.201866575	0.080079838	2.781538236	0.006628619	-2.401606685
SOX2OT_exon4	0.327286824	0.098992208	2.781529461	0.006628783	-2.40162717
MIR128-1	0.337740574	0.158408208	2.781328903	0.006632541	-2.402095315
HCG20	0.200575338	0.138098321	2.780547957	0.006647191	-2.40391794
AC005540.3	0.246687005	0.411617646	2.780339259	0.006651111	-2.404404945
FAM160A1	0.289375389	0.69082263	2.780052646	0.006656498	-2.405073717
ATR	0.255657066	1.530568412	2.779510527	0.006666698	-2.40633852
BNIP3	0.681229468	3.645718016	2.778617402	0.006683533	-2.408421804
IFNL3P1	0.265024519	0.099744283	2.778609275	0.006683687	-2.408440758
RP5-837I24.6	0.204693072	0.10710948	2.778479366	0.006686139	-2.408743735
ECT2	0.368929825	2.580574377	2.778385	0.00668792	-2.408963809
CTD-2010I22.2	0.255647479	0.118118587	2.77802522	0.006694717	-2.409802812
CCDC186	0.2929593	1.405752768	2.777967372	0.006695811	-2.409937703
MIR3926-2	0.241181222	0.165238414	2.776840011	0.006717153	-2.412566073
CCDC124	-0.397924694	5.03472601	-2.776351761	0.006726415	-2.413704122
RP11-404L6.2	0.233104467	0.09298611	2.776233531	0.00672866	-2.413979677
CTD-2005H7.1	0.225576513	0.166782121	2.775793208	0.006737026	-2.415005838
RWDD3	-0.313966974	2.073558732	-2.775625082	0.006740223	-2.415397618
CTD-2192J16.21	0.380513118	0.490865735	2.775520342	0.006742215	-2.41564168
RN7SL223P	0.215097023	0.081455248	2.775108737	0.006750049	-2.416600714
CYTH4	-0.610291109	2.277569948	-2.775098096	0.006750252	-2.416625508
RP11-115D7.3	0.334348989	0.537973813	2.775070474	0.006750778	-2.41668986
DRD2	0.289285691	0.358578115	2.775024996	0.006751644	-2.416795818
CTD-2377D24.4	0.220146472	0.153544378	2.774907161	0.006753889	-2.417070342
DPRXP2	0.271106208	0.182007754	2.774770568	0.006756492	-2.417388559
RN7SKP33	0.202333938	0.067420859	2.774645692	0.006758872	-2.417679467
RBP7	-0.545815744	1.85430263	-2.774644423	0.006758897	-2.417682423

AL355490.1	0.304274112	0.26974174	2.774608519	0.006759581	-2.417766062
RP11-428P16.3	0.233903615	0.104837094	2.774271044	0.006766019	-2.418552171
RNU6-179P	0.352946402	0.216471341	2.774255913	0.006766308	-2.418587415
RP11-344N10.2	0.335136259	0.393832663	2.77416432	0.006768056	-2.418800758
MYCBP2	0.370624957	2.181291628	2.774009415	0.006771014	-2.419161554
CTD-2568A17.1	0.326977861	0.381141624	2.773981438	0.006771548	-2.419226715
EIF4G1	0.260635165	5.738826453	2.772355348	0.00680267	-2.423013082
RPS26P5	0.45773654	0.184059163	2.772134421	0.006806909	-2.423527372
RPL21P23	0.240928219	0.142432484	2.771547199	0.006818186	-2.424894187
CACYBPP1	0.238933359	0.229618469	2.770993536	0.006828834	-2.42618267
FAM72D	0.218476237	0.765863388	2.770622862	0.006835972	-2.427045185
RP13-20L14.10	0.262841093	0.604529073	2.770063317	0.00684676	-2.428346998
CYCSP11	0.264277243	0.12416634	2.769324678	0.006861024	-2.430065155
AC018643.4	0.290076799	0.114643813	2.769155989	0.006864285	-2.430457492
TCF7L1	-0.527019672	2.819074022	-2.7689252	0.006868749	-2.430994227
RP11-496B10.3	0.388028676	0.240057039	2.768873417	0.006869751	-2.431114653
DPRXP5	0.307495862	0.150724609	2.76883069	0.006870578	-2.431214015
RP11-14I17.2	0.317058748	0.313446395	2.768542619	0.006876156	-2.431883899
TMEM14C	-0.345741663	5.640482386	-2.768329597	0.006880283	-2.432379227
TRAV15	0.224648476	0.104488066	2.767084766	0.006904445	-2.435273133
S100A4	-0.763534504	7.599231741	-2.767013986	0.006905821	-2.435437645
RN7SKP269	0.31369203	0.518865284	2.766928784	0.006907478	-2.435635674
AGAP1	0.334026737	2.063729821	2.766831614	0.006909368	-2.435861514
AP000275.64	0.266971201	0.119631536	2.765983316	0.006925889	-2.437832822
RAPH1	0.263769626	0.673568697	2.765402648	0.006937219	-2.439181914
PCA3_1	0.204658952	0.200827862	2.765267109	0.006939865	-2.439496783
RP11-357H14.16	0.332035545	0.253516306	2.764236204	0.006960028	-2.44189126
SLC12A8	0.466190347	1.506393179	2.763756154	0.006969435	-2.443006019
PIAS2	0.269279175	1.389738111	2.763492729	0.006974603	-2.443617669
RP11-356M20.1	0.201829565	0.121335666	2.763398117	0.006976459	-2.443837339
RNU6-223P	0.647140641	0.246183722	2.763337065	0.006977658	-2.443979085
RPL21P24	0.302842449	0.135180329	2.763179823	0.006980745	-2.444344147
CTC-508F8.1	0.445111467	0.502320472	2.763005799	0.006984163	-2.444748151

RP11-278C7.5	-0.28737173	0.731659197	-2.762411474	0.006995848	-2.446127747
CTC-459M5.1	0.236398789	0.135838187	2.762285274	0.006998332	-2.446420662
CTD-2139B15.5	0.312636269	0.160802076	2.762104326	0.007001894	-2.446840628
MIR3147	0.25266856	0.150570283	2.761995749	0.007004033	-2.447092616
RP11-122F24.1	0.222630669	0.125797525	2.761256611	0.007018605	-2.448807809
AC092675.3	0.26369535	0.170896753	2.761093676	0.007021822	-2.449185853
CTD-2329K10.1	0.41015164	0.692455004	2.760575911	0.007032051	-2.450387061
KAT6A	0.362406762	2.462080474	2.760070933	0.00704204	-2.451558421
RP11-274B21.14	0.352390849	1.201249335	2.76004655	0.007042523	-2.451614976
AC027612.1	0.305065241	0.502283594	2.759944638	0.007044541	-2.451851352
CENPF	0.448905778	3.036929616	2.759749339	0.007048409	-2.452304308
MSNP1	0.330279956	1.168165136	2.759346829	0.007056387	-2.453237766
RP11-166J22.1	0.2470838	0.134781952	2.758506884	0.007073063	-2.455185316
TNFRSF14	-0.48962118	2.658467194	-2.757151976	0.007100038	-2.458325858
RP11-426L16.3	0.285293762	0.383213413	2.7569615	0.007103838	-2.458767261
RP11-159F24.3	0.228141967	0.603329462	2.756647838	0.007110099	-2.459494078
RP3-499B10.3	0.278023819	0.121503134	2.756073083	0.007121585	-2.460825719
RP11-466I1.1	0.217336577	0.088640276	2.755885785	0.007125332	-2.461259615
CTC-535M15.1	0.342438479	0.221482291	2.755782497	0.007127398	-2.461498884
AC093270.1	0.27966904	0.102215766	2.755746	0.007128129	-2.461583428
RP11-63E16.1	0.300783604	0.289266619	2.754962355	0.00714383	-2.463398499
RGS22	0.358971492	0.567065209	2.754124363	0.007160655	-2.465338973
NPM1P43	0.219165242	0.144213644	2.753980369	0.00716355	-2.465672359
GAS2L3	0.340189931	2.116305248	2.75207125	0.00720203	-2.470091154
AL049871.1	0.265965834	0.191733785	2.752046861	0.007202523	-2.470147588
ZDHHC20P3	0.255179328	0.097753234	2.751981789	0.007203838	-2.470298158
RN7SL843P	0.222988575	0.106506866	2.751802892	0.007207454	-2.470712086
C5	0.271697023	0.817126418	2.751514778	0.007213281	-2.471378675
ACER2	0.214112109	0.510245946	2.751473727	0.007214112	-2.471473647
ZNF221	0.293450539	1.10062389	2.751322718	0.007217169	-2.471822997
MIR1254-1	0.591142872	1.672917713	2.751322621	0.007217171	-2.471823222
RP11-1127D7.1	0.200229216	0.083123634	2.750599265	0.007231828	-2.473496446
AC022616.1	0.278850198	0.089002461	2.75052291	0.007233376	-2.473673046

BRCA2	0.234683863	1.007586176	2.749781622	0.007248428	-2.475387333
RP4-785G19.2	0.271436165	0.688209423	2.749712907	0.007249825	-2.475546222
RP11-383C6.2	0.318295906	0.222810603	2.749495824	0.007254239	-2.476048161
A2ML1-AS2	0.305493666	0.133526122	2.749390586	0.00725638	-2.476291482
RIF1	0.327955669	2.226400192	2.749282431	0.00725858	-2.476541537
RNU6ATAC29P	0.396742349	0.202754305	2.748123934	0.007282191	-2.479219488
AL121933.1	0.382161911	0.156433759	2.747715975	0.007290522	-2.480162296
MON2	0.254071979	2.026306939	2.747455399	0.007295847	-2.480764433
RNU7-48P	0.530155911	0.333561403	2.746860462	0.00730802	-2.482139037
CEACAM21	-0.227659519	0.48761943	-2.746758826	0.007310102	-2.482373841
VN1R10P	0.217928411	0.095038464	2.746616557	0.007313016	-2.482702508
RNA5SP26	0.30151592	0.109407013	2.746439597	0.007316643	-2.483111298
AC078899.1	0.295032885	1.074176233	2.746426846	0.007316904	-2.483140752
DEFB118	0.207293694	0.081396678	2.746059895	0.00732443	-2.48398836
SPOPL	0.298483355	1.958079606	2.746044562	0.007324745	-2.484023775
PSENEN	-0.396701825	4.0413378	-2.746014213	0.007325367	-2.484093874
RPL32P33	0.27991451	0.337977777	2.745581628	0.007334249	-2.485092954
AC104841.1	0.557430838	1.08652042	2.745564146	0.007334608	-2.485133325
RNU6-1156P	0.250724797	0.153238987	2.745498795	0.007335951	-2.485284246
LINC01359	0.214179999	0.180321758	2.745243991	0.007341189	-2.485872656
RP11-419I17.1	0.218009059	0.235873446	2.745154535	0.007343028	-2.486079221
CTD-2296D1.1	0.219036858	0.07226299	2.744545148	0.007355571	-2.487486234
RNU6-556P	0.334898781	0.157463981	2.744317885	0.007360253	-2.488010895
RP11-213G2.5	0.218037215	0.166086494	2.744247562	0.007361703	-2.488173236
APOL1	-0.854990007	3.431655102	-2.744217382	0.007362325	-2.488242907
RP4-583P15.16	0.270090672	0.45886295	2.744210745	0.007362462	-2.488258227
FAM111B	0.556709613	2.358141837	2.744101773	0.007364708	-2.488509779
CTC-774J1.1	0.248665336	0.104047573	2.743865802	0.007369576	-2.489054472
ISCA2	-0.252942027	2.099793634	-2.743534493	0.007376415	-2.489819167
MBNL2	0.418386691	3.079636525	2.743512945	0.00737686	-2.489868899
PIWIL4	0.248955781	0.632195966	2.742075814	0.007406595	-2.493185037
MEX3C	0.264047823	2.409455457	2.742037913	0.00740738	-2.493272472
RNU6-603P	0.321978315	0.137194872	2.741979987	0.007408581	-2.493406104

RN7SL426P	0.404155308	0.302347466	2.741751739	0.007413315	-2.493932631
PFN1	-0.385188348	8.43650457	-2.741441081	0.007419762	-2.494649208
NUTF2	-0.356904331	4.555919746	-2.739567302	0.007458759	-2.498969917
TNIK	0.390877038	1.89810083	2.739413648	0.007461965	-2.499324115
RP11-177N22.3	0.267100156	0.177307073	2.73750055	0.007501989	-2.503732765
TBXAS1	-0.467096075	1.829191688	-2.736831855	0.007516024	-2.505273143
TIPARP	0.405590152	2.87172957	2.736641408	0.007520026	-2.505711793
ZNF236	0.251051436	1.276881555	2.735889477	0.007535845	-2.507443437
RP11-393N4.1	0.279022837	0.107823739	2.735494659	0.007544164	-2.508352519
MYO3B	0.313014106	0.347618083	2.734432561	0.007566582	-2.510797495
RNU6-709P	0.220637994	0.068539594	2.734288477	0.007569628	-2.511129119
SMAD6	0.704268666	3.186536183	2.733366548	0.007589145	-2.513250695
NDUFA3P1	0.284827036	0.341158869	2.732803928	0.007601077	-2.514545126
RP11-545M17.2	0.249510158	0.44625359	2.73245358	0.007608516	-2.515351065
FAM222A-AS1	0.317044243	0.405649635	2.731731932	0.007623861	-2.517010875
RP11-1018N14.2	0.304827552	0.116018621	2.730969528	0.007640102	-2.518764031
RP11-357N13.1	0.303633127	0.444017889	2.730891769	0.00764176	-2.518942816
HDAC4	0.29353681	1.439937054	2.730867961	0.007642268	-2.518997554
SDCCAG3P1	0.215872659	0.152150817	2.730641156	0.007647107	-2.519519
RNA5SP207	0.420317188	0.257419233	2.730570299	0.007648619	-2.5196819
RNU4-6P	0.392068595	0.166436057	2.73013268	0.007657966	-2.520687905
DZIP1	0.434875835	2.353353363	2.729313421	0.007675491	-2.522570872
RP1-166H1.2	0.287509252	0.104097523	2.729113872	0.007679765	-2.523029439
ANKRD36C	0.220330817	0.528921215	2.729019596	0.007681785	-2.523246077
RNU6-502P	0.718034744	0.291766715	2.728987338	0.007682476	-2.523320201
MRPL49P1	0.310181513	0.108084162	2.728708984	0.007688444	-2.523959795
LINC00943	0.206200575	0.183013165	2.728597926	0.007690826	-2.524214964
CTSL	-0.538380723	5.383141326	-2.728101356	0.007701486	-2.525355794
NSA2	-0.38528968	3.723737908	-2.727972536	0.007704253	-2.52565172
AC002306.1	0.226914597	0.192425149	2.727928554	0.007705198	-2.525752753
RNU6-155P	0.304360112	0.169969189	2.727790846	0.007708158	-2.526069079
RNU11-3P	0.30004531	0.28932736	2.727505462	0.007714295	-2.526724586
USP31	0.227443107	1.412037958	2.727407256	0.007716408	-2.526950145

RP11-1094M14.14	0.338668449	0.16801955	2.726115372	0.007744252	-2.529916713
RP11-295K2.3	0.344876788	0.325751121	2.72608915	0.007744818	-2.529976917
CTD-2200P10.3	0.256187332	0.10241652	2.725913775	0.007748605	-2.530379538
IL2RA	-0.312163488	0.53673658	-2.725782146	0.007751449	-2.530681716
RP11-15M15.2	0.27491779	0.20160585	2.725326914	0.007761291	-2.531726688
AC091178.2	0.331145376	0.197545264	2.725029879	0.007767718	-2.532408446
MIR515-2	0.226100226	0.070121176	2.724955622	0.007769326	-2.53257887
RP11-589N15.1	0.25135252	0.146827797	2.724585219	0.00777735	-2.533428918
AF001548.5	-0.430949701	1.499830086	-2.724227755	0.007785101	-2.53424918
AC091487.1	0.303365495	0.106475708	2.724111355	0.007787626	-2.534516261
RB1CC1	0.42444586	3.368442821	2.7234116	0.007802823	-2.536121651
RP11-298J20.4	0.475805691	1.300606122	2.723175813	0.00780795	-2.536662522
AC073316.2	0.340246536	0.137290119	2.723056749	0.00781054	-2.536935627
AL645937.1	0.221291956	0.065381714	2.722163232	0.007830002	-2.538984833
ARL6IP5	-0.366588342	5.66481831	-2.721650642	0.007841187	-2.540160165
RP11-55L4.1	0.261695993	0.153849947	2.721610173	0.007842071	-2.54025295
NUGGC	0.22199446	0.212324401	2.721401585	0.007846627	-2.540731168
RPL21P106	0.232233166	0.212586362	2.720840563	0.007858893	-2.542017243
AC018512.1	0.711967153	0.836082161	2.719815933	0.007881341	-2.544365514
DLG1-AS1	0.23706676	0.099394115	2.719700607	0.007883871	-2.544629775
RNA5SP443	0.271156197	0.133340295	2.719179137	0.007895321	-2.54582457
AC151960.1	0.22922811	0.097574489	2.718619865	0.007907618	-2.547105766
SCMH1	0.295375692	3.320300826	2.717505957	0.007932162	-2.549656885
RP11-284F21.7	0.3199835	0.383537181	2.717486528	0.007932591	-2.549701374
RP11-6C14.1	0.394805559	0.204510307	2.716844205	0.007946776	-2.551172046
RP4-744I24.4	0.354413634	0.40946126	2.71670628	0.007949825	-2.551487804
CYCSP43	0.234646652	0.086361386	2.716444685	0.007955611	-2.55208665
CD48	-0.459278072	1.153551333	-2.715751557	0.00797096	-2.553673129
RP11-344N10.4	0.272875796	0.347472715	2.714393624	0.008001108	-2.556780289
SNORD116	0.269075187	0.084678841	2.713771588	0.008014953	-2.558203171
HIST3H3	0.378042998	0.192059267	2.713325067	0.008024905	-2.559224404
RP11-781M16.2	0.222125621	0.106625186	2.713291816	0.008025647	-2.559300447
GAPDHP25	0.220988439	0.277588186	2.713207673	0.008027523	-2.559492872

TBX4	0.544908126	1.216154117	2.712616596	0.008040718	-2.560844453
NDUFA6	-0.413317727	5.462192863	-2.712325028	0.008047234	-2.561511075
KANK1	0.404334786	1.606923563	2.711987603	0.008054781	-2.562282468
MIR4505	0.357585178	0.239348319	2.711582552	0.008063849	-2.563208358
SLC35C1	-0.313934513	3.110103696	-2.711178616	0.008072902	-2.564131582
SMAD9-IT1	0.314371811	0.423290017	2.710740819	0.008082723	-2.565132069
MIR1278	0.295750069	0.183944637	2.71024626	0.008093831	-2.566262113
CRYGA	0.247251685	0.106578806	2.710178524	0.008095354	-2.566416874
SUMF1	-0.31824137	2.162996327	-2.710061649	0.008097981	-2.566683897
RP11-525J21.1	0.214508998	0.085666737	2.710045173	0.008098352	-2.566721539
GAPDHP51	0.209946891	0.14960515	2.710027011	0.00809876	-2.566763031
PTPDC1	0.289851669	1.842563607	2.709548896	0.008109518	-2.567855266
PEF1	-0.319012303	4.914595514	-2.709451498	0.008111711	-2.568077749
RP11-647P12.1	0.216544717	0.217774321	2.708534423	0.008132387	-2.570172257
COX6B1P2	0.496066306	0.219013353	2.708475426	0.008133719	-2.57030698
RP11-312B8.2	0.395583282	0.387850385	2.708090616	0.008142411	-2.571185659
ITPK1	-0.324621048	2.673960308	-2.707763235	0.008149812	-2.571933121
AC023672.1	0.271145268	0.238815363	2.707321835	0.0081598	-2.572940788
RP11-480O10.2	0.235704629	0.084406899	2.707185755	0.008162881	-2.573251415
RP11-153F5.2	0.249363536	0.079084018	2.706924852	0.008168793	-2.573846939
RP11-293P20.2	0.280783628	0.149069338	2.706349721	0.008181837	-2.575159534
RP11-667K14.3	-0.356928269	1.330152781	-2.706116187	0.008187139	-2.575692451
GLUDP5	0.304809456	0.22684087	2.706057287	0.008188477	-2.575826852
AC007193.6	0.337994558	0.280205251	2.705535378	0.008200339	-2.577017675
FUNDC2	-0.28297966	2.478239561	-2.705471349	0.008201795	-2.577163755
CCRL2	-0.276292301	0.878114341	-2.704670573	0.008220029	-2.578990451
RP11-526K21.2	0.246755874	0.124966487	2.70385919	0.008238542	-2.580840885
SHOX2	0.585393522	3.385254999	2.703334239	0.00825054	-2.582037838
RP11-713C19.2	0.237839956	0.554158265	2.703132854	0.008255147	-2.58249697
PLCB4	0.524903641	1.877673163	2.703049315	0.008257059	-2.58268742
RP11-706C16.7	0.358735722	0.141075234	2.702955763	0.0082592	-2.582900691
ATF6B	0.331982618	4.721197002	2.702851449	0.008261588	-2.58313849
RNU6-1162P	0.356374272	0.215038952	2.702443616	0.008270932	-2.584068128

MYO1F	-0.415112066	1.686929762	-2.701745946	0.008286938	-2.585658169
RP11-552E4.2	0.29776943	0.117224762	2.701607406	0.00829012	-2.58597387
RNU6-989P	0.269977445	0.085651737	2.701564047	0.008291116	-2.586072674
RP11-399D15.1	0.203529407	0.099421981	2.700533911	0.008314811	-2.588419673
RN7SKP46	0.257421495	0.109152077	2.700456765	0.008316588	-2.588595408
LINC01017	0.200713337	0.082231706	2.700055194	0.008325844	-2.589510099
HNRNPUL1	0.3513381	5.544716383	2.699303456	0.008343196	-2.591222092
LINC01221	0.732577715	0.873402789	2.699236777	0.008344736	-2.591373927
CBR3-AS1	0.219456834	0.473383811	2.698629502	0.00835878	-2.592756605
RN7SL399P	0.283129156	0.137295125	2.698341514	0.008365447	-2.593412223
RP11-17A4.3	0.21461046	0.084928342	2.698240288	0.008367792	-2.593642653
RP11-671J11.1	0.307445367	0.248756725	2.698199661	0.008368733	-2.593735134
MGA	0.238012997	1.831277181	2.698079843	0.00837151	-2.594007876
SNHG22	0.227103947	0.286803822	2.697038057	0.008395685	-2.59637887
RP11-705C15.2	-0.298506721	1.269394706	-2.696936091	0.008398055	-2.596610894
RNU7-193P	0.380972357	0.34218226	2.696815714	0.008400853	-2.5968848
MIR519A1	0.275701962	0.104996333	2.695218446	0.008438063	-2.600518285
RP11-432M8.18	0.390161266	0.147393402	2.695212854	0.008438194	-2.600531002
IMMP1L	-0.227048307	1.794362854	-2.694799837	0.00844784	-2.601470245
STX4	-0.347465331	3.212029533	-2.694451356	0.008455986	-2.602262634
RHBDD2	-0.338373459	4.789738727	-2.694291645	0.008459722	-2.602625761
UXS1	-0.303238384	2.938778156	-2.694035165	0.008465725	-2.603208872
RNU6-993P	0.322395428	0.132499314	2.693929535	0.008468199	-2.60344901
TCERG1	0.259059455	2.648134287	2.693766257	0.008472023	-2.603820188
TBCAP3	0.22934489	0.186334618	2.69344947	0.008479448	-2.604540283
RP11-629G13.1	0.419638656	0.706904999	2.693233164	0.008484521	-2.605031931
INVS	0.245634497	1.840411249	2.692861729	0.008493239	-2.605876102
RP11-332H18.3	0.20132853	0.319076003	2.692505222	0.008501614	-2.606686254
CPO	0.224686891	0.107960789	2.692390449	0.008504312	-2.606947053
RP11-809N8.4	0.267359033	0.452455545	2.692248795	0.008507643	-2.607268922
MID1IP1-AS1	0.459003672	1.234652927	2.692139426	0.008510215	-2.607517424
CDC42P1	0.252250541	0.437532147	2.691595409	0.008523022	-2.608753375
RN7SL492P	0.291871138	0.107318023	2.691548256	0.008524132	-2.608860491

RNU6-21P	0.358978862	0.120306931	2.691503268	0.008525192	-2.608962689
DAW1	0.204007382	0.076835954	2.690965757	0.008537866	-2.610183622
RP11-137L10.5	0.307565236	0.236871649	2.690649859	0.008545322	-2.610901074
NSMCE1	-0.368731035	3.219463073	-2.690411521	0.008550952	-2.61144233
C5orf34	0.292566762	1.585404417	2.690324278	0.008553013	-2.611640447
C1orf143	0.218039805	0.13169464	2.690237759	0.008555058	-2.611836912
FBXL4	0.264525225	1.710542848	2.689711508	0.008567505	-2.613031801
NAGA	-0.31238587	3.900839054	-2.689643926	0.008569105	-2.613185237
PLA2G15	-0.349552357	2.98237179	-2.689568868	0.008570882	-2.613355643
RP11-749H20.1	0.279104209	0.128602076	2.688374102	0.008599213	-2.6160676
RP11-122M14.3	0.26659535	0.126013677	2.688327173	0.008600327	-2.616174104
RNU6-1261P	0.3098508	0.114056497	2.688109058	0.008605509	-2.616669078
BCL6	0.398824237	2.699514796	2.686875994	0.008634857	-2.619466688
MDC1	0.407936845	3.15088235	2.686426824	0.00864557	-2.62048551
MIR1276	0.270315954	0.338505847	2.686408435	0.008646009	-2.620527218
RP11-126D17.1	0.399447019	0.208561196	2.686377422	0.008646749	-2.620597557
SYVN1	0.265195612	3.7813074	2.68596513	0.008656594	-2.621532593
IRAK3	0.479637419	1.278867144	2.685833634	0.008659736	-2.621830787
RP11-89M22.3	0.229973899	0.10357083	2.685786701	0.008660858	-2.621937215
DNAJC5G	0.264793725	0.130146741	2.685091755	0.008677484	-2.623512924
SELK	-0.346562753	3.56608015	-2.685016914	0.008679276	-2.623682597
RP11-159F24.5	0.315746971	0.480886854	2.684675374	0.008687459	-2.624456857
RNU6-710P	0.22754685	0.074017375	2.683858251	0.008707065	-2.626308913
RP11-7M10.2	0.20435768	0.202721479	2.68360252	0.008713209	-2.626888446
AC013251.1	0.362853486	0.326616184	2.682791419	0.008732723	-2.628726243
RP11-760D2.9	0.358343294	0.162151609	2.682724259	0.008734341	-2.628878395
RP11-356K23.2	0.298824693	0.204375614	2.682418456	0.008741709	-2.629571151
RP11-47P18.1	0.246495668	0.181742738	2.682356208	0.00874321	-2.629712156
RP11-301A5.2	0.383037765	0.195627366	2.68233873	0.008743631	-2.629751747
RP11-54F2.9	0.215920453	0.086291145	2.682257248	0.008745596	-2.62993632
ZNF490	0.203435893	0.575058597	2.682204719	0.008746863	-2.630055304
RNF185	-0.296074621	3.401392961	-2.682023411	0.008751237	-2.630465975
SUMO2P19	0.294576472	0.29708442	2.681501167	0.008763847	-2.631648751

SND1	0.30435847	5.745176577	2.680943859	0.008777321	-2.632910728
DONSON	0.444707548	3.163374045	2.680865008	0.008779229	-2.633089262
RP1-102G20.5	0.262663455	0.258937099	2.680732959	0.008782425	-2.633388237
RP11-1084E5.1	0.263924585	0.132152407	2.679709486	0.008807233	-2.635705087
RP11-345K9.2	0.201451556	0.153741787	2.679476902	0.008812879	-2.636231487
RP11-449H3.1	0.339450409	0.471046126	2.678831798	0.008828557	-2.637691334
ERN1	0.354742312	1.624451352	2.678387084	0.00883938	-2.638697532
CGREF1	0.945010207	3.139804802	2.678182106	0.008844373	-2.639161264
RP11-395G23.3	-0.550024397	2.92261428	-2.677935199	0.00885039	-2.639719815
CSF3R	-0.303350562	0.744350911	-2.677915703	0.008850865	-2.639763917
ERCC6L2	0.234051614	1.348320154	2.677603556	0.008858478	-2.640469988
BCKDHB	0.323127385	2.280814569	2.677464976	0.008861859	-2.64078343
SLC43A2	-0.407309469	1.618497577	-2.676563911	0.008883876	-2.642821149
RNU6-192P	0.302647528	0.121506123	2.675557598	0.008908523	-2.645096206
AL136234.1	0.382699726	0.172610774	2.675407787	0.008912198	-2.645434833
OAZ1	-0.295440923	6.456046883	-2.675333246	0.008914026	-2.645603318
RNA5SP204	0.254941468	0.114217897	2.675120396	0.00891925	-2.646084402
RP11-15I11.2	0.209883408	0.21899579	2.674710129	0.008929328	-2.647011594
PEBP1P3	0.278355782	0.187517102	2.674588901	0.008932307	-2.647285546
RNU6-1064P	0.282995739	0.223669819	2.674430825	0.008936194	-2.647642747
C2CD3	0.230313829	1.614956955	2.6744275	0.008936276	-2.647650261
AC002539.2	0.270984619	0.097289025	2.674273388	0.008940067	-2.647998488
RP11-386P4.1	0.206191819	0.0873085	2.674243393	0.008940804	-2.648066263
SNORD74	0.293032486	0.255563415	2.67420865	0.008941659	-2.648144764
RP11-466F5.4	0.216538868	0.215780362	2.674195817	0.008941975	-2.648173761
RN7SL636P	0.209231072	0.126369509	2.674114207	0.008943983	-2.648358153
RPL7P11	0.233823162	0.466178013	2.674049137	0.008945585	-2.648505172
DGUOK	-0.298208068	4.843710515	-2.673198422	0.008966548	-2.650426995
CTC-490E21.13	0.23813526	0.092609189	2.67305481	0.008970092	-2.650751372
RNU6-837P	0.324106932	0.105406739	2.672855573	0.00897501	-2.651201369
MIR566	0.240970237	0.183454495	2.67262175	0.008980784	-2.651729444
RP11-731C17.1	0.241432839	0.134978757	2.671404728	0.009010895	-2.654477397
CTD-2125J1.1	0.213604622	0.081063187	2.671025681	0.009020292	-2.655333046

ZFC3H1	0.280537235	1.882792137	2.670885835	0.009023761	-2.655648705
PTPN14	0.385059841	3.211744056	2.670484785	0.009033716	-2.656553872
SETP7	0.204845784	0.123254454	2.67034751	0.009037126	-2.656863674
RP11-203M5.2	0.242083458	0.172295737	2.670100921	0.009043255	-2.657420145
RP11-424D14.1	0.251031471	0.118644715	2.669883064	0.009048672	-2.65791174
RNU7-30P	0.301366008	0.149893378	2.669499775	0.00905821	-2.658776553
RP11-416N2.4	0.332957938	0.538504337	2.669145037	0.009067046	-2.659576853
RP11-736N17.10	0.412201735	0.358524585	2.667953458	0.009096784	-2.662264448
RP11-166N6.2	0.203803568	0.069381508	2.667880447	0.009098609	-2.66242909
AC106870.2	0.302228724	0.135555114	2.667790908	0.009100847	-2.662631
GMEB2	0.244510189	2.5256073	2.667765986	0.00910147	-2.662687198
RP11-325K19.1	0.20446546	0.083476681	2.667737743	0.009102177	-2.662750882
HNRNPCP9	0.233534152	0.116026962	2.667648481	0.009104409	-2.662952159
RP11-142L1.3	0.212649339	0.171147182	2.667502918	0.00910805	-2.663280375
LMLN	0.230348125	1.227063652	2.667395873	0.009110729	-2.663521729
DLX1	0.492003091	1.711777002	2.667283345	0.009113546	-2.663775439
SERBP1P3	0.224178989	0.471989232	2.666795714	0.009125761	-2.664874766
MIR4767	0.535914034	0.931233237	2.66660875	0.009130449	-2.665296218
SNORA64	0.321732693	0.211796262	2.66634447	0.009137079	-2.665891913
RNU6-233P	0.315921403	0.239743258	2.666127035	0.009142536	-2.66638198
RNU6-143P	0.355469696	0.216905081	2.66558272	0.009156212	-2.66760864
PDZD11	-0.346647197	4.655442946	-2.665478768	0.009158826	-2.667842882
RP11-438P9.1	0.360209253	0.390899004	2.665314294	0.009162963	-2.668213486
RN7SL580P	0.352578999	0.154616335	2.664764137	0.009176814	-2.669452995
GSS	-0.331041765	4.454610215	-2.664661662	0.009179396	-2.669683851
PITPNM1	0.406620872	2.406251904	2.664618367	0.009180487	-2.669781382
SF1	0.227994925	5.280685067	2.66377934	0.009201654	-2.671671221
RNU6-262P	0.290141837	0.216301277	2.663752174	0.00920234	-2.671732402
MUC1	0.568953922	1.345008396	2.663193701	0.009216455	-2.672990031
PABPC1P10	0.294786737	0.727022134	2.662918425	0.00922342	-2.673609845
PLEKHA3	0.233828857	1.490266133	2.662875112	0.009224516	-2.673707364
MEMO1	0.227743766	1.292451294	2.662675624	0.009229567	-2.674156495
MIR548P	-0.31871971	0.325446713	-2.66223837	0.009240646	-2.675140836

MTMR12	0.381662221	2.475573178	2.661987083	0.009247019	-2.675706469
MYL6B	-0.43367618	3.520882611	-2.661974006	0.00924735	-2.675735905
SETP3	0.228088164	0.484240645	2.661324237	0.009263847	-2.677198287
ZC3H6	0.268041752	1.027268117	2.6609645	0.009272992	-2.678007788
RP11-148K1.10	0.307198785	0.441305747	2.660858238	0.009275695	-2.678246889
RN7SKP229	0.212327595	0.121883375	2.660742679	0.009278635	-2.678506899
ZFR	0.293632004	3.761181109	2.660708518	0.009279505	-2.678583759
RNA5SP367	0.331835508	0.211419462	2.660704697	0.009279602	-2.678592356
RP4-764O22.1	0.372902228	0.242289185	2.660558835	0.009283315	-2.678920532
RP11-5O23.1	0.223536522	0.12851773	2.660408313	0.009287148	-2.679259175
RNU6-252P	0.209635646	0.105336715	2.660358031	0.009288428	-2.679372296
BLOC1S4	-0.346565899	3.062685372	-2.660286843	0.009290242	-2.679532445
RP11-11L12.2	0.23170648	0.185789224	2.660129482	0.009294251	-2.679886445
RP11-57G10.1	0.268961557	0.145681886	2.659913757	0.009299751	-2.680371711
RP3-343K2.4	0.28264211	0.180288421	2.659847239	0.009301447	-2.680521336
RP11-643G5.2	0.221090103	0.078353908	2.659617728	0.009307302	-2.681037565
AC027612.2	0.214967443	0.079695781	2.659584813	0.009308142	-2.681111597
RP4-800G7.3	0.255259253	0.497324137	2.65950693	0.00931013	-2.681286766
RP11-5N11.4	0.393541747	0.196428161	2.65870722	0.009330562	-2.683085171
METTL21AP1	0.2137214	0.147165034	2.658557418	0.009334394	-2.683421998
LTB	-0.525842849	1.077320044	-2.658458678	0.009336921	-2.683644005
CCDC58P5	0.269309615	0.225516143	2.657897987	0.00935128	-2.684904532
RPL36P18	0.20022212	0.108082402	2.657562844	0.009359873	-2.685657882
RP11-390D11.1	0.276019478	0.224115555	2.657445899	0.009362872	-2.685920738
SPATA18	0.384342497	0.781121418	2.657329568	0.009365858	-2.686182206
GPX4	-0.386774956	6.492706097	-2.657252772	0.009367829	-2.686354808
RNU4-16P	0.363215857	0.132204102	2.657030753	0.009373529	-2.686853781
AC104532.3	0.215171638	0.239039616	2.656886896	0.009377224	-2.68717707
SIRPB2	-0.223430736	0.546493393	-2.656813683	0.009379106	-2.687341596
CTD-2555K7.3	0.249406813	0.137574004	2.65611682	0.009397028	-2.688907415
AC022816.2	0.204886765	0.190527666	2.655798614	0.009405222	-2.689622295
RPP25	0.512373255	3.002547527	2.655758843	0.009406247	-2.689711639
UQCR11	-0.359688299	4.669062811	-2.655599256	0.009410359	-2.690070137

ORMDL3	-0.298123468	3.836630779	-2.655544699	0.009411766	-2.690192688
HTR3E-AS1	0.235218632	0.097363037	2.655319333	0.009417577	-2.690698912
HIST1H1D	0.402121829	0.581279942	2.655118277	0.009422764	-2.691150497
RP11-737G21.1	0.236987063	0.082934291	2.654671689	0.009434294	-2.692153465
ATP11A-AS1	0.20096841	0.148084922	2.65437262	0.009442023	-2.692825048
AC005487.2	0.237999969	0.092302243	2.654090259	0.009449326	-2.693459053
RPL21P7	0.313286969	0.590655479	2.65397	0.009452437	-2.693729063
RNU6-456P	0.401309415	0.135581254	2.653801558	0.009456797	-2.694107237
MTMR14	-0.250513509	2.276992965	-2.653378926	0.009467745	-2.695056014
KLHL28	0.381592719	1.589509449	2.65287975	0.00948069	-2.696176461
AC012363.13	0.260734325	0.131102812	2.652721262	0.009484803	-2.696532164
PEX5	-0.335003673	2.814236233	-2.652402884	0.009493071	-2.697246668
RP11-123C5.5	0.244727614	0.157449281	2.652240389	0.009497294	-2.69761131
LGALS1	-0.596823256	9.45184972	-2.651872466	0.009506861	-2.698436868
RP11-545P6.2	0.216549494	0.078732735	2.651593359	0.009514124	-2.699063074
TUBBP11	0.314000977	0.128353017	2.651368745	0.009519973	-2.69956698
AC110611.1	0.385209362	0.364686637	2.651312541	0.009521437	-2.699693064
C9orf47	0.205488322	0.456686294	2.651169852	0.009525154	-2.700013151
RP11-10D7.5	0.231311522	0.248887203	2.650897138	0.009532263	-2.700624879
SHCBP1L	0.207684629	0.150821017	2.650244516	0.009549296	-2.70208857
RN7SL596P	0.339309199	0.301633657	2.649629858	0.009565362	-2.70346684
TRAPPC10	0.270764188	1.502218124	2.64942703	0.009570669	-2.70392159
RP11-230C9.1	0.254535219	0.123901212	2.648829865	0.00958631	-2.705260291
RP1-81D8.4	0.291672579	0.104112849	2.648575506	0.009592979	-2.705830426
B4GALNT2P1	0.299568633	0.101924185	2.647973225	0.009608787	-2.707180233
CTC-251I16.1	-0.598212848	1.68935754	-2.647542691	0.009620102	-2.708144971
HSCB	-0.336274705	2.943000978	-2.647164275	0.009630057	-2.708992814
RP11-120I21.3	0.318123305	0.181519919	2.647150733	0.009630414	-2.709023154
TNFRSF1A	-0.376130431	5.101023828	-2.646948925	0.009635727	-2.70947526
DNAJC21	0.267094369	2.527393013	2.646617811	0.00964445	-2.71021699
RP11-297L6.2	0.220053359	0.123823369	2.64660931	0.009644674	-2.710236032
TMEM242	-0.263495263	2.06732801	-2.646386699	0.009650542	-2.710734659
AC012627.1	0.421524299	0.493156985	2.646298745	0.009652862	-2.710931658

RP11-299H22.7	0.24919972	0.082557522	2.645823893	0.009665394	-2.711995132
POC1B	0.276032672	1.516758526	2.645811376	0.009665725	-2.712023162
CDRT7	0.330325527	0.111719136	2.645805184	0.009665888	-2.712037029
NAV1	0.37159338	2.898218069	2.645656912	0.009669805	-2.712369062
RNA5SP19	0.353156011	0.2093052	2.64553946	0.009672908	-2.712632066
IRF1	-0.425425366	2.340720675	-2.645346393	0.009678011	-2.713064374
IMMP1LP3	0.430321239	0.354931826	2.645225777	0.0096812	-2.713334438
CHCHD2P4	0.222594377	0.103380231	2.645121343	0.009683963	-2.713568262
C2orf88	0.296822378	0.631955297	2.645040188	0.00968611	-2.713749959
RP11-388G22.1	0.378290598	0.151016091	2.644911778	0.009689508	-2.714037447
XCL2	-0.374481189	0.530152485	-2.644189879	0.009708631	-2.71565343
ADH5	-0.279493016	4.279125223	-2.643885657	0.0097167	-2.716334327
AL359851.1	0.235064682	0.138459442	2.643830896	0.009718154	-2.716456884
NKG7	-0.709139185	1.567788681	-2.643767447	0.009719837	-2.716598881
CSF1	-0.772187305	4.45116872	-2.643564166	0.009725234	-2.717053803
GSC	0.608243326	2.250214067	2.643406527	0.009729421	-2.717406562
RP11-157P23.2	0.29903783	0.106146775	2.642792144	0.009745755	-2.718781237
CTD-2213F21.4	0.217971223	0.07501067	2.642714177	0.00974783	-2.718955669
C21orf62	0.229761125	0.14415894	2.640670621	0.009802349	-2.723526067
MED13L	0.354403844	2.569198408	2.64000339	0.00982021	-2.725017683
ARHGAP11A	0.390134964	2.672795725	2.639527805	0.009832958	-2.726080676
MIR181A2HG	0.358605618	1.19135678	2.639395477	0.009836508	-2.726376417
RP11-535M15.1	0.559724706	2.669938765	2.639361672	0.009837415	-2.726451967
RP11-383G6.3	0.243947028	0.315218542	2.63928371	0.009839507	-2.726626196
OSBP	0.298408664	3.778649241	2.639137078	0.009843443	-2.72695388
EIF2A	0.287865608	4.126574981	2.639042582	0.00984598	-2.727165045
RP11-288K12.1	0.275980138	0.209197698	2.638602071	0.009857816	-2.728149347
RNU6-1018P	0.339255542	0.145170691	2.638463931	0.009861531	-2.728457987
MIR1285-2	0.289610048	0.133800516	2.638415924	0.009862822	-2.728565243
RP11-399E6.2	0.220350802	0.136395067	2.638216987	0.009868174	-2.729009688
AC010243.1	0.291056445	0.202208913	2.637481657	0.009887978	-2.730652244
NEDD8	-0.425661454	4.232195555	-2.63713146	0.009897423	-2.731434367
AL606500.1	0.35476225	0.471719274	2.636828316	0.009905605	-2.732111334

AC131011.1		0.211546278	0.194947321	2.636645592	0.00991054	-2.73251935
RNU6-672P		0.382592284	0.433561203	2.635897216	0.009930775	-2.73419021
SDHAF2		-0.267251782	2.798035313	-2.634609188	0.009965689	-2.737064989
ACACB		0.225271443	1.057685037	2.633794415	0.009987832	-2.738882893
DYRK4		-0.32815089	1.843144971	-2.632844001	0.010013718	-2.741002837
RP11-210K20.6		0.321137816	0.155476629	2.632156778	0.010032473	-2.742535324
RN7SL810P		0.257699851	0.128394512	2.631766819	0.01004313	-2.74340477
HMGB1P28		0.265300974	0.138150356	2.631552598	0.010048988	-2.743882349
NFX1		0.28164447	2.799332517	2.630742838	0.010071162	-2.745687309
AC073046.1		0.229529566	0.110411693	2.630739344	0.010071257	-2.745695097
SNX25		0.299657019	2.315488484	2.630512679	0.010077472	-2.74620025
RN7SL648P		0.338662604	0.555704892	2.630498669	0.010077856	-2.746231471
	1-Mar	-0.283487564	0.80444233	-2.630469567	0.010078654	-2.746296328
RP11-871F6.3		0.201925174	0.108321515	2.629793992	0.0100972	-2.747801699
ZNF131		0.315854847	2.519708286	2.629195607	0.010113652	-2.749134801
NNAT		0.486920261	0.792381738	2.628146629	0.010142552	-2.751471133
TRAJ36		0.366379389	0.244873701	2.62812798	0.010143066	-2.751512662
FXYD5		-0.552032162	5.156753339	-2.627878613	0.010149947	-2.752067945
RP11-236P24.1		0.200763616	0.199313113	2.626946645	0.010175703	-2.754142834
PHIP		0.318570842	2.458342538	2.626568913	0.010186159	-2.754983623
PRKCA		0.494356322	2.510217841	2.626500649	0.010188049	-2.75513556
RN7SL535P		0.394119291	0.570180854	2.626477441	0.010188692	-2.755187212
RP11-239E10.3		0.229810165	0.095627062	2.626143486	0.010197946	-2.755930453
AC012370.3		0.223646335	0.134248401	2.625560896	0.010214109	-2.757226856
AC010655.1		0.455342203	0.168099108	2.625478208	0.010216404	-2.757410838
RN7SL304P		0.243832951	0.100831266	2.625381247	0.010219097	-2.75762657
EFNA5		-0.515120547	1.982302096	-2.62534999	0.010219965	-2.757696113
RN7SL526P		0.318392736	0.62401652	2.62534321	0.010220154	-2.757711198
RP11-3K16.1		0.230802847	0.351721514	2.62526489	0.010222329	-2.757885448
RSRC1		0.278167907	2.621615826	2.624472741	0.010244358	-2.759647617
AC138123.1		0.208376258	0.080485366	2.624260807	0.010250259	-2.760118997
RP11-443P15.2		0.212401418	0.19086502	2.623962418	0.010258573	-2.760782615
GK5		0.306563144	1.367184438	2.623911874	0.010259982	-2.760895019

FAM175A	0.321115902	1.164932392	2.623715082	0.010265469	-2.761332642
AC108044.1	0.281010921	0.136745135	2.623485923	0.010271861	-2.76184221
AL160011.1	0.537337382	0.481785067	2.623395208	0.010274393	-2.762043918
RNU4-34P	0.205035125	0.128145293	2.623380724	0.010274797	-2.762076123
YBX3	-0.713522308	3.093569932	-2.623140466	0.010281505	-2.76261031
CNKSR2	0.473041754	0.787738505	2.622948128	0.010286878	-2.763037925
RNU6-145P	0.314534914	0.221182028	2.622893519	0.010288404	-2.763159329
RP11-141B14.2	0.212047425	0.164626396	2.622824516	0.010290332	-2.763312731
FAM212A	-0.352467455	2.060469785	-2.622810348	0.010290728	-2.763344229
AC073850.6	0.359235995	0.52127122	2.62274366	0.010292592	-2.763492478
RN7SL62P	0.206323268	0.115641712	2.621272906	0.010333782	-2.766761238
CDC14B	0.355145126	2.001522913	2.621259504	0.010334158	-2.766791016
AC105402.2	0.270498923	0.237418683	2.62122298	0.010335183	-2.766872172
SLC36A4	0.339919201	2.091136143	2.621207756	0.01033561	-2.766905998
PGLS	-0.395267307	3.891937146	-2.620873631	0.01034499	-2.767648362
AC004449.6	-0.245645247	0.504138134	-2.620718936	0.010349335	-2.767992041
AC005546.2	0.336466987	0.741343792	2.620673003	0.010350625	-2.768094083
AC118550.1	0.302445538	0.132946729	2.62058862	0.010352997	-2.768281542
MIR3170	0.375350275	0.389046374	2.620432122	0.010357396	-2.768629193
RNU6-874P	0.468581645	0.291822534	2.620372666	0.010359067	-2.768761267
RP11-814E24.1	0.256978461	0.129144683	2.620108733	0.010366491	-2.769347527
AL590085.1	0.574079451	0.423526092	2.620089421	0.010367034	-2.769390421
RP5-1116H23.6	0.234478257	0.191690825	2.619748742	0.010376625	-2.770147075
CTA-331F8.1	0.210643888	0.302159037	2.619562918	0.010381859	-2.77055976
CCNB3P1	0.299307177	0.121167084	2.619507811	0.010383412	-2.770682139
RP5-1106H14.1	0.220439961	0.092110842	2.619478393	0.010384241	-2.770747468
RP11-474I16.8	0.262260826	0.30644601	2.619429488	0.010385619	-2.77085607
AC022395.1	0.353670555	0.188137831	2.619114741	0.010394493	-2.771554984
TMEM219	-0.350936621	4.548101488	-2.618926944	0.010399791	-2.771971963
RP11-646E18.2	0.252469167	0.116420786	2.618504089	0.01041173	-2.77291077
UROD	-0.35145346	4.234697768	-2.618406251	0.010414494	-2.773127967
IL11	0.839416974	1.023756802	2.617832499	0.010430716	-2.774401546
MRPL51	-0.443636387	5.499060872	-2.61782505	0.010430927	-2.774418079

TMEM115	-0.270032766	4.303685287	-2.61774044	0.010433321	-2.774605871
UQCRBP3	0.212927612	0.097227298	2.617338982	0.010444688	-2.775496836
RP11-475D10.5	0.273499492	0.094482721	2.617111125	0.010451145	-2.776002473
AC092933.4	0.222581079	0.13361094	2.61665285	0.010464142	-2.777019318
FTLP3	-0.559952987	5.140190735	-2.616529745	0.010467636	-2.777292446
RP11-487E1.2	0.292390426	0.304387556	2.615734681	0.010490226	-2.779056151
PSME2P2	-0.441700762	1.770031644	-2.615113929	0.010507894	-2.780432865
MXD1	0.352678951	2.037438256	2.61499701	0.010511225	-2.780692139
RP3-507I15.2	0.245386777	0.150243879	2.614648847	0.010521149	-2.78146415
CCDC14	0.307619751	2.089365919	2.614618945	0.010522001	-2.781530451
RNU6-1238P	0.295673738	0.422488148	2.614614146	0.010522138	-2.781541092
CTD-2025J6.1	0.322411771	0.142226506	2.614140591	0.010535651	-2.782590994
RP11-363G15.2	0.258178091	0.119345375	2.613662142	0.01054932	-2.783651587
PRRG1	0.303181259	1.770614666	2.613448527	0.010555428	-2.784125062
SUMO2P18	0.310608139	0.229792386	2.61325744	0.010560894	-2.784548575
LINC00605	0.228138708	0.214883077	2.61321212	0.010562191	-2.784649014
RP3-416J7.1	0.245566553	0.086199435	2.612861083	0.010572241	-2.785426952
HMMR	0.485564189	2.63485983	2.612768067	0.010574906	-2.785633071
VSTM1	0.219993408	0.125470042	2.612435462	0.010584438	-2.78637006
RNU2-13P	0.273443621	0.127764602	2.611990289	0.010597209	-2.78735635
AC233299.1	0.23469299	0.079863827	2.611866477	0.010600763	-2.787630634
RNU4-68P	0.38969027	0.559099261	2.611839991	0.010601523	-2.787689308
CTD-3066C23.1	0.224705541	0.15552465	2.611596669	0.010608512	-2.788228307
FAM73A	0.292391038	2.106072537	2.611334209	0.010616055	-2.788809655
NNT	0.340664745	3.281412691	2.611131361	0.010621888	-2.789258927
RP11-224P11.1	0.205804858	0.085824839	2.611103044	0.010622703	-2.789321644
RNA5SP197	0.310352016	0.183162919	2.610821561	0.010630803	-2.789945025
RP11-648O15.2	0.252164885	0.159403777	2.610415561	0.010642495	-2.790844065
SNORD7	-0.411442683	0.70818483	-2.610090508	0.010651865	-2.791563774
RP11-458F8.1	0.38291888	1.128795489	2.609791059	0.010660503	-2.792226725
RP5-866L20.2	0.332260791	0.196673358	2.609772996	0.010661024	-2.792266714
RPL21P5	0.354303341	0.471764761	2.60965465	0.01066444	-2.792528702
OSMR-AS1	0.266474043	0.558905923	2.609587694	0.010666373	-2.79267692

RNU6-753P	0.560127488	0.20804544	2.609218955	0.010677024	-2.793493132
APOC1	-0.78648809	3.703174268	-2.609140651	0.010679287	-2.793666446
PNPLA4	-0.421762796	2.174148231	-2.60892747	0.010685451	-2.79413827
RP1-128O3.5	0.231752112	0.179121557	2.608703833	0.01069192	-2.794633203
HLA-A	-0.533372201	7.972182422	-2.608670649	0.01069288	-2.794706638
TPCN2	0.314601613	2.079887411	2.608549292	0.010696392	-2.794975197
DDHD2	0.366946151	1.976652563	2.607797787	0.010718165	-2.796638008
RP11-359K18.4	0.50401627	1.507091215	2.60778509	0.010718533	-2.796666098
CFTRP3	0.255634521	0.094520184	2.607730578	0.010720114	-2.796786699
RP11-252M24.1	0.218750691	0.071903865	2.607726722	0.010720226	-2.796795229
OOSP1P1	0.280987284	0.112497701	2.607653963	0.010722337	-2.796956193
RNA5SP517	0.260900409	0.084531521	2.607652019	0.010722393	-2.796960493
AP001469.9	0.280786748	0.780252126	2.606597916	0.010753011	-2.799292058
PHLPP1	0.343965624	1.818511508	2.606530161	0.010754982	-2.799441898
IL23R	0.202516737	0.230133949	2.606214538	0.010764167	-2.800139853
AC087499.7	0.201316471	0.124639663	2.605793796	0.010776422	-2.801070154
WDR44	0.421985414	2.464268259	2.605544215	0.010783697	-2.80162194
USF2	-0.32972979	4.681122757	-2.605191614	0.010793983	-2.802401414
PRAMEF17	0.214305956	0.078336074	2.604840754	0.010804227	-2.80317695
CXCL9	-0.906974142	1.526733558	-2.604446311	0.010815754	-2.804048718
MIR4644	0.21159583	0.135213356	2.604128881	0.010825039	-2.804750196
RNU6-344P	0.310992875	0.10148853	2.603913657	0.010831338	-2.805225771
RP11-59H7.1	0.408684846	0.226886835	2.603481154	0.010844006	-2.806181364
SLC26A3	0.204015245	0.091401484	2.603105008	0.010855035	-2.807012328
MYO5C	0.238797263	0.648183166	2.603089863	0.010855479	-2.807045784
RN7SKP224	0.215064082	0.104196322	2.602838245	0.010862863	-2.807601589
CIRBP	-0.312355061	4.677642879	-2.602818866	0.010863431	-2.807644393
FAM210A	0.323374344	2.620032923	2.601703012	0.010896231	-2.810108667
HLA-B	-0.644681493	7.596144129	-2.601145235	0.01091266	-2.81134014
SGK494	0.265818206	0.906752511	2.600615514	0.010928283	-2.812509463
RNU6-1223P	0.37346736	0.655401796	2.600522262	0.010931036	-2.812715289
CASP8AP2	0.302496525	1.729241477	2.600371782	0.010935479	-2.813047416
BCL2L11	0.403576392	2.528524325	2.59895041	0.010977525	-2.816183761

RNU6-463P	0.250892813	0.082304651	2.598888057	0.010979373	-2.816321313
RP11-575L7.8	0.382510795	1.081608568	2.598789371	0.010982298	-2.816539011
LINC00383	0.203975751	0.089911453	2.598240658	0.010998575	-2.817749331
RNU6-1092P	0.388498038	0.258412369	2.597979579	0.011006327	-2.818325128
BEND2	0.365542146	0.127046983	2.597841598	0.011010426	-2.818629417
AL022344.2	0.233286488	0.094871897	2.596536324	0.011049272	-2.821507276
CNNM1	0.246804578	0.217117609	2.595911677	0.011067905	-2.822884062
GPR142	0.232981559	0.208638185	2.595484932	0.011080651	-2.823824491
TMEM51	-0.385067024	3.177621236	-2.595001035	0.011095121	-2.82489071
ZNF532	0.368962176	2.864883251	2.594921774	0.011097492	-2.825065338
RP11-348H3.5	0.31513539	0.503919638	2.594338488	0.01111496	-2.826350295
BATF	-0.586138475	1.605902264	-2.594049156	0.011123634	-2.82698759
IGHVII-67-1	0.22920025	0.073825072	2.59391273	0.011127725	-2.827288069
AL022400.1	0.493337425	0.20965296	2.593567344	0.011138091	-2.828048721
CAPN15	0.341723158	2.817487311	2.593360029	0.011144317	-2.828505255
AC024162.2	0.290509772	0.140167448	2.592900024	0.011158143	-2.829518134
RP11-296O14.3	0.283436522	0.816419871	2.592713835	0.011163744	-2.829928058
MIR4474	0.265398862	0.107393401	2.592481462	0.011170737	-2.830439628
BOLA2P2	0.297206387	0.491560009	2.592340528	0.01117498	-2.830749876
RP4-550H1.4	0.23555389	0.149714061	2.591679382	0.011194906	-2.832205115
BNIP3P1	0.368727557	0.876696398	2.591404043	0.011203214	-2.832811067
RNU7-26P	0.273523001	0.243838008	2.591391591	0.011203589	-2.832838471
ADIPOR1	0.33573154	5.000512132	2.591129376	0.011211507	-2.833415488
STAT5A	-0.334312273	2.193681119	-2.590993253	0.011215619	-2.833715014
GPSM3	-0.544742239	3.310835135	-2.590758896	0.011222701	-2.834230662
RP11-960H2.2	0.37537969	0.161918253	2.590742765	0.011223189	-2.834266153
SLC27A1	-0.342813062	2.178333819	-2.5903825	0.011234085	-2.835058754
CEP76	0.202185834	1.365455396	2.589507194	0.011260598	-2.836984083
HNRNPRP2	0.219786494	0.096711112	2.587956888	0.011307695	-2.840392798
RP11-126O1.6	0.248570302	0.109768752	2.58735764	0.011325948	-2.841709926
PIKFYVE	0.365833477	2.145989424	2.587234542	0.011329701	-2.841980459
RIC8B	0.210387843	1.732485135	2.587226582	0.011329943	-2.841997953
RP5-1198O20.5	0.201066536	0.126166842	2.587199788	0.01133076	-2.842056838

HERC2	0.275594719	2.315173247	2.586778813	0.011343604	-2.842981925
AC074121.4	0.273737939	0.166800892	2.586777371	0.011343648	-2.842985093
GYPC	-0.513327024	3.297622211	-2.586317249	0.0113577	-2.843996061
RFC4	0.360883307	3.047261699	2.586101486	0.011364296	-2.844470076
F13A1	-0.85589333	2.66827671	-2.585874376	0.011371241	-2.844968983
PARP3	-0.324878677	2.166129844	-2.584848419	0.011402666	-2.847222316
RN7SKP17	0.31459814	0.10934551	2.584244734	0.011421193	-2.848547849
ZMAT2	-0.356762249	4.813688191	-2.583868925	0.01143274	-2.849372895
CTD-2522E6.4	0.260566702	0.302959105	2.583405858	0.011446983	-2.850389365
WNT5A-AS1	0.4884228	1.514532851	2.58330081	0.011450216	-2.850619933
AC112497.2	0.209252965	0.096972926	2.582957031	0.011460803	-2.851374432
HLA-E	-0.481797745	6.524086665	-2.582748679	0.011467224	-2.851831665
MICAL3	0.261021096	1.043837697	2.5824618	0.01147607	-2.852461177
BX842568.2	0.296962577	0.681799792	2.58244121	0.011476705	-2.852506355
THAP5	0.321672809	2.290334693	2.582409892	0.011477671	-2.852575075
CREBL2	-0.342576788	3.263008526	-2.582367829	0.011478969	-2.852667369
ING4	-0.333776675	2.961868507	-2.581941657	0.011492124	-2.853602398
MIR543	0.336301699	0.120711837	2.581873762	0.011494221	-2.853751349
AC134772.1	0.520705319	0.563264141	2.580969582	0.011522182	-2.855734665
RP11-696L21.2	0.386626039	0.324833585	2.580855174	0.011525724	-2.855985575
RP11-21J7.1	0.22751212	0.099956116	2.58070117	0.011530494	-2.856323311
AP000560.3	0.219100979	0.412135478	2.580322924	0.011542217	-2.857152746
KB-1980E6.2	0.220720524	0.236361928	2.5803222	0.011542239	-2.857154332
RP11-210K20.2	0.431755946	0.35985133	2.580021387	0.01155157	-2.857813895
HHEX	-0.348925211	1.565243858	-2.579614531	0.0115642	-2.858705863
KB-1410C5.2	0.210248528	0.079031226	2.579552021	0.011566142	-2.858842896
FNDC7	0.236538197	0.125581083	2.579222097	0.011576395	-2.859566099
RNU1-108P	0.264410813	0.172709734	2.57914566	0.011578771	-2.859733642
AHCYL2	0.398895299	2.340995459	2.57858231	0.011596301	-2.860968313
RP11-420A6.2	0.210579811	0.359957086	2.57853775	0.011597688	-2.861065964
AC010971.1	0.250297606	0.097371905	2.578215654	0.011607722	-2.861771775
RNU6-168P	0.286206555	0.106225456	2.577387483	0.011633558	-2.863586212
SLC7A7	-0.48491597	2.2074804	-2.576996856	0.011645762	-2.864441864

CTD-2377D24.2	0.261399292	0.124923626	2.576906715	0.01164858	-2.864639298
RPL7P10	0.304737238	0.553689592	2.576830968	0.011650949	-2.864805201
DHRS1	-0.298641578	1.857611653	-2.576825137	0.011651131	-2.864817972
SERBP1P6	0.258722542	0.867547051	2.576363339	0.011665579	-2.865829323
NIP7	-0.301455614	3.307658609	-2.576305981	0.011667375	-2.86595493
AC132068.1	0.301242076	0.168759758	2.576085311	0.011674286	-2.866438139
MIR3913-2	0.211409292	0.067983242	2.57348469	0.011756011	-2.872130195
MIR4748	0.359018921	0.450575677	2.573073079	0.011768993	-2.873030655
CTD-2293H3.2	0.242299386	0.254492366	2.57291865	0.011773867	-2.87336846
CD160	0.226837163	0.395262877	2.572838623	0.011776393	-2.873543508
AC022909.1	0.441078486	0.202398066	2.572471794	0.011787981	-2.874345834
XXbac-BPG154L12.4	0.258487384	0.146372685	2.572229153	0.011795651	-2.874876485
RP11-448A19.1	0.207395132	0.572197462	2.572201172	0.011796535	-2.874937677
AP000289.6	0.209758824	0.074120089	2.57209112	0.011800016	-2.875178343
RN7SL679P	0.321093931	0.155330712	2.57196508	0.011804003	-2.875453961
RNU6-981P	0.396731325	0.257990767	2.571708265	0.011812131	-2.876015515
ZNF680	0.265278472	1.348883501	2.571307373	0.01182483	-2.876892014
CTC-518B2.9	0.204126803	0.075139946	2.570880495	0.011838365	-2.877825202
RP11-78O7.2	-0.332631381	0.998732053	-2.570231221	0.011858978	-2.879244314
NDUFA12	-0.357912331	4.647231995	-2.569940774	0.01186821	-2.879879041
AC027319.1	-0.209565121	0.237724179	-2.569896917	0.011869604	-2.87997488
HSPE1P6	0.313733345	0.509079697	2.569858569	0.011870824	-2.880058678
LINC01358	-0.33662188	0.702734922	-2.569354714	0.011886857	-2.881159604
RN7SL259P	0.232143943	0.094150616	2.568974207	0.011898978	-2.881990894
RP11-1277A3.2	0.249088609	0.583807555	2.568638462	0.011909682	-2.882724306
C3orf70	0.515922436	1.45447919	2.568600284	0.0119109	-2.882807697
RP11-412D9.4	0.314875112	1.207215076	2.567832595	0.011935411	-2.884484336
KMT2D	0.30933382	2.54924386	2.567675032	0.011940447	-2.884828401
GSKIP	-0.268397316	1.962251823	-2.567299071	0.011952472	-2.885649306
RPL21P126	0.214112808	0.08764824	2.567215027	0.011955162	-2.885832801
P4HA1	0.562312117	5.917539045	2.566956846	0.011963427	-2.886396458
RP5-1022P6.4	0.218239196	0.119868738	2.566744202	0.011970239	-2.886860666
CRBN	-0.255747721	1.959061201	-2.566426604	0.011980419	-2.887553928

SEC24A	0.310057747	2.868778762	2.566382391	0.011981837	-2.887650433
CTD-2026J24.1	0.233385613	0.088758432	2.566237025	0.0119865	-2.887967714
CD72	-0.295513709	0.962987216	-2.566039956	0.011992824	-2.88839782
RP11-697M17.2	0.274538072	0.210115473	2.565855275	0.011998752	-2.888800863
AL360074.1	0.347940567	0.558215064	2.565740302	0.012002445	-2.889051764
TMEM39A	0.303449408	3.33891705	2.565482811	0.012010718	-2.889613643
TAF4	0.232986663	1.756148838	2.565382311	0.012013948	-2.889832934
ZNF525	0.269100017	1.401246193	2.565160304	0.012021087	-2.890317329
RP11-151H2.1	0.26773621	0.20804626	2.565066421	0.012024107	-2.890522161
CACFD1	-0.341508254	2.341551294	-2.564989806	0.012026572	-2.890689312
RNA5SP432	0.221003826	0.108525899	2.564687724	0.012036296	-2.891348327
GIGYF2	0.25985434	2.359950188	2.564081199	0.012055841	-2.892671308
CBY1	-0.32240506	2.947382908	-2.564057287	0.012056612	-2.892723461
RP11-479J7.1	0.271458468	0.112948673	2.56391663	0.012061149	-2.893030228
SRGAP2	0.246123633	2.456515087	2.563864556	0.012062829	-2.893143798
MIR491	0.205416876	0.107761673	2.563778753	0.012065598	-2.893330919
GART	0.367059608	3.533224409	2.563511139	0.012074237	-2.893914509
RP1-163M9.7	0.202638486	0.126096049	2.563181008	0.012084902	-2.894634361
KIF11	0.428990327	3.291944111	2.563009354	0.012090451	-2.895008622
U5	0.241285988	0.105135863	2.562779993	0.012097869	-2.895508668
RP11-549L6.3	0.284036163	0.237020911	2.562728563	0.012099533	-2.89562079
DGKE	0.211192143	0.708123296	2.562553958	0.012105183	-2.896001428
RP11-397H6.1	0.234958991	0.137342878	2.562477675	0.012107652	-2.896167718
AC099799.1	0.232614199	0.112295159	2.562215804	0.012116132	-2.89673854
RP11-1023L17.2	0.272601542	0.353766342	2.561078777	0.012153015	-2.899216437
ATL3	0.328484251	4.059114402	2.560695457	0.012165472	-2.900051587
RP11-103J17.1	0.259352698	0.155806755	2.560542516	0.012170446	-2.900384774
RP11-235E17.4	0.233660815	0.3476673	2.560417054	0.012174527	-2.900658086
ALDH1L2	0.49977701	2.784524202	2.560392689	0.01217532	-2.900711163
RP11-90P5.1	0.217779193	0.220979791	2.560250141	0.012179959	-2.901021677
CD300LF	-0.272106124	0.662927937	-2.560172354	0.012182491	-2.901191117
RP11-439C8.1	0.286688435	0.167054843	2.560033048	0.012187026	-2.901494549
HIATL2	0.347998682	2.42803842	2.559711711	0.012197494	-2.90219442

ZNF501	-0.275016442	1.152753583	-2.559648668	0.012199549	-2.902331717
KDM5C-IT1	0.222226918	0.230070905	2.559050835	0.012219049	-2.903633572
PTBP3	0.271100469	3.518441969	2.557940864	0.012255329	-2.906049988
FNTAP2	0.227661207	0.315797549	2.557760705	0.012261227	-2.906442111
MIR550A2	0.288029195	0.250512207	2.557707181	0.012262979	-2.906558603
TMEM209	0.334545533	2.717133081	2.557606983	0.012266261	-2.906776674
RP1-90J20.14	0.224572393	0.082197245	2.557341924	0.012274946	-2.907353515
RP1-63P18.2	0.311307082	0.182143843	2.557322181	0.012275593	-2.907396477
CTC-232P5.1	0.206559737	0.542088075	2.556953856	0.012287671	-2.908197964
AC093680.1	0.341287401	0.111371099	2.555450273	0.012337088	-2.911468787
RP11-739N10.1	0.200856317	0.097460099	2.555155504	0.012346797	-2.912109823
RP11-373I8.1	0.227354447	0.08111343	2.554920647	0.012354538	-2.912620523
RNU6-1086P	0.260627477	0.172973237	2.554766925	0.012359606	-2.912954771
ACVR1C	0.219991342	0.396357761	2.554695447	0.012361964	-2.913110186
CTC-422A18.1	0.298265454	0.168115346	2.553964862	0.012386084	-2.914698479
PVRL3-AS1	0.458461704	0.612507463	2.553783075	0.012392092	-2.915093625
ACP2	-0.285016044	4.001267649	-2.553649154	0.01239652	-2.915384711
RP11-708L7.7	0.227354402	0.196532865	2.55335825	0.012406143	-2.916016964
RN7SKP160	0.314741347	0.465046559	2.553344877	0.012406585	-2.916046027
C10orf54	-0.47878475	2.56002386	-2.552869103	0.012422339	-2.91707994
RN7SL811P	0.211071094	0.10564871	2.552097781	0.012447917	-2.918755769
RFXANK	-0.332937603	3.831945526	-2.551953873	0.012452694	-2.919068387
RP11-436M15.3	0.24056801	0.267466759	2.551428407	0.012470153	-2.920209749
RNU7-105P	0.601270079	0.251560541	2.551206617	0.012477528	-2.92069144
CHRNA7	0.202503965	0.369356885	2.550325271	0.012506876	-2.922605222
TST	-0.39480685	2.666483476	-2.550303951	0.012507586	-2.92265151
RP11-274B21.4	0.5245615	3.321765828	2.54987613	0.012521855	-2.923580286
FAM219B	0.260497059	1.619896609	2.549156806	0.012545879	-2.925141599
PRRX2-AS1	0.229859375	0.399118831	2.5486159	0.012563972	-2.926315405
MRPL35P3	0.239386222	0.346703997	2.548424473	0.012570381	-2.926730763
AC107072.2	0.275308043	0.250897712	2.548374474	0.012572055	-2.926839249
GIMAP7	-0.558855789	2.222837912	-2.547996174	0.01258473	-2.927659995
CXCR3	-0.39807054	0.643486388	-2.547481467	0.012601994	-2.928776522

CCDC15	0.294026144	1.220694254	2.546742774	0.012626808	-2.930378593
RP11-1018N14.1	0.215298875	0.083150966	2.546700043	0.012628245	-2.930471256
RNU1-112P	0.34233788	0.163310051	2.546025865	0.012650932	-2.931933043
C1orf27	0.27901013	2.405424134	2.545955298	0.012653309	-2.93208603
AC087392.1	0.538751717	0.513476268	2.545705163	0.012661737	-2.932628289
RAB26	0.256657744	0.422680971	2.545344293	0.012673905	-2.933410528
RP11-158L12.4	0.235591227	0.646585874	2.545320555	0.012674706	-2.93346198
RNU6-409P	0.262626186	0.187927642	2.54531601	0.01267486	-2.933471832
TBX20	0.427115997	0.636664873	2.545119605	0.012681487	-2.933897524
AC091042.1	0.261053288	0.093971493	2.544765932	0.01269343	-2.934664013
AC144836.1	0.269805118	0.199821176	2.544402374	0.012705716	-2.935451831
FBXO7	-0.249265853	3.459796457	-2.543979686	0.012720015	-2.936367661
TMEM47	0.708988675	3.327123555	2.543907218	0.012722468	-2.936524664
CD209	-0.558585303	1.886414594	-2.543289262	0.012743401	-2.937863314
RNU6-1333P	0.26256789	0.158299354	2.54308402	0.012750361	-2.938307858
ZDHHC4	-0.365195519	3.290250743	-2.542856906	0.012758066	-2.938799742
TRIM26BP	0.219716125	0.192700109	2.54207029	0.012784786	-2.940503106
RP11-185B14.1	0.27266257	0.153605304	2.542027275	0.012786248	-2.940596239
CIITA	-0.289430616	0.91860466	-2.541703739	0.012797254	-2.941296696
RP4-728D4.3	0.230567274	0.18725112	2.541655088	0.012798909	-2.941402017
STK3	0.257358923	1.759872176	2.541574418	0.012801655	-2.941576653
RNU6-696P	0.374859121	0.210371907	2.541427274	0.012806665	-2.941895182
RP4-797C5.2	0.308686502	0.191821611	2.541405261	0.012807415	-2.941942833
HJURP	0.372620315	2.893375058	2.541038824	0.012819899	-2.942735998
TMEM9B	-0.253644158	3.600603865	-2.540480146	0.012838953	-2.94394509
RNU6-42P	0.369362177	0.624933683	2.540355184	0.012843219	-2.944215502
PRR11	0.412380283	4.307453233	2.540345427	0.012843552	-2.944236615
AC005048.1	0.311563311	0.092052796	2.54032926	0.012844104	-2.944271598
TTC26	0.254153475	1.586923549	2.540208228	0.012848237	-2.944533492
C5orf28	0.327071732	2.23861457	2.540012069	0.012854937	-2.944957929
RP11-492D6.3	0.207205123	0.157380803	2.539852825	0.012860379	-2.945302469
RP11-216P16.4	0.206888134	0.129243803	2.539780533	0.012862851	-2.945458875
SENP6	0.298054337	2.493254947	2.539720454	0.012864905	-2.945588854

RN7SKP85	0.256397467	0.147979922	2.539516417	0.012871883	-2.946030262
RNU6-273P	0.392968527	0.205385513	2.539413518	0.012875403	-2.946252859
AC006126.3	0.219589068	0.363440433	2.539178719	0.012883439	-2.946760763
RP5-884C9.3	0.23119367	0.091771993	2.538937108	0.012891714	-2.947283361
LA16c-380H5.3	0.213149299	0.107126808	2.538719867	0.012899157	-2.947753211
GZMH	-0.450117154	0.616367483	-2.538528754	0.012905709	-2.948166522
CCDC12	-0.301384924	2.522816256	-2.538329982	0.012912526	-2.94859637
MTCO2P1	0.264699116	0.162751741	2.538293589	0.012913775	-2.948675067
RP11-96O20.2	0.406411561	0.19621863	2.537990855	0.012924165	-2.94932967
STX8	-0.568204363	2.800410148	-2.53778926	0.012931088	-2.949765544
HSPE1P14	0.269678053	0.183664327	2.537659465	0.012935547	-2.950046162
RP11-206L10.2	0.244403482	0.674698409	2.537497111	0.012941127	-2.950397155
RN7SL156P	0.209225491	0.09244322	2.537299163	0.012947932	-2.950825073
RP11-252I14.1	0.245683997	0.16883674	2.537285775	0.012948393	-2.950854012
RNU7-49P	0.525951433	0.968719052	2.537145537	0.012953217	-2.951157157
CENPE	0.38042243	2.284653982	2.536714066	0.012968068	-2.952089751
RP11-342K2.1	0.296576522	0.893739858	2.536620392	0.012971294	-2.952292202
RAC1	-0.334262914	7.040483861	-2.536078251	0.012989981	-2.953463775
CTD-3032H12.2	0.428062623	0.662970523	2.535862073	0.012997439	-2.953930879
RP11-318G8.2	0.249631738	0.176231546	2.535799312	0.012999605	-2.954066482
RNU6-432P	0.260739719	0.124630862	2.535751577	0.013001253	-2.954169617
RP11-344H11.5	0.333721778	0.171381472	2.535439847	0.013012017	-2.954843099
AC044907.1	0.667158154	1.578774435	2.535439016	0.013012045	-2.954844895
TMEM120B	0.372858986	1.923961019	2.534887744	0.013031101	-2.956035725
RP11-94P14.1	0.220734397	0.244564989	2.534782453	0.013034743	-2.956263145
DOK3	-0.352688668	1.587378995	-2.534636401	0.013039797	-2.956578592
MCM3AP-AS1	0.215278017	0.682555759	2.53454237	0.013043052	-2.956781673
DEFB109P1	0.269192152	0.170161209	2.534484117	0.013045068	-2.956907482
TRBV16	0.325828624	0.122061633	2.534363402	0.013049248	-2.95716818
GSN-AS1	0.322265568	0.908274718	2.534321997	0.013050682	-2.957257595
LINC01486	0.291004213	0.115030612	2.534247996	0.013053246	-2.957417403
RP11-313I2.11	0.307290422	0.11848656	2.533619769	0.013075025	-2.958773908
GATA3	-0.424997049	0.754857019	-2.533487556	0.013079612	-2.959059354

CDC42BPA	0.306228613	2.646988218	2.533483983	0.013079736	-2.959067068
RPL36P20	0.32038548	0.124713885	2.533465397	0.013080381	-2.959107194
RNU6-119P	0.255252874	0.075415622	2.533382337	0.013083264	-2.959286512
KLC3	0.216653914	0.28388894	2.532272268	0.01312185	-2.961682551
HIGD1B	-0.264282234	0.801113302	-2.532137982	0.013126524	-2.961972341
RP11-16E23.3	0.298355399	0.3216269	2.531953545	0.013132947	-2.962370337
RNU6-487P	0.281033182	0.110190917	2.531585205	0.013145783	-2.963165101
RP11-449M6.3	0.244578949	0.085148239	2.531558992	0.013146697	-2.963221657
RNU6-542P	0.273918915	0.123598884	2.531029256	0.013165177	-2.964364483
GNG5P3	0.269169382	0.308968725	2.530794282	0.013173382	-2.964871338
AC142293.3	0.244198075	0.117827102	2.530573958	0.01318108	-2.965346556
RP13-685P2.8	0.267429441	0.152045951	2.529256908	0.013227179	-2.968186576
E2F7	0.440725966	1.779680815	2.528637607	0.013248905	-2.969521571
NBPF8P	0.281168886	1.544665788	2.528391272	0.013257556	-2.970052506
RP11-266K4.9	0.344580786	0.652017828	2.528276615	0.013261585	-2.970299615
BRWD1-IT1	0.200879209	0.257352212	2.528083149	0.013268384	-2.970716551
RNU1-7P	0.336433351	0.119006188	2.528017889	0.013270679	-2.970857188
AL359709.1	0.332735918	0.157507239	2.527990411	0.013271645	-2.970916402
CA1	0.218624309	0.113616205	2.527135129	0.013301749	-2.97275923
ST13P14	0.223298666	0.090007638	2.52707709	0.013303794	-2.972884264
IL18R1	0.365653437	0.742411967	2.526966419	0.013307695	-2.973122676
SIPA1L3	0.285098637	2.338260835	2.52645395	0.01332577	-2.974226549
FUBP3	0.300767243	3.45009506	2.526400822	0.013327645	-2.974340978
LPPR3	-0.68936439	1.065783444	-2.525934089	0.013344128	-2.975346148
CXCR6	-0.22494526	0.311184576	-2.525914267	0.013344828	-2.975388835
RP11-98D18.7	0.285824329	0.133499315	2.52584381	0.013347318	-2.975540558
BLNK	-0.27680769	0.79198972	-2.525528127	0.013358479	-2.976220313
ALPK2	0.298635817	0.535780749	2.525502907	0.013359371	-2.976274615
RP11-315O6.2	0.2549162	0.091851549	2.525457184	0.013360989	-2.976373063
CBX3P1	0.35833425	0.520862992	2.525252208	0.013368242	-2.976814384
SYNJ1	0.296128561	1.751515331	2.52473309	0.013386626	-2.977931932
POLE2	0.367931942	2.009760342	2.524245063	0.01340393	-2.97898237
RP11-285C1.2	0.25156096	0.1292868	2.524080349	0.013409774	-2.979336862

VHL	-0.321450529	4.041314444	-2.523658495	0.013424754	-2.980244678
RP11-662J14.2	0.204683797	0.113715783	2.523469861	0.013431457	-2.980650569
FAM64A	0.448022025	2.286162187	2.523182036	0.013441691	-2.981269847
ATAD2B	0.213689239	1.413820907	2.523161641	0.013442416	-2.981313725
RNU6-623P	0.295174026	0.463271962	2.523021852	0.013447389	-2.981614467
SLC16A8	0.642484109	1.344080541	2.522889997	0.013452082	-2.981898128
RP11-390F4.3	0.262682101	0.361390172	2.522339877	0.013471674	-2.983081466
RP11-887P2.6	0.206283388	0.181698344	2.522012575	0.013483343	-2.983785407
PITPNM3	0.310969902	0.628391429	2.521994623	0.013483984	-2.983824014
RP11-781P14.3	0.357186277	0.176022687	2.52168728	0.01349495	-2.984484954
AC069542.1	0.250930406	0.180252686	2.521455611	0.013503221	-2.98498311
RP11-463C8.5	0.260232371	0.105925017	2.521391122	0.013505525	-2.985121775
ST3GAL4-AS1	0.210650705	1.187966993	2.52135319	0.01350688	-2.985203334
RNU7-14P	0.355112473	0.12803684	2.521171808	0.013513361	-2.985593319
RP11-672A2.7	0.241429321	0.160454178	2.521010681	0.01351912	-2.985939734
CTRC	0.306542528	0.152231283	2.520635575	0.013532536	-2.98674612
RP3-477M7.5	0.201046203	0.270771672	2.520579454	0.013534545	-2.986866758
GPR180	0.364916667	2.102293998	2.520545553	0.013535758	-2.986939629
ARRDC2	-0.262269969	2.721353778	-2.520209112	0.013547804	-2.987662786
RP13-643D4.1	0.200409588	0.076762951	2.519938326	0.013557507	-2.988244763
RP3-399J4.4	0.261656113	0.100451681	2.519700042	0.01356605	-2.98875684
RP11-479O9.2	0.241650623	0.371827995	2.519498193	0.013573291	-2.989190587
NFE2L3	-0.38904919	1.327830695	-2.519308996	0.013580081	-2.98959712
LRRC37A14P	0.334306936	0.237904276	2.518640729	0.013604088	-2.991032832
TMEM65	0.397175853	3.050614064	2.517954909	0.013628766	-2.99250592
TMCO3	0.457060356	2.652018667	2.517913857	0.013630244	-2.992594085
CTD-2130O13.1	0.200632979	0.084177846	2.516898527	0.013666856	-2.994774276
DOCK2	-0.291299516	0.954151982	-2.516714839	0.013673489	-2.995168624
HLX-AS1	0.24614549	0.297357339	2.516034551	0.013698079	-2.996628878
RP11-250B2.2	0.220300154	0.310235144	2.515872223	0.013703953	-2.99697727
RP11-665C16.5	0.238867133	0.242013796	2.515663445	0.01371151	-2.997425325
RP11-514F8.2	0.213118725	0.08108465	2.515262758	0.013726025	-2.998285144
RP11-658F2.8	0.28576931	1.598497004	2.51473892	0.013745023	-2.999409052

AC006373.1	0.687738665	0.919572169	2.51435516	0.013758955	-3.000232291
ARHGAP42	0.3596512	1.881660836	2.51435349	0.013759015	-3.000235873
RNU6-133P	0.295646692	0.105436407	2.514266946	0.013762159	-3.000421513
WI2-87327B8.2	0.286889509	0.397920874	2.514083725	0.013768817	-3.000814507
IDI2-AS1	0.244126703	0.25893995	2.513714911	0.013782227	-3.001605513
SAMM50	-0.26325521	2.851042275	-2.513549796	0.013788234	-3.001959606
CICP14	0.358127726	1.985992446	2.513273317	0.013798299	-3.002552481
TRAJ39	0.542868084	0.377122985	2.5130705	0.013805686	-3.00298736
RP11-104E19.1	0.236397549	0.207998453	2.512732333	0.013818011	-3.003712389
TRIM36-IT1	0.215116171	0.099318596	2.512184205	0.013838009	-3.004887398
RP11-137F15.1	0.206960488	0.131565875	2.512144376	0.013839463	-3.00497277
GAS7	-0.524173685	1.642729848	-2.511312824	0.013869854	-3.006754908
EEF1B2P2	0.376369824	0.917081074	2.511130073	0.013876541	-3.007146503
CTC-482H14.5	0.214272193	0.24825706	2.511124657	0.01387674	-3.007158108
RP11-274B21.2	0.446273426	2.660972077	2.51091416	0.013884446	-3.007609125
RP11-23D5.1	0.246982149	0.089879678	2.509979827	0.013918697	-3.009610666
RNU6-818P	0.327385152	0.149351837	2.509624582	0.01393174	-3.01037151
RP11-693L9.2	0.271888903	0.128261965	2.50935044	0.013941812	-3.01095859
RNU6-1040P	0.314249444	0.155651846	2.509316063	0.013943076	-3.011032206
CH17-140K24.1	0.256335618	0.09628832	2.509143968	0.013949403	-3.011400719
RP11-365O16.3	0.217528134	0.289619956	2.509096865	0.013951135	-3.011501577
RASSF4	-0.489300557	1.967648391	-2.508868379	0.01395954	-3.0119908
CTD-2623N2.5	0.220753074	0.167917901	2.508859158	0.013959879	-3.012010542
NCRUPAR_1	0.258690996	0.135544125	2.508614271	0.013968893	-3.012534839
ZBTB6	0.278512996	2.065934655	2.508503421	0.013972975	-3.012772151
RP1-17K7.2	0.218558573	0.104114215	2.508299345	0.013980492	-3.01320902
RNU6-24P	0.268291296	0.211997263	2.508020942	0.013990754	-3.013804957
POLI	0.21599788	1.152139853	2.507947172	0.013993474	-3.013962857
AL359546.1	0.334659194	0.14674642	2.507358278	0.014015205	-3.015223197
HMGA1P5	0.322274963	0.209768366	2.506775868	0.014036727	-3.01646941
RP11-515C16.7	0.22519018	0.095510976	2.506678054	0.014040345	-3.016678685
TCEA3	0.578920859	3.935227015	2.506638168	0.01404182	-3.016764019
RNU6-1250P	0.235827433	0.100415799	2.506311831	0.014053896	-3.01746216

ARHGEF17	0.368584884	3.398257526	2.505970362	0.014066542	-3.01819259
SUCLG1	-0.300828698	3.859012306	-2.505279477	0.01409216	-3.019670187
RN7SL697P	0.228605587	0.147749786	2.505105781	0.014098607	-3.020041618
CTC-246B18.8	0.289024687	0.750544415	2.504849206	0.014108135	-3.020590236
RP13-488H8.1	0.262105639	0.124955684	2.504784313	0.014110546	-3.020728985
ALG9	0.217675999	1.381886207	2.504630639	0.014116256	-3.021057545
COMMD3	-0.294737524	2.328448007	-2.504327992	0.014127509	-3.021704566
SIGLEC14	-0.372158855	0.869485555	-2.504281949	0.014129221	-3.021802994
CYCSP27	0.22336794	0.235521697	2.503643482	0.014152989	-3.023167714
HMGN1P8	0.310829321	0.308417851	2.503577251	0.014155456	-3.023309266
MIR516A2	0.248245805	0.077123675	2.503329627	0.014164685	-3.023838467
CHM	0.264949416	1.826929865	2.503268404	0.014166968	-3.023969303
WASF2	0.31752336	5.603640125	2.503090169	0.014173615	-3.024350177
RP11-407A16.3	0.227859537	0.186066525	2.502774204	0.014185405	-3.025025312
RP11-809N15.2	0.239293675	0.227258071	2.502572038	0.014192954	-3.025457252
RP11-215P8.1	0.264972987	0.549001053	2.502498198	0.014195712	-3.025615008
RP11-57C13.3	0.205223611	0.07882492	2.502305161	0.014202925	-3.026027405
TES	-0.573084241	2.563170742	-2.502028437	0.014213269	-3.026618538
GIMAP6	-0.42829146	2.007266232	-2.501862714	0.014219468	-3.026972527
RP11-306K13.1	0.248899	0.10041168	2.501850825	0.014219913	-3.026997922
RNA5SP87	0.301078135	0.156137475	2.501849624	0.014219958	-3.027000486
RP11-96C23.12	0.218399077	0.094563508	2.501504776	0.014232864	-3.027737024
AC005162.5	0.232732493	0.119001602	2.501324948	0.014239599	-3.028121071
LAMTOR4	-0.38174522	4.561093852	-2.500753094	0.014261034	-3.029342189
RNU4ATAC3P	0.246171814	0.086755552	2.500563759	0.014268137	-3.029746436
SPICE1	0.327922604	1.463423097	2.500098137	0.014285619	-3.030740468
RNU6-315P	0.234292079	0.359103621	2.499944506	0.014291392	-3.031068413
RNU7-9P	0.246457343	0.122370485	2.499338574	0.014314179	-3.03236168
HEBP1	-0.248659489	3.249496289	-2.499298318	0.014315694	-3.032447591
RP11-354P11.8	0.221814962	0.348952953	2.498887573	0.014331161	-3.033324099
C18orf25	0.262305618	1.716265024	2.498810869	0.014334051	-3.033487767
RNU6ATAC4P	0.280867311	0.189638534	2.498765574	0.014335758	-3.033584415
LAG3	-0.412948046	0.960561096	-2.498223007	0.014356218	-3.034741987

MYO1G	-0.232258733	0.553695795	-2.497865594	0.01436971	-3.035504415
AC005682.5	0.321315825	1.0207386	2.497695917	0.014376119	-3.035866333
RNY4P17	0.435587154	0.262194077	2.497590617	0.014380098	-3.036090926
BNIP3P16	0.248349956	0.350973198	2.49741357	0.01438679	-3.036468529
RN7SKP75	0.256751535	0.129737877	2.496671989	0.01441485	-3.038049916
CTD-3035D6.2	0.329585159	0.686027478	2.496244868	0.014431034	-3.038960549
CTC-575C13.3	0.365995329	0.208156425	2.495542475	0.014457683	-3.040457779
MIR559	0.232531579	0.119078248	2.495465026	0.014460624	-3.04062285
CCDC28B	-0.284690756	2.134384996	-2.495289701	0.014467284	-3.040996509
RPL23P4	0.256515583	0.121328054	2.49514378	0.014472829	-3.041307483
AC098680.1	0.243648344	0.075981922	2.494779323	0.014486688	-3.042084118
TERT	0.231385041	0.148814883	2.494678114	0.014490538	-3.04229977
TMEM150B	-0.354233199	1.00981511	-2.494384645	0.014501708	-3.04292504
RP11-1074O12.1	0.226394628	0.156571717	2.494286394	0.01450545	-3.043134361
NDUFA12P1	0.269323292	0.109966813	2.493294755	0.014543261	-3.045246633
RP11-701B6.1	0.240468031	0.13052959	2.492944857	0.014556623	-3.045991771
USP32P3	0.796277421	1.821518814	2.492863093	0.014559747	-3.046165881
MINOS1	-0.294519098	2.718690781	-2.492764826	0.014563503	-3.046375128
RP11-382D8.3	0.207068445	0.35542785	2.492391802	0.014577766	-3.047169369
PARD3-AS1	0.275759568	0.48739143	2.492125418	0.01458796	-3.047736492
RP11-617O8.1	0.226408476	0.071231587	2.491546353	0.014610141	-3.048969119
P3H2-AS1	0.272012345	0.290609647	2.491412912	0.014615257	-3.049253134
RNU5E-5P	0.20423207	0.089615796	2.491280068	0.014620352	-3.049535866
CHCHD3P3	0.212101775	0.64675183	2.491118995	0.014626531	-3.049878658
RSF1	0.303896976	2.211253502	2.490935485	0.014633573	-3.05026918
RARRES2P3	0.300216496	0.138089384	2.490819725	0.014638018	-3.050515513
ZC3H11A	0.287000246	3.770943212	2.490283589	0.014658617	-3.051656256
AP002954.3	0.214556648	0.498890022	2.490262514	0.014659427	-3.051701094
RP11-72L22.1	0.286995647	0.143018894	2.489764546	0.014678584	-3.052760432
RNU6-959P	0.228282815	0.127121122	2.489636477	0.014683514	-3.053032846
RP11-138E2.1	0.419058606	0.289503033	2.489445633	0.014690864	-3.053438769
RP5-1186P10.1	0.21863286	0.123086695	2.489009927	0.014707656	-3.054365406
RPL23AP82	-0.276921483	1.475067084	-2.48890172	0.014711829	-3.054595513

RNU4-45P	0.326691853	0.149978689	2.488654241	0.014721378	-3.055121758
RP11-667M19.10	0.28829266	0.48866239	2.488529574	0.014726189	-3.055386834
C21orf58	0.393871123	1.66278032	2.48846949	0.014728509	-3.055514586
ZNF30	-0.283871623	1.313077541	-2.488208069	0.014738606	-3.056070394
RPL21P111	0.245981019	0.17696814	2.488115763	0.014742172	-3.056266632
PEX16	-0.322450769	2.661608807	-2.488115317	0.014742189	-3.056267581
TMCO1	0.340747291	4.69238094	2.487962107	0.014748111	-3.056593288
AC074338.5	0.372741535	0.160039639	2.487819817	0.014753612	-3.056895764
RP11-416H1.1	0.346950213	0.1966709	2.487551019	0.014764009	-3.057467126
CTSD	-0.446233538	8.031544749	-2.486729623	0.014795822	-3.059212777
ATP6V1E1	-0.411368463	5.010793218	-2.486357356	0.014810261	-3.060003766
RP11-25I15.2	0.203384896	0.082145737	2.486237714	0.014814903	-3.060257957
RNU6-129P	0.255252181	0.075415417	2.486165256	0.014817716	-3.060411897
RNU6-369P	0.236751711	0.105783092	2.485751986	0.014833767	-3.061289832
TTK	0.347625588	2.376644396	2.485196931	0.014855348	-3.062468774
AL158086.1	0.326557193	0.110070638	2.484648649	0.014876694	-3.063633107
DAP3P1	0.294329134	0.37516544	2.48457027	0.014879748	-3.063799535
NDC80	0.378791541	3.021473401	2.48456113	0.014880104	-3.063818942
AL591610.1	0.34558781	0.118196428	2.484349598	0.014888349	-3.06426808
MIR577	0.274939312	0.136245797	2.484238216	0.014892692	-3.06450456
RP1-206D15.6	0.229505801	0.553652362	2.484207558	0.014893887	-3.064569649
OTC	0.204605142	0.090615429	2.484117256	0.014897409	-3.064761364
RNA5SP349	0.213452038	0.063065375	2.483828163	0.014908689	-3.065375083
RP11-351O1.4	0.214637426	0.242297118	2.48344367	0.014923704	-3.066191231
MIR4293	0.233573093	0.081379851	2.482945477	0.014943179	-3.067248567
BTAF1	0.326466997	2.307142452	2.48277595	0.014949811	-3.067608319
CTD-2102P23.1	0.317651207	0.717794575	2.482658309	0.014954415	-3.067857952
LINC01273	0.306086935	0.702906219	2.482459085	0.014962214	-3.068280681
MRPS21P9	0.293070237	0.242895093	2.482198843	0.014972408	-3.068832838
CLEC2D	-0.234913665	0.952022805	-2.48193706	0.014982668	-3.069388215
CTD-2659N19.4	0.38490608	0.579727418	2.481802784	0.014987934	-3.069673065
RP11-554D14.5	0.213333548	0.0783185	2.481721864	0.014991107	-3.06984472
RP11-158M2.3	0.291628999	0.459441763	2.481281111	0.015008405	-3.070779602

RP11-223J15.2	0.215921756	0.083770443	2.480876396	0.015024305	-3.071637918
EMC2	-0.332130873	2.993818868	-2.480842672	0.01502563	-3.071709435
RP11-569G9.7	0.295684953	0.140653028	2.480732315	0.015029969	-3.071943457
RP11-767N6.7	-0.317339276	2.098262547	-2.480567045	0.015036468	-3.072293906
MIR5003	0.241530817	0.186335796	2.480489046	0.015039536	-3.072459296
TRBV28	-0.499947044	0.710694005	-2.48035578	0.015044779	-3.072741862
RPL19P13	0.395570449	0.186680735	2.480103812	0.015054698	-3.073276078
RP11-497D6.3	0.341904022	0.18056959	2.479885801	0.015063284	-3.07373826
LIMK1	-0.346571639	3.321065489	-2.479825464	0.015065661	-3.073866167
PBDC1	-0.396372205	3.878737531	-2.479396032	0.01508259	-3.074776444
RNA5SP33	0.270406166	0.24017756	2.479385366	0.01508301	-3.074799052
SNORA13	-0.315686052	0.550777592	-2.479256545	0.015088092	-3.075072089
TRIM61	0.346607174	0.607790041	2.479093879	0.015094511	-3.075416842
AC078785.1	0.272108437	0.146446053	2.478856335	0.015103889	-3.075920258
RNU6-332P	0.2587776	0.119188555	2.478750521	0.015108068	-3.076144492
SMUG1	-0.285209183	2.431828066	-2.478544493	0.015116208	-3.076581069
RP4-566D2.1	0.338888	0.196021918	2.477597498	0.015153674	-3.078587365
RPS23P7	0.21705326	0.187399242	2.477584072	0.015154206	-3.078615805
RP11-274B21.3	0.478330772	3.043972041	2.476732605	0.015187965	-3.080419147
DMAP1	-0.250658026	2.711977541	-2.476618456	0.015192496	-3.080660866
VTCN1	0.205274779	0.200429872	2.476534686	0.015195822	-3.080838249
CTD-2639E6.4	-0.256467378	0.606026418	-2.476466682	0.015198522	-3.080982243
RP11-45P15.2	0.213601477	0.088708171	2.476035369	0.015215659	-3.081895444
ZNF32	-0.370996777	3.67047832	-2.475438738	0.015239393	-3.083158444
UNC13D	-0.254156893	0.869901664	-2.475359475	0.015242548	-3.083326214
TSPO	-0.484805182	5.641656738	-2.474927197	0.015259768	-3.084241108
RP11-108P20.4	0.337241356	0.137913641	2.474723184	0.015267901	-3.084672845
RNU6-752P	0.322843444	0.13154832	2.474618848	0.015272062	-3.08489363
RNU4-21P	0.327064736	0.411974311	2.474587353	0.015273318	-3.084960277
RP11-666A20.4	0.312196508	0.558721389	2.474564437	0.015274232	-3.085008767
RP11-47F1.1	0.29540634	0.354344325	2.473641722	0.015311078	-3.086960944
AC010987.5	0.321107857	0.153989334	2.473569947	0.015313948	-3.08711277
RP11-1348G14.8	0.207774963	0.823094778	2.472656909	0.015350492	-3.089043811

RP11-96J19.1	0.20151525	0.200613265	2.47256868	0.015354027	-3.089230381
AC012360.1	0.274022874	0.197839736	2.47251345	0.015356241	-3.089347167
RP11-287F9.2	0.225329004	0.250031561	2.472432537	0.015359484	-3.089518257
SESN1	-0.253693107	1.947415401	-2.472332608	0.01536349	-3.08972955
RP11-138H8.4	0.261714229	0.124979523	2.47177102	0.015386024	-3.09091685
ABCB10	0.28653252	2.089917313	2.471028208	0.015415874	-3.092486937
PILRB	0.224324839	0.829501163	2.470993125	0.015417285	-3.092561083
ATL1	0.365527486	1.072836654	2.470703247	0.015428949	-3.093173684
RP1-63M2.5	0.445450455	0.308577519	2.47037016	0.015442361	-3.093877521
CLEC5A	-0.412533392	0.954083385	-2.470335249	0.015443767	-3.093951286
RPL4P3	0.290039646	0.923204062	2.470181572	0.015449959	-3.094275985
RP11-884K10.7	-0.237102966	1.415952811	-2.470077387	0.015454158	-3.094496106
RNU6-927P	0.356655021	0.21204017	2.469905585	0.015461085	-3.094859066
SLC44A1	0.312619974	3.536324294	2.469814347	0.015464765	-3.095051815
RPS26P4	0.284847495	0.174078021	2.469677525	0.015470284	-3.095340851
TMEM173	-0.318436579	3.729284353	-2.468871276	0.015502845	-3.097043769
SNORD30	0.242991854	0.201756882	2.468780832	0.015506501	-3.09723477
AC068489.1	0.270354774	0.116821712	2.46865931	0.015511415	-3.097491394
ESPL1	0.309932463	1.797773285	2.468398185	0.015521979	-3.098042787
HIGD1AP17	0.369630287	0.261877317	2.467269744	0.015567705	-3.10042503
RPL21P71	0.247844388	0.213720795	2.466910006	0.015582307	-3.101184275
NAP1L5	-0.433390505	2.240262398	-2.466438512	0.015601464	-3.102179242
RUSC1-AS1	0.287308629	1.39320687	2.466425716	0.015601984	-3.102206242
BRIP1	0.299314003	1.37208961	2.465784376	0.015628077	-3.103559358
NUMA1	0.258622209	3.424619573	2.465719235	0.01563073	-3.103696778
MFAP3	0.335752952	2.243351378	2.465690657	0.015631894	-3.103757063
NUP210P3	0.289056195	0.241510539	2.465507958	0.015639335	-3.104142459
RP11-5A11.1	0.262060704	0.182627428	2.46512987	0.015654746	-3.104939941
AP000719.1	0.320575465	0.33353608	2.465004782	0.015659848	-3.10520376
RNF6P1	0.206512697	0.087718682	2.464778964	0.015669061	-3.105679995
RP13-104F24.3	-0.436368134	2.255243563	-2.464698414	0.015672349	-3.105849861
RNU6-1097P	0.20222014	0.066445666	2.464285828	0.015689198	-3.106719857
IRS1	0.431062604	2.82676297	2.463889942	0.01570538	-3.107554523

MIR554	0.45365248	0.273860898	2.463467055	0.015722683	-3.108445988
RP11-80I15.1	0.287250623	0.841558385	2.463000779	0.015741781	-3.109428766
ZNF835	0.298936947	0.598121213	2.462861646	0.015747484	-3.10972199
CYCSP24	0.270426292	0.689919922	2.46282923	0.015748812	-3.109790305
RP1-191L6.2	0.249220889	0.115980281	2.462429162	0.015765222	-3.110633357
AC005077.7	0.226162478	0.199030204	2.462254519	0.01577239	-3.111001342
MIR190B	0.365920435	0.132920574	2.461956005	0.015784648	-3.111630278
RNU6-1183P	0.355014347	0.16968944	2.461701903	0.01579509	-3.112165591
RNA5SP143	0.348698584	0.153218151	2.461189519	0.015816164	-3.113244881
RP5-930J4.5	0.305942382	0.152044666	2.461185621	0.015816324	-3.113253092
RP3-329A5.1	0.271866528	0.253986401	2.461076271	0.015820825	-3.113483402
DHRS3	-0.629175912	3.402302109	-2.460849003	0.015830183	-3.113962041
RNU6-317P	0.218637998	0.094569725	2.46039061	0.015849072	-3.114927324
YAP1	0.511789142	4.114782775	2.460024487	0.015864174	-3.115698196
AP001464.4	0.277405558	0.117160402	2.460023939	0.015864197	-3.11569935
AP003064.1	0.331753815	0.28215099	2.45993856	0.01586772	-3.115879099
CTD-2542C24.9	0.303832574	0.344414956	2.459868324	0.015870619	-3.116026966
TMEM199	-0.250071291	1.75008417	-2.459365776	0.015891377	-3.117084863
CTD-3007L5.1	0.331422077	0.147049033	2.45921902	0.015897443	-3.117393758
RP5-1021I20.6	0.2460414	0.225655117	2.458810162	0.015914354	-3.11825425
CTA-212A2.2	0.239194813	0.100697721	2.45866535	0.015920348	-3.118558995
AC025016.1	0.292600833	0.260140332	2.45823669	0.015938102	-3.119460986
RP4-539M6.18	0.258313406	0.106201798	2.457555973	0.015966331	-3.120893079
RNU7-12P	0.536233193	0.660146283	2.457356543	0.01597461	-3.121312574
RNU6-428P	0.339115774	0.187571811	2.457352904	0.015974761	-3.121320228
RP11-218E20.2	0.242481125	0.117605092	2.457146936	0.015983315	-3.121753446
CCL5	-0.791144578	2.625582564	-2.456847537	0.015995757	-3.122383124
RP11-510N19.2	0.482776098	0.33597164	2.456687863	0.016002396	-3.122718915
HSP90B3P	0.217128525	0.979807821	2.456574253	0.016007122	-3.122957823
LL22NC03-86D4.1	0.275715386	0.101431147	2.456348662	0.016016508	-3.123432183
ZNF512B	0.420567306	3.126813129	2.456346995	0.016016577	-3.123435687
NME2	-0.361070322	1.581365107	-2.455956932	0.016032819	-3.124255803
RP11-626P14.1	0.242546249	0.116133842	2.455832825	0.01603799	-3.124516716

MED9	-0.539687246	2.516045225	-2.455763683	0.016040871	-3.12466207
AP000721.1	0.369726489	0.28602172	2.454513617	0.016093045	-3.127289427
RP11-318I4.2	0.215921566	0.100252097	2.454459704	0.016095298	-3.127402716
RP11-140I24.2	0.260816577	0.094868863	2.453828748	0.016121693	-3.12872838
RP11-382A18.3	0.256876207	0.125921202	2.453537578	0.016133887	-3.129340044
RP11-2C15.1	0.252959334	0.103583374	2.453387585	0.016140172	-3.129655111
AC019198.1	0.473966157	0.174055857	2.453188633	0.016148511	-3.130072993
PEPD	-0.38975166	3.81671206	-2.4531288	0.01615102	-3.130198661
ARID3B	0.320183965	1.111357522	2.452697009	0.016169134	-3.131105481
EIF1P6	0.364999114	0.862012915	2.452677096	0.01616997	-3.131147297
HIST1H2AG	0.422834006	1.49863964	2.452008498	0.016198057	-3.132551166
WNT16	0.621818565	0.898223967	2.451601758	0.016215165	-3.133405044
CTD-2194L12.2	0.248091522	0.101825185	2.451323547	0.016226876	-3.133989031
UBE2F	-0.294690759	2.471816743	-2.451117058	0.016235573	-3.134422429
RP11-313F23.4	0.356920119	0.470845078	2.451059172	0.016238012	-3.134543919
RP11-227P3.1	0.20837978	0.094399947	2.450520816	0.016260709	-3.1356737
RNA5SP203	0.328497873	0.410923171	2.45045049	0.016263676	-3.135821269
AMELX	0.728789293	0.33616525	2.450433276	0.016264402	-3.13585739
RP11-297L6.1	0.207229383	0.07061815	2.450144113	0.016276608	-3.136464116
RP11-805F19.2	0.23330761	0.094562313	2.449850158	0.016289024	-3.13708083
TNFSF11	0.560102057	1.649004434	2.449352711	0.016310054	-3.138124326
LAMTOR3	-0.233211193	2.910966558	-2.449341935	0.01631051	-3.138146929
RP11-644F5.16	0.350160619	0.142897332	2.449156811	0.016318342	-3.138535217
RP11-21M24.3	0.213930929	0.419977129	2.44908772	0.016321267	-3.138680126
CCDC34P1	0.23563931	0.114484006	2.448935258	0.016327721	-3.138999878
PLCG2	-0.274245987	1.047989546	-2.448537841	0.016344556	-3.13983329
MIR30C1	0.2420841	0.110519101	2.448220577	0.016358007	-3.140498529
XXyac-YX155B6.5	0.214554804	0.116169503	2.448089263	0.016363577	-3.140773847
RP4-560B9.5	0.261336963	0.113390045	2.447865695	0.016373064	-3.141242559
ZNF462	0.333694091	2.087736649	2.447481911	0.016389362	-3.14204708
RNU6-662P	0.477334204	0.3088885	2.447324482	0.016396051	-3.142377065
RRP7A	-0.320598494	3.673256273	-2.446688785	0.016423089	-3.143709355
CTD-2210P15.2	0.229042053	0.399006419	2.44650369	0.016430968	-3.144097218

RP11-259P15.4	0.27372841	0.600508924	2.445916241	0.016455999	-3.145328046
RP11-356B19.11	0.279018514	0.716390553	2.445837323	0.016459365	-3.145493377
RP11-536C10.1	0.227659812	0.364536061	2.445828203	0.016459754	-3.145512482
RP11-32P22.1	0.246044201	0.168397578	2.445233907	0.016485116	-3.146757358
OXCT1	0.47395432	4.220567049	2.444499093	0.016516524	-3.14829622
AGBL4-IT1	0.237552759	0.094968803	2.4444291	0.016519519	-3.148442779
MTND4P23	0.277975926	0.13729709	2.444045693	0.016535931	-3.149245539
RC3H1	0.259320564	2.136378349	2.443979636	0.01653876	-3.149383836
RN7SL825P	0.276736054	0.174868784	2.443540984	0.016557557	-3.150302111
AC099759.1	0.264986657	0.130357293	2.443373062	0.016564758	-3.150653603
RP11-257I8.1	0.202101446	0.162221412	2.443112542	0.016575935	-3.151198877
DOT1L	0.35607798	2.762021705	2.443009041	0.016580377	-3.151415492
RP11-561B11.3	0.204316453	0.148673798	2.44300238	0.016580663	-3.151429434
RIC1	0.316878738	2.09723268	2.442382525	0.016607291	-3.152726553
ATM	0.298891557	1.998691118	2.442290087	0.016611265	-3.152919965
C10orf25	-0.267537827	0.888936008	-2.442265762	0.016612311	-3.152970861
RP11-20I20.2	0.244341392	0.307218459	2.442257648	0.01661266	-3.152987838
RP11-639F1.2	0.223170667	0.080973433	2.44222343	0.016614132	-3.153059433
SLK	0.3336594	3.074768136	2.442160302	0.016616847	-3.153191512
RP4-799D16.1	0.242035352	0.103089963	2.442136052	0.01661789	-3.153242248
DLG5	0.349656093	2.079546559	2.442121782	0.016618503	-3.153272102
RP11-181C21.4	0.216357684	0.839052796	2.442077908	0.016620391	-3.153363895
SPIRE1	0.286535612	2.158397026	2.441855472	0.016629962	-3.153829249
SMC5-AS1	0.217422987	0.643719266	2.44161442	0.01664034	-3.154333509
SNORA7B	-0.339601881	0.694657295	-2.441514006	0.016644665	-3.154543554
GNA15	-0.573417009	2.277575732	-2.441341067	0.016652116	-3.154905289
VN1R8P	0.221777039	0.098685955	2.441179178	0.016659094	-3.155243889
METTL11B	0.333975889	0.637101106	2.441162922	0.016659794	-3.155277888
RP1-266L20.4	0.246188905	0.112638381	2.440962825	0.016668423	-3.155696375
RP11-543F8.2	0.391423505	0.158169579	2.440880419	0.016671977	-3.155868712
INPPL1	0.274073368	4.885847682	2.440777448	0.01667642	-3.15608405
MRPL34	-0.282694466	4.05742323	-2.440623834	0.016683049	-3.156405278
TADA3	-0.308671649	3.761201113	-2.440567236	0.016685492	-3.156523629

RP11-68E19.2	0.201668409	0.12679585	2.440499294	0.016688426	-3.156665698
LARP1B	0.216562473	1.575784083	2.440483773	0.016689096	-3.156698153
RP11-334L9.1	0.345302546	1.173555529	2.44039248	0.016693038	-3.156889043
C1orf131	0.24061003	1.579553152	2.440213657	0.016700763	-3.157262936
SLC52A1	0.215899934	0.232011148	2.440142518	0.016703836	-3.157411669
CTD-2521M24.9	-0.252746347	0.864960972	-2.440092267	0.016706008	-3.15751673
ZBTB7B	0.353369928	3.349788368	2.440080131	0.016706532	-3.157542103
AC144634.1	0.239124531	0.111466666	2.440036362	0.016708424	-3.15763361
CCL3	-0.446290705	1.43470279	-2.439979574	0.016710879	-3.157752333
LIAS	-0.253665921	2.103719094	-2.439665757	0.01672445	-3.15840837
GGA1	-0.245944726	2.582692265	-2.439343824	0.016738381	-3.159081295
ZNF600	0.209673367	1.006854395	2.439293005	0.016740582	-3.159187514
CTD-2282P23.1	0.200207449	0.227650078	2.438790416	0.016762355	-3.160237889
PRKCI	0.306244134	2.505429812	2.438789186	0.016762408	-3.160240459
DEFB123	0.2471761	0.094081015	2.438538402	0.016773282	-3.160764508
ATP8A2P1	0.268695342	0.11912434	2.438486117	0.01677555	-3.160873761
CTD-2128F4.1	0.300351239	0.097956873	2.438431792	0.016777907	-3.160987272
RP11-44N21.3	0.218783402	0.161249628	2.43827949	0.016784515	-3.161305495
RP11-63G10.2	0.30323075	0.149947863	2.437990864	0.016797045	-3.16190851
AC131263.1	0.487219939	2.63681034	2.43789915	0.016801029	-3.162100111
MIR5739	0.381525838	0.244760767	2.43736641	0.016824183	-3.163212945
MIR4322	0.277447912	0.339637448	2.437208852	0.016831037	-3.163542026
DNAJB5-AS1	0.340309271	0.214691442	2.437124019	0.016834728	-3.163719203
RNA5SP329	0.21384508	0.108957447	2.436984153	0.016840815	-3.164011308
RN7SL447P	0.221211589	0.103187834	2.436487591	0.016862442	-3.165048242
RP11-680B3.2	0.234497559	0.137956045	2.436359033	0.016868045	-3.165316672
RNU6-961P	0.257869393	0.109504562	2.436017142	0.016882954	-3.16603048
RP11-392P7.1	0.427246723	1.510450635	2.435845504	0.016890444	-3.166388797
PUS7	0.304066945	2.699495049	2.435837216	0.016890805	-3.1664061
GRTP1-AS1	0.318401252	0.375178025	2.435681499	0.016897603	-3.166731158
ZNF367	0.40331363	2.317275428	2.435638349	0.016899487	-3.166821232
AL135745.1	0.251695708	0.388940356	2.435595165	0.016901372	-3.166911375
RP11-219H23.1	0.22663929	0.08898012	2.434882867	0.016932503	-3.168398027

ANKRD42	0.218117761	1.244129022	2.434735903	0.016938932	-3.168704714
LOXL4	0.722023628	2.475893056	2.434686005	0.016941115	-3.168808837
ARHGEF12	0.315471246	2.864793609	2.434449864	0.016951452	-3.169301575
PSMD8P1	0.202362758	0.514622528	2.434431805	0.016952242	-3.169339255
FAN1	0.259177895	1.618125501	2.433549841	0.016990899	-3.171179212
LENG8-AS1	0.306485752	1.40367075	2.433030323	0.017013706	-3.172262762
RP11-520P18.1	0.40583941	0.26563132	2.432828318	0.017022582	-3.172684028
KLHL34	0.201470906	0.206311811	2.432825186	0.017022719	-3.172690559
RP11-16L9.2	0.367477541	0.590613877	2.432429395	0.017040121	-3.173515861
NOP10	-0.414543866	6.145046039	-2.432375139	0.017042508	-3.173628987
RN7SKP181	0.241824489	0.115432574	2.431966012	0.017060516	-3.174481959
ST6GAL1	-0.335752758	1.383998508	-2.431601338	0.017076581	-3.175242149
RPL30P5	0.224559036	0.196549773	2.431293862	0.017090137	-3.175883029
SCAF4	0.315216571	3.288452335	2.431028135	0.01710186	-3.176436833
MIS18BP1	0.337272288	2.457696707	2.430828578	0.017110668	-3.176852699
RP11-68I18.2	0.34110724	0.490663158	2.430508139	0.017124821	-3.177520414
EIF1B	-0.286657778	3.852286568	-2.430347964	0.017131899	-3.17785415
MTND2P9	0.294890559	0.49663176	2.429675598	0.01716164	-3.179254868
CD247	-0.247341016	0.415302903	-2.429122901	0.017186123	-3.180406032
RNU4-74P	0.20990752	0.064767129	2.428772952	0.01720164	-3.181134795
AC098477.1	0.314334422	0.217406979	2.42876948	0.017201794	-3.181142024
AE000658.30	0.250229158	0.123283376	2.428721656	0.017203916	-3.181241609
AC108488.4	-0.309971263	1.439811163	-2.428564511	0.017210889	-3.181568824
LINC01150	-0.354763894	0.901877878	-2.428496978	0.017213886	-3.18170944
RNU5E-8P	0.230833294	0.102101677	2.428397932	0.017218283	-3.181915666
RP11-88E10.4	0.263568557	0.429683764	2.428354593	0.017220207	-3.182005899
TEX264	-0.270539681	2.943472875	-2.42835166	0.017220338	-3.182012006
PHF5A	-0.36816541	4.488754307	-2.427950857	0.017238143	-3.182846434
AP000705.7	0.229666412	0.207313865	2.42748007	0.017259078	-3.183826412
RP11-1072A3.4	-0.27559371	0.764242101	-2.427000345	0.017280433	-3.184824824
RP13-225O21.5	0.208140563	0.161994968	2.426967997	0.017281874	-3.18489214
CXorf57	-0.450829578	0.913780345	-2.426956347	0.017282393	-3.184916383
MDM1	0.243537903	1.161575923	2.426722391	0.017292818	-3.185403229

NIFKP3	0.226908199	0.17300836	2.426468226	0.017304149	-3.185932081
RN7SL39P	0.21057521	0.082695118	2.426040671	0.017323226	-3.186821606
AC234582.1	0.31357093	0.343188764	2.425745369	0.017336412	-3.187435899
RP11-355B11.1	0.200702384	0.071422492	2.425695809	0.017338626	-3.187538988
PPP1R8P1	0.227215549	0.202540114	2.425340588	0.017354502	-3.18827783
SPATA20	-0.382866207	3.15722653	-2.425133937	0.017363744	-3.18870761
RP11-521C10.1	0.238055796	0.087196954	2.424935292	0.017372632	-3.18912071
RP11-644F5.17	0.305274497	0.146164122	2.424386305	0.017397216	-3.190262226
RN7SL146P	0.29149218	0.440466843	2.424268997	0.017402474	-3.190506116
AC092071.1	0.230799273	0.130946695	2.424267528	0.017402539	-3.19050917
RP1-134O19.3	0.240157247	0.243408018	2.424265352	0.017402637	-3.190513694
RP11-402G3.6	0.2933731	0.119125932	2.424015854	0.017413823	-3.191032382
TNFSF10	-0.641033513	2.204482776	-2.423959784	0.017416338	-3.191148942
SF3B5	-0.363307051	6.250487516	-2.423937003	0.01741736	-3.191196298
RNU4-84P	0.269457195	0.170442688	2.423683447	0.017428737	-3.191723363
VTI1B	-0.234429375	3.576715121	-2.423026266	0.017458254	-3.193089213
NPM1P28	0.206852236	0.089747061	2.422932034	0.017462491	-3.193285034
RP11-79J24.1	0.597840218	0.204903554	2.422395533	0.017486626	-3.194399795
CTC-444N24.7	0.402164085	1.687383873	2.422241563	0.017493558	-3.194719681
COX6C	-0.382865067	4.711532063	-2.421849791	0.017511208	-3.195533539
GRIP1	0.240602385	0.270213366	2.421632308	0.017521013	-3.195985285
PDK1	0.389108264	1.407219521	2.4216051	0.01752224	-3.196041799
ADRA1B	-0.324123095	0.780004468	-2.421480288	0.017527869	-3.196301033
RP11-111A22.1	0.245764364	0.278916235	2.42145231	0.017529131	-3.196359142
GBAP1	0.263925692	1.117493745	2.421206332	0.017540231	-3.196870002
TEF	-0.38341991	2.126222584	-2.421060914	0.017546796	-3.197171993
AL157788.1	0.247653649	0.104593093	2.420963397	0.0175512	-3.197374498
DYRK1A	0.294189547	3.12233876	2.420760833	0.01756035	-3.197795124
SRXN1	0.358630654	1.012096413	2.420744219	0.017561101	-3.19782962
RC3H2	0.256635459	2.61229847	2.420164435	0.017587317	-3.19903337
RP11-380G5.2	0.330108062	0.359056132	2.419764971	0.017605399	-3.199862594
RP11-567C20.2	0.216596222	0.071259911	2.419744517	0.017606325	-3.19990505
NECAB3	0.367693468	2.583635385	2.419513966	0.01761677	-3.20038358

RP11-486A14.1	0.20710162	0.169109096	2.41948603	0.017618036	-3.200441561
RP11-416K24.1	0.214304147	0.103491711	2.418755291	0.017651178	-3.201958002
ACAA1	-0.245414268	2.10568296	-2.4187122	0.017653134	-3.202047413
MIR606	0.380804724	0.238469869	2.418686346	0.017654308	-3.202101059
RNY3P8	0.43100362	0.661551133	2.418579843	0.017659143	-3.202322036
HMGCL	-0.264617183	2.670479512	-2.418282546	0.017672649	-3.202938841
RPS29P24	0.311303764	0.130840736	2.418195908	0.017676586	-3.203118576
RP11-158D2.1	0.25364088	0.228964725	2.417888166	0.017690578	-3.203756962
PKIG	0.325206538	4.460964672	2.417846827	0.017692458	-3.203842712
RP5-1096D14.6	0.233638655	0.114617928	2.417549514	0.017705987	-3.204459388
CD5	-0.239635287	0.422434857	-2.417512608	0.017707667	-3.204535933
MIR320D2	0.252053695	0.089925941	2.417393833	0.017713074	-3.20478227
TBC1D24	0.223349291	1.965152025	2.417266659	0.017718866	-3.205046015
SLC6A3	0.269990559	0.157459668	2.417213897	0.017721269	-3.205155435
MIR3163	0.248820741	0.201303164	2.417000161	0.017731008	-3.205598664
KCNQ1	-0.312719589	1.659185081	-2.41683745	0.017738425	-3.205936059
RP11-490K7.1	0.273826713	0.218284872	2.416722028	0.017743688	-3.206175385
ST13P19	0.210579483	0.479091378	2.416537269	0.017752116	-3.20655846
RNU6-788P	0.221894875	0.085099794	2.416426835	0.017757155	-3.206787419
SNORA11C	0.242244412	0.131154415	2.416361164	0.017760152	-3.206923567
RP11-47A8.5	0.584123909	3.196040035	2.415792685	0.017786115	-3.208102005
CRAMP1L	0.202055744	1.549975336	2.415645721	0.017792833	-3.208406616
MLLT4	0.376768471	2.586977385	2.415372552	0.017805325	-3.208972772
RP11-228B15.4	0.317093144	1.146787865	2.414478896	0.017846248	-3.210824527
RP11-680F8.3	0.297492085	0.996436195	2.414242971	0.017857066	-3.211313291
MIR130B	0.253423481	0.286325385	2.414235238	0.01785742	-3.211329311
LRRC37A7P	0.430169603	0.739459234	2.414200603	0.017859009	-3.211401059
TMEM88	-0.449090442	1.493933544	-2.413749361	0.017879718	-3.212335766
RP11-24M17.3	0.31008522	0.36279486	2.413509597	0.01789073	-3.212832354
RP5-968D22.1	0.202283754	0.114181958	2.413235417	0.01790333	-3.213400172
RP11-449J3.3	0.211241977	0.070337518	2.413003994	0.017913971	-3.213879396
KLF4	0.551305864	3.312887124	2.412810234	0.017922885	-3.214280598
SRGAP1	0.257771064	1.162129369	2.41280785	0.017922995	-3.214285536

AC011998.4	0.238374323	0.192158056	2.4123452	0.017944295	-3.215243392
PPP4C	-0.299858072	4.90566203	-2.412221325	0.017950002	-3.215499834
RP11-275I14.4	0.21807518	0.640657977	2.411995245	0.017960421	-3.215967824
GPR84	-0.314986622	0.824888068	-2.411508572	0.01798287	-3.216975121
DRAM1	-0.325376819	1.970943102	-2.411391766	0.017988261	-3.217216856
RGS18	-0.230474213	0.611596126	-2.411104471	0.018001528	-3.217811381
CKAP4	0.367596132	6.738592593	2.410719139	0.018019337	-3.218608687
C8orf89	0.454098411	0.305981275	2.410640478	0.018022974	-3.218771433
PNPLA8	0.278369082	2.728822365	2.409969155	0.018054042	-3.220160194
CDK19	0.297917968	2.307759843	2.409911327	0.018056721	-3.220279807
RP5-1155K23.4	0.25232587	0.118563319	2.409472119	0.018077075	-3.221188192
SH2D2A	0.394222785	1.238228391	2.408937555	0.018101877	-3.222293603
EXOC4	0.229437926	2.86668208	2.408346774	0.018129322	-3.223515018
RFXAP	0.253465784	1.723715415	2.407974951	0.018146614	-3.224283613
RFTN1	-0.393202471	3.121393337	-2.407796465	0.01815492	-3.224652525
AL139383.1	0.216837951	0.114885431	2.407296678	0.018178196	-3.225685409
MIR4280	0.282926129	0.107414769	2.407203424	0.018182541	-3.225878111
RP3-523E19.2	0.297255487	0.813490014	2.406981879	0.01819287	-3.22633589
RP11-259F16.3	0.204399062	0.180194351	2.40672274	0.018204958	-3.226871307
RN7SL499P	0.285903712	0.260847387	2.406264276	0.01822636	-3.22781843
RPL23AP62	0.20444109	0.12015906	2.406003286	0.018238554	-3.228357532
GIP	0.258002519	0.171501613	2.405913301	0.01824276	-3.228543392
RN7SKP177	0.248936547	0.109905308	2.405706801	0.018252416	-3.228969888
RN7SL834P	0.366174854	0.436002024	2.405185056	0.018276831	-3.230047338
HOXD10	0.442463581	1.136582388	2.405146373	0.018278642	-3.230127214
RP11-146N23.1	0.552194228	1.504255319	2.404815754	0.01829413	-3.230809857
CCDC144NL	0.242972698	0.276365015	2.404598209	0.018304328	-3.231258987
RNA5SP28	0.281877223	0.201215338	2.40427408	0.01831953	-3.2319281
SNORD81	0.487706129	0.312036402	2.403724431	0.018345336	-3.233062583
RP11-665J16.1	0.203324056	0.096051886	2.403624558	0.018350029	-3.233268699
SNORD8	0.381886994	0.54620991	2.403139355	0.018372841	-3.234269943
STAG3L4	0.29128164	1.662076877	2.402676857	0.01839461	-3.23522417
MIR320C1	0.258940703	0.418808775	2.402545552	0.018400794	-3.235495051

KAT6B	0.314549099	1.597355711	2.402415203	0.018406935	-3.235763946
AIFM1	-0.313185563	3.483017114	-2.40233854	0.018410548	-3.235922088
RP11-25L19.1	0.310652569	0.11607123	2.402335552	0.018410689	-3.235928251
UQCR10	-0.399641432	5.949230621	-2.402134464	0.018420168	-3.236343037
CT867977.1	0.321352384	0.175639587	2.401479291	0.018451083	-3.237694258
RNU7-52P	0.290369094	0.270007241	2.401415268	0.018454106	-3.237826282
EIF6	-0.351074521	5.753974574	-2.401162419	0.018466051	-3.238347657
RP11-843F11.1	0.234781087	0.097537819	2.401040303	0.018471822	-3.238599443
TAPBPL	-0.416248509	2.309576812	-2.399987032	0.018521668	-3.240770686
SPG20	0.288084742	3.02384649	2.399898859	0.018525846	-3.240952411
AC093724.2	0.29948314	1.979492654	2.399882165	0.018526637	-3.240986818
RN7SL104P	0.23178759	0.096261537	2.399806631	0.018530218	-3.241142487
ZDHHC18	0.213692048	3.045396601	2.399424125	0.018548357	-3.241930742
RNU6-633P	0.301079392	0.279072362	2.3994193	0.018548585	-3.241940685
DECR1	-0.314765965	3.775362273	-2.39924649	0.018556786	-3.242296768
PDCD4-AS1	-0.371139408	2.177763474	-2.399229508	0.018557592	-3.24233176
AL133243.3	0.303661	0.287916662	2.399020062	0.018567535	-3.242763301
SLC25A36	0.323009014	2.764873522	2.398688187	0.0185833	-3.24344703
RP11-77M5.1	0.256113857	0.245798695	2.39868717	0.018583349	-3.243449123
RP11-196D18.5	0.20921383	0.090918542	2.398617784	0.018586646	-3.243592062
DYNLL1	-0.252979382	6.036200335	-2.398608383	0.018587093	-3.243611428
AC144530.1	0.506406828	2.322279523	2.398526475	0.018590987	-3.243780158
RP11-317N8.4	0.20175844	0.450169429	2.398411401	0.018596458	-3.244017201
SMG7	0.284066193	3.348871911	2.39841003	0.018596523	-3.244020025
HAVCR2	-0.469022392	2.331038094	-2.398195744	0.018606715	-3.244461407
AP001604.1	0.464563825	0.268035451	2.397433916	0.018642991	-3.246030336
POPDC2	-0.275426154	1.316763839	-2.397279064	0.018650372	-3.246349188
SMARCC1	0.294913871	3.943461405	2.397204775	0.018653914	-3.24650215
LINC01078	0.251863172	0.088999978	2.397144436	0.018656791	-3.246626384
RN7SL619P	0.205651323	0.238033067	2.396638459	0.018680935	-3.247668058
RNU6-402P	0.311579698	0.173023916	2.396050987	0.018709002	-3.248877272
MIR508	0.272449672	0.085127424	2.396026544	0.01871017	-3.248927579
TDG	0.267497668	2.825292739	2.395573805	0.018731827	-3.249859283

CARD8-AS1	-0.239107437	1.466386606	-2.394991074	0.018759735	-3.251058277
FTLP8	0.233619637	0.21341105	2.393754589	0.018819074	-3.253601564
PLA2G7	-0.38948843	1.841136083	-2.393599211	0.018826543	-3.253921075
MIR7-2	0.222010914	0.121682407	2.392934679	0.018858514	-3.255287385
SLC16A7	0.316266678	1.262368734	2.392829575	0.018863575	-3.255503453
ATPAF1	0.237061203	3.319095574	2.392822051	0.018863938	-3.255518919
RP11-785G17.1	0.230490457	0.107317065	2.392788965	0.018865531	-3.255586934
AC078927.1	0.319055121	0.10944159	2.392653299	0.018872066	-3.255865816
RP11-565F19.3	0.275027193	0.208113608	2.392640175	0.018872699	-3.255892794
RNU6-1236P	0.27315091	0.104596459	2.39221593	0.018893148	-3.256764795
HLA-DQB1-AS1	-0.310949493	0.575817055	-2.391398937	0.018932585	-3.258443685
RP11-295D4.4	0.293306421	0.371853456	2.391279501	0.018938357	-3.258689079
LL0XNC01-220B11.1	0.265971014	0.128760605	2.39122861	0.018940816	-3.258793637
RAB23	0.418451517	2.503501389	2.391197642	0.018942313	-3.258857261
RP11-465N4.4	0.205138387	0.377305175	2.391017513	0.018951022	-3.259227325
CD200R1	-0.200505373	0.469972503	-2.390800467	0.018961521	-3.259673201
PSME2	-0.416585364	4.710455204	-2.390688414	0.018966943	-3.259903379
MIR6070	0.335766526	0.215068573	2.390387192	0.018981525	-3.260522093
RP4-601P9.1	0.255844585	0.111044583	2.390023916	0.018999124	-3.261268179
ASPH	0.357128594	3.562074694	2.389717507	0.01901398	-3.261897397
AHCYL1	-0.260253576	5.297028621	-2.389267655	0.019035809	-3.26282105
VTRNA2-2P	0.399973251	0.211629413	2.389034856	0.019047115	-3.263298984
ARID4B	0.264939324	1.959262556	2.38887538	0.019054863	-3.263626361
LGALS3	-0.422081825	5.405840345	-2.388802495	0.019058405	-3.263775977
RNU6-178P	0.339460699	0.295633225	2.38869634	0.019063565	-3.26399388
MFSD5	-0.24727698	3.722972325	-2.388541311	0.019071102	-3.264312091
AC097463.1	0.273936528	0.112779817	2.38820635	0.019087398	-3.264999569
MKI67	0.411351982	3.582384194	2.388068472	0.019094109	-3.265282525
RP11-267J23.1	0.230878196	0.326840303	2.3880146	0.019096732	-3.265393079
BCAS2P3	0.243525545	0.109211714	2.387843447	0.019105066	-3.265744298
RP11-526D8.11	0.307499615	1.507652068	2.387765286	0.019108874	-3.265904684
AC073508.1	0.276829023	0.164150381	2.387757932	0.019109232	-3.265919774
NCKAP1L	-0.443024206	1.91588844	-2.387378919	0.019127704	-3.266697437

RNU6-111P	0.300854785	0.115014018	2.387157637	0.019138496	-3.267151415
AC007568.1	0.213465184	0.109769433	2.3869634	0.019147974	-3.267549879
RP5-859M6.1	0.252193975	0.14390673	2.38686529	0.019152762	-3.267751134
RN7SL380P	0.216320544	0.081824393	2.386191182	0.019185694	-3.269133754
ATP5J2P3	0.219990025	0.134877498	2.386168059	0.019186825	-3.269181173
EMC3	-0.273903902	3.478405088	-2.385775219	0.01920604	-3.269986739
CABLES2	0.345155143	2.285984993	2.385763516	0.019206613	-3.270010736
AC003664.1	0.371155245	0.239864413	2.385456456	0.019221645	-3.270640317
RP11-780O17.1	0.2238603	0.075066036	2.385158161	0.019236257	-3.27125186
HSDL1	-0.320131814	2.59183014	-2.385096318	0.019239288	-3.271378637
TSPAN4	-0.34652352	3.293731207	-2.385080666	0.019240055	-3.271410721
RP11-73E6.2	0.237896368	0.145118679	2.385031396	0.01924247	-3.271511724
SRGNP1	0.209400805	0.06574537	2.385022029	0.019242929	-3.271530925
RP11-517B11.6	0.224228558	0.11150247	2.384975402	0.019245215	-3.271626504
MIR181A2	0.229741379	0.179567728	2.384916509	0.019248103	-3.271747227
RNU6-1139P	0.215574506	0.09285094	2.384268272	0.01927991	-3.273075849
EFNB3	-0.686086114	1.493399105	-2.384119471	0.019287217	-3.273380787
COX4I1	-0.410838429	5.389478443	-2.383953922	0.019295351	-3.273720026
CTD-2313J17.5	0.219985585	0.392892641	2.383602172	0.019312642	-3.274440758
B3GALTL	0.307145627	2.365880985	2.383584395	0.019313516	-3.274477181
PTMS	-0.354166986	7.927148131	-2.383512044	0.019317075	-3.274625414
AC003989.4	0.298063047	0.185577195	2.383390821	0.019323038	-3.274873769
MDM2	0.365230781	2.426419393	2.382500255	0.019366899	-3.276697977
IFI30	-0.321270354	1.04740667	-2.382422593	0.019370728	-3.276857029
RP11-200A13.1	0.336678808	0.18281081	2.382257011	0.019378894	-3.277196127
RPS27P29	0.324295926	0.984643834	2.382244296	0.019379521	-3.277222165
RP11-473M20.9	-0.210571159	0.597943727	-2.382199711	0.019381721	-3.277313468
CTD-3065B20.2	0.223662601	0.488318175	2.38208642	0.01938731	-3.277545463
RN7SL89P	0.361954616	0.210545593	2.382069322	0.019388154	-3.277580473
CTB-167G5.3	0.234384178	0.431875338	2.382050379	0.019389089	-3.277619263
PKD1P5	0.253943182	0.457298804	2.382029735	0.019390108	-3.277661536
AC118463.1	0.274673905	0.179551916	2.381921927	0.019395429	-3.27788229
PPME1	0.236473576	2.917935975	2.381879653	0.019397516	-3.277968848

AC073330.1	0.452855688	0.238635414	2.381721509	0.019405325	-3.27829265
FAM177A1P1	0.206762529	0.160118162	2.381638615	0.019409419	-3.278462371
RP11-204C16.4	0.254235143	1.153949707	2.381192101	0.019431486	-3.279376486
CDK5	-0.328173734	3.021015169	-2.381130906	0.019434512	-3.279501755
TAF5L	0.200685605	2.094740009	2.381120735	0.019435015	-3.279522575
RNU6-1323P	0.211970513	0.111592744	2.380167267	0.019482219	-3.281473994
RNU1-138P	0.365732386	0.294181275	2.379947562	0.019493111	-3.281923557
RP11-350G8.4	0.344704225	0.158927793	2.379732012	0.019503801	-3.282364585
TRAPPC1	-0.654341543	5.954141324	-2.379430912	0.019518744	-3.282980593
U4atac	0.231749104	0.155019733	2.378690461	0.019555533	-3.284495168
HCK	-0.476438079	2.458258983	-2.37858318	0.019560868	-3.284714574
ARMC7	-0.298805986	2.694571568	-2.378539932	0.01956302	-3.284803021
RP11-53L24.1	0.32324091	0.28028672	2.378110634	0.019584385	-3.285680903
ZC3H4	0.249615611	3.136135575	2.377240977	0.019627729	-3.287458868
CTD-2291D10.3	0.25906952	0.140458452	2.377192609	0.019630142	-3.287557736
RP5-887A10.1	0.26516276	0.125210762	2.377127451	0.019633394	-3.287690923
MZT1	0.396658513	3.268317374	2.376886421	0.019645425	-3.288183574
PCDH9-AS2	0.212770003	0.132032437	2.376794243	0.019650028	-3.288371969
RP11-486F17.1	0.225174679	0.272529699	2.376563557	0.019661551	-3.288843422
TUBGCP3	0.392259613	2.316698678	2.376404273	0.019669511	-3.289168929
C11orf54	0.290530243	2.008372618	2.375803185	0.019699577	-3.290397114
GLDC	0.281207732	0.528878046	2.375773141	0.01970108	-3.290458495
RNU7-188P	0.226081714	0.120863065	2.375519766	0.019713767	-3.290976121
R3HDM4	-0.264464931	4.417924554	-2.375242571	0.019727654	-3.291542356
RNU6-716P	0.340262005	0.123651203	2.375222594	0.019728655	-3.291583163
SECISBP2	0.226508812	1.887201507	2.374726904	0.019753512	-3.292595573
CLEC18A	0.219239119	0.243109724	2.374639589	0.019757893	-3.292773888
RNU6-940P	0.34267067	0.118897617	2.374572988	0.019761236	-3.292909898
AC083884.8	0.203823425	0.387575532	2.37453483	0.019763151	-3.292987822
TTC13	0.28467548	2.085905895	2.374311068	0.019774386	-3.293444747
RP11-274B21.13	0.396081239	1.959956457	2.374215547	0.019779183	-3.293639792
AKAP9	0.328788757	1.954553397	2.373988268	0.019790603	-3.294103846
AC011897.2	0.219089721	0.095827062	2.373960117	0.019792018	-3.29416132

AC002115.5	0.327671917	0.182256721	2.373835533	0.01979828	-3.294415673
AC006534.1	0.260897438	0.100493387	2.373715224	0.019804329	-3.29466129
TSPAN9	-0.384422724	3.806780258	-2.373711319	0.019804526	-3.294669261
MAPKAPK3	-0.327783065	3.4418886	-2.373680253	0.019806088	-3.294732682
KALRN	0.244622508	0.988062258	2.37366793	0.019806708	-3.294757839
DNAJC16	0.235351155	2.252467464	2.373441474	0.0198181	-3.295220119
RP13-20L14.4	0.243201248	0.268899296	2.373432365	0.019818558	-3.295238713
AC114755.5	0.247322068	0.192730077	2.373151982	0.019832672	-3.295811024
AC012485.2	0.236316046	0.099238658	2.373034348	0.019838596	-3.296051117
RP5-1073F15.1	0.270224069	0.23353217	2.372941363	0.019843279	-3.296240895
AP000436.4	0.219243014	0.167612222	2.372713389	0.019854767	-3.296706151
HECTD2	0.234747115	1.269731232	2.372708104	0.019855033	-3.296716935
RNU6-839P	0.201218767	0.07680878	2.372214883	0.019879906	-3.29772338
LILRB4	-0.506454624	1.672733137	-2.371932393	0.019894165	-3.298299733
LINC00337	0.263226237	0.444124033	2.371184271	0.01993197	-3.299825813
EXOSC7	-0.289588975	2.423174889	-2.370913154	0.019945686	-3.300378757
RP5-1142J19.2	0.280526765	0.607828219	2.370765265	0.019953172	-3.300680354
MAGI2	0.33254326	1.401446296	2.37050408	0.019966398	-3.30121296
UHRF2	0.311771046	2.303960633	2.370343558	0.01997453	-3.301540269
HMGB3P19	0.25503124	0.105133277	2.370043248	0.019989753	-3.302152559
RNY4P27	0.421996705	0.185507589	2.369058097	0.020039761	-3.304160675
HIST1H2APS4	0.217735005	0.273016165	2.368865169	0.020049568	-3.30455385
FAM76B	0.256731694	1.58361272	2.368618022	0.020062136	-3.30505748
MUC17	0.247107897	0.085212199	2.368216155	0.020082588	-3.305876299
HNRNPCP7	0.290707861	1.132635474	2.367696281	0.020109073	-3.306935382
LINC01047	0.207107079	0.0807189	2.367253639	0.020131648	-3.307836971
RP11-58A17.3	0.219875476	0.232187427	2.367214969	0.020133621	-3.307915728
CTD-2340D6.1	0.230117967	0.180056539	2.366883842	0.020150525	-3.308590073
AL031905.1	0.2995757	0.146285875	2.3667954	0.020155042	-3.308770171
RP11-864I4.1	0.221749677	1.380741175	2.366331404	0.020178754	-3.309714939
WDR27	0.324715214	1.315504955	2.366202848	0.020185328	-3.30997667
KLHL35	0.243508461	0.858024922	2.364521123	0.020271505	-3.313399408
TRIM24	0.238503602	2.152238065	2.363613038	0.020318174	-3.315246714

RP11-497G19.2	0.247816312	0.141291381	2.363427854	0.020327702	-3.315623357
RN7SKP213	0.206195292	0.107334238	2.363210775	0.020338877	-3.316064837
RP11-192B18.1	0.217102794	0.078384524	2.363125192	0.020343285	-3.316238878
CHORDC1	0.321010276	1.886937441	2.362609432	0.020369862	-3.317287621
RP11-26H16.1	0.277722199	0.818359463	2.362111588	0.020395546	-3.318299743
RN7SL670P	0.227040622	0.163666227	2.36099817	0.020453091	-3.320562665
DHPS	0.36559803	3.852903861	2.360081854	0.020500557	-3.3224243
MIR24-2	0.30488444	0.368031961	2.359961158	0.020506817	-3.322669466
CREBBP	0.287597466	3.085476095	2.359857225	0.020512208	-3.322880571
RP11-384C12.1	0.235388004	0.105581509	2.359759082	0.0205173	-3.323079911
RNU6-1189P	0.265626232	0.344342361	2.35966951	0.020521949	-3.323261834
IL1B	-0.234717495	0.57408278	-2.359430284	0.020534369	-3.323747682
TYMP	-0.668050152	3.685027599	-2.359124618	0.020550247	-3.3243684
MDGA2	0.235942932	0.192399319	2.35858136	0.020578495	-3.325471424
AC092198.2	0.324718433	0.124654098	2.35844405	0.020585641	-3.325750184
RP11-323I14__A.1	0.267809036	0.154233552	2.358328802	0.02059164	-3.325984141
RNU6-768P	0.224762646	0.101618581	2.357792675	0.020619566	-3.32707237
RP11-115E19.1	0.21012179	0.092224714	2.35773635	0.020622502	-3.327186687
RNU7-175P	0.354477093	0.259761252	2.357589696	0.020630148	-3.32748432
RP11-160N1.8	0.213789034	0.079703191	2.35718109	0.020651465	-3.328313502
CERS2	0.332459901	5.440897954	2.357014111	0.020660182	-3.328652316
CARD9	-0.222727896	1.009940138	-2.356728294	0.02067511	-3.329232211
RP11-307I14.2	0.20358576	0.340108529	2.356575971	0.020683069	-3.329541237
AL158040.1	0.302922891	0.19987794	2.356519932	0.020685998	-3.329654921
FRG1	-0.297548713	3.515700316	-2.356475536	0.020688319	-3.329744984
RTN2	-0.384177432	1.674242912	-2.35628673	0.020698191	-3.330127983
RP11-336K24.5	0.294233886	0.153186639	2.356170127	0.02070429	-3.330364505
POLR2E	-0.287323597	4.876590924	-2.355990119	0.020713708	-3.330729618
RP11-95M15.1	0.238218881	0.114928689	2.35584995	0.020721044	-3.331013907
RN7SL225P	0.208215009	0.211658291	2.355660955	0.02073094	-3.331397204
RP11-21M7.2	0.2518048	0.207994049	2.354962404	0.020767553	-3.332813685
RNU6-777P	0.327758646	0.195042263	2.354875519	0.02077211	-3.332989839
AL117341.1	0.255585486	0.229477078	2.354760585	0.020778141	-3.333222854

RNY1P16	0.444052088	0.79847493	2.354368462	0.020798727	-3.334017757
MIR3135B	0.241975791	0.121208382	2.354163183	0.020809512	-3.33443385
AL391095.1	0.297852039	0.097549587	2.353838986	0.020826553	-3.335090919
RASGEF1A	0.344816653	0.642914023	2.353645005	0.020836756	-3.335484035
ATP5J2LP	0.221562348	0.207080226	2.353442856	0.020847393	-3.335893673
CU104787.1	0.414090519	0.338603093	2.353287407	0.020855576	-3.336208657
DIP2A	0.242222371	1.880989764	2.352982832	0.020871617	-3.336825761
SPRYD7	0.314557663	2.003696858	2.352613874	0.020891064	-3.337573218
HMGB1P19	0.246385168	0.244599295	2.352532709	0.020895344	-3.337737633
NMUR1	0.277585021	0.519812817	2.35248604	0.020897806	-3.337832167
WARS	-0.39789028	4.289450134	-2.35217912	0.020914	-3.338453838
SOSTDC1	-0.485684999	0.542913128	-2.351993734	0.020923787	-3.338829305
LSM4	-0.305428142	4.503882356	-2.351879752	0.020929806	-3.339060143
ENOSF1	0.277721095	1.596358059	2.351708423	0.020938857	-3.339407102
RP11-486A14.2	0.226763804	0.459797823	2.351658266	0.020941507	-3.339508671
FAUP2	0.316195732	0.205697241	2.351431156	0.020953511	-3.339968551
CASC11	0.321719398	0.149010301	2.351430246	0.020953559	-3.339970394
FAM58A	-0.317112291	3.280844302	-2.351308116	0.020960017	-3.340217682
RHOG	-0.362238263	4.883925246	-2.351039399	0.020974232	-3.340761738
CTD-2341M24.1	-0.327322567	1.10548437	-2.350954291	0.020978736	-3.34093404
FADS1	0.457406254	3.643431746	2.350512794	0.021002114	-3.34182777
RNA5SP317	0.431600764	0.990138493	2.350419921	0.021007034	-3.342015755
WDR31	0.214749507	0.966731458	2.350369987	0.021009681	-3.342116824
RP11-286B14.2	0.41004903	0.350883577	2.350239939	0.021016573	-3.34238004
RNU6-597P	0.378267821	0.404866563	2.350140372	0.021021852	-3.342581555
RTN3P1	0.339884619	1.203767645	2.349838817	0.021037846	-3.343191828
NDUFB8	-0.36439503	3.503325083	-2.349556205	0.021052846	-3.343763705
RP11-925D8.1	0.29447276	0.195210224	2.349555221	0.021052898	-3.343765695
HOOK2	0.372937748	1.935858263	2.349187577	0.021072425	-3.344509547
RPS3P6	0.445981246	1.50232611	2.349137429	0.02107509	-3.344611003
CHMP2A	-0.296421493	4.467310725	-2.349072806	0.021078524	-3.344741742
RNU6-687P	0.211038248	0.06235221	2.349059047	0.021079255	-3.344769578
RP11-148B3.2	0.244668327	0.142072026	2.348758223	0.02109525	-3.345378129

DNAJB9	0.392579878	3.558635444	2.34763521	0.021155053	-3.347649326
RP11-253I19.3	0.239680584	0.42333533	2.347523943	0.021160987	-3.347874303
AC019330.1	0.243973211	0.231856872	2.347285878	0.021173687	-3.348355627
SEPW1P	0.375807049	0.499118308	2.346946799	0.021191787	-3.34904111
RP11-395L14.11	-0.314154517	1.940498805	-2.346551882	0.021212886	-3.349839369
AC011516.1	0.212777178	0.090595489	2.345578285	0.02126498	-3.35180683
SAC3D1	0.444322527	3.650749957	2.345497066	0.021269331	-3.351970925
RP11-286H14.8	0.278847032	0.600351271	2.345288428	0.021280511	-3.352392439
RN7SL180P	0.352619816	0.377295815	2.345005139	0.0212957	-3.352964719
RP11-890B15.2	0.253395087	0.146735554	2.344843035	0.021304396	-3.353292164
CNPY2	-0.318329098	3.434078792	-2.344795024	0.021306972	-3.35338914
RNU6-554P	0.38072963	0.182291529	2.344788111	0.021307343	-3.353403103
PPP1R15B	0.296782592	3.740705627	2.34460412	0.021317218	-3.353774728
RP13-923O23.7	0.222768972	0.522962231	2.344436045	0.021326242	-3.354114181
RNU6-19P	0.473830252	0.588666471	2.344330904	0.021331889	-3.354326519
NDUFA2	-0.392065861	4.496514173	-2.344328462	0.02133202	-3.35433145
RP11-96D24.1	0.256770382	0.120465706	2.344309562	0.021333035	-3.35436962
CH17-132F21.4	0.285703281	0.125039807	2.344144334	0.021341912	-3.354703286
SELPLG	-0.604791543	3.213166181	-2.343679795	0.021366888	-3.355641285
AP001198.1	0.45215795	0.344369471	2.343662591	0.021367813	-3.35567602
RP11-26L16.1	0.225156218	0.137162098	2.343434744	0.021380073	-3.356136028
FBXO21	0.251969804	2.63514727	2.343313383	0.021386606	-3.356381032
RP11-325B23.2	0.213886802	0.084104915	2.343189744	0.021393263	-3.356630623
RP4-614O4.12	0.229860954	0.38870595	2.3429341	0.021407034	-3.357146657
NUBP1	-0.228564992	2.969569948	-2.342709194	0.021419155	-3.357600603
RP11-377K22.2	0.611099637	1.948329568	2.342203494	0.021446433	-3.358621162
MIR101-2	0.312393305	0.232357007	2.34219186	0.02144706	-3.358644638
NANOGNBP3	0.214188792	0.269658093	2.342135317	0.021450112	-3.358758736
NCOR1P2	0.235340848	0.219242371	2.342073192	0.021453466	-3.358884095
SHISA5	-0.296850848	5.045154738	-2.34206652	0.021453826	-3.358897559
RNU6ATAC14P	0.448075817	0.468571295	2.342012428	0.021456747	-3.359006705
GBP4	-0.513474416	1.516314833	-2.341821365	0.021467065	-3.359392213
MIR3692	0.228596267	0.117811738	2.341818026	0.021467245	-3.359398951

ACAP2-IT1	0.215110232	0.431729399	2.341810001	0.021467679	-3.359415141
C7orf43	-0.250947736	2.333299312	-2.341486472	0.021485161	-3.360067864
RP11-3M1.3	0.246788029	0.09628227	2.340967407	0.021513236	-3.361114915
LSM6P2	0.287327136	0.226157994	2.340602391	0.021532998	-3.361851101
RP11-22C8.1	0.253852506	0.165803895	2.340187649	0.021555472	-3.362687456
CTD-2145A24.5	-0.226734797	0.554400538	-2.340059947	0.021562396	-3.362944949
ENO1-IT1	0.345254941	0.663808838	2.339589179	0.021587938	-3.36389408
USE1	-0.317613205	2.808832944	-2.339582986	0.021588274	-3.363906567
CREG1	-0.417519133	4.635218888	-2.338853221	0.021627922	-3.36537754
CTBP2P1	0.291868488	0.278388859	2.338550542	0.021644386	-3.365987528
RP11-504P24.3	0.232179636	0.774121825	2.338495438	0.021647384	-3.36609857
TBC1D10C	-0.221965258	0.536277813	-2.338021068	0.021673212	-3.367054409
AL132671.1	0.463943343	0.180284241	2.337782038	0.021686236	-3.367535979
LCK	-0.329435218	0.642947072	-2.337737525	0.021688662	-3.367625656
MIR7856	0.420397142	0.627575032	2.337597432	0.0216963	-3.367907876
CTD-2547L16.3	0.202306801	0.159808554	2.337251818	0.021715152	-3.368604058
AC097361.1	0.237622587	0.103604315	2.337206816	0.021717608	-3.368694701
PIK3AP1	-0.458042989	2.032058696	-2.337104353	0.0217232	-3.368901075
CLEC1A	-0.288958877	0.801921419	-2.336807	0.021739437	-3.36949994
RP11-343J18.2	0.220246434	0.085816555	2.336566396	0.021752583	-3.369984463
ARHGAP4	-0.4936839	2.443290607	-2.33633542	0.021765209	-3.370449558
RP11-439L18.2	0.215393117	0.535834848	2.335515608	0.021810077	-3.372100012
RTTN	0.237041717	1.141499082	2.335316285	0.021820998	-3.372501215
RP11-142L4.2	0.242896804	0.487819991	2.335295939	0.021822113	-3.372542167
RP11-132A1.4	0.530364024	1.081303702	2.334313012	0.021876042	-3.374520178
RNA5SP398	0.232241156	0.09443387	2.334293803	0.021877098	-3.374558825
RNU4-75P	0.217884844	0.087731356	2.334128285	0.021886191	-3.374891835
PID1	0.528616021	2.343353608	2.333726405	0.021908283	-3.375700302
BRD2	0.295003542	5.162384434	2.33351352	0.021919994	-3.376128516
RP11-252K23.1	0.21092451	0.240401432	2.332790967	0.021959784	-3.377581662
RNY1P12	0.291684399	0.29354837	2.332091538	0.02199836	-3.37898793
GALM	-0.434617129	3.409873056	-2.332040497	0.022001178	-3.379090537
E2F1	0.364140136	3.865497637	2.331698032	0.02202009	-3.379778949

XXbac-BPG299F13.14	0.252669814	0.516568828	2.331149828	0.022050395	-3.380880744
CTD-2515A14.1	0.308311318	0.536056326	2.330955542	0.022061144	-3.381271172
RP11-561O4.1	0.246379944	0.165683849	2.330687046	0.022076006	-3.381810682
AP4E1	0.244222093	1.798761238	2.330646998	0.022078223	-3.381891148
CBWD1	0.207510962	0.777376812	2.330335482	0.022095479	-3.382517023
TIMM9P2	0.355414976	0.655895087	2.33008671	0.022109269	-3.383016786
PDZD2	0.361752437	1.457209372	2.329925512	0.022118208	-3.383340596
AC025750.7	0.213143294	0.137787205	2.329910418	0.022119045	-3.383370914
RNU6-59P	0.231371664	0.118047046	2.329229856	0.022156821	-3.384737779
MORC3	0.310424482	2.524692741	2.329041099	0.022167308	-3.385116824
RPL21P33	0.260499613	0.157412525	2.32885679	0.022177553	-3.385486911
MIR4288	0.244778489	0.182065101	2.328635035	0.022189885	-3.385932155
NIPSNAP1	-0.462368125	3.377129653	-2.328333153	0.022206682	-3.386538219
TDGF1P6	0.283868535	0.294966453	2.328195814	0.022214327	-3.386813921
WDR60	0.303842697	1.708703467	2.328187593	0.022214785	-3.386830424
RP11-380M21.1	0.209143167	0.197868237	2.327872447	0.022232337	-3.387463009
KIF24	0.219102315	1.080980038	2.327626518	0.022246044	-3.387956606
UBR3	0.29965131	2.494824017	2.327280993	0.022265313	-3.388650021
SLC15A3	-0.444359167	2.431284744	-2.327024065	0.022279651	-3.389165578
SNCG	-0.5425557	1.743437502	-2.326986188	0.022281765	-3.389241578
PI4K2A	-0.261947103	3.093889487	-2.32673676	0.022295694	-3.38974203
FAT3	0.680350825	2.970586583	2.32655955	0.022305594	-3.390097556
IL21-AS1	0.243210559	0.165968527	2.326266064	0.022321999	-3.390686307
SCX	0.887723695	2.580244146	2.325980665	0.022337963	-3.391258773
TP53I3	-0.34027093	3.28557332	-2.325767122	0.022349913	-3.391687066
SLMO1	0.276230366	1.689245346	2.325512582	0.022364166	-3.392197542
NAF1	0.256433016	1.794514819	2.325059752	0.022389541	-3.393105563
RNU7-34P	0.274809553	0.091933208	2.324904445	0.02239825	-3.393416952
C12orf5	-0.311228529	2.323912389	-2.324817798	0.02240311	-3.393590669
C18orf8	0.285910965	2.912974492	2.32459287	0.02241573	-3.394041601
RP11-900F13.2	0.266807521	0.135264588	2.324429177	0.022424919	-3.394369744
PHB2	-0.373609458	5.83060025	-2.324305207	0.02243188	-3.394618246
RP4-694B14.8	0.270090519	0.588927971	2.324242837	0.022435383	-3.394743262

MIR6130	0.260023242	0.091403435	2.324181677	0.022438818	-3.394865853
CEP57	0.282462808	2.534197174	2.324114569	0.022442588	-3.395000362
HELLS	0.344543818	1.902169713	2.323997641	0.022449158	-3.395234718
ZC3HAV1	0.266663515	3.644597833	2.323996719	0.02244921	-3.395236566
RNU1-78P	0.250950135	0.107129273	2.323733883	0.022463985	-3.395763329
AC093642.5	0.205554867	0.646909192	2.322891372	0.022511403	-3.397451492
TRIM46	0.228319401	0.685275502	2.321029842	0.022616488	-3.401179602
MIR518C	0.200086531	0.11369447	2.320841969	0.022627118	-3.401555713
LINC01548	0.256048612	0.099713938	2.320727468	0.022633599	-3.401784925
RP11-331H2.3	0.239780172	0.116814566	2.320513007	0.022645741	-3.402214213
RP11-711C17.1	0.257401545	0.127498967	2.320462967	0.022648575	-3.402314373
RP11-785D18.3	-0.434565346	1.088740072	-2.320422392	0.022650873	-3.402395586
RP11-38J22.2	0.263534238	0.114993929	2.320058644	0.022671485	-3.403123598
MYO9A	0.291641759	2.047995355	2.320053028	0.022671804	-3.403134836
PTGES3P4	0.343629128	0.568117295	2.320040306	0.022672525	-3.403160298
RP11-24D15.1	0.266798715	0.115364409	2.319971465	0.022676428	-3.403298065
RP11-1094M14.12	0.237431127	0.083672133	2.319591303	0.022697992	-3.404058797
APOL3	-0.443630169	1.665586279	-2.319587119	0.022698229	-3.404067169
PM20D2	0.426275618	2.308950157	2.319160422	0.022722455	-3.404920888
CALM2P2	0.326042077	2.116043224	2.318908833	0.02273675	-3.405424194
AC092155.1	0.25892301	0.647410923	2.318776583	0.022744267	-3.405688744
RNU6-577P	0.211891547	0.242441718	2.318761124	0.022745146	-3.405719667
SLFN11	0.537568175	2.98202603	2.318757503	0.022745351	-3.405726909
GTF3C4	0.273877681	2.677228531	2.318671159	0.022750261	-3.405899621
POLA2	0.235412819	2.483533441	2.31857025	0.022755999	-3.406101459
LRRC23	-0.286643638	1.615906682	-2.318324534	0.022769978	-3.406592908
RN7SL278P	0.240675062	0.128151403	2.318257413	0.022773798	-3.406727145
AC116618.1	0.286587785	0.264473391	2.318148948	0.022779972	-3.406944063
RP11-715G15.2	0.218688687	0.102312024	2.318091267	0.022783256	-3.407059414
LGMNP1	-0.210427636	0.933644351	-2.317997704	0.022788584	-3.407246519
RNU6-413P	0.240521997	0.092549357	2.31744413	0.022820128	-3.408353402
DCAKD	-0.276702097	3.695716723	-2.316315156	0.022884579	-3.410610092
CCNL1	0.302488123	2.59749579	2.3162668	0.022887343	-3.410706728

INPP1	0.295904936	2.710859054	2.316107015	0.022896479	-3.411026038
AC012671.3	0.226423634	0.085702359	2.315598576	0.022925571	-3.412041956
RNU6-104P	0.206530523	0.089499284	2.315024261	0.022958471	-3.413189269
RANBP6	0.316388207	2.490509173	2.314972083	0.022961462	-3.413293493
KIAA0368	0.259042145	3.919348176	2.31487255	0.022967169	-3.413492301
PSMA5	-0.291266337	4.014801618	-2.314774711	0.02297278	-3.413687718
BRWD3	0.254562454	1.695640981	2.314705707	0.022976738	-3.413825538
AP001432.14	0.26308117	0.857142214	2.314439777	0.022991997	-3.414356639
TMEM263	0.286023424	4.786658503	2.314386846	0.022995035	-3.414462345
RP11-567G24.3	0.210144531	0.104619751	2.313934192	0.023021032	-3.415366222
PHLDB1	0.300511344	2.68099568	2.313722306	0.023033209	-3.41578927
RP11-512M8.11	0.273419878	0.319639656	2.313586899	0.023040995	-3.416059604
RP11-478B9.2	0.208718438	0.097318111	2.313443189	0.02304926	-3.416346502
OR7L1P	0.232714052	0.08183324	2.313187964	0.023063945	-3.416855981
PTCH2	0.351338324	1.081690709	2.313169749	0.023064994	-3.41689234
RNU6-216P	0.414867111	0.765757208	2.313113183	0.02306825	-3.417005249
NICN1	-0.237314826	1.935898953	-2.311877661	0.023139469	-3.419470836
AC092106.2	0.201113477	0.366008486	2.311867564	0.023140052	-3.41949098
CTD-2192J16.11	0.225290845	0.32545258	2.311827945	0.023142339	-3.419570024
ZFP90	-0.209209651	2.027655739	-2.311823338	0.023142605	-3.419579217
RNA5SP418	0.319703941	0.107695332	2.311100681	0.023184356	-3.421020776
NBEAP1	0.247158543	0.218700794	2.310597312	0.023213477	-3.422024664
AC195454.1	0.33468466	0.229060905	2.310566495	0.023215261	-3.422086118
TXNDC15	-0.22892317	3.34954917	-2.310369363	0.023226675	-3.42247921
AC079603.1	0.346115697	0.123523601	2.310185824	0.023237307	-3.42284517
AC067742.1	0.235542187	0.160042914	2.310080374	0.023243417	-3.423055418
DIXDC1	0.275666025	1.673110512	2.309951428	0.02325089	-3.423312498
ZNF517	-0.353851807	1.764856253	-2.309277439	0.023289988	-3.424656035
AL365202.1	0.212258259	0.238655219	2.309132657	0.023298395	-3.4249446
MIR6715A	0.230810177	0.099954478	2.308405244	0.023340671	-3.42639417
AC011313.1	0.376229815	0.134699583	2.308238393	0.023350377	-3.426726608
RP11-692M12.5	0.232196974	0.373866258	2.308212567	0.02335188	-3.426778063
DYNLT1	-0.297196482	3.864484369	-2.308187889	0.023353316	-3.426827229

TRDC	-0.606468642	0.872963435	-2.308048109	0.023361451	-3.427105712
AL162151.4	0.220074558	0.085320563	2.307798531	0.023375983	-3.427602904
FAM208B	0.281234252	2.429953892	2.30731407	0.023404215	-3.428567883
AP2A2	-0.223411943	3.16620176	-2.307218492	0.023409788	-3.42875824
RP11-313E4.1	0.266188344	0.169115114	2.307189068	0.023411504	-3.42881684
RP11-53I6.4	0.290702488	0.484149158	2.307164623	0.023412929	-3.428865523
ERBB4	0.291659791	0.455346533	2.307107649	0.023416253	-3.42897899
PTS	0.291679789	2.958090976	2.306616127	0.023444939	-3.429957773
GSTK1	-0.268335211	3.81478724	-2.306095886	0.023475335	-3.430993548
D4S234E	0.335950507	0.381491462	2.305949179	0.023483913	-3.431285599
C21orf33	-0.408829981	1.207532953	-2.305794727	0.023492947	-3.431593049
CTB-191K22.6	0.230827108	0.296338303	2.305772701	0.023494236	-3.431636891
TCP11L2	0.201895042	0.914815797	2.305681041	0.023499598	-3.431819339
CTD-3179P9.1	0.230456965	0.161661498	2.305519446	0.023509056	-3.432140975
MAMDC4	0.258053202	0.810683642	2.305384991	0.023516927	-3.432408577
MIR6728	0.31387224	0.387111499	2.305270484	0.023523633	-3.432636465
SMCHD1	0.337450218	2.423871034	2.304765285	0.023553237	-3.433641783
DTD1	-0.324697124	2.612110659	-2.304756911	0.023553728	-3.433658445
FNBP1P1	0.258353395	0.815123048	2.304710801	0.023556432	-3.43375019
RNU6-644P	0.363713072	0.200844027	2.304680148	0.023558229	-3.433811182
COX14	-0.330342146	4.177346905	-2.304671593	0.023558731	-3.433828203
MIR5698	0.29458362	0.18545316	2.30463415	0.023560927	-3.433902702
RP11-134G8.5	0.227977288	0.963928058	2.303991114	0.023598667	-3.435181963
RP11-272K23.3	0.238760565	0.111463974	2.303917531	0.023602989	-3.435328331
ELP6	-0.242444375	2.880987123	-2.303533503	0.023625557	-3.43609215
IKZF3	-0.431547465	0.692689292	-2.303049868	0.023654005	-3.437053925
KMT2E	0.289616657	2.962306173	2.302982702	0.023657958	-3.43718748
ENTPD7	0.364369275	2.876569071	2.302794063	0.023669064	-3.437562558
RNA5SP79	0.20022645	0.13732178	2.301907608	0.023721315	-3.439324766
TUBA8P2	0.213652355	0.145257335	2.301470652	0.023747109	-3.440193184
RNU1-141P	0.29437695	0.138494332	2.300840402	0.023784355	-3.441445507
AC092289.2	0.294329398	0.141886444	2.300524899	0.023803021	-3.442072308
RNU1-101P	0.267169331	0.133797707	2.300476581	0.02380588	-3.442168292

DBP	-0.273708322	1.236743071	-2.300342587	0.023813812	-3.442434467
URB1	0.344389255	2.581561053	2.300141021	0.023825748	-3.442834845
RP11-114F3.5	0.225218558	0.635964208	2.300050426	0.023831115	-3.443014787
FBXL19-AS1	0.245515262	0.89659194	2.299963927	0.02383624	-3.443186587
MIR4681	0.207483846	0.097133174	2.299600554	0.023857779	-3.443908241
MTR	0.294704796	2.411239427	2.299432018	0.023867775	-3.444242919
LUZP4P1	0.230113507	0.112537844	2.299390233	0.023870254	-3.444325891
RNU6-950P	0.268111702	0.127616098	2.299375913	0.023871104	-3.444354327
RP11-471J7.1	0.277696208	0.581388576	2.29917666	0.023882928	-3.444749965
RBP1	-0.336220153	0.849451152	-2.299169636	0.023883345	-3.44476391
RP11-101O6.2	0.211203189	0.348903046	2.299091288	0.023887996	-3.44491947
MIR3143	0.235359232	0.190237424	2.299081411	0.023888582	-3.44493908
STARD3	-0.216030255	3.342710361	-2.298790834	0.023905839	-3.445515978
ARFGEF2	0.288258181	2.737386941	2.298667864	0.023913145	-3.445760096
RN7SL339P	0.215568539	0.078485503	2.298648509	0.023914295	-3.445798518
HIST1H2BC	-0.553357056	2.595272546	-2.298628905	0.02391546	-3.445837435
CD2	-0.504137566	0.999389328	-2.298573177	0.023918772	-3.445948061
CTSW	-0.447055843	1.216209929	-2.298561027	0.023919494	-3.44597218
SNORD6	0.448290996	1.695875604	2.298463628	0.023925284	-3.446165519
RBM42	-0.313871234	5.196378042	-2.29821145	0.023940279	-3.446666067
GPC5-AS1	0.304922202	0.157415757	2.298188506	0.023941644	-3.446711606
RP11-726G23.6	0.215227215	0.096007875	2.297807184	0.023964336	-3.447468391
PPP2R1B	0.274669033	2.479085385	2.297629001	0.023974946	-3.447821983
AP5B1	0.318889631	2.044579394	2.297357957	0.023991094	-3.448359806
RP11-20B24.3	0.235833713	0.135646627	2.296901721	0.024018296	-3.449264969
KISS1	0.236711884	0.145235958	2.296816132	0.024023402	-3.449434759
RNU6-131P	0.319997158	0.158962105	2.296771883	0.024026042	-3.449522538
AC018902.1	0.502672066	0.400864859	2.296741418	0.02402786	-3.449582971
C1QTNF1-AS1	0.476389509	1.025228799	2.296670475	0.024032094	-3.449723699
TPT1P12	0.220317181	0.393045849	2.296588849	0.024036966	-3.449885612
SNORD11	0.307546071	0.546514021	2.296214619	0.024059314	-3.450627873
MED28P1	0.225453593	0.155099008	2.295960971	0.024074472	-3.451130906
FANCC	0.248357199	2.07067908	2.295659045	0.024092525	-3.45172962

UBE2L4	0.352380504	0.819452348	2.2952234	0.024118596	-3.452593378
APOBEC3C	-0.393265945	4.091034115	-2.295200821	0.024119948	-3.452638141
DNAJC3-AS1	0.343507019	1.209422323	2.295116379	0.024125004	-3.452805548
ARL13B	0.243972306	1.734364713	2.294732255	0.024148018	-3.453567002
RNU7-160P	0.264689507	0.264185299	2.294547262	0.024159108	-3.453933677
NDUFA11	-0.31818211	3.152879907	-2.294310618	0.024173301	-3.454402692
LINC01551	0.323124643	0.202148635	2.294255272	0.024176621	-3.454512378
TMEM208	-0.392938374	4.462580804	-2.294238405	0.024177633	-3.454545804
TMEM42	-0.25974888	1.921529431	-2.294212539	0.024179185	-3.454597065
RP11-449H3.2	0.379974647	0.523165147	2.294135967	0.02418378	-3.454748812
RELN	0.282120261	0.385592202	2.293913737	0.024197121	-3.455189192
RABGAP1L	0.30665136	1.874951326	2.293849579	0.024200973	-3.455316321
CTD-2550O8.7	0.210046341	0.317356888	2.293727257	0.02420832	-3.455558697
CLEC7A	-0.337412928	1.261389759	-2.293395072	0.024228281	-3.45621685
RNU6-147P	0.271454572	0.272519513	2.293378473	0.024229279	-3.456249737
UTP18	0.292915707	3.853084606	2.292824823	0.024262581	-3.457346482
DOCK8	-0.242400402	1.011661877	-2.292821517	0.02426278	-3.45735303
RP11-76N22.1	0.30437646	0.170718522	2.292650359	0.024273084	-3.457692037
RP11-91J19.3	-0.311244741	0.992718613	-2.29260505	0.024275812	-3.457781775
MIR4500HG	0.201377887	0.148087767	2.29254896	0.02427919	-3.457892863
PTBP1	0.232619321	5.427994562	2.292339767	0.024291791	-3.458307158
NDUFA5P2	0.291942933	0.2050309	2.292191087	0.02430075	-3.458601589
MXD4	-0.276573459	3.943101152	-2.291758264	0.024326849	-3.459458616
RP11-301G19.1	0.249166314	0.120058557	2.291676308	0.024331794	-3.45962088
RP11-567M16.2	0.203223691	0.153495543	2.291493215	0.024342844	-3.459983366
LINC01194	0.205415852	0.218857698	2.291201519	0.024360457	-3.460560811
RPL32P28	0.282996999	0.138985582	2.29064975	0.024393804	-3.461652923
RP11-216P6.2	0.235051974	0.219905524	2.290350956	0.02441188	-3.462244226
RP11-528A10.1	0.214949441	0.251715668	2.28954795	0.024460515	-3.463833016
RN7SL822P	0.238218753	0.109306757	2.289219507	0.024480432	-3.464482718
HARS2	-0.258841783	2.982743755	-2.288967372	0.024495732	-3.464981417
AC006534.2	0.463976833	0.326188748	2.288709189	0.024511407	-3.465492028
ZDHHC21	0.252419478	1.132947855	2.288662983	0.024514213	-3.465583405

TTLL11	0.241420302	1.584166304	2.288605533	0.024517703	-3.465697016
MIR527	0.216631271	0.09297596	2.28846205	0.02452642	-3.46598075
WDR25	-0.209161835	1.651195766	-2.288383763	0.024531177	-3.466135554
PHOX2A	0.278128152	0.270302255	2.288047148	0.024551642	-3.466801124
USF1	-0.261939671	4.430237535	-2.287775136	0.024568191	-3.467338894
RNU4ATAC5P	0.343605891	0.145674595	2.286934353	0.024619404	-3.469000776
RP11-288I21.1	0.202758161	0.587567881	2.286585596	0.024640674	-3.469689968
CEP290	0.262020561	1.377483843	2.28657222	0.02464149	-3.4697164
RNU7-2P	0.218948481	0.082587108	2.286520202	0.024644664	-3.469819186
CTNS	-0.305740535	2.810984146	-2.286503142	0.024645705	-3.469852895
SLC25A52	0.246582908	0.258314432	2.286461081	0.024648272	-3.469936005
TK2	-0.269777556	2.283438177	-2.286214459	0.024663328	-3.470423286
LHPP	-0.296972647	1.853828639	-2.28616311	0.024666463	-3.470524737
MIR3197	0.294384909	0.227813402	2.286030078	0.024674589	-3.470787558
RP1-179N16.3	0.209943124	0.346338595	2.286001204	0.024676353	-3.470844601
CTD-2168K21.1	0.213868129	0.112582111	2.285940576	0.024680057	-3.470964375
RNU4ATAC17P	0.2714576	0.094331493	2.285711865	0.024694034	-3.471416177
CTB-31N19.2	0.246399095	0.179425171	2.285542461	0.024704392	-3.471750798
RP11-214K3.22	0.211962939	0.45917524	2.285385536	0.02471399	-3.472060749
SH3TC1	-0.258063885	1.169777841	-2.284805479	0.024749496	-3.473206292
RN7SL402P	0.215091782	0.117087494	2.284688974	0.024756633	-3.473436344
GPR153	0.637911187	4.117648422	2.284650971	0.024758961	-3.473511383
AL590084.1	0.280993177	0.12910914	2.284536902	0.024765951	-3.473736612
GAPDHP68	0.292414657	0.562543222	2.284392206	0.024774821	-3.474022299
AC097721.2	0.238425114	0.459647052	2.284090917	0.024793297	-3.474617113
AC104654.2	-0.34694176	0.664209178	-2.283748076	0.024814337	-3.475293878
RP11-179G8.1	0.249292769	0.102916171	2.283704632	0.024817004	-3.475379629
RP11-666A8.11	0.226734803	0.197980294	2.283696188	0.024817523	-3.475396296
RBL1	0.305078033	2.30360021	2.283582351	0.024824513	-3.475620983
TMEM245	0.267545038	3.042221353	2.283221988	0.024846653	-3.476332194
AC004054.1	0.223179377	0.086815283	2.283191433	0.024848531	-3.476392494
FBXL15	-0.350241715	2.445690605	-2.282349688	0.024900318	-3.478053354
EPOR	0.312287389	1.820712737	2.282305777	0.024903022	-3.478139981

RNU7-11P	0.329649939	0.348168702	2.281884427	0.024928983	-3.478971138
AC006372.6	0.210463058	0.100923354	2.281807391	0.024933732	-3.479123085
EDC3	0.256764377	3.183115965	2.281193455	0.024971607	-3.480333862
RNU6-1122P	0.30455097	0.181430645	2.281126458	0.024975744	-3.480465972
SEC13	-0.250155853	4.685254132	-2.281024416	0.024982045	-3.480667182
FUBP1	0.281675501	4.012186176	2.280873883	0.024991343	-3.480963993
ZNF611	0.245935482	1.729019847	2.280805979	0.024995538	-3.481097877
RP4-533D7.4	0.242294825	0.378570693	2.280627545	0.025006565	-3.481449669
RP11-432M8.6	0.31397992	0.162948506	2.280073366	0.02504084	-3.482542114
MTND4LP1	0.501263876	1.265592704	2.279109494	0.025100552	-3.484441622
RP11-47L3.1	-0.216345076	0.456120791	-2.279083125	0.025102187	-3.484493577
AL121594.1	0.328464224	0.43724046	2.278989339	0.025108004	-3.484678364
RNA5SP385	0.287005798	0.505814588	2.278859064	0.025116087	-3.48493503
NF1	0.229888975	1.750309802	2.278853714	0.025116419	-3.484945571
RP11-452H21.1	0.214297074	0.200098705	2.278847267	0.025116819	-3.484958273
RP1-5O6.5	0.245218001	0.199543776	2.278728829	0.025124169	-3.485191608
ANLN	0.370967701	3.501468403	2.278029127	0.02516763	-3.486569871
RTCB	-0.295280526	4.703362499	-2.277957352	0.025172092	-3.486711233
IST1	-0.22650528	3.706227532	-2.277791593	0.025182399	-3.487037678
C17orf107	0.443997785	1.191224229	2.277529032	0.025198733	-3.487554725
HOXB4	0.337644501	1.450261922	2.277078764	0.025226767	-3.488441291
RP11-996F15.6	-0.21357614	1.034589482	-2.276952157	0.025234654	-3.488690548
CCDC170	-0.280554602	0.867105504	-2.276355632	0.025271846	-3.489864795
C1orf50	-0.229780489	2.355467691	-2.276328275	0.025273552	-3.489918639
APOL6	-0.536448969	2.808083324	-2.275922359	0.025298889	-3.490717514
ARHGAP24	-0.295348686	1.500520959	-2.275385493	0.025332434	-3.491773916
LCLAT1	0.299464691	2.111098753	2.275349879	0.025334661	-3.491843987
ACTR8	-0.278020314	2.568046867	-2.275283508	0.025338811	-3.49197457
SNRPGP14	0.36564208	0.455504464	2.275125574	0.025348689	-3.492285286
RNU6-353P	0.40385228	0.326978679	2.275098484	0.025350383	-3.492338581
CEP95	0.248115108	1.921222104	2.274995077	0.025356853	-3.49254201
CITF22-92A6.1	-0.22406907	1.059320875	-2.274259593	0.025402911	-3.493988664
AC093430.1	0.337793811	0.15589812	2.274241327	0.025404055	-3.494024586

SEC24C	0.303364181	2.765091566	2.273882189	0.025426573	-3.494730835
SMN1	-0.219218583	1.445323972	-2.273782951	0.025432798	-3.494925971
GABPB2	0.334508929	2.272532632	2.272988543	0.02548268	-3.496487777
SMKR1	0.411508998	0.836149188	2.272764733	0.025496749	-3.496927701
RNU5A-7P	0.267138681	0.190058313	2.272696789	0.025501022	-3.497061245
TROVE2	0.329251818	2.546466069	2.272145025	0.02553574	-3.498145608
JAK2	0.362895346	1.871065237	2.272045517	0.025542006	-3.498341145
DDX46	0.228993079	2.729432635	2.271913772	0.025550304	-3.498600013
ACOT11	0.236005685	0.926900458	2.27170515	0.025563448	-3.499009914
RNU6-517P	0.207609471	0.222990166	2.271658415	0.025566394	-3.499101735
RP11-295M3.2	0.206281381	0.234431247	2.271413331	0.025581845	-3.499583225
AC016716.2	0.216981644	0.123459532	2.271068201	0.025603617	-3.50026119
TSEN15	0.317927457	3.742624918	2.271038399	0.025605498	-3.500319728
CHD2	0.2561401	2.427041495	2.270491641	0.025640026	-3.501393566
RNU6-1315P	0.298566047	0.315101389	2.270300037	0.025652135	-3.501769824
SERINC3	0.251141551	5.17388406	2.270273634	0.025653804	-3.50182167
MCM2	0.391956153	4.094345205	2.269981099	0.025672303	-3.502396071
MED21	-0.259751129	2.790982111	-2.269856045	0.025680215	-3.502641598
RAB20	-0.46237702	2.665962634	-2.269797456	0.025683922	-3.502756625
CTC-661I16.2	0.339189484	0.21625731	2.269238342	0.025719325	-3.503854201
GPC3	-0.68681624	1.425985536	-2.269220203	0.025720475	-3.503889806
AL354981.1	0.350564174	0.163405642	2.269058023	0.025730752	-3.504208127
RNU6-256P	0.253834771	0.10760386	2.268853217	0.025743737	-3.504610087
SEC63P2	0.214775451	0.085142115	2.268042605	0.025795184	-3.506200711
RP11-289K10.1	0.219851479	0.349110995	2.26798477	0.025798858	-3.506314179
VKORC1	-0.337662034	4.226086724	-2.267905299	0.025803907	-3.506470091
SAPCD2	0.504481817	2.670417475	2.267731776	0.025814936	-3.506810503
LRRC29	-0.209193415	0.925620987	-2.267608428	0.025822777	-3.507052469
GRIK1-AS1	0.287752527	0.18696389	2.267138083	0.025852699	-3.507975021
AL157392.1	0.223911982	0.209756334	2.267128061	0.025853336	-3.507994676
CCDC71	-0.283188504	3.400113552	-2.267029459	0.025859613	-3.508188056
MRPL40	-0.353622466	3.788794152	-2.266772493	0.025875977	-3.508691986
CSTL1	0.21548646	0.120496066	2.266294753	0.025906424	-3.509628736

CTD-2231E14.2	0.270060723	0.908756823	2.266087641	0.025919633	-3.510034786
CTD-2311M21.5	0.261363586	0.202776852	2.265457819	0.025959838	-3.511269375
KRTAP19-6	0.352424632	0.137744843	2.265301184	0.025969846	-3.511576366
RP11-97C16.1	-0.335069515	1.785266088	-2.265032053	0.025987049	-3.512103798
GS1-259H13.7	0.236273383	0.59241027	2.264987069	0.025989925	-3.51219195
SHFM1	-0.37243206	3.028177266	-2.264827891	0.026000105	-3.512503869
MCM8	0.304449889	2.078237383	2.264627869	0.026012903	-3.512895797
PPFIA4	0.311480653	0.84799842	2.264487479	0.026021888	-3.513170862
RN7SL756P	0.255836326	0.1590838	2.26437348	0.026029187	-3.513394209
AC016727.3	0.333437589	0.194732192	2.264307855	0.026033389	-3.513522776
RP11-321M21.3	0.248951221	0.107254984	2.264027264	0.026051363	-3.514072454
RP11-110I1.12	0.378604342	1.574564994	2.263794817	0.026066262	-3.514527771
EMB	-0.467649021	1.490179409	-2.263744285	0.026069502	-3.514626748
SNRPEP5	0.230660633	0.330571426	2.263627307	0.026077003	-3.514855866
AC004447.2	-0.285987828	2.579853496	-2.263524907	0.026083571	-3.515056421
LZTS1	0.428899091	3.224964508	2.263498249	0.026085281	-3.51510863
RP1-59D14.9	0.330452266	0.205884753	2.263324718	0.026096415	-3.515448477
SERPING1	-0.686200059	4.321669514	-2.263241721	0.026101742	-3.515611013
CCDC173	0.202053219	0.288247082	2.263082471	0.026111965	-3.515922861
NIPBL	0.291020704	2.959367652	2.262615879	0.02614194	-3.516836447
MT-ND4L	0.534869365	11.11427466	2.262380825	0.026157051	-3.517296619
KLRB1	-0.246538757	0.545376816	-2.262265957	0.026164439	-3.517521482
C2CD2L	0.216564651	1.65484272	2.261811834	0.026193664	-3.518410374
CDK5RAP2	0.225182991	2.784498456	2.261629944	0.026205377	-3.518766358
UBXN7	0.252545018	2.788982004	2.261610572	0.026206625	-3.51880427
SMDT1	-0.358214456	3.737564619	-2.261546942	0.026210724	-3.518928795
AC005412.1	0.207895898	0.160361761	2.261396846	0.026220395	-3.519222525
RABGAP1	0.247206614	2.405989393	2.261178768	0.026234452	-3.519649263
RAB3IL1	-0.412046208	3.062394393	-2.261175073	0.02623469	-3.519656493
FERMT1	0.394448916	1.38903157	2.260852081	0.026255522	-3.520288458
ANAPC1	0.203748655	1.472293941	2.260821506	0.026257495	-3.520348279
RNU7-194P	0.342197908	0.29281089	2.260677106	0.026266813	-3.520630781
RN7SL174P	0.337776778	0.152146432	2.260593827	0.026272189	-3.520793701

RP11-819C21.1	0.229925297	0.704610944	2.259678043	0.026331366	-3.522584913
RNU6-600P	0.282659632	0.148108879	2.259551631	0.026339544	-3.522832116
AC005682.6	0.260982821	0.808194656	2.259266925	0.02635797	-3.523388823
TMEM147	-0.347433051	5.085458358	-2.259244308	0.026359434	-3.523433047
KIF18B	0.32643187	3.138309313	2.25900164	0.026375149	-3.523907501
AC096582.7	0.254044072	0.563695815	2.258838731	0.026385704	-3.52422599
AP000870.1	0.200843044	0.219915969	2.258629398	0.026399272	-3.524635208
MIR4664	0.660532823	1.254738838	2.258517008	0.026406559	-3.524854901
AC007511.1	0.288000605	0.103126738	2.257911262	0.026445864	-3.526038814
SLC25A14	-0.250061686	1.734565492	-2.257703508	0.026459357	-3.526444798
RP5-1042I8.7	-0.300188861	1.350582369	-2.257474528	0.026474235	-3.526892225
TSPAN6	-0.339822263	3.39739585	-2.257445554	0.026476118	-3.526948836
LSM5	-0.31053133	2.665377382	-2.257312899	0.026484741	-3.527208023
VTI1BP4	0.209605139	0.200820808	2.257214276	0.026491154	-3.527400707
HPDL	0.481469567	1.563994331	2.257092889	0.026499048	-3.527637855
CTC-461H2.2	0.227891701	0.144689688	2.257017965	0.026503922	-3.527784225
INA	0.504118979	0.858261853	2.256789495	0.026518789	-3.528230536
APAF1	0.260818112	2.144744586	2.256449855	0.026540903	-3.528893941
EIF5A2P1	0.203547118	0.233697496	2.256320585	0.026549324	-3.529146416
HDAC8	-0.203513973	2.085680303	-2.255936709	0.026574346	-3.529896079
AC091801.1	0.205653144	0.102788027	2.255824525	0.026581662	-3.530115141
ARL6IP6	0.337775003	2.621725966	2.255644423	0.026593411	-3.530466804
CTB-49A3.4	0.308898668	0.114834834	2.25539055	0.02660998	-3.53096247
KB-1090H4.2	0.22761527	0.097448279	2.254941535	0.026639308	-3.531839015
RP11-121L11.3	0.206296793	0.08364328	2.254804741	0.026648248	-3.532106027
AC013470.6	0.25233545	0.340182299	2.254677344	0.026656577	-3.532354684
RP11-386G11.5	0.203781786	0.71791148	2.254232334	0.026685687	-3.533223169
PSORS1C3	0.292998342	0.407481232	2.253630207	0.02672512	-3.534398046
ARMC6	-0.29162455	3.30985877	-2.253066688	0.026762071	-3.53549734
MIR3672	0.247807556	0.142614411	2.252839357	0.02677699	-3.53594074
DAB1	0.284777565	0.204725443	2.252000155	0.026832129	-3.537577234
RNA5SP339	0.220379773	0.092061904	2.251824931	0.026843654	-3.537918863
RP11-87P13.2	0.202828584	0.091205912	2.251551235	0.026861665	-3.538452434

MIR520C	0.245899986	0.100522764	2.251358881	0.02687433	-3.538827395
TRANK1	-0.242459601	1.144246186	-2.25130406	0.02687794	-3.538934253
RP11-20O24.1	0.421781658	1.37600198	2.25127729	0.026879703	-3.538986433
HNRNPA0	-0.212464184	4.558570871	-2.251268733	0.026880267	-3.539003112
RP11-51J9.4	0.203581347	0.245377674	2.250488925	0.026931673	-3.540522868
CTC-255N20.1	0.216328296	0.132026302	2.250395818	0.026937816	-3.540704292
RP11-90P5.5	0.215931806	0.236512426	2.250235311	0.02694841	-3.541017034
CLIC3	0.641864332	2.267700679	2.250066324	0.026959567	-3.541346275
NR1H3	-0.254674788	1.921300304	-2.24972614	0.02698204	-3.542009
AC064853.3	0.247237291	0.09982105	2.249405806	0.027003217	-3.542632975
RP11-459K23.2	0.264826248	0.091881661	2.24929421	0.027010597	-3.542850332
GS1-421I3.2	0.232552238	0.112211747	2.249117965	0.027022258	-3.543193589
SFMBT2	-0.25945805	0.913415943	-2.249026669	0.027028299	-3.543371389
BCAS2	-0.297197337	5.049661419	-2.248497853	0.027063319	-3.544401136
RPL35AP4	0.52924108	1.091713565	2.248440446	0.027067123	-3.544512911
RP11-73E17.3	0.260851761	0.222451448	2.248426545	0.027068044	-3.544539976
RP11-864N7.1	0.236877173	0.103279166	2.247995921	0.027096595	-3.545378337
SIRT3	-0.21096392	1.937349214	-2.247785087	0.027110583	-3.54578875
ZW10	0.315613971	3.175066948	2.247732832	0.027114051	-3.545890463
RPF2P1	0.247678109	0.312926951	2.247386614	0.027137037	-3.546564326
POU5F1	0.228055104	0.568737244	2.247187995	0.027150232	-3.546950869
MIR548AY	0.285664087	0.18949339	2.246900169	0.027169363	-3.547510968
UQCRC1	-0.272955618	5.47888152	-2.246786205	0.027176941	-3.54773272
SQRDL	-0.433452212	2.15014417	-2.246754437	0.027179054	-3.547794533
PARD6G-AS1	0.281506866	0.697360415	2.246725434	0.027180983	-3.547850964
RP3-477M7.6	0.226033815	0.263418427	2.246573636	0.027191081	-3.548146313
AC009022.1	0.234267502	0.449558989	2.246570447	0.027191293	-3.548152516
VPS25	-0.290637165	4.377951327	-2.246049337	0.027225984	-3.549166285
ADAMTS10	0.531289366	3.304674641	2.245948252	0.027232718	-3.549362913
NUDT12	-0.231754965	1.086483888	-2.245889436	0.027236637	-3.549477317
TNRC6C-AS1	-0.200496033	0.529987103	-2.245737558	0.027246759	-3.549772723
DCTD	-0.257508805	4.673442867	-2.245605531	0.02725556	-3.550029504
DARS	0.251793536	4.368378375	2.245183686	0.027283699	-3.550849869

CTD-3001H11.1	0.210427913	0.300031274	2.245148624	0.027286038	-3.550918047
AC020934.1	0.206171578	0.150570308	2.244303973	0.02734246	-3.552560213
RP11-295P9.12	-0.219865849	0.551648795	-2.243965153	0.027365122	-3.553218793
RN7SL336P	0.302053388	0.545557435	2.24392218	0.027367997	-3.553302315
RFPL4AP7	0.254308946	0.093386894	2.243808086	0.027375632	-3.553524061
MALSU1	-0.265866837	2.575260364	-2.243577488	0.02739107	-3.553972208
RP11-29O13.1	0.237935961	0.140396245	2.243575069	0.027391232	-3.553976907
HTRA1	-0.682251103	6.641261472	-2.242928619	0.027434551	-3.555233006
PTPN3	0.363799526	1.704345688	2.242916499	0.027435364	-3.555256554
IGHA1	-1.122091004	2.037983538	-2.242743929	0.027446939	-3.555591814
AP000648.5	0.210349534	0.878083803	2.242023317	0.027495317	-3.556991536
DOCK7	0.248292559	2.446857341	2.241698555	0.027517145	-3.557622226
HIRA	-0.277171573	2.528113626	-2.24169835	0.027517158	-3.557622625
PAK2	0.243218751	4.012134233	2.241191526	0.027551253	-3.558606721
FERMT3	-0.557953328	3.311529178	-2.24068064	0.027585658	-3.559598506
AC009961.5	0.323331273	0.308957496	2.240638348	0.027588508	-3.559680598
HSPE1P8	0.27574379	0.483150379	2.240537922	0.027595276	-3.559875529
ADNP2	0.290154685	2.505575192	2.240492045	0.027598368	-3.559964575
AES	-0.354588213	5.23145463	-2.239696887	0.027652014	-3.561507708
RP11-19J3.5	0.213720871	0.278901952	2.23924299	0.027682677	-3.562388353
MCM3AP	0.286888853	3.285885898	2.239216244	0.027684485	-3.56244024
CTD-3020H12.4	0.497907844	0.500760053	2.238812926	0.027711758	-3.56322261
GADD45G	0.461938483	1.922090263	2.238657681	0.027722263	-3.563523728
FAM122B	0.254446657	2.607200153	2.238302256	0.027746325	-3.56421305
FAM107B	-0.290136483	2.326519542	-2.237971949	0.027768703	-3.564853569
CH17-431G21.1	0.208918298	0.749342379	2.237951454	0.027770092	-3.56489331
COX6CP10	0.305864893	0.320879296	2.23739169	0.027808053	-3.565978587
MIR550A3	0.206522277	0.16123674	2.237106708	0.027827397	-3.566531023
KXD1	-0.245375592	3.944421675	-2.237104613	0.027827539	-3.566535082
RP11-88E10.5	0.286534782	0.476141293	2.236603994	0.027861549	-3.567505376
AC104842.1	0.209961373	0.073461237	2.236583593	0.027862936	-3.567544914
ZNF679	0.204885882	0.173570258	2.236219148	0.027887718	-3.568251149
LINC00618	0.276166614	0.436978003	2.235986122	0.027903574	-3.568702665

ELK4	0.245306279	2.475355254	2.235923805	0.027907816	-3.568823404
USP32	0.238822533	2.359503935	2.235858419	0.027912267	-3.568950085
AD000092.3	0.553871123	2.556451468	2.235676581	0.027924649	-3.569302371
SMARCB1	-0.299326214	5.360416359	-2.235639476	0.027927176	-3.569374253
SIL1	-0.332855768	4.007570483	-2.235432548	0.027941274	-3.56977511
SGSH	-0.308827764	2.603018962	-2.235307702	0.027949782	-3.570016944
SHANK1	0.445240704	1.440696608	2.235299192	0.027950362	-3.570033429
RNA5SP71	0.389995052	0.454039062	2.235218332	0.027955874	-3.570190051
NUP205	0.271068595	3.318416264	2.234955075	0.027973827	-3.570699937
KIAA0907	0.310363233	3.056927901	2.234448522	0.028008398	-3.571680899
MCM4	0.368134121	4.718825302	2.233364377	0.028082516	-3.573779736
RNU6-898P	0.304040808	0.145494302	2.232998158	0.028107591	-3.574488509
RP11-72I2.1	0.302983848	0.13929849	2.232988012	0.028108286	-3.574508144
RP11-203F10.1	0.275314722	0.121249116	2.232831714	0.028118994	-3.574810607
RP11-1018N14.5	0.203919634	0.120292078	2.232317166	0.028154272	-3.575806215
AC022182.1	0.262926766	0.258831174	2.232212751	0.028161435	-3.576008225
EGFL8	0.22755832	0.735973542	2.232197615	0.028162474	-3.576037509
RNU2-50P	0.294884157	0.192693108	2.232150187	0.028165728	-3.576129263
ACO1	0.243322177	2.849087557	2.231762126	0.028192368	-3.576879945
RP6-24A23.8	0.384927732	0.196054005	2.231562126	0.028206107	-3.577266789
RP5-1009N12.1	0.226611693	0.499819389	2.231164239	0.028233457	-3.578036301
MAP4K1	-0.327380812	0.876737466	-2.230923158	0.028250039	-3.57850249
AC087294.2	0.207867823	0.449992472	2.230742822	0.028262449	-3.578851186
XXbac-B476C20.13	0.20915293	0.188056922	2.23071439	0.028264406	-3.578906159
RP11-973H7.4	-0.395568486	0.67257243	-2.230308748	0.02829234	-3.579690403
RNU6-1297P	0.378959966	0.539781089	2.230235859	0.028297362	-3.579831309
RP5-1065J22.4	0.208620077	0.645822793	2.229625236	0.028339463	-3.581011581
RP11-333A23.2	0.218327677	0.20416521	2.229622273	0.028339667	-3.581017308
RP11-396N11.1	0.20684046	0.08367383	2.229587259	0.028342083	-3.581084979
RP1-281H8.3	0.202670467	0.311182613	2.229499052	0.02834817	-3.581255447
RP11-304C16.3	0.292025623	0.094778434	2.229442271	0.028352089	-3.581365178
ATP5J2P6	0.221445946	0.155738274	2.229024137	0.028380961	-3.582173166
LINC01169	0.225113645	0.221547145	2.228612282	0.028409425	-3.582968888

MIR4514	0.273920171	0.251881653	2.228414003	0.028423137	-3.583351927
TAF13P2	0.229494045	0.166441136	2.228385149	0.028425133	-3.583407665
CTC-470C15.1	0.353119867	0.367653893	2.228311947	0.028430197	-3.583549068
HADHA	-0.224276031	5.521739455	-2.228017919	0.028450547	-3.584116998
RP11-44D19.1	0.204420453	0.093171172	2.227834618	0.028463239	-3.584471018
MAP3K15	0.206010041	0.209761182	2.227828147	0.028463687	-3.584483516
RNY1P14	0.436539181	0.321711468	2.227540406	0.028483622	-3.585039196
RNU6-1327P	0.270504291	0.179151122	2.227344287	0.028497215	-3.5854179
LINC00903	0.224422995	0.086772454	2.226771961	0.028536919	-3.586522889
SH3BP1	-0.361207604	2.119247078	-2.226639377	0.028546123	-3.586778835
NCR1	0.23297226	0.277418045	2.226537824	0.028553175	-3.586974867
IMPDH1	0.338052849	4.613038051	2.226354418	0.028565915	-3.587328884
INIP	0.233362803	3.317418483	2.226207855	0.028576099	-3.587611765
RPS27L	-0.263013116	2.308761578	-2.226183355	0.028577802	-3.587659051
RNU6-338P	0.308862017	0.147905304	2.225700817	0.028611356	-3.588590275
RP5-1172N10.2	0.220341703	0.208079931	2.225103429	0.028652944	-3.589742896
POLR2L	-0.404741073	6.063144619	-2.225030242	0.028658043	-3.589884088
RP11-88H1.1	0.21077531	0.077651525	2.225024175	0.028658465	-3.589895791
STT3A	0.356299444	5.146453606	2.224987531	0.028661019	-3.589966482
CTD-2571L23.8	0.312827338	0.699271484	2.224861176	0.028669824	-3.59021023
SERF2	-0.367999367	5.813298504	-2.224627797	0.028686094	-3.590660401
SPDL1	0.32542468	2.532378188	2.224482353	0.028696237	-3.590940931
AC067805.1	0.350100502	0.246436692	2.224363432	0.028704534	-3.591170293
RNU6-288P	0.33810143	0.690077808	2.224133344	0.028720591	-3.591614029
ARMC4	0.297645924	0.764108875	2.224034526	0.02872749	-3.591804592
TMSB4XP8	-0.686569705	7.449611641	-2.222892594	0.028807316	-3.59400618
NUS1P1	0.332999908	1.72725665	2.222772112	0.02881575	-3.594238405
WDR35	0.222255998	1.626688279	2.222024589	0.028868124	-3.595678984
GHR	0.43920307	2.340101942	2.221929771	0.028874773	-3.59586168
MSL3P1	0.302645436	0.696315977	2.221851777	0.028880243	-3.596011954
C3	-0.614250363	1.543518363	-2.221810277	0.028883154	-3.596091912
IGSF21	-0.50095242	1.902209741	-2.221773676	0.028885722	-3.596162431
RPL37P25	0.299965611	0.182050277	2.221757511	0.028886856	-3.596193576

RP11-164H5.1	0.258292667	0.338813842	2.22159111	0.028898533	-3.59651416
RP4-756H11.3	0.387534143	1.85026785	2.221093512	0.028933475	-3.597472697
AP000936.5	0.210318975	0.128717756	2.220786304	0.028955066	-3.598064384
CTC-661I16.1	0.238887966	0.125298509	2.220600997	0.028968096	-3.598421256
RP11-30K9.4	0.224493011	0.267494936	2.220538058	0.028972523	-3.598542459
RAPGEF2	0.279556309	2.281501966	2.220387078	0.028983145	-3.598833193
PIEZO2	0.46559003	1.549898703	2.220234643	0.028993872	-3.599126712
EPG5	0.207947954	1.501315747	2.219838739	0.02902175	-3.599888957
AC005783.1	0.221877751	0.29452371	2.219671973	0.0290335	-3.600210001
AC012363.2	0.27481603	0.355106245	2.219435891	0.029050141	-3.600664448
ATXN2L	0.265481462	4.652340344	2.219263658	0.029062287	-3.600995962
AC116609.1	0.286671445	0.118976234	2.219075796	0.02907554	-3.601357534
AC027319.3	0.311546157	0.340639399	2.218882665	0.02908917	-3.601729218
ZNF800	0.212546537	2.068226664	2.218647944	0.029105743	-3.602180905
RNU6-1121P	0.237150398	0.206039455	2.218162182	0.029140067	-3.603115547
SLC2A13	0.217756659	1.343232514	2.217762189	0.029168357	-3.603885029
CAPZA1	-0.269185801	5.459103978	-2.217147234	0.029211898	-3.605067802
RPL12P37	0.331008957	0.408132283	2.217050089	0.029218782	-3.60525462
RUSC2	0.307547107	3.044058211	2.216939319	0.029226632	-3.605467629
ANXA10	0.202124487	0.175616099	2.216710851	0.02924283	-3.605906944
RP11-381N20.1	0.257649772	0.134053247	2.216543624	0.029254691	-3.606228473
RP11-16B13.1	0.210264877	0.185419144	2.216499561	0.029257817	-3.60631319
CXCL11	-0.531527578	0.927713814	-2.216207006	0.02927858	-3.606875629
RP11-363L24.3	0.272196838	0.161914727	2.215883304	0.029301568	-3.607497872
NUP155	0.292446464	3.037534413	2.215840936	0.029304578	-3.607579309
AC013660.1	0.208938136	0.070003327	2.215786	0.029308481	-3.6076849
RP1-167A14.2	0.28710203	0.632973475	2.215463899	0.029331375	-3.608303961
SIK3	0.256230239	2.504164162	2.214387164	0.029408022	-3.610372813
RNY4P25	0.417801862	0.696191804	2.214100778	0.029428438	-3.610922927
ASCL2	-0.26825669	0.619773166	-2.213905373	0.029442375	-3.611298242
FAM102A	0.33366592	3.857022153	2.213769839	0.029452045	-3.611558546
SYNPR-AS1	0.204012959	0.182175221	2.213761358	0.02945265	-3.611574835
DUSP19	0.261698629	1.360669399	2.213459812	0.029474176	-3.612153923

RP11-14K3.4	0.255845194	0.185446793	2.213294151	0.029486007	-3.612472031
GBP2	-0.498911215	2.551265101	-2.212977532	0.029508631	-3.613079952
MIR8059	0.339884154	0.153934418	2.212943581	0.029511058	-3.613145133
KB-173C10.1	0.204097504	0.130791963	2.212819289	0.029519944	-3.613383755
DCAF6	0.270604189	3.262163509	2.212663585	0.029531079	-3.613682665
LDHAP5	0.334160132	1.317480972	2.212561745	0.029538364	-3.613878161
ATXN2	0.259267637	2.421475988	2.212457189	0.029545845	-3.614078862
AK3	0.338516009	3.433980904	2.212448399	0.029546474	-3.614095736
AC016995.3	0.200317415	0.145753848	2.212291957	0.029557671	-3.614396019
RNU2-39P	0.278655444	0.126318	2.212094589	0.029571802	-3.614774829
RP11-456D7.1	0.201325381	0.167944581	2.210885534	0.029658497	-3.617094738
PLEKHH2	0.257241159	0.946296976	2.210816391	0.029663461	-3.617227374
UBE4A	0.336469846	2.790357879	2.210809511	0.029663955	-3.61724057
ABHD11	-0.288632092	2.544412454	-2.210657219	0.029674893	-3.617532697
RP11-459O1.2	0.261030787	0.165566343	2.210637874	0.029676283	-3.617569804
C14orf132	0.500548458	3.296632555	2.210632922	0.029676638	-3.617579302
RNASE2	-0.439195556	0.797278391	-2.210574563	0.029680831	-3.61769124
PAQR3	0.216682607	1.266679045	2.210167016	0.029710123	-3.618472882
SETD8	0.256300559	3.347294299	2.209863495	0.029731955	-3.619054927
IGKC	-1.114003833	1.812079037	-2.209742603	0.029740655	-3.619286737
RP11-166O4.6	0.388795127	0.889882984	2.208931503	0.02979908	-3.620841717
IL17RD	-0.324746161	0.671717731	-2.208761885	0.029811311	-3.621166832
RP11-225B17.2	-0.341885972	2.017239034	-2.208717695	0.029814498	-3.62125153
SFXN1	0.280766669	2.798493868	2.208551251	0.029826505	-3.621570534
RP11-760D2.7	0.209805875	0.517539958	2.208481213	0.029831559	-3.621704762
RABL3	0.222646565	2.090471154	2.208395452	0.029837748	-3.621869117
RP11-390F4.10	0.309249096	0.264160022	2.208181645	0.029853183	-3.622278841
ARPC1B	-0.337802158	5.210956954	-2.208117055	0.029857848	-3.622402609
AC025171.1	0.2322044	1.071319652	2.208062054	0.02986182	-3.622508
IGBP1-AS2	0.20036092	0.318319797	2.207697344	0.029888172	-3.623206787
AC005071.4	0.250549283	0.516286632	2.207449073	0.029906122	-3.623682417
CA11	-0.344658922	1.143620994	-2.206993086	0.029939115	-3.624555863
AC009963.4	0.232375769	0.106308415	2.206686762	0.029961297	-3.625142538

RP11-848P1.4	0.202212433	0.437697736	2.206608972	0.029966933	-3.62529151
RP1-55C23.7	0.210295844	0.08542115	2.206527285	0.029972851	-3.625447941
AC025918.1	0.246353914	0.321969676	2.205992234	0.030011644	-3.626472438
LPCAT4	-0.267213875	1.958440785	-2.205867775	0.030020674	-3.626710715
AC111200.2	0.227118718	0.155407649	2.205597112	0.03004032	-3.627228862
FBXO45	0.228199012	2.40210092	2.205385562	0.030055682	-3.627633805
PRDM14	0.233314086	0.177652923	2.205315849	0.030060747	-3.627767241
RP11-266N13.2	0.201629773	0.177468726	2.205042722	0.030080594	-3.628289988
RP11-113K21.6	0.261850773	0.675676982	2.205031882	0.030081382	-3.628310733
GTF2H5	-0.23056404	2.132070746	-2.204679832	0.030106982	-3.628984446
AC104304.1	0.425294267	0.352922379	2.204567499	0.030115155	-3.629199398
AC026992.1	0.276984788	0.173500165	2.204400733	0.030127292	-3.629518487
RP11-977G19.5	-0.465378854	2.166816866	-2.203691953	0.030178921	-3.63087443
CTD-3025N20.3	-0.312971131	1.26755835	-2.203058062	0.030225161	-3.632086778
ABCD1P5	0.246336866	0.122008499	2.20276028	0.030246904	-3.632656195
RP1-319M7.2	0.207161759	0.150505388	2.202271153	0.030282648	-3.633591351
GPHA2	0.253062982	0.253753586	2.202006516	0.030302003	-3.634097231
FAM120A	0.209463513	4.707980913	2.201804066	0.030316817	-3.634484199
MIR6085	0.322187978	0.189132986	2.201512174	0.030338186	-3.635042071
RHOQP3	0.22233753	0.7071551	2.201120839	0.030366857	-3.635789901
RNU6-225P	0.358388657	0.201518626	2.20107141	0.03037048	-3.63588435
CUL5	0.310773768	2.814158786	2.20089468	0.030383437	-3.63622203
TRAPPC2L	-0.355387018	2.89661408	-2.20087217	0.030385087	-3.636265038
ABCC5-AS1	0.244676785	0.336966729	2.200647759	0.030401548	-3.636693785
FUT4	0.236397361	1.645289588	2.20046914	0.030414655	-3.637035015
UBE2B	-0.202725517	3.621370519	-2.200168504	0.030436727	-3.637609292
LILRA6	-0.204708681	0.584433964	-2.200143557	0.030438559	-3.637656943
PRRC2A	0.350960666	5.206396065	2.200111594	0.030440907	-3.637717994
PRPF3	0.364522522	3.579443989	2.200064932	0.030444334	-3.637807119
AL133493.2	0.222273445	0.370553194	2.199880359	0.030457895	-3.638159642
BCKDHA	0.304851189	1.589494027	2.19983169	0.030461472	-3.638252592
AP001258.5	0.201633584	0.19233618	2.199566638	0.030480957	-3.638758769
SLC25A40	0.261654518	2.035267186	2.199270915	0.030502709	-3.639323454

MIR4419A	0.285097755	0.314484043	2.1992577	0.030503681	-3.639348687
RP11-539L10.3	-0.359412273	1.541712742	-2.199245486	0.03050458	-3.63937201
RPL26P3	0.223111626	0.163331602	2.199067385	0.030517688	-3.639712058
SLC35E3	0.30223066	1.189968869	2.197948653	0.030600137	-3.641847507
AKAP11	0.262108335	2.132479458	2.197775237	0.030612935	-3.642178439
CSF2RA	-0.363780457	1.272645791	-2.197395951	0.030640942	-3.642902156
RNU7-24P	0.242562973	0.089114708	2.19726319	0.030650751	-3.643155451
CTD-3220F14.2	0.244460102	0.650382143	2.197007831	0.030669625	-3.643642613
CYFIP1	-0.220236405	3.496556777	-2.196530834	0.030704907	-3.644552473
ITPR2	0.294876649	2.087114605	2.196471405	0.030709306	-3.64466582
RP11-196G11.4	0.247594086	0.724298043	2.196340679	0.030718983	-3.644915139
MIR762HG	-0.285772788	0.918186966	-2.196338268	0.030719161	-3.644919738
RNU6-1237P	0.41499219	0.564834022	2.196018274	0.03074286	-3.645529973
CCDC39	0.273511621	0.872848853	2.195657451	0.030769602	-3.646217976
CTA-929C8.5	0.278201238	0.094899462	2.195503194	0.030781041	-3.646512075
RP5-1097P24.1	0.287175672	0.091371262	2.195104179	0.030810647	-3.647272736
MIR181B2	0.286772222	0.308737536	2.194850611	0.030829474	-3.647756062
BACE1	0.352669121	3.770087124	2.194788941	0.030834054	-3.647873603
CFAP97	0.296814414	2.688683767	2.194745126	0.030837309	-3.647957112
SCAPER	0.202246866	1.037861301	2.194723605	0.030838908	-3.64799813
CEP170B	0.376786021	3.156559214	2.194596162	0.030848377	-3.648241017
METTL16	0.315525733	2.64344307	2.194532663	0.030853095	-3.648362033
AC069082.1	0.226647188	0.066963942	2.193191842	0.030952883	-3.650916616
COX7A2L	-0.241437755	3.945605595	-2.193080543	0.030961179	-3.651128605
HLA-C	-0.478136049	7.546278821	-2.192747246	0.030986033	-3.651763374
AL162430.1	0.25930445	0.103975279	2.192648931	0.030993368	-3.651950599
CTB-41I6.2	-0.203437025	0.484503407	-2.192642559	0.030993843	-3.651962732
RP11-25I15.1	0.38448492	1.224601243	2.19254682	0.031000987	-3.652145046
RP11-60I3.4	0.236454581	0.159565477	2.192476867	0.031006208	-3.652278249
RP5-1116H23.5	0.20764913	0.27407293	2.1923454	0.031016022	-3.65252858
STAC3	-0.643786334	1.840615487	-2.192168722	0.031029216	-3.652864974
AC023818.1	0.296794131	0.119157626	2.192129249	0.031032164	-3.652940128
RNA5SP465	0.393642303	0.433082533	2.192107712	0.031033773	-3.652981133

RP11-785H20.1	0.239288373	0.114771265	2.191914528	0.031048206	-3.653348919
CEP152	0.236259967	1.271734993	2.191764819	0.031059395	-3.653633918
XRN1	0.285536043	1.917812426	2.191141372	0.031106029	-3.654820577
AC093166.4	0.265104741	0.241192256	2.191018427	0.031115233	-3.655054554
RP11-368I23.2	0.21142526	0.158880256	2.190941984	0.031120957	-3.655200027
IGHVIII-67-2	0.268972753	0.105763801	2.190886038	0.031125146	-3.65530649
IFNAR1	0.264961062	3.976411401	2.190735824	0.031136397	-3.655592331
HEATR5B	0.21293236	2.135428895	2.190565952	0.031149125	-3.655915557
SDCCAG3P2	0.51396012	1.075475269	2.190456817	0.031157305	-3.656123205
CCDC163P	0.218652644	1.165276888	2.190311709	0.031168183	-3.656399283
CTD-2538G9.3	0.259960969	0.130781976	2.19021704	0.031175282	-3.656579387
RNU6-453P	0.328641969	0.121862484	2.189722789	0.031212368	-3.657519574
PCOLCE	-0.468696522	7.674801728	-2.18949958	0.031229129	-3.657944111
RNU7-110P	0.222600891	0.21540649	2.189337266	0.031241322	-3.658252803
TRPV4	0.479555661	2.480686893	2.189160436	0.03125461	-3.658589078
RN7SL735P	0.25035988	0.266518811	2.188880871	0.031275629	-3.659120675
AC092332.1	0.250023808	0.096242601	2.188094647	0.031334806	-3.660615368
C1orf56	0.330488227	2.425498302	2.188034013	0.031339374	-3.66073062
SCARA5	0.681203968	1.71489784	2.187986155	0.03134298	-3.660821585
KLHL8	0.276048621	2.551058679	2.187952474	0.031345518	-3.660885602
SLC31A1	0.24984536	4.09599059	2.187740505	0.031361494	-3.661288474
RN7SL549P	0.278448765	0.105715197	2.187597668	0.031372263	-3.661559931
AC020931.1	-0.214960088	0.285460971	-2.18757182	0.031374212	-3.661609052
MLLT10P2	0.231359403	0.092395664	2.187107989	0.031409208	-3.662490433
RP11-328P23.2	0.262955084	0.496170443	2.186719031	0.031438581	-3.663229413
SLC18B1	-0.341648586	2.631888076	-2.186705335	0.031439615	-3.663255431
COG5	0.217791612	2.167717533	2.186064789	0.031488041	-3.664472138
NOS1	0.270224177	0.196755052	2.186063889	0.031488109	-3.664473848
RP11-192C21.3	0.398623097	0.304513977	2.185631993	0.031520797	-3.665294048
POM121	0.289568616	2.471057544	2.185444803	0.031534974	-3.665649492
RP1-8B22.2	0.219762676	0.14147606	2.184923759	0.031574464	-3.666638724
ARHGEF11	0.292237949	3.476022145	2.184797437	0.031584045	-3.666878522
HSBP1L1	0.322416993	1.646184401	2.184553673	0.03160254	-3.667341227

NEU4	0.280557466	0.217873579	2.184152391	0.031633006	-3.66810283
DENND4C	0.234433989	1.919425339	2.1833652	0.031692847	-3.669596494
ERMN	0.458128992	1.215477315	2.183358672	0.031693343	-3.669608878
DDAH2	-0.357289672	4.193163912	-2.183124767	0.031711144	-3.67005261
FTLP2	-0.390413813	2.651109052	-2.182922958	0.031726508	-3.670435422
RP11-794C22.2	0.213302927	0.196013453	2.18283959	0.031732857	-3.670593554
RP13-324D24.1	0.2354399	0.111536586	2.182766754	0.031738405	-3.670731704
CWC25	-0.204824993	2.580580283	-2.182297616	0.03177416	-3.67162143
C11orf97	0.2669972	0.101827608	2.182184701	0.03178277	-3.671835551
LINC01517	0.352517644	0.314544223	2.181979943	0.03179839	-3.672223808
RNU6-212P	0.433430429	0.525008207	2.181803568	0.031811851	-3.672558219
FAM20B	0.330713112	3.678804122	2.181598829	0.031827481	-3.672946378
ARHGAP30	-0.38711818	1.962592477	-2.181501856	0.031834887	-3.673130215
TMEM238	0.435288294	1.059373456	2.18135576	0.031846047	-3.673407166
SLC38A6	-0.234285835	1.536821533	-2.181126074	0.0318636	-3.67384254
FEM1A	0.249799343	0.977738541	2.180939666	0.031877851	-3.67419585
C2CD4D	0.38990829	0.669377841	2.180894517	0.031881304	-3.674281421
LINC01036	0.27318636	0.347139886	2.180815644	0.031887336	-3.674430903
BTBD7P1	0.234832684	0.339351008	2.180710313	0.031895393	-3.674630521
RN7SL323P	0.231198061	0.140032666	2.180579817	0.031905378	-3.674877819
RP11-452I5.2	0.350915213	0.786368797	2.180475259	0.031913381	-3.675075953
AC079807.2	0.33180057	1.479006921	2.180408146	0.031918518	-3.675203125
AC133106.2	0.273093231	0.450086761	2.180292027	0.031927408	-3.675423152
RP11-666E17.1	0.246058653	0.147420043	2.18020402	0.031934148	-3.675589904
AF213884.3	0.324357459	0.13054291	2.17990042	0.031957406	-3.676165106
TRIM58	0.62322632	1.150677921	2.1796895	0.031973573	-3.676564674
HSPE1P10	0.225302841	0.123085893	2.17968224	0.03197413	-3.676578428
STON1	0.360299475	2.726723073	2.179360677	0.031998792	-3.67718753
RP13-923O23.6	0.233188023	0.163035513	2.179300275	0.032003426	-3.677301935
MIR3677	0.442866342	0.638310779	2.178902998	0.032033922	-3.678054327
RP11-331H13.1	0.241360053	0.362885249	2.178844601	0.032038407	-3.678164913
RP11-70O5.2	0.20899322	0.310545523	2.178833007	0.032039297	-3.678186869
RNU6-370P	0.286295825	0.18939794	2.178608184	0.032056569	-3.67861259

SLC4A8	0.261154016	0.624074357	2.178605181	0.0320568	-3.678618276
AQP11	0.363070752	0.956704222	2.178347905	0.032076575	-3.679105398
MIR3129	0.720292038	1.680753763	2.178300683	0.032080205	-3.679194803
RNU6-681P	0.209686951	0.097299298	2.17816055	0.032090982	-3.679460103
RNA5SP319	0.257862237	0.189838139	2.17776621	0.032121324	-3.680206586
RNU6-424P	0.216951873	0.07865747	2.177068285	0.032175087	-3.681527462
RP11-3G20.2	0.30616364	0.236228036	2.17696245	0.032183247	-3.681727729
NCRNA00250	0.238727823	0.145351584	2.176577641	0.03221293	-3.682455818
RP11-108O10.2	0.207654636	0.262038392	2.176021176	0.032255896	-3.683508487
AL354680.1	0.210201271	0.113672546	2.175908535	0.032264599	-3.683721544
SNORD36	0.207533397	0.061316686	2.175868794	0.03226767	-3.68379671
TMPO	0.32390614	3.60787878	2.175379637	0.032305493	-3.684721798
AC021192.1	0.225142965	0.093020287	2.17529091	0.032312357	-3.684889578
ILDR2	0.49849036	1.171750371	2.174963173	0.032337725	-3.685509265
RPS26P43	0.304411654	0.275577938	2.174456857	0.032376949	-3.686466449
AC011499.1	0.250200239	0.167426735	2.174181726	0.03239828	-3.686986498
RN7SL574P	0.253389103	0.439289728	2.173717073	0.032434334	-3.687864645
RN7SL425P	0.245151081	0.320547103	2.173631515	0.032440976	-3.688026323
OCEL1	-0.23089246	2.504437811	-2.173111025	0.032481411	-3.689009764
RP3-408N23.4	0.218700787	0.267625237	2.17285194	0.032501554	-3.689499214
RNU6-83P	0.301791138	0.14547474	2.172735803	0.032510588	-3.689718598
RN7SL189P	0.279676677	0.099286551	2.172722485	0.032511624	-3.689743755
ESPN	0.455102955	0.698535667	2.172532963	0.03252637	-3.69010174
SLC33A1	0.225623293	2.37050297	2.172107829	0.032559469	-3.690904665
RNA5SP485	0.483892076	0.176821254	2.171592131	0.032599659	-3.691878447
PAIP1	0.264154587	4.110784893	2.171523694	0.032604995	-3.692007659
TMEM194A	0.299849451	3.061980989	2.171063127	0.03264093	-3.69287714
HDLBP	0.310211231	5.880035312	2.170901361	0.03265356	-3.693182491
RP1-144C9.2	0.210570698	0.108929117	2.170361582	0.032695733	-3.694201238
RP11-361M10.3	0.23530872	0.291645911	2.169925306	0.032729854	-3.695024473
NCBP1	0.245539458	3.454156052	2.169743507	0.032744082	-3.695367477
ATP11A	0.388979885	1.862204951	2.168992718	0.032802896	-3.696783737
RP1-159A19.4	0.238653366	0.388337957	2.168457204	0.032844903	-3.697793644

RP11-616M22.7	0.264356453	0.10303005	2.168397267	0.032849607	-3.697906665
ANXA5	-0.238939033	7.430334133	-2.168290729	0.032857971	-3.69810755
PAK1IP1	-0.261977441	3.650937324	-2.168234779	0.032862364	-3.698213044
MFI2-AS1	0.507694525	2.325102452	2.16812025	0.032871358	-3.69842898
ESYT3	0.237848319	0.695127781	2.166969378	0.032961856	-3.700598322
RHEBP3	0.392820712	0.316025673	2.16694016	0.032964156	-3.700653383
SQLE	0.494670826	3.998839397	2.166181005	0.033023974	-3.702083778
SMG1	0.206784982	2.065820596	2.166027044	0.033036117	-3.702373816
CNPY3	-0.383638308	5.582899146	-2.165813772	0.033052945	-3.702775558
C1orf112	0.224903737	1.577627206	2.165801728	0.033053895	-3.702798244
AP000251.3	0.292522782	0.72461322	2.165375422	0.033087554	-3.703601166
AC087174.1	0.209730474	0.061965822	2.164956039	0.033120696	-3.704390914
RP11-254G11.1	0.212124027	0.514143338	2.16490388	0.03312482	-3.704489126
ANP32BP3	0.213830647	0.253902016	2.164750056	0.033136984	-3.704778754
RP11-292K15.2	0.215825586	0.096016946	2.164668643	0.033143424	-3.704932035
MAGEA11	-0.513443203	0.722738984	-2.163961157	0.033199431	-3.706263849
CKAP2L	0.330327196	2.49117653	2.163766867	0.033214826	-3.706629524
SLC13A3	0.332335159	0.538786937	2.163696376	0.033220413	-3.706762188
RP11-505P4.5	0.204049069	0.104936816	2.163614165	0.033226931	-3.706916905
CAV1	-0.597896999	3.907260584	-2.163481176	0.033237475	-3.707167173
MIR548L	0.200815461	0.235833063	2.162966115	0.033278342	-3.708136319
MIR2909	0.215003359	0.210779926	2.162847975	0.033287722	-3.708358585
GRM6	0.208038594	0.334423007	2.162610924	0.03330655	-3.708804533
ARL8B	-0.267717072	4.205027507	-2.162193893	0.033339695	-3.70958896
ZNF114	0.337907606	0.903705418	2.162094028	0.033347637	-3.709776785
CEP19	0.280655805	1.79263746	2.161680626	0.033380529	-3.71055422
RP11-649A16.1	0.259465678	0.264272085	2.161634146	0.033384229	-3.710641622
PNN	0.300788503	4.17584528	2.161562636	0.033389922	-3.710776087
SDCBP	-0.310759871	5.10582362	-2.161352016	0.033406694	-3.711172104
NAA20	-0.262043987	4.156040355	-2.16127351	0.033412948	-3.711319707
MIR199A1	0.304913852	0.458610113	2.161064056	0.033429638	-3.711713487
RNA5SP206	0.328699527	0.124379571	2.160498799	0.033474716	-3.712776015
HSPA13	0.390065746	3.834329926	2.160368355	0.033485126	-3.713021179

RNA5SP341	0.226654169	0.143855358	2.160294354	0.033491033	-3.713160255
CDC73	0.291811808	2.857565453	2.159910963	0.03352165	-3.713880724
PLEKHO1	-0.316839531	3.678340758	-2.159842617	0.033527111	-3.714009147
RP11-349A22.3	0.206308157	0.121709596	2.159567568	0.033549094	-3.714525937
CTD-2015A6.2	0.262171863	0.408444671	2.158952923	0.033598265	-3.715680578
PSMB6	-0.305887401	5.694908362	-2.158919261	0.03360096	-3.715743806
RNU6-333P	0.25222097	0.100077802	2.158887904	0.03360347	-3.715802703
RAMP2	-0.506441252	3.959738748	-2.157799801	0.033690683	-3.717845988
STK24	0.325616221	2.406390089	2.157659878	0.033701912	-3.718108675
C1orf189	0.246237738	0.335333021	2.155850465	0.033847417	-3.721504243
LYRM5	-0.299049756	2.500546649	-2.155456912	0.033879137	-3.722242453
MIR4797	0.292496514	0.156706854	2.155402596	0.033883516	-3.722344328
TIMM23B	0.224864016	1.73372254	2.154774002	0.03393424	-3.723523145
FBXL3	0.303450472	2.575956721	2.154755003	0.033935774	-3.72355877
RP11-2J18.1	0.215333709	0.41228687	2.154523541	0.033954469	-3.723992755
RN7SL170P	0.275580045	0.111864262	2.154424292	0.033962488	-3.724178832
FES	-0.300648264	2.628296707	-2.154081323	0.033990211	-3.724821787
NIM1K	0.224247012	0.511544754	2.153836713	0.034009996	-3.725280297
RP11-675M1.2	0.300758402	0.628289185	2.153569393	0.034031628	-3.725781321
RNA5SP292	0.203065662	0.078363568	2.153428403	0.034043043	-3.72604555
C1orf52	-0.254108002	2.935695647	-2.153133612	0.03406692	-3.726597965
EIF4EBP1P1	0.229058567	0.336048469	2.152520545	0.034116622	-3.72774659
ZNF341-AS1	0.293621333	0.449470967	2.152426988	0.034124212	-3.727921852
RBM41	0.228574699	1.452531134	2.151953509	0.034162649	-3.728808714
RP11-680F8.1	0.307838612	0.7204248	2.151887397	0.034168019	-3.728932534
CTD-2522B17.6	0.240085646	0.105177311	2.151779755	0.034176763	-3.729134127
BLVRA	-0.305780053	4.180450946	-2.150753408	0.03426024	-3.731055822
CCP110	0.231693999	1.562083329	2.150538973	0.034277703	-3.731457219
BMP8A	0.615013218	2.162895443	2.15040194	0.034288867	-3.731713711
FADS2	0.566625055	4.298520524	2.150363283	0.034292016	-3.731786064
RP11-94B19.5	0.271154933	0.102569996	2.14966406	0.034349034	-3.733094585
FP236383.3	1.23651432	5.564734695	2.149496337	0.034362723	-3.733408405
RASAL3	-0.296666736	1.227781931	-2.149390016	0.034371403	-3.733607327

CHCHD7		-0.268485089	2.128711213	-2.149249703	0.034382862	-3.733869832
CA3		1.31072317	4.47945461	2.149127599	0.034392835	-3.734098257
RNF216P1		-0.23490451	2.182329906	-2.148955964	0.034406859	-3.734419325
RP11-575A19.2		-0.295510725	0.665954916	-2.148885965	0.03441258	-3.734550262
RNU6-1247P		0.356615827	0.155210562	2.148553812	0.034439738	-3.735171518
RNA5SP125		0.2936638	0.199987809	2.148223603	0.034466755	-3.735789053
HSPG2		0.490311447	5.214632825	2.147817612	0.034499998	-3.736548195
RP11-227D13.5		0.227810603	0.137000158	2.147729995	0.034507176	-3.736712009
UBXN7-AS1		0.242983314	0.342043321	2.147421339	0.034532472	-3.737289043
PPAPDC1B		0.354796213	3.264027203	2.147017387	0.034565603	-3.738044122
RNU6-471P		0.220876651	0.072619759	2.146724737	0.034589622	-3.738591073
JRKL		0.298555897	2.13389824	2.146702794	0.034591423	-3.73863208
NUTF2P6		0.249450233	0.456083408	2.146611335	0.034598933	-3.738802999
KCTD20		0.24901965	4.396043937	2.146507685	0.034607446	-3.73899669
NREP		-0.409387484	3.656222016	-2.146450656	0.03461213	-3.739103259
UBQLN1		0.212802372	3.991211249	2.146264919	0.034627391	-3.73945032
KANSL1L		0.221395619	1.199626088	2.146094052	0.034641435	-3.739769571
SSR3		0.28679922	5.801790889	2.145861104	0.034660589	-3.740204779
LIMS2		-0.265073059	0.94525676	-2.145535143	0.034687407	-3.74081369
AC008169.1		0.232940656	0.091111391	2.145110687	0.034722356	-3.741606472
KCNK15		0.243188847	0.467509023	2.144611953	0.034763459	-3.742537808
FERMT2		0.310409993	3.786615891	2.144280208	0.034790824	-3.743157204
KIAA1377		0.247093587	0.846390205	2.1438521	0.034826164	-3.743956389
STAG1		0.290233446	2.09414185	2.143375961	0.034865507	-3.74484507
PSMA1		-0.253768774	4.378997755	-2.143059027	0.034891716	-3.745436509
ZNF460		0.347201807	1.474344167	2.1427878	0.034914159	-3.745942591
CISH		-0.283349474	1.415144823	-2.142252239	0.034958511	-3.746941725
RNU5E-10P		0.34023029	0.536391577	2.142178364	0.034964632	-3.747079529
	1-Sep	-0.22387355	0.799148121	-2.142117882	0.034969645	-3.747192346
GAR1		-0.2696522	4.013816592	-2.141966921	0.034982159	-3.747473919
ACA59		0.213853721	0.351334867	2.141697909	0.035004469	-3.747975642
RP11-855A2.1		0.251571001	0.73923246	2.141587399	0.035013637	-3.748181732
AC064846.1		0.200527001	0.064985415	2.141107954	0.035053438	-3.749075743

SYTL2	0.492873017	2.725684527	2.140321311	0.035118825	-3.75054219
FAM63B	0.333919041	2.486624739	2.140244282	0.035125234	-3.750685761
MED14	0.285866636	3.203217997	2.139473395	0.035189425	-3.752122324
RP11-843P14.1	0.211109284	0.552029509	2.138763826	0.0352486	-3.753444214
IDH3G	-0.28981961	4.54638988	-2.138562234	0.035265428	-3.753819698
AC007919.19	0.220855182	0.129371431	2.138521168	0.035268856	-3.753896183
AL161757.1	0.241144943	0.089371504	2.138507215	0.035270021	-3.75392217
RNA5SP209	0.279683782	0.139552869	2.138376732	0.035280918	-3.754165185
CFL1P2	0.28435515	1.064688726	2.138182201	0.035297169	-3.754527459
RNU6-845P	0.236018095	0.08953946	2.13805899	0.035307466	-3.754756899
PRICKLE1	-0.390580504	1.028867434	-2.137716923	0.035336065	-3.755393826
AC079907.1	0.439778078	0.954740962	2.137359205	0.035365994	-3.756059796
PTPRD-AS1	0.279264048	0.989496247	2.137104761	0.035387296	-3.75653344
GS1-309P15.3	0.255769282	0.319762163	2.136834532	0.035409932	-3.757036411
LRIG1	-0.553043553	1.458285434	-2.136766481	0.035415634	-3.757163063
LLNLR-304A6.1	0.223379294	0.350262592	2.136669714	0.035423744	-3.757343154
RNU6-497P	0.29875642	0.191200215	2.136616758	0.035428183	-3.757441707
TMEM161B-AS1	-0.209436546	0.99602696	-2.136114887	0.035470274	-3.758375594
RNU6-176P	0.315928705	0.227043272	2.136099906	0.035471531	-3.758403467
KLHL4	-0.366235827	0.592373754	-2.136066937	0.035474298	-3.75846481
ZNF863P	0.20058843	0.107261408	2.135142169	0.035551978	-3.76018508
SH3BGRL	-0.312424228	5.305417619	-2.134881573	0.035573895	-3.760669726
PROSER1	0.24035159	2.288621363	2.134681779	0.035590706	-3.761041259
RP11-71L14.3	0.340122027	0.643163818	2.134592016	0.035598261	-3.761208171
AL080243.1	0.281148349	0.182095982	2.134545292	0.035602194	-3.76129505
MIR516B1	0.200676418	0.085547736	2.134306109	0.035622334	-3.761739765
AC097724.3	0.207822564	0.555552805	2.134240846	0.035627832	-3.761861101
ANKRD9	0.352566448	2.428640594	2.13409611	0.035640025	-3.762130179
HCG25	0.235423539	0.857919128	2.133780336	0.035666641	-3.76271718
RP11-339A7.1	0.239828204	0.240964928	2.133214712	0.035714359	-3.763768439
PPIH	-0.305265623	3.999176737	-2.133171542	0.035718004	-3.763848664
SNORA70	0.368647287	0.549872946	2.133075996	0.03572607	-3.764026217
RN7SKP55	0.310421825	0.169875578	2.132942488	0.035737345	-3.764274301

NAPSB		-0.299890614	0.615195646	-2.132573542	0.035768518	-3.764959806
UTP14C		0.229577372	2.362827912	2.13222329	0.035798133	-3.76561048
RP11-259N19.1		-0.355817411	1.469509959	-2.131515887	0.035858012	-3.766924351
ZSCAN12		0.221811523	1.082516846	2.130945122	0.035906388	-3.767984161
EPHA6		0.23034303	0.312567858	2.130843848	0.035914978	-3.768172182
FBXO4		0.253718765	1.932683131	2.13063488	0.035932707	-3.768560119
RP6-24A23.3		0.553360013	0.367233355	2.129579844	0.036022335	-3.770518213
RN7SL503P		0.225783832	0.229859309	2.129430912	0.036035003	-3.770794553
RNU4-42P		0.412888242	0.232993524	2.128870913	0.036082669	-3.771833466
AC008622.1		0.400462842	0.354270023	2.128530131	0.036111703	-3.772465569
RP11-381I15.1		0.214969622	0.067193487	2.128455448	0.036118068	-3.772604081
NDUFA3P2		0.23604365	0.391976738	2.128390646	0.036123592	-3.772724266
	10-Sep	0.329144437	3.498014079	2.128211083	0.036138903	-3.773057274
AC093673.5		-0.389831566	2.68742522	-2.128082861	0.036149839	-3.773295052
C9orf40		0.328985728	2.66037841	2.12793507	0.036162449	-3.773569103
S1PR2		-0.288324885	2.656637986	-2.127702431	0.036182305	-3.774000457
MIR876		0.218637724	0.093974938	2.127593157	0.036191635	-3.774203055
RNU7-20P		0.419730366	0.147770216	2.1275626	0.036194244	-3.774259707
SLC25A17		-0.229561285	3.004004293	-2.127320156	0.036214953	-3.774709168
MIR100		0.20343717	0.094230824	2.127117014	0.036232312	-3.775085733
RP11-7N14.1		0.296090975	0.126819502	2.126966548	0.036245175	-3.775364631
RNU6-1057P		0.25698938	0.276191705	2.126875417	0.036252968	-3.775533541
MIR4790		0.251582982	0.150827989	2.126789285	0.036260334	-3.775693178
EPHA8		0.21237482	0.119422452	2.126472832	0.036287409	-3.776279644
GPX1P2		-0.201218042	0.672700057	-2.126193663	0.036311309	-3.776796947
RP11-561N12.6		0.488404163	0.860023915	2.126102596	0.036319108	-3.776965683
RP1-96H9.5		0.37633203	0.84582319	2.125895628	0.036336839	-3.777349145
LEMD3		0.240483106	2.688840556	2.125630513	0.036359562	-3.77784029
BATF3		-0.23348205	0.751287616	-2.125599393	0.036362231	-3.777897938
RNU7-174P		0.637634606	0.512528576	2.125560833	0.036365537	-3.777969368
CTA-228A9.3		0.565052724	1.437903802	2.125257421	0.036391561	-3.778531376
RTP4		-0.386045687	1.192056052	-2.125166113	0.036399396	-3.778700492
UGGT1		0.24811137	3.929714021	2.124651285	0.036443599	-3.779653905

NDUFC2		-0.286078406	3.370988257	-2.123784434	0.036518133	-3.781258768
RP11-1079K10.1		0.213519836	0.13271937	2.12369859	0.036525521	-3.781417665
RNU6-1300P		-0.322805608	0.569768258	-2.122781684	0.036604516	-3.783114501
RP11-114H24.6		-0.320222483	0.688632825	-2.122618741	0.036618569	-3.783415976
DNER		0.220508074	0.192212194	2.122604787	0.036619773	-3.783441793
CHAF1B		0.376811503	2.640147507	2.122222912	0.036652729	-3.784148248
CLIP1		0.213352343	1.997675831	2.12160793	0.036705855	-3.785285707
AP003025.2		0.260421886	0.144073747	2.121590404	0.03670737	-3.785318119
MIR519B		0.248607149	0.119912818	2.121536671	0.036712015	-3.785417488
	8-Sep	-0.235915055	3.221953843	-2.121293791	0.036733018	-3.785866618
RP5-1053E7.2		0.2376135	0.105942319	2.120616108	0.036791676	-3.78711954
ERGIC3		-0.268000142	5.152860045	-2.12040811	0.036809696	-3.787504021
PRKDC		0.358923289	4.601246892	2.120285395	0.036820331	-3.787730843
PSME1		-0.358938602	5.548408973	-2.12000265	0.036844845	-3.788253412
AC079756.1		0.238337055	0.11323148	2.119948689	0.036849525	-3.788353136
HIP1R		-0.263100746	1.233548755	-2.119827733	0.036860017	-3.788576664
FICD		0.224592552	1.622426273	2.119820009	0.036860687	-3.788590937
STAG3L5P		0.233667976	1.085238387	2.119420541	0.036895358	-3.78932907
COPB2		0.225552933	4.523363937	2.118872545	0.036942966	-3.790341452
TRAPPC6A		-0.38970267	3.451790423	-2.118230739	0.036998791	-3.791526843
TEX22		0.224660457	0.395871151	2.118102267	0.037009975	-3.791764087
GALNT2		0.292917424	3.691614621	2.118095373	0.037010575	-3.791776818
AMICA1		-0.218113015	0.532908899	-2.117979324	0.03702068	-3.79199111
RN7SL149P		0.207111484	0.109450947	2.117063492	0.037100507	-3.793681881
RNA5SP177		0.274502855	0.127693134	2.117057963	0.03710099	-3.793692086
PRRC2C		0.34467276	4.377107599	2.116950543	0.037110363	-3.793890358
DTX4		0.580227594	3.738595252	2.116363681	0.037161606	-3.794973403
IRS4		0.630119982	0.385157734	2.116186223	0.037177114	-3.795300847
AC012168.1		0.220609366	0.134262436	2.116039503	0.037189939	-3.795571554
RNU6-46P		0.248320463	0.204436453	2.115619759	0.037226652	-3.796345915
SSR1P1		0.251073603	0.084267829	2.11463093	0.037313264	-3.798169605
RSF1-IT1		0.278595083	0.434078271	2.114291726	0.037343016	-3.798795022
RHCE		0.33158345	1.356289588	2.114263443	0.037345497	-3.798847166

OLFM3	-0.349042663	0.419848282	-2.114172473	0.03735348	-3.799014876
RNU6-612P	0.228547793	0.098179361	2.114004002	0.037368268	-3.79932545
ATP5E	-0.333922012	4.707354695	-2.113980445	0.037370336	-3.799368875
LRP5	0.346184916	3.289898037	2.113166647	0.037441842	-3.800868772
LINC01420	-0.364265101	4.314157121	-2.112538134	0.037497149	-3.802026822
GLIPR2	-0.396271771	2.813850704	-2.112348687	0.037513834	-3.802375822
AC018693.5	0.207046751	0.099191425	2.112332654	0.037515246	-3.802405356
RAB1A	-0.232447983	5.8202623	-2.11188191	0.03755497	-3.8032356
CMKLR1	-0.420754341	2.220547974	-2.111581537	0.037581462	-3.803788782
HEATR6	0.208069659	2.325969579	2.111543575	0.037584812	-3.803858691
BCL9L	0.382817391	2.809903889	2.111374309	0.037599749	-3.804170382
COBLL1	0.225318494	0.622503266	2.110792517	0.037651129	-3.805241545
AC092170.1	0.201002828	0.132025834	2.110764408	0.037653613	-3.805293291
ZNF664	0.346672914	4.411208236	2.110506271	0.037676431	-3.805768469
HMGN2P18	0.242179536	0.261926129	2.110374763	0.03768806	-3.806010527
RP11-178L8.9	0.211012484	0.434356059	2.110265674	0.037697709	-3.806211312
EVI2B	-0.510215674	2.875843971	-2.110242489	0.03769976	-3.806253984
SOWAHC	0.248602284	1.840715757	2.110218984	0.037701839	-3.806297244
RP11-448N11.2	0.246780048	0.165233511	2.110139139	0.037708903	-3.806444193
CTD-2036J7.1	0.201424221	0.250374742	2.109813923	0.037737689	-3.807042681
TCF23	0.218352498	0.229838873	2.109247959	0.037787829	-3.808084018
RC3H1-IT1	0.303661924	0.583761829	2.108968039	0.037812649	-3.808598961
IGLC3	-0.739599333	1.047147361	-2.108866428	0.037821662	-3.808785869
AC093376.1	0.204557556	0.105791135	2.108745028	0.037832433	-3.809009168
POFUT2	0.292493514	3.895842697	2.108517803	0.0378526	-3.809427087
RP11-18B16.2	0.498160259	1.788735886	2.108406336	0.037862497	-3.809632086
PLAA	0.260795933	3.013668406	2.107577003	0.037936199	-3.811157009
MIEN1	-0.309828712	3.260898166	-2.107431593	0.037949135	-3.811424324
EPRS	0.315613295	4.784316459	2.107187729	0.037970837	-3.811872595
AC093816.1	0.273498635	0.144256856	2.106971706	0.03799007	-3.812269649
RNU6-967P	0.214266181	0.068207963	2.106742252	0.038010509	-3.812691352
HIGD1AP9	0.320681275	0.460997697	2.106355093	0.038045016	-3.813402797
C15orf61	0.220007702	1.458799284	2.106342511	0.038046138	-3.813425916

NUDT2	-0.372767161	3.409798865	-2.106290398	0.038050785	-3.81352167
MRPS31P4	0.20974807	1.094206389	2.105495624	0.038121718	-3.814981746
MICAL1	-0.286908657	2.149781693	-2.105278358	0.038141128	-3.815380799
AL353805.1	0.301347682	0.328851833	2.10498976	0.038166925	-3.815910812
AL121899.1	0.263657272	0.225620036	2.104764704	0.038187052	-3.816324082
ZBTB34	0.2349898	2.034310103	2.104431966	0.038216826	-3.816935018
RNU6-859P	0.226229708	0.160580799	2.104380091	0.03822147	-3.817030257
NES	0.577839879	5.63630695	2.104289527	0.038229579	-3.817196522
AC116035.1	0.249767768	0.147684335	2.103331176	0.038315472	-3.818955547
RN7SKP65	0.205863759	0.111435533	2.103326047	0.038315932	-3.81896496
NEURL1	0.3276793	0.796661319	2.103008617	0.038344419	-3.819547434
EFS	0.573093923	4.083116864	2.102798335	0.0383633	-3.819933251
FAM72B	0.204030743	1.166231404	2.101666287	0.038465086	-3.8220097
HARS	-0.225673503	2.850224012	-2.101224847	0.03850484	-3.822819136
AP3S1	-0.208902364	4.108833497	-2.100960071	0.038528702	-3.823304562
SIN3A	0.236899425	2.65171624	2.10078527	0.038544462	-3.823625003
SNORD59	0.412344734	0.230318857	2.100670894	0.038554777	-3.823834663
GIMAP8	-0.366634739	1.836158949	-2.100447474	0.038574934	-3.824244176
MTUS1	-0.274544615	1.556830268	-2.100136879	0.03860297	-3.824813411
RP11-384C4.2	0.245320466	0.480917758	2.099977393	0.038617373	-3.825105677
RNU7-169P	0.304123687	0.172450159	2.099913598	0.038623136	-3.825222579
OLMALINC	0.313429025	1.802294764	2.099754713	0.038637491	-3.825513714
PTPLB	0.269304345	3.409939138	2.099641638	0.03864771	-3.825720896
RNA5SP276	0.205386136	0.072636095	2.099391762	0.038670301	-3.826178699
MBTD1	0.208479578	1.761282324	2.098872018	0.038717326	-3.827130776
TMEM185B	0.268077475	3.650227166	2.098436329	0.038756785	-3.827928718
SPINT2	-0.420863463	1.229968632	-2.09824491	0.038774132	-3.828279245
GPD1L	-0.222978978	1.983619206	-2.098203847	0.038777854	-3.828354436
RP11-511B23.1	0.209700763	0.108509813	2.098125222	0.038784982	-3.828498403
IL2RG	-0.475925894	1.812272764	-2.097855458	0.038809446	-3.828992321
RP11-295D4.3	0.210586047	0.713687175	2.097646771	0.03882838	-3.829374373
RP11-73M18.10	0.318153285	1.799764452	2.097646767	0.038828381	-3.829374381
BNIP3P35	0.216342677	0.104669823	2.097601711	0.03883247	-3.829456862

RP11-785H5.1	-0.313860594	3.084352955	-2.097433613	0.038847728	-3.829764574
ANKRD10-IT1	0.492611053	2.836583865	2.09729027	0.038860744	-3.830026955
LRRC8D	-0.359348556	1.869695941	-2.09715661	0.038872884	-3.830271597
PRR15	0.355684603	0.465968248	2.097151931	0.03887331	-3.830280161
RP3-522P13.1	0.239634692	0.219934131	2.096934422	0.038893073	-3.830678243
RNU6-1165P	0.328097222	0.520660469	2.096663879	0.038917667	-3.831173336
CEP97	0.226919341	1.412406051	2.096598721	0.038923592	-3.831292566
RNU7-29P	0.207555035	0.086272703	2.096395355	0.03894209	-3.831664678
6-Sep	-0.30942366	2.249772435	-2.096253636	0.038954986	-3.83192397
AL022237.3	0.200272758	0.185847458	2.095591497	0.039015284	-3.83313523
AC073842.19	0.202218493	0.155505001	2.095571991	0.039017062	-3.833170907
ZNF83	0.245796459	2.129942374	2.095453712	0.039027842	-3.833387238
AC100821.1	0.288745487	0.268483338	2.095193823	0.039051538	-3.833862534
MIR1254-2	0.257803497	0.084058439	2.09487202	0.039080896	-3.834450989
AC136932.2	0.309563152	0.12466021	2.094681977	0.039098243	-3.834798467
A2ML1-AS1	0.209605704	0.101219777	2.094664043	0.03909988	-3.834831257
BCR	-0.244821392	1.865478921	-2.094521334	0.039112911	-3.835092168
RELT	0.278175299	2.10764443	2.094388062	0.039125084	-3.83533581
RNU6-857P	0.305679387	0.157825007	2.094293868	0.039133689	-3.835508005
MYO10	0.33129339	3.729823849	2.094238867	0.039138715	-3.835608548
MAF	-0.483997561	3.85520898	-2.094178019	0.039144275	-3.835719777
KLF12	0.281068115	1.727125635	2.094120027	0.039149575	-3.835825781
MEF2D	0.294827406	4.178930197	2.094060677	0.039155	-3.835934267
RP11-360D2.2	0.334109151	0.974063541	2.093987531	0.039161687	-3.836067965
EIF3F	-0.275783462	3.913145979	-2.09384317	0.039174886	-3.836331819
WWTR1-IT1	0.351837942	0.694418671	2.093621231	0.039195187	-3.836737435
MELK	0.316743562	3.378895331	2.093492469	0.039206969	-3.836972743
PAXBP1	0.282326572	2.361045631	2.092853086	0.03926552	-3.838141001
RP6-24A23.7	0.693204283	0.436437206	2.092683958	0.03928102	-3.838449972
AC016722.3	0.233973389	0.139833418	2.09211841	0.03933289	-3.83948298
IFT57	-0.276687345	2.564185292	-2.091988874	0.039344778	-3.83971955
ADD3	0.314898336	2.89696586	2.091951375	0.039348221	-3.839788031
CTD-2313J17.3	0.204170606	0.183638453	2.091883664	0.039354437	-3.839911684

AP000547.1		0.249774653	0.102723098	2.091757734	0.039366	-3.840141646
IFITM10		-0.502586355	2.321288258	-2.091752427	0.039366488	-3.840151336
CHEK1		0.300037669	2.395338127	2.091711493	0.039370247	-3.840226083
RNU6-659P		0.286508185	0.12749511	2.091506713	0.039389058	-3.840599999
RNU6-776P		0.264968124	0.092058749	2.091388857	0.039399888	-3.840815182
RP11-350N15.4		0.455688589	1.798051087	2.091308105	0.03940731	-3.840962614
MLANA		0.286239919	0.72899444	2.091002089	0.039435447	-3.841521273
MIR8054		0.21715666	0.12197979	2.09055188	0.039476874	-3.842343036
LPHN2		-0.418882982	3.322282937	-2.090155361	0.039513391	-3.843066666
RP11-444D13.1		0.22379818	0.153792892	2.090071003	0.039521164	-3.8432206
COCH		0.744175869	1.366680343	2.089931954	0.039533979	-3.843474321
ASB13		-0.239092359	2.499978306	-2.08981964	0.039544332	-3.843679248
TRBV20-1		-0.296670067	0.368769214	-2.089705157	0.039554888	-3.843888122
IVD		-0.235318832	2.984277409	-2.08955908	0.039568361	-3.844154624
FLJ20021		-0.311042089	2.500231001	-2.089280954	0.039594023	-3.84466199
WDR4		0.289176554	3.056845893	2.088785348	0.039639787	-3.845565938
CETN2		-0.255371914	3.993872635	-2.088348057	0.039680204	-3.846363365
SDF2		-0.264119206	3.43143286	-2.087836058	0.039727572	-3.847296838
ZNF713		0.2539847	0.868623936	2.087632121	0.039746453	-3.847668596
	2-Mar	-0.333034232	2.768926837	-2.087233666	0.039783365	-3.848394851
ITGB1		0.400048573	6.006735313	2.086642996	0.039838138	-3.849471221
NPM1P21		0.216892357	0.625708162	2.086594402	0.039842647	-3.849559761
RP11-438L7.1		0.205538106	0.082791951	2.086572837	0.039844648	-3.849599054
AP000997.1		0.301859106	0.33633686	2.086536633	0.039848007	-3.849665016
AC108479.3		0.315660888	0.834621419	2.086403344	0.039860379	-3.849907859
TMEM184A		0.248293286	0.310321205	2.086020986	0.039895887	-3.850604408
FASN		0.414132176	4.716801095	2.085994725	0.039898327	-3.850652244
GPIHBP1		-0.415895581	1.300285102	-2.085876219	0.039909338	-3.850868103
LMO2		-0.370275626	2.427076418	-2.085444064	0.039949515	-3.851655181
RNU1-87P		0.273516389	0.293118574	2.08528385	0.039964418	-3.85194694
PCP4L1		0.496473329	1.250335352	2.084965954	0.039994004	-3.852525786
RP1-200K18.1		0.238021576	0.153500151	2.084814639	0.040008094	-3.852801285
PHRF1		0.229319028	3.330988189	2.084540198	0.040033658	-3.85330091

RPUSD3	-0.241219885	2.139545162	-2.083907902	0.040092612	-3.854451791
HYOU1	0.32846824	4.917939723	2.083286527	0.04015062	-3.855582492
LPGAT1	0.253310073	2.631277551	2.083049851	0.040172734	-3.856013085
RP13-152O15.5	0.246172511	0.700226496	2.082782532	0.040197723	-3.856499376
IGLC2	-0.937054347	1.608406268	-2.082771982	0.04019871	-3.856518567
EXOSC4	-0.369701043	4.301961409	-2.082287542	0.040244031	-3.857399685
RP11-179A7.2	0.201014807	0.136006383	2.081698641	0.040299185	-3.858470551
RP4-612C19.1	0.202491952	0.275843694	2.081540926	0.040313967	-3.858757298
MIR1302-6	0.230619456	0.093489536	2.081401729	0.040327017	-3.85901036
RNA5SP42	0.208174289	0.104909886	2.081149452	0.040350678	-3.859468963
CTC-250I14.1	0.296613927	0.915349213	2.080928832	0.04037138	-3.859869976
AC008686.1	0.233311276	0.49855085	2.080521571	0.040409619	-3.860610142
RP11-173M1.4	0.244330081	0.34118394	2.080470808	0.040414388	-3.860702391
RP11-803B1.3	0.211453366	0.229097677	2.080413882	0.040419736	-3.860805838
CTC-339F2.2	0.250442061	0.672275397	2.080355847	0.040425189	-3.860911296
IPO9	0.246712016	3.228552581	2.080233779	0.04043666	-3.861133105
RP4-657E11.10	0.414784101	2.20181081	2.080129242	0.040446486	-3.861323048
UHMK1	0.26350345	3.310714382	2.079250017	0.040529212	-3.86292026
RBM47	-0.292591407	1.32465518	-2.079007288	0.040552076	-3.863361099
TRIM62	-0.235406648	1.67495758	-2.078968966	0.040555686	-3.863430693
ZNF330	-0.268063606	3.45259383	-2.078968299	0.040555749	-3.863431905
RN7SL612P	0.310887668	0.206581915	2.07881348	0.040570339	-3.863713055
CTD-2189E23.1	0.214736049	0.195593892	2.078688376	0.040582132	-3.863940228
SNORA2B	0.253850555	0.563417191	2.078549078	0.040595267	-3.864193161
AC090666.1	0.255777828	0.220272913	2.078514034	0.040598572	-3.864256791
RP11-169D4.2	0.553421087	0.763823434	2.077935523	0.040653163	-3.865307059
AL132640.1	0.262815198	0.120452739	2.077297853	0.040713411	-3.866464423
RP11-577B7.1	0.244927798	0.138798019	2.077100545	0.040732068	-3.866822471
RNU6-899P	0.255053809	0.128416788	2.076998162	0.040741752	-3.867008249
LMO7	0.403568936	2.946476332	2.076956213	0.040745721	-3.867084365
RP11-1263C18.1	0.211123571	0.107790974	2.076504623	0.040788463	-3.867903683
NDUFS3	-0.297501057	3.716688269	-2.076474864	0.040791281	-3.867957669
MIR583	0.211273119	0.120149994	2.075431719	0.040890167	-3.869849607

AP003385.2	0.200065603	0.0899147	2.075409102	0.040892313	-3.869890617
ST3GAL5	-0.335987798	2.087609128	-2.075386787	0.040894431	-3.86993108
DENND1B	0.244932966	1.094376576	2.075317163	0.040901039	-3.870057323
ASCC3	0.219205823	2.240462709	2.074868403	0.040943652	-3.87087093
CTB-186G2.1	0.237895745	0.201689376	2.07482189	0.040948071	-3.87095525
CIZ1	0.250861152	4.584552265	2.074742047	0.040955658	-3.871099986
MRPL23	-0.417490882	2.632361139	-2.074627123	0.04096658	-3.871308307
AC113133.2	0.223540181	0.090674016	2.074599383	0.040969217	-3.87135859
COQ9	-0.228372634	2.978981591	-2.074351047	0.040992827	-3.871808706
IL17RA	-0.317999966	1.731881662	-2.074113337	0.041015439	-3.872239517
RN7SL824P	0.284599484	0.12788931	2.074065745	0.041019967	-3.872325763
ZNF347	0.208898963	1.309802617	2.073969813	0.041029097	-3.872499609
CFAP36	-0.251013952	2.652959233	-2.073770934	0.041048028	-3.872859989
FAM168B	0.251063481	4.747701237	2.073675962	0.041057071	-3.873032073
JMJD1C	0.267170163	2.376655079	2.073399335	0.041083421	-3.873533263
ACTA2	-0.75240135	4.390479298	-2.073324324	0.041090569	-3.873669156
NFATC2IP	0.202937151	3.071984864	2.073024147	0.041119183	-3.87421293
SLA	-0.349035057	1.485195339	-2.072186958	0.041199078	-3.875729131
RP11-315D16.4	0.22671035	0.161222811	2.071606675	0.041254534	-3.876779736
CYP11A1	0.378642338	0.613716849	2.071472403	0.041267375	-3.877022799
RP11-291H24.1	0.405812656	0.610145899	2.071352937	0.041278803	-3.877239047
FKSG48	0.492136088	1.041438808	2.071334852	0.041280534	-3.877271783
RP11-328L11.1	0.409264917	0.98830877	2.071195158	0.041293901	-3.87752463
TOR1AIP1	0.282315009	2.9343257	2.070926173	0.04131965	-3.878011454
TRAPPC8	0.254283324	2.246299619	2.070798672	0.04133186	-3.878242191
HNRNPCP1	-0.257682329	1.569027367	-2.070164185	0.041392668	-3.879390229
SNRPEP7	0.200890345	0.160560613	2.070148437	0.041394178	-3.879418719
RNF2	0.238024887	2.887040774	2.069473494	0.04145895	-3.880639604
HDGF	0.273091116	6.31976796	2.069432758	0.041462862	-3.880713278
RP11-5P18.10	0.241616909	0.421326366	2.069087592	0.041496023	-3.881337489
U2SURP	0.292465541	2.89312028	2.068997406	0.041504691	-3.881500569
MAFK	0.333558792	2.90959964	2.068886441	0.041515358	-3.881701215
NDUFS5	-0.385862324	7.978552677	-2.068715605	0.041531786	-3.882010099

MIR4491	0.379664109	0.130880296	2.068557862	0.041546959	-3.882295291
TNR	0.4745878	0.46228536	2.068407422	0.041561435	-3.88256726
PLK2	-0.416791693	3.46995206	-2.06822556	0.04157894	-3.882896011
MIR5699	0.226102076	0.259121886	2.068141781	0.041587006	-3.88304745
RNU7-147P	0.263796928	0.159368817	2.06786097	0.041614052	-3.883555002
RRM1-AS1	0.210222172	0.278666095	2.067765359	0.041623264	-3.883727799
RNU1-33P	0.332886691	0.201977466	2.0673346	0.041664789	-3.884506223
AL590762.6	0.273754634	0.805224694	2.066719992	0.041724099	-3.885616626
NFXL1	0.21536023	1.624827568	2.066489509	0.041746359	-3.886032961
RP11-568J23.1	0.270858911	1.061563309	2.066470704	0.041748176	-3.886066927
CCNT1	0.240760365	2.641328747	2.066386152	0.041756345	-3.886219645
RP4-697K14.3	0.222175462	0.127081217	2.066194158	0.0417749	-3.886566407
PHOSPHO1	1.024598476	5.228589497	2.06604823	0.041789008	-3.88682995
UFM1	0.245259319	3.684286254	2.06540064	0.041851663	-3.887999278
MFSD3	-0.347732619	2.987369144	-2.065250072	0.041866242	-3.888271107
RP11-545G3.2	0.210479604	0.258052609	2.065201902	0.041870907	-3.888358066
PRPF40A	0.259185553	3.355659601	2.064840023	0.041905969	-3.889011299
CERS6	0.351548499	2.247578474	2.064523422	0.041936664	-3.889582714
AC104301.2	0.276959687	0.158960549	2.064517711	0.041937218	-3.88959302
SH3PXD2A	0.324528169	4.342607916	2.064384526	0.041950136	-3.889833374
DAPK1-IT1	0.217175271	0.185300481	2.06432659	0.041955757	-3.889937926
DARS2	0.340139785	3.332050378	2.064287755	0.041959525	-3.890008005
RNU6-1188P	0.383212211	0.337998509	2.06415752	0.041972163	-3.890243013
RP11-413N13.1	0.258348474	0.107040031	2.063972628	0.041990111	-3.890576624
VIM	-0.277390433	9.105173711	-2.06324534	0.042060774	-3.891888654
RNU7-156P	0.225738549	0.099024196	2.062970086	0.042087544	-3.892385104
AC021052.1	0.460955014	0.253799089	2.062882937	0.042096023	-3.892542273
CACNA2D4	-0.346283441	1.219166373	-2.06283619	0.042100572	-3.892626577
LINC01549	0.769958319	2.30125148	2.062508255	0.042132493	-3.893217933
IRX5	0.491259018	3.021082659	2.062179287	0.042164536	-3.893811065
RPS5P2	0.226049242	0.309750549	2.06191405	0.042190387	-3.894289227
DSTNP1	0.250373477	1.124822327	2.061540177	0.042226849	-3.894963142
KDELC2	0.394649212	4.235388731	2.061058905	0.042273824	-3.895830485

MPHOSPH6	-0.274787598	2.596574261	-2.060564174	0.04232216	-3.896721893
DNM1L	-0.220240788	3.393361624	-2.060201084	0.042357665	-3.897375989
FOXP1	-0.390294826	2.070986468	-2.060159862	0.042361698	-3.897450243
SP2	0.20264518	3.495232458	2.059913855	0.04238577	-3.89789335
ADAM9	0.365360112	3.75908356	2.059909997	0.042386148	-3.897900298
CRYBA1	0.225973948	0.195771768	2.059822898	0.042394673	-3.898057168
B9D1	-0.344179071	1.779021722	-2.059663903	0.042410241	-3.898343513
VAT1	-0.287328149	5.540498047	-2.059548635	0.042421529	-3.898551093
AC107399.2	0.265144884	0.151308173	2.059251258	0.042450665	-3.89908658
RP11-145M9.6	0.345160781	0.88997799	2.058927039	0.042482451	-3.89967032
CTD-2267D19.3	-0.251806497	1.036276077	-2.05862142	0.042512432	-3.900220498
FLYWCH1	0.215843882	2.3069651	2.058407211	0.042533456	-3.900606073
SLC46A1	0.231947556	1.183894747	2.058331387	0.0425409	-3.900742547
ACTL6A	0.254196485	3.829215548	2.057960447	0.042577334	-3.901410132
KB-1507C5.3	0.27427623	0.336043701	2.057018341	0.042669989	-3.903105164
HIVEP2	0.265424256	1.931353639	2.056513218	0.042719738	-3.904013691
AC072062.1	0.268265814	0.893187932	2.056410466	0.042729865	-3.90419848
GLRX	-0.327995971	2.237609559	-2.05632826	0.042737967	-3.904346312
LINC01540	-0.281510152	0.98826198	-2.055950182	0.042775251	-3.905026146
BOLA3P4	0.234707478	0.096370312	2.055927917	0.042777447	-3.905066177
ARTN	0.321795528	0.807442165	2.055861963	0.042783954	-3.90518476
ECSCR	-0.381602026	1.755866744	-2.055787196	0.042791332	-3.905319183
TYMSOS	0.334487258	1.291035649	2.055522544	0.042817455	-3.905794961
RP11-12K11.1	0.20824325	0.289499296	2.054691732	0.042899551	-3.907288198
SERPINA1	-0.527542533	1.978497778	-2.054584708	0.042910137	-3.907480514
RPL36A	-0.39697955	3.888132144	-2.05390401	0.042977515	-3.908703486
PSD	-0.370522055	1.303138305	-2.053734929	0.042994265	-3.909007207
RGS9	0.213762663	0.455857118	2.053703146	0.042997414	-3.909064297
RN7SKP78	0.262411502	0.56754125	2.05350286	0.043017265	-3.90942404
USP39	-0.208084191	4.002894301	-2.053105826	0.043056638	-3.910137074
ZNF37BP	0.209570465	1.099223817	2.052828389	0.043084169	-3.91063525
LINC00393	0.283987385	0.281392853	2.051994596	0.043167001	-3.912132078
MRPL27	-0.316608584	3.639811352	-2.051969756	0.043169471	-3.912176661

HSD17B14	-0.392257557	2.465867135	-2.051530015	0.043213214	-3.912965857
RP11-243M5.2	0.223564886	0.427268094	2.050461245	0.043319688	-3.914883323
GJA1P1	0.375140056	1.569374278	2.050461035	0.043319709	-3.914883701
RP11-999E24.3	-0.419869072	1.360718622	-2.050434237	0.043322381	-3.914931766
RNU6-387P	0.433155077	1.234744855	2.050241712	0.043341586	-3.915277073
RIC8A	-0.208335347	4.034788187	-2.050237619	0.043341994	-3.915284415
SIRT6	-0.305532948	2.917364308	-2.050086027	0.043357121	-3.915556286
AC079776.2	0.503388731	0.916173057	2.049933511	0.043372345	-3.915829794
RP11-1079K10.2	0.287483308	0.519814886	2.049801246	0.043385551	-3.916066972
LA16c-361A3.3	0.234974994	0.917766318	2.049182983	0.043447327	-3.917175458
HMGCS1	0.360261747	3.166003768	2.049080755	0.043457549	-3.917358714
SEPT4-AS1	0.205142301	0.622227735	2.048657591	0.043499884	-3.9181172
SNORA2A	0.299255416	0.800746255	2.048224688	0.043543229	-3.918892995
MBD2	0.253322225	3.450270822	2.048064382	0.04355929	-3.919180238
C1QTNF7	-0.437236736	0.761029447	-2.047221938	0.043643774	-3.920689435
SNORA55	0.308316328	0.911792296	2.047183583	0.043647624	-3.920758132
MIR1205	0.318263627	0.426753062	2.046809574	0.04368518	-3.92142796
RP11-677O4.5	0.244547314	0.112709637	2.046193788	0.043747073	-3.922530557
MAD1L1	-0.285589489	3.063163999	-2.04604806	0.043761732	-3.922791446
HLCS	0.249797935	2.299770035	2.045994812	0.043767089	-3.922886769
AL354764.1	0.22060893	0.108905655	2.045859598	0.043780695	-3.923128815
TIMM22	-0.242800715	3.169163449	-2.045762653	0.043790452	-3.923302348
AL133230.1	0.247365091	0.209893642	2.045586759	0.043808161	-3.923617182
SC5D	0.356172794	2.203986898	2.044674911	0.043900061	-3.925248908
PATL1	0.221760968	3.715393385	2.044550955	0.043912566	-3.925470672
USH1C	0.250776001	0.260679449	2.044411518	0.043926637	-3.925720119
LA16c-83F12.6	0.246347497	0.297951331	2.044391019	0.043928706	-3.925756789
ZNF830	-0.230122773	2.607351226	-2.044365282	0.043931304	-3.92580283
FAM105A	-0.305696643	1.572195527	-2.044198796	0.043948111	-3.926100641
PTGER1	0.32787568	0.602432817	2.043867169	0.043981607	-3.926693791
ZNF702P	0.230146915	0.758436333	2.043863539	0.043981973	-3.926700284
FAM86DP	-0.241880206	1.667544023	-2.043763207	0.043992112	-3.926879721
AC002075.4	0.44144319	1.747938334	2.043717081	0.043996773	-3.926962212

RP11-728F11.4	0.219409224	0.472768327	2.043589196	0.044009699	-3.927190909
NADK2	0.244571342	2.814283478	2.043235737	0.044045444	-3.927822936
RP1-228P16.7	0.223017281	0.761826162	2.042491849	0.044120752	-3.929152775
AC009305.2	0.203116713	0.143055973	2.04239408	0.044130658	-3.929327522
ETV5	-0.333545604	2.438453311	-2.041854168	0.044185396	-3.9302924
RPS15AP16	0.300159953	0.848384901	2.041697938	0.044201246	-3.930571556
ADAMTSL2	0.382803648	1.881141241	2.041347055	0.044236862	-3.931198455
CTC-444N24.8	-0.215476162	2.282463679	-2.041272587	0.044244424	-3.93133149
MIR520E	0.247394688	0.118904392	2.040987543	0.04427338	-3.93184067
RP11-598P20.3	0.457360569	3.317239243	2.040575896	0.044315224	-3.932575889
CTA-276O3.4	0.535909904	2.533195699	2.040484251	0.044324545	-3.932739553
RP11-66B24.5	0.209166602	0.15677231	2.040269684	0.044346374	-3.933122711
ZXDC	0.211552205	2.039525986	2.04021317	0.044352125	-3.933223623
TCF19	0.386407848	3.902401078	2.040119404	0.044361668	-3.933391048
MYO6	0.475775419	2.810460367	2.03999739	0.044374088	-3.933608902
RNA5SP100	0.211217225	0.065417252	2.039457434	0.04442909	-3.934572839
RNU6-358P	0.401745458	0.466597203	2.039336587	0.044441408	-3.934788546
RP11-589M2.1	0.212594094	0.209200247	2.039169444	0.04445845	-3.935086869
STX18	-0.272103735	2.760663122	-2.039055568	0.044470064	-3.935290108
DLX2	0.484608231	1.238926593	2.038663664	0.044510053	-3.935989472
RP11-242O24.3	0.231762365	0.28075639	2.038600118	0.04451654	-3.93610286
ZCCHC11	0.218216574	1.91509546	2.03824859	0.04455244	-3.936730052
UBE2V2P2	0.215582486	0.154166364	2.037833698	0.044594843	-3.937470171
GPR124	0.432579949	4.316617357	2.037193128	0.044660378	-3.938612609
RNU6-628P	0.355902182	0.296442522	2.037043942	0.044675653	-3.938878631
KIAA1161	0.444346406	2.700177821	2.036778578	0.044702834	-3.939351774
LRP2BP	0.235005754	0.485209841	2.036358136	0.044745929	-3.940101307
LYZ	-0.643747181	3.823243207	-2.036296612	0.044752238	-3.940210975
INPP4A	0.295130553	2.36031282	2.035910244	0.044791877	-3.940899621
NDUFS7	-0.24949372	2.701708761	-2.035756519	0.044807656	-3.941173581
RP11-113K21.1	0.212833776	0.164643358	2.035383265	0.044845989	-3.941838698
BAG4	0.280474266	2.557014884	2.035345103	0.04484991	-3.941906693
AL132709.3	0.318941766	0.116312839	2.034590198	0.044927531	-3.943251528

TAGAP	-0.215431763	0.82021428	-2.034430989	0.044943916	-3.943535094
SMO	0.324926593	4.310583883	2.034403001	0.044946797	-3.943584943
RNU6-106P	0.319977176	0.359489162	2.034369354	0.04495026	-3.943644868
BAIAP2-AS1	-0.285187728	2.347870542	-2.034288148	0.044958621	-3.943789492
P2RY11	-0.271210161	1.981912476	-2.033828158	0.045006002	-3.944608618
CYSTM1	-0.351974256	3.253197011	-2.033365269	0.045053726	-3.945432735
LINC00087	-0.204326055	0.524963038	-2.033279395	0.045062584	-3.945585605
CYCSP5	0.244550066	0.099762972	2.032788773	0.045113223	-3.946458882
ASXL1	0.22653741	3.15459958	2.032696181	0.045122785	-3.946623668
AL354749.1	0.229636972	0.074800743	2.032354811	0.045158054	-3.947231149
RP11-134L4.2	0.283604566	0.122442425	2.031806697	0.045214732	-3.948206345
RP1-120G22.11	0.435553656	1.022482355	2.031134445	0.045284331	-3.949402081
RP11-104N10.2	0.258949231	1.070823868	2.030744579	0.045324736	-3.950095374
PHTF2	0.270219313	2.746456573	2.030680244	0.045331406	-3.950209769
SNORD65	0.225370818	0.11009859	2.030139314	0.045387526	-3.95117147
RNY4P36	0.336135942	0.71333279	2.030032236	0.045398642	-3.951361812
MIR621	0.36311596	3.877645011	2.029783544	0.045424468	-3.951803856
HNRNPU	0.223880116	5.400091109	2.029747273	0.045428236	-3.951868321
CYP1B1	0.639429147	3.161064878	2.029518596	0.045451996	-3.952274739
AC104651.2	0.202580382	0.485128095	2.029482439	0.045455754	-3.952338996
RNU4-43P	0.224950081	0.09569829	2.02926631	0.045478222	-3.952723066
DEGS1	0.277432582	5.333091049	2.029150952	0.045490218	-3.952928049
RIN3	-0.293584147	2.476780159	-2.028690623	0.045538115	-3.95374591
CNNM4	0.271516298	2.290782625	2.028590004	0.04554859	-3.953924658
BAX	-0.266482932	4.264990767	-2.028518691	0.045556015	-3.954051338
LINC01137	-0.312963784	1.882718893	-2.028470516	0.045561032	-3.954136914
GRAMD2	0.247554287	0.505401049	2.028400484	0.045568326	-3.954261313
C11orf24	0.233054258	4.55133859	2.028389107	0.045569511	-3.954281521
RP11-568K15.1	0.241112308	1.139319603	2.028230104	0.045586075	-3.954563944
CHST13	0.680192369	2.455324008	2.028026877	0.045607253	-3.954924888
RP11-537P22.2	0.21615793	0.116089578	2.02775973	0.045635106	-3.955399308
IQGAP2	-0.309846232	1.170457842	-2.02768198	0.045643215	-3.955537374
HIST2H2AA4	-0.396195302	1.271970838	-2.027225828	0.045690814	-3.956347286

MIR518A2	0.232488346	0.085410179	2.026537253	0.045762746	-3.957569563
NEK4	0.200136961	1.715780783	2.026516543	0.045764911	-3.957606317
AL050321.1	0.233430169	0.255072525	2.02648117	0.045768609	-3.957669097
RNU1-17P	0.20837727	0.117105293	2.026429752	0.045773985	-3.957760351
NCEH1	-0.21911122	1.396199876	-2.025971499	0.045821921	-3.958573542
DHX36	0.223695115	2.588498546	2.025964226	0.045822682	-3.958586446
RNA5SP423	0.26170335	0.085710337	2.025960563	0.045823065	-3.958592945
TSTD2	0.236787305	1.471351882	2.025536373	0.045867477	-3.959345537
FBLN2	0.757999157	3.849504968	2.024770925	0.045947711	-3.960703225
CTNND2	0.328503478	0.647245811	2.024752137	0.045949682	-3.960736543
AC092657.2	0.209073946	0.130486305	2.024512041	0.045974874	-3.961162306
GUSBP6	0.208354046	0.160974191	2.024432181	0.045983256	-3.961303913
FAM192A	-0.214585955	3.320418059	-2.02429521	0.045997636	-3.961546776
ZNF736	0.218994787	1.148398748	2.024050936	0.046023289	-3.961979859
UBE2E2	-0.337273088	2.688077016	-2.023796022	0.046050074	-3.962431755
RPS6KA3	0.310974115	3.568083617	2.023602995	0.046070364	-3.962773908
TBC1D22A	-0.222423182	2.450189291	-2.023593842	0.046071327	-3.962790132
RP11-771F20.1	0.226119773	0.678674485	2.023038981	0.046129696	-3.963773494
GNAI1	0.475848937	3.610570912	2.02276707	0.046158324	-3.964255302
MIR7641-1	0.547950951	0.668538021	2.02253963	0.046182281	-3.964658267
MPV17L2	-0.274710514	3.024439648	-2.022385062	0.046198568	-3.964932099
MYCN	-0.22189843	0.476563399	-2.021855005	0.046254459	-3.965870998
CIC	0.340662409	3.949538489	2.021594404	0.046281958	-3.966332525
LMTK2	0.213206182	1.788617752	2.021556264	0.046285984	-3.966400067
AC092580.4	-0.268906361	0.364794647	-2.021516955	0.046290134	-3.966469678
RNU7-167P	0.228706628	0.097564229	2.021464539	0.046295668	-3.966562497
PLCB2	-0.335643705	1.695629232	-2.021027742	0.046341804	-3.967335902
AHDC1	0.349114815	3.44846073	2.020917926	0.046353409	-3.967530323
C17orf59	-0.405414449	2.196617419	-2.020737846	0.046372445	-3.967849117
AC005227.1	0.229272181	0.13672306	2.02051801	0.046395693	-3.968238258
NAGPA	-0.217555292	1.635974737	-2.020225408	0.046426651	-3.968756145
HOXB6	0.285899145	1.873024722	2.019940781	0.046456783	-3.969259853
RN7SL70P	0.251142799	0.131512178	2.0198732	0.04646394	-3.969379442

YPEL5	-0.225008593	4.197278186	-2.019677657	0.046484653	-3.969725449
RP11-767I20.1	0.227094796	0.234121972	2.019204004	0.046534858	-3.970563436
RP11-243J18.2	0.237448819	0.471852018	2.019078711	0.046548147	-3.970785075
RP3-428L16.2	0.50311302	2.060548262	2.018841392	0.046573325	-3.971204851
AP000266.7	0.236729881	0.310323155	2.018802256	0.046577479	-3.97127407
TOP2A	0.354329935	5.014307087	2.01871101	0.046587163	-3.971435454
CTD-2132N18.4	-0.249929313	0.516701668	-2.018575621	0.046601536	-3.971674898
RP11-820I16.2	0.285373627	0.160412011	2.018565261	0.046602636	-3.97169322
C9orf41	0.229489541	2.316803332	2.018105261	0.0466515	-3.972506652
JADE2	-0.243250923	1.638116431	-2.017761245	0.046688072	-3.973114875
RPL39P40	0.388868695	0.613237994	2.017529415	0.046712731	-3.9735247
RN7SL68P	0.232562601	0.530418004	2.017109586	0.046757416	-3.974266758
KIF5B	0.295613336	4.59761702	2.017053388	0.0467634	-3.974366078
ATOX1	-0.290671378	2.967044664	-2.017048622	0.046763907	-3.974374503
PRSS35	0.764733121	4.091017698	2.016850178	0.046785044	-3.974725198
DLG2	0.206267459	0.469138338	2.016772478	0.046793322	-3.974862504
SSR4P1	0.230127071	0.986072621	2.01668574	0.046802565	-3.975015775
FANCA	0.277580937	1.614393495	2.01625196	0.046848811	-3.975782201
IL12RB1	-0.239686553	0.733441671	-2.015919258	0.046884308	-3.976369934
RP11-180P8.5	0.212750704	0.36507405	2.01481984	0.047001771	-3.978311487
RNA5SP36	0.277045868	0.119869709	2.014465503	0.047039682	-3.978937037
CALM2	-0.251351497	5.807206986	-2.014407107	0.047045932	-3.97904012
AL450992.2	0.636556413	1.576262121	2.014396439	0.047047074	-3.97905895
CXCR2P1	-0.337785872	0.638379551	-2.014267712	0.047060855	-3.979286174
AC087738.1	0.243022458	0.11889674	2.013714931	0.047120074	-3.980261773
KIF21A	0.427682754	1.58933398	2.013000768	0.047196675	-3.981521835
RP11-419C5.3	0.312708151	0.223315443	2.012962478	0.047200785	-3.981589383
CARD10	-0.215304919	1.035909605	-2.012100608	0.047293378	-3.983109496
GLB1	-0.273981072	4.245934165	-2.011797257	0.047326005	-3.983644386
HMGB1P46	0.20574671	0.117175939	2.011709037	0.047335497	-3.983799929
CTPS1	0.328832259	3.676721083	2.01128779	0.047380843	-3.984542552
DPH3	-0.240348708	2.698368338	-2.011057926	0.047405604	-3.984947726
RP11-480D4.2	0.229259727	0.253479163	2.01102641	0.047408999	-3.985003275

RP11-269G24.4	0.202449148	0.492862819	2.011013125	0.047410431	-3.98502669
EIF4A1P12	0.204723153	0.216093259	2.010312922	0.047485929	-3.986260627
GABARAPL1	-0.290190713	3.368316588	-2.010180835	0.047500183	-3.986493356
RNU6-1102P	0.252717428	0.131833531	2.010173815	0.047500941	-3.986505725
TMTC3	0.214410366	2.340076025	2.010121538	0.047506583	-3.986597828
AL109653.1	0.201625494	0.07554788	2.009862881	0.047534509	-3.987053512
RP11-894P9.1	0.371672156	2.006220819	2.009727144	0.047549169	-3.987292621
MIR4753	0.38402195	0.405759795	2.009410701	0.047583362	-3.987850002
C1orf216	-0.21248159	3.279883407	-2.009406522	0.047583814	-3.987857363
NEURL1B	0.373012202	2.768982046	2.009270317	0.047598537	-3.988097248
CPLX1	-0.313003903	1.01288466	-2.009056027	0.04762171	-3.988474628
SAV1	0.281628007	2.860886374	2.00892698	0.04763567	-3.988701871
MIR4327	0.228169785	0.126796841	2.008899351	0.047638659	-3.988750522
MIR5690	0.350330908	0.396601856	2.008327942	0.047700516	-3.989756565
TBC1D4	0.285880704	3.039168376	2.008113927	0.047723702	-3.990133301
LMO7-AS1	0.503193627	1.302435095	2.008078495	0.047727542	-3.990195669
RNU4-35P	0.371319327	0.687140629	2.007187616	0.047824166	-3.991763489
GSTM2	-0.267325182	1.41571676	-2.006760221	0.047870581	-3.992515422
SGOL2	0.301572057	2.081495794	2.006593344	0.047888714	-3.992808975
RAB8A	-0.226655948	3.101475957	-2.00646335	0.047902844	-3.993037633
BX255923.2	0.264891479	0.439551184	2.006350859	0.047915074	-3.993235491
RBPJL	0.202717437	0.204346817	2.00627654	0.047923155	-3.993366206
RPL21P136	0.269276869	0.415300595	2.006234129	0.047927767	-3.993440796
RIMKLBP2	0.202605092	0.596771851	2.006032132	0.04794974	-3.993796042
RP11-114N19.3	0.209370444	0.47589989	2.005846974	0.047969888	-3.994121646
RNU6-1262P	0.251523442	0.524821465	2.005772198	0.047978027	-3.994253133
ALG8	0.259072515	3.632541064	2.005411992	0.04801725	-3.994886462
RP11-30L3.2	0.251723253	0.453326654	2.005366545	0.048022201	-3.994966363
RNU6-1318P	0.216439768	0.180714682	2.005332942	0.048025861	-3.995025438
RP11-5P18.11	0.202528466	0.064503002	2.005306785	0.048028711	-3.995071422
ASH2L	0.27804597	3.214728668	2.004757737	0.048088562	-3.996036539
AP000462.3	0.342462023	1.052864093	2.004727311	0.048091881	-3.996090013
MTBP	0.250357439	1.173725333	2.004436258	0.048123636	-3.996601523

RP3-473L9.4	-0.316371508	0.788403511	-2.004028023	0.048168206	-3.997318859
AC008069.2	0.21121557	0.12512231	2.003416084	0.048235082	-3.99839389
PLXNA4	0.21833977	0.377709196	2.00337859	0.048239182	-3.998459747
SCML1	0.316910051	1.797895012	2.003212274	0.048257374	-3.998751869
AMOTL1	0.405336162	2.848944888	2.002972676	0.04828359	-3.999172666
MRGPRF	0.697228274	1.528078518	2.002950636	0.048286002	-3.999211372
MAP4K5	0.256860675	2.849780022	2.002620964	0.048322096	-3.999790281
GUCY1A2	0.375014629	1.103364568	2.002371508	0.048349423	-4.000228274
AL596220.1	0.257022091	0.195113241	2.001976802	0.048392689	-4.000921194
TMEM60	-0.260817053	3.825433439	-2.001645615	0.048429017	-4.001502508
RNU6-888P	0.366514745	0.122291862	2.001097727	0.048489166	-4.002463995
COL13A1	0.571676617	2.71266299	2.000810544	0.04852072	-4.002967878
RPS29P2	0.201615294	0.10974307	2.000597389	0.048544151	-4.00334183
MED18	-0.243150113	3.215494354	-1.999976156	0.048612497	-4.004431496
WAC	0.211984162	3.395482408	1.999888229	0.048622176	-4.004585699
AC003988.1	0.203226474	0.328518	1.99986272	0.048624985	-4.004630433
RNA5SP399	0.246676244	0.205546681	1.999289325	0.048688154	-4.005635867
AL161908.1	0.243799411	0.140047015	1.999179372	0.048700275	-4.005828638
BORA	0.233149618	1.4553897	1.999014369	0.04871847	-4.006117903
STRN	0.216416686	2.538560315	1.998731936	0.048749627	-4.006612984
RPL13P6	0.241273255	0.529319272	1.998703419	0.048752774	-4.006662969
ING1	0.297723087	1.863241578	1.998006951	0.048829682	-4.007883533
TPM3P8	0.238534743	0.863793352	1.997794409	0.048853173	-4.008255938
RP11-647K16.1	-0.224295475	0.705730829	-1.997654847	0.048868604	-4.008500452
snoU13	0.338316649	0.627252804	1.997648885	0.048869263	-4.008510895
ITK	0.449600611	0.863240009	1.997629064	0.048871455	-4.008545621
RP11-49O14.2	0.241359955	0.862355095	1.997391452	0.048897736	-4.008961878
RP11-25B7.1	0.25147701	0.410797616	1.997383544	0.048898611	-4.008975732
EIF3D	-0.260675667	5.268920872	-1.996905179	0.048951559	-4.009813609
CTD-2555K7.2	0.235110036	0.438435331	1.996499076	0.048996547	-4.010524774
CYP51A1	0.267156701	1.135683486	1.996253284	0.049023793	-4.01095514
AC010311.1	0.22980015	0.297220989	1.996138636	0.049036506	-4.011155866
RNA5SP413	0.366167653	0.129536316	1.99597963	0.049054143	-4.011434236

DHX58	-0.300462147	1.572296628	-1.995912537	0.049061586	-4.011551689
RN7SL49P	0.224166869	0.489076114	1.995510411	0.049106219	-4.012255572
TXNDC12	-0.201572587	4.485400838	-1.995424146	0.049115799	-4.012406555
LSM14B	0.246064411	3.959996906	1.99541267	0.049117073	-4.012426641
PLSCR4	0.390251095	1.726934395	1.994923331	0.049171443	-4.013282974
DDIAS	0.220145892	1.436838319	1.994880406	0.049176215	-4.013358082
RPL7P52	0.217959352	0.380199678	1.994757837	0.049189842	-4.013572541
KHSRP	0.273528588	5.65675339	1.994727591	0.049193206	-4.013625461
LPP	0.252987877	2.5432609	1.994702652	0.049195979	-4.013669093
IFI27L1	-0.280418968	2.225315762	-1.99442415	0.049226959	-4.014156333
ALDH7A1	-0.273642313	2.819953234	-1.99442017	0.049227402	-4.014163295
KLF13	0.300226116	3.898134352	1.994216344	0.049250086	-4.014519849
CYFIP2	0.670458935	2.031838197	1.994067495	0.049266657	-4.01478021
CLSPN	0.335039179	2.504669548	1.993854678	0.049290358	-4.01515243
TUFT1	0.339332967	1.547752649	1.9938422	0.049291748	-4.015174253
AP2S1	-0.300319687	5.835273196	-1.993822531	0.049293939	-4.015208654
RP11-340I6.11	0.290563078	0.125526319	1.993822171	0.049293979	-4.015209284
RNF38	0.246156921	2.640860964	1.993708664	0.049306625	-4.015407791
CXADR	0.515136323	1.672484295	1.993472646	0.049332929	-4.015820523
PRAC1	0.306842129	0.202843721	1.993307559	0.049351334	-4.016109188
UTP3	-0.228927346	3.917367389	-1.993198663	0.049363478	-4.016299587
RNU6-1004P	0.390698279	0.688843518	1.993170962	0.049366568	-4.016348022
OACYLP	0.297985263	0.603081479	1.993024362	0.049382922	-4.016604326
ARC	0.30934083	0.770927018	1.992775786	0.049410662	-4.017038882
RP11-433M22.1	0.270849369	0.134176573	1.992504927	0.049440904	-4.017512336
REV3L-IT1	0.267035925	0.492985109	1.992396622	0.049453	-4.017701634
PLD3	-0.327254611	6.06683129	-1.992375802	0.049455326	-4.017738022
HSPE1P7	0.272812934	0.434409593	1.991516656	0.04955138	-4.019239312
YME1L1	0.234874757	4.181632671	1.991459479	0.049557778	-4.019339203
UFL1	0.248784111	2.727715627	1.991078577	0.049600419	-4.020004595
ANKRD36B	0.200060133	0.397912423	1.990691844	0.049643744	-4.020680054
FAM90A1	0.200058747	0.493453941	1.990637285	0.049649859	-4.020775336
HERPUD1	-0.26448486	4.110955716	-1.990248525	0.049693449	-4.0214542

YY1AP1	0.250279403	3.548962887	1.990217096	0.049696975	-4.021509076
RPS23P5	0.2636241	0.426960122	1.99000347	0.049720943	-4.021882059
RPL26L1	-0.339377443	3.933221437	-1.989975729	0.049724056	-4.021930491
TP53	1.121347768	3.883007178	1.989799087	0.049743883	-4.022238871
SUCO	0.307128281	2.870546899	1.989736402	0.049750921	-4.0223483
RP11-94B19.1	0.213929046	0.077248649	1.989511631	0.049776163	-4.022740654
RPL5P12	-0.276836016	1.118135507	-1.989286162	0.049801495	-4.023134188
IDUA	-0.320840594	2.011789288	-1.988998983	0.049833776	-4.023635371
RP11-153M3.1	0.249481979	1.015109423	1.98897651	0.049836303	-4.023674588
WDR82	-0.210556182	4.430040596	-1.98873757	0.049863176	-4.024091531
LETM2	0.226917558	1.031758974	1.988676931	0.049869998	-4.024197336
SARAF	-0.252119693	5.523443849	-1.988206036	0.049923001	-4.025018878
GOLIM4	0.284410544	4.673866677	1.988120771	0.049932603	-4.025167617
MANEA	0.213138477	1.650217422	1.987827728	0.049965617	-4.025678761
TAF4B	0.266233503	1.468510983	1.987613109	0.049989808	-4.026053071

Table S2. Potential prognostic genes identified in the training cohort (p-value < 0.05).

ID	KM_pvalue	HR	HR_95L	HR_95H	Cox_pvalue
ANXA5	0.03557234	0.420397926	0.196286843	0.900388498	0.025748239
ATP6V1E1	0.049105093	0.573283685	0.338769103	0.970142144	0.038181513
BTAF1	0.018128901	2.161298445	1.160348572	4.025696316	0.015157217
DLEU2	0.003123082	2.236812115	1.231254809	4.063601133	0.00821937
HMGCL	0.02887385	0.410662316	0.182639787	0.92336692	0.031332581
IFNGR1	0.032615795	0.379721423	0.164515537	0.8764422	0.023268251
LHPP	0.024523477	0.414822177	0.197755603	0.870152027	0.019915081
NPC2	0.002741643	0.611409875	0.418744999	0.89272	0.010846857
PLCB4	0.010465829	1.899903349	1.224044305	2.94893961	0.004220247
PLD3	0.001897011	0.40600305	0.219443529	0.75116581	0.004086015
SELPLG	0.011190205	0.635989475	0.45094155	0.896973482	0.009887602
SLC7A1	0.009278499	2.116380897	1.225514858	3.65484602	0.007155879
SMAD9	0.042806027	1.624726378	1.116978468	2.363282622	0.011129406
UHRF2	0.005393576	2.731663526	1.576974661	4.731836092	0.000337123
ZFP90	0.048226782	0.203007104	0.076698513	0.537323121	0.001323988

Table S3. Correlations that determined between the nine-gene signature and the 22 TICs by the Spearman coefficient.

TIC	r	p-value
B cells naive	-0.031988755	0.771334762
B cells memory	-0.111564132	0.309382468
Plasma cells	-0.089067199	0.417587696
T cells CD8	-0.374387032	0.000415769
T cells CD4 naive	0.416125486	7.47E-05
T cells CD4 memory resting	0.198608186	0.068430188
T cells CD4 memory activated	-0.300497265	0.005200901
T cells follicular helper	-0.212147088	0.051272949
T cells regulatory (Tregs)	-0.239137344	0.027511702
T cells gamma delta	0.057539749	0.600926011
NK cells resting	0.15627282	0.153227701
NK cells activated	0.041344767	0.707142363
Monocytes	-0.251936732	0.020018308
Macrophages M0	0.262106703	0.015608989
Macrophages M1	-0.072761503	0.508116473
Macrophages M2	-0.266972836	0.013735627
Dendritic cells resting	0.030832464	0.779386213
Dendritic cells activated	0.14979394	0.171212615
Mast cells resting	-0.042790681	0.697388957
Mast cells activated	0.341342571	0.001388518
Eosinophils	0.007665937	0.944487369
Neutrophils	-0.046040092	0.675650429

Table S4. The prognostic ability of 22 TICs tested by the Kaplan-Meier estimator.

TIC	P-value
B cells naive	0.348294868
B cells memory	0.814319965
Plasma cells	0.581366016
T cells CD8	0.021726632
T cells CD4 naive	0.008624662
T cells CD4 memory resting	0.36425876
T cells CD4 memory activated	0.10030296
T cells follicular helper	0.254635984
T cells regulatory (Tregs)	0.674650397
T cells gamma delta	0.184566798
NK cells resting	0.518362195
NK cells activated	0.890663643
Monocytes	0.329225779
Macrophages M0	0.885003231
Macrophages M1	0.534206839
Macrophages M2	0.802931997
Dendritic cells resting	0.093454413
Dendritic cells activated	0.635381386
Mast cells resting	0.641581025
Mast cells activated	0.000124234
Eosinophils	0.555229953
Neutrophils	0.470803973
Risk score	1.916e-07
